# Supplementary material for: Rapid Detection of DNA and RNA Shrimp Viruses Using CRISPR-Based Diagnostics
Source: Appl Environ Microbiol. 2023 May 23;89(6):e02151-22. doi: 10.1128/aem.02151-22 (PMC10304985; doi:10.1128/aem.02151-22)
Supplement: Supplemental file 1 — Supplemental material. Download aem.02151-22-s0001.pdf, PDF file, 2.4 MB [file aem.02151-22-s0001.pdf]

Supplemental material for

Rapid detection of DNA and RNA shrimp viruses using CRISPR-based diagnostics

Samuel R. Major[a], Matthew J. Harke[a], Roberto Cruz-Flores[b*], Arun K. Dhar[b], Andrea G. Bodnar[a], and Shelly A. Wanamaker[a#]

[a]Gloucester Marine Genomics Institute, Gloucester, MA, United States.

[b]Aquaculture Pathology Laboratory, School of Animal and Comparative Biomedical Sciences, The University of Arizona, Tucson, AZ, United States.

[#]Please address manuscript correspondence to Shelly A. Wanamaker (shelly.wanamaker@gmgi.org).

[*]Present address: Centro de Investigación Científica y de Educación Superior de Ensenada, (CICESE), Carretera Ensenada-Tijuana No. 3918, Zona Playitas, 22860 Ensenada, Baja California, Mexico.

Table of contents

Supplemental Protocol. One-pot SHERLOCK protocol for WSSV and TSV detection

Supplemental Figures
Figure S1. Effects of WSSV LAMP primer modifications
Figure S2. Target regions of guide RNAs screened for TSV and WSSV assays
Figure S3. Optimizing amount of non-target and target DNA for the TSV SHERLOCK assay
Figure S4. Comparison of synthetic and genomic target dilution series
Figure S5. Non-specific LAMP does not give rise to Cas cleavage

Supplemental Tables
Table S1. Consensus genome, gBlock oligos, primers, sgRNA, and reporter oligo sequences used for developing TSV and WSSV assays
Table S2. Composition of LAMP primer evaluation reactions, Cas sgRNA evaluation reactions, and one-pot SHERLOCK reactions, template dilutions, and 10x primer mix
Table S3. Viral concentrations estimated by qPCR, SHERLOCKv1, and SHERLOCKv2 assays

Supplemental Protocol. One-pot SHERLOCK protocol for WSSV and TSV detection

Materials

- Nuclease free water
- 10X Isothermal Amplification Buffer (New England BioLabs, Cat# B0537S)
- 100 mM $MgSO_4$ (New England BioLabs, Cat# B1003S, supplied with M0538L)
- 10 mM dNTPs (New England BioLabs, Cat# N0447L)
- Bst 2.0 WarmStart DNA Polymerase (New England BioLabs, Cat# M0538L)
- WarmStart RTx Reverse Transcriptase (New England BioLabs, Cat# M0380L)
- 500 mM Taurine (Millipore Sigma, Cat# 86329)
- 2 M Glycine (ThermoFisher, Cat# BP381-500)
- *Aap* Cas12b enzyme (synthesized by GenScript)
- 5'FAM/TTTTT/3'IBFQ reporter (100 µM, Integrated DNA Technologies)
- SYTO-82 Fluorescent Stain (ThermoFisher, Cat# S11363)
- Sample DNA or RNA
- 10 µM Guide RNA
    - Guide RNA can be prepared ahead of time and stored at -80°C before use.
    - Prepare guide RNA as follows:
        1. Amplify guide RNA template using the following oligos and conditions:
            - *Aap Cas12b sgRNA scaffold gBlock* 5'-TGTAAAACGACGGCCAGTcatataTAATACGACTCACTATAGGGGTCTAGAGGACAGAATTTTTCAACGGGTGTGCCAATGGCCACTTTCCAGGTGGCAAAGCCCGTTGAGCTTCTCAAATCTGAGAAGTGGCAC GTCATAGCTGTTTCCTG-3'
            - Cas12b_sgRNA_fwd primer 5'-CATATATAATACGACTCACTATAGGGGTCTAGAGG-3'
            - Cas12b_sgRNA_rev primer(s): Replace x's with 20 base target region 5'-xxxxxxxxxxxxxxxxxxxxGTGCCACTTCTCAGATTTGAGAAGCTC-3'
            - 1X HF Buffer (New England BioLabs), 200 µM dNTPs, 0.5 µM forward primer, 0.5 µM reverse primer, 0.01 ng gBlock DNA, and 1 unit Phusion DNA polymerase (New England BioLabs) in 50 µL.
            - Run reaction on a thermocycler (Applied Biosystems) with the following parameters: 98°C for 2 minutes, 30 cycles of 98°C for 10 seconds, 58°C for 30 seconds, and 72°C for 15 seconds, 72°C for 5 minutes, and a hold at 10°C.
        2. Gel purify PCR products on a 2% agarose 1x TAE gel and using the QIAquick Gel Extraction kit (Qiagen) following manufacturer's instructions.
        3. Transcribe guide RNA using the Ampliscribe T7 flash transcription kit (Lucigen)
            - In a 40 µL reaction, combine 50 ng of gel purified PCR product, 9 mM of each ribonucleotide, 9 mM of DTT, 1 µL of RiboGuard

RNase inhibitor, 4 µL of T7 RNA polymerase, and 1X transcription reaction buffer.
    - Run reactions at 42°C for 4 hours
    - Incubate with 2 µL of DNase I (Lucigen) at 37°C for 15 minutes
    - Purify using the RNA Clean and Concentrate Kit (Zymo Research) following manufacturer's instructions
    - Store purified guide RNA at -80°C.

- 10X LAMP Primer Mix
  - 10X Primer mix can be prepared ahead of time and stored at -20°C before use.
  - Prepare a 10x primer mix of the LAMP primers as follows:

10X Primer Mix			
**LAMP Primer** (100 µM)	**10x concentration** (µM)	**Reaction Concentration** (µM)	**Volume** (µL)
F3	2	0.2	2
B3	2	0.2	2
BIP	16	1.6	16
FIP	16	1.6	16
LF*	4	0.4	4
LB*	4	0.4	4
ddH$_2$O			56
Total			100

* If not included in LAMP primer set, replace with water

Procedure

1. Prepare sample DNA or RNA
   - For synthetic target, dilutions are made as follows:
     - For TSV, combine RNA stock containing 10^{10} copies TSV target, nuclease free water, and specific pathogen free shrimp genomic RNA (5 ng per 20 µL one-pot reaction)
     - For WSSV, combine DNA stock containing 10^9 copies WSSV target, nuclease free water, and specific pathogen free shrimp genomic DNA (20 ng per 20 µL one-pot reaction)
   - For genomic DNA or RNA extracted from shrimp, dilutions are made as follows:

Template dilution component	WSSV	TSV
Sample DNA/RNA	0.25 ng/µL (1ng)	2 ng/µL (10 ng)
Pathogen free shrimp nucleic acid (DNA for WSSV, RNA for TSV)	5 ng/µL (20 ng)	1 ng/µL (5 ng)
Nuclease free water	variable	variable
Total volume/one-pot reaction	4 µL	5 µL

2. Prepare the master mix by combining the reagents in the following order:

**One-Pot SHERLOCK Reaction component**	**Initial concentration**	**Final concentration**	**WSSV reaction volume (μL)**	**TSV reaction volume (μL)**
water			3.44	1.46
Isothermal Amp Buffer	10x	1x	2	2
$MgSO_4$	100 mM	8 mM	1.6	1.6
glycine	2 M	200 mM	2	
taurine	500 mM	50 mM		2
Cas12b	8.73 μM	200 nM (150 nM for TSV)	0.458	0.344
sgRNA	10 μM	200 nM (600 nM for TSV)	0.4	1.2
dNTPs	10 mM	1.4 mM	2.8	2.8
incubate at room temperature for 15 minutes to facilitate formation of CRISPR-Cas complex				
WarmStart RTx Reverse Transcriptase	15000 units/mL	150 units/mL		0.2
WarmStart Bst 2.0	8000 units/mL	320 units/mL	0.8	0.8
Primer mix	10x	1x	2	2
SYTO-82 LAMP dye	100 μM	0.5 μM (1 μM for TSV)	0.1	0.2
5'FAM/TTTTT/3'IBFQ reporter	100 μM	2 μM	0.4	0.4
Sample DNA/RNA			4	5
Total			20	20

3. Run on real-time PCR thermocycler at 62°C for WSSV or 60°C for TSV for 1 hour taking a reading every 2 minutes.

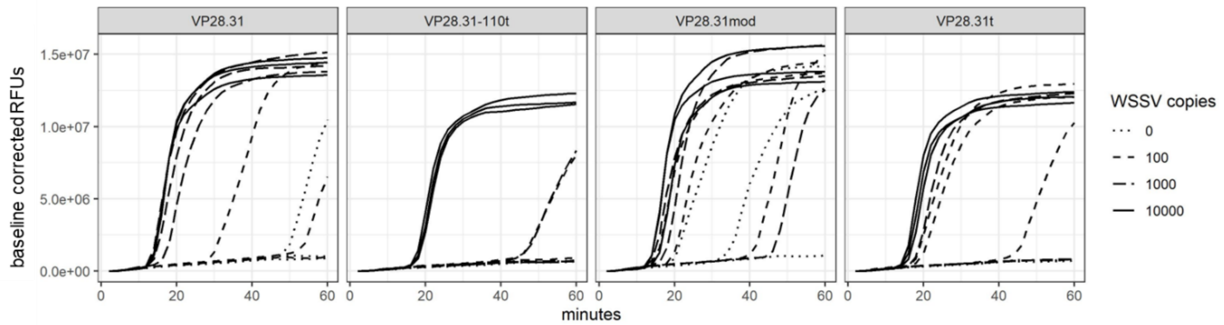

Figure S1. Amplification plots of triplicate LAMP reactions showing the effects of different modifications (indicated by plot titles and shown in **Table S1**) to the VP28.31 primer set for WSSV.

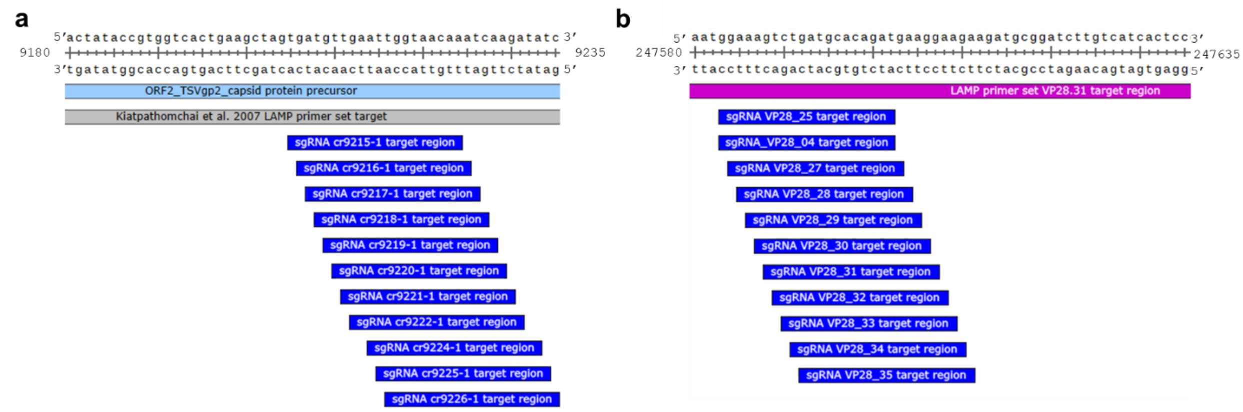

Figure S2. Diagrams of annotated consensus genome segments showing target regions of guide RNAs (dark blue) screened for **(a)** TSV or **(b)** WSSV.

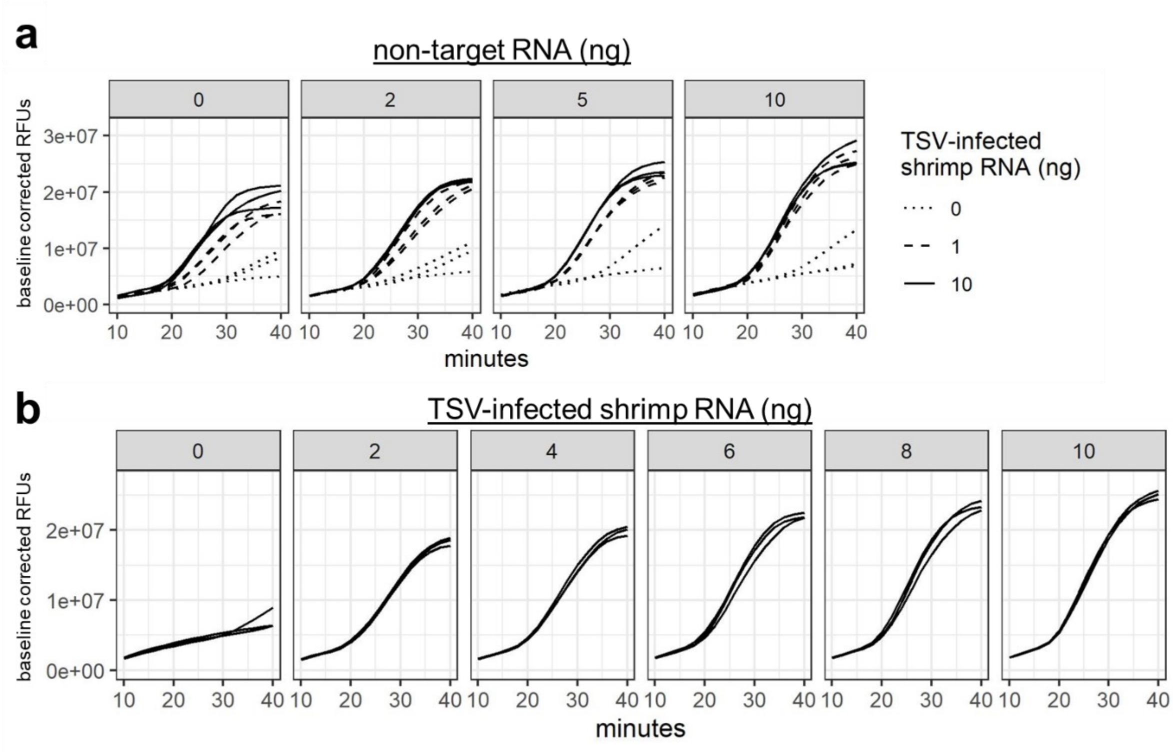

Figure S3. Optimizing amount of non-target and target RNA for the TSV SHERLOCK assay. (**a**) Cas cleavage activity plots of TSV SHERLOCK reactions containing varying amounts (ng; nanograms) of non-target RNA and varying amounts of target RNA (TSV-infected shrimp genomic RNA). (**b**) Cas cleavage activity plots of TSV SHERLOCK reactions containing varying amounts (ng; nanograms) of TSV-infected shrimp genomic RNA (gRNA).

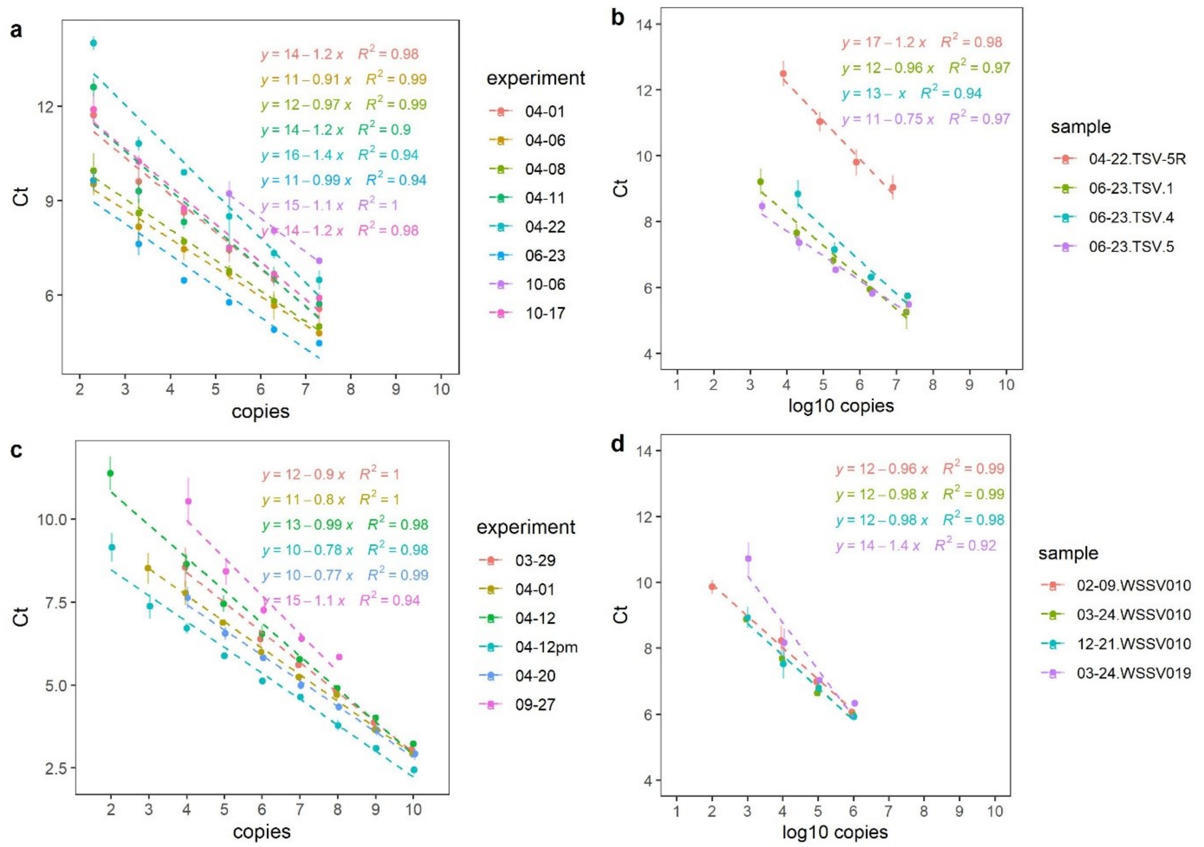

FigureS4. Comparison of synthetic and genomic target dilution series**.** (**a**) TSV SHERLOCK standard curves generated with synthetic target for eight different experiments. (**b**) TSV SHERLOCK curves generated with a 10-fold dilution series of genomic target isolated from samples with high viral load. (**c**) WSSV SHERLOCK standard curves generated with synthetic target for six different experiments. (**d**) WSSV SHERLOCK curves generated with a 10-fold dilution series of genomic target isolated from samples with high viral load. Linear equations are shown for each regression model (dashed line) with color corresponding to experiment number.

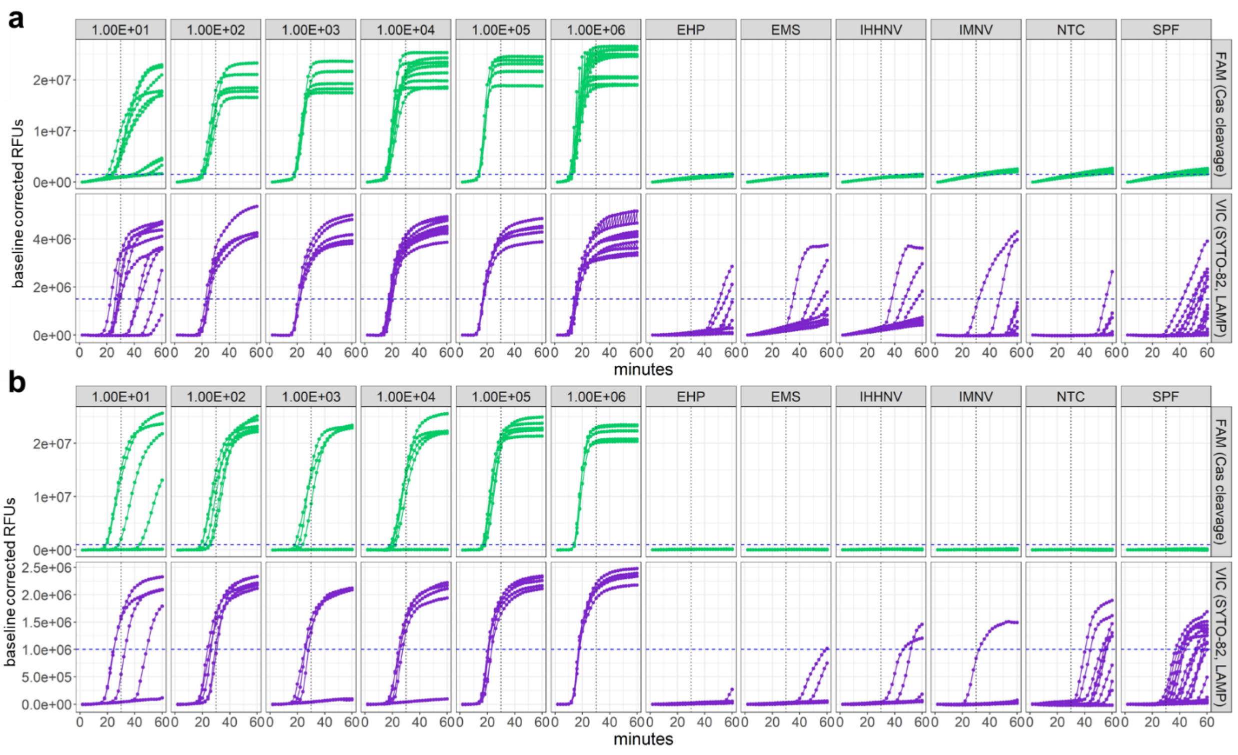

Figure S5. Non-specific LAMP signal does not give rise to Cas cleavage signal. (**a**) TSV and (**b**) WSSV SHERLOCKv2 assay fluorescence shows only samples containing target sequence produce a Cas cleavage signal (green) above the CT (blue dashed line), and non-specific LAMP signal (purple) produced in samples without target does not lead to Cas cleavage. Plots titled 1e+01 through 1e+06 indicate viral copies present in each reaction; 'NTC' signifies no template control; SPF signifies specific-pathogen free shrimp samples; EHP, EMS, IHHNV, and IMNV signify sample infected with nontarget viruses.

Table S1. Consensus genome, gBlock oligos, primers, sgRNA, and reporter oligo sequences used for developing TSV and WSSV assays. Bold indicates oligos used in final versions of assays.

Virus	Oligo type	Name	LAMP Primer Mix Name	Notes	Sequence (5'-3')
**TSV**	**LAMP Primer**	**TSV _F3**	**TSV**		**CAATTGAAATTCTGAGATTAGAGTC**
**TSV**	**LAMP Primer**	**TSV _FIP**	**TSV**		**CTAGCTTCAGTGACCACGGTATAGTTTTATTTTGAGTCCAAAGCTCCA**
**TSV**	**LAMP Primer**	**TSV_LF**	**TSV**		**TACCTGGAGTAAATTTCAC**
**TSV**	**LAMP Primer**	**TSV_B3**	**TSV**		**GGTACATATCGAGCCACTC**
**TSV**	**LAMP Primer**	**TSV _BIP**	**TSV**		**GCGAACCCATGCGGGTATAGTTTTCAATGCGACCAATGACTG**
**TSV**	**LAMP Primer**	**TSV_LB**	**TSV**		**ATGAAGAACCGCCAGTTAAGCAA**
TSV	LAMP Primer	TSV_P123L7_F3	TSV_P123L7		GTCCAGCTTTCACTTTTGG
TSV	LAMP Primer	TSV_P123L7_FIP	TSV_P123L7		ACAAATTCATGGCACCTCAACCCCTGCCGAATTGGCTTAA
TSV	LAMP Primer	TSV_P123L7_LF	TSV_P123L7		TGACGGGTCTACACCTGAAGG
TSV	LAMP Primer	TSV_P123L7_BIP	TSV_P123L7		TGGTGCATTTATCTCAACAGTTTTCTCTCTCAGAGTAAAAACCCTG
TSV	LAMP Primer	TSV_P123L7_B3	TSV_P123L7		CTCTAATAGACCTTAGTGAGCA
TSV	LAMP Primer	TSV_P73L39_F3	TSV_P73L39		TTCCAAGTCTTTCCCTCAA
TSV	LAMP Primer	TSV_P73L39_FIP	TSV_P73L39		ATCACCAGCACTTGCAAGCAATCAATTAGTAATTTGGAAAGGACG
TSV	LAMP Primer	TSV_P73L39_LB	TSV_P73L39		TCACTTTTGGACCTGCCGA
TSV	LAMP Primer	TSV_P73L39_BIP	TSV_P73L39		GACCACACTTTCTCTCAATTGGCCTGAAGGGTGTTAAATTAAGCC
TSV	LAMP Primer	TSV_P73L39_B3	TSV_P73L39		ACAATGACGGGTCTACAC
TSV	LAMP Primer	TSV_P215L25_F3	TSV_P215L25		GTTGAACAACAGTGATCCAT
TSV	LAMP Primer	TSV_P215L25_FIP	TSV_P215L25		TTGAGGTTGCAAAAACAAAAGATGGGTACATATGTCCAGTGTCAGG
TSV	LAMP Primer	TSV_P215L25_LB	TSV_P215L25		GATGCATTCAGACGGCGTTTAG
TSV	LAMP Primer	TSV_P215L25_BIP	TSV_P215L25		GCACGTATGTCCCCAAGTCTACTTTAACATCTACATAAACGCATAG
TSV	LAMP Primer	TSV_P215L25_B3	TSV_P215L25		CGATGCGTGCAAACTCAT
WSSV	LAMP Primer	VP28_F3	VP28	initial design	CTCCGCATTCCTGTGACTG
WSSV	LAMP Primer	VP28_B3	VP28	initial design	CCACACCTTGAATGTTCCCT
WSSV	LAMP Primer	VP28_FIP	VP28	initial design	GCCCAAGGTGTCGCTGTCAAACTGAGGTTGGATCAGGCTAC
WSSV	LAMP Primer	VP28_BIP	VP28	initial design	CTTGTCATCACTCCCGTGGAGGAAGGTGAGATTCTGCCCCA
WSSV	LAMP Primer	VP28_LF	VP28	initial design	GGACACATCAGTCATCTTG
WSSV	LAMP Primer	VP28_LB	VP28	initial design	GCCGAGCACTCGAAGTG
WSSV	LAMP Primer	VP28.1_F3	VP28.1	initial design	GGTTGGATCAGGCTACT

WSSV	LAMP Primer	VP28.1_B3	VP28.1	initial design	CCAGTGATGTTGATCTTTCTTGATG
WSSV	LAMP Primer	VP28.1_FIP	VP28.1	initial design	CCATTGCGGATCTTGATTTTGTCAAGATGACTGATGTGTC
WSSV	LAMP Primer	VP28.1_BIP	VP28.1	initial design	GCCGAGCACTCGAAGTCACACCTTGAATGTTCCCTCA
WSSV	LAMP Primer	VP28.1_LF	VP28.1	initial design	GGTGTCGCTGTCAAAG
WSSV	LAMP Primer	VP28.1_LB	VP28.1	initial design	GTGGGGCAGAATCTCA
**WSSV**	**LAMP Primer**	**VP28.31_F3**	**VP28.31**	**initial design**	**AGGCTACTTCAAGATGAC**
**WSSV**	**LAMP Primer**	**VP28.1_B3**	**VP28.31**	**initial design**	**CCAGTGATGTTGATCTTTCTTGATG**
WSSV	LAMP Primer	VP28.31_FIP	VP28.31	initial design	CAGACTTTCCATTGCGGATTGATGTGTCCTTTGACAG
WSSV	LAMP Primer	VP28.1_BIP	VP28.31	initial design	GCCGAGCACTCGAAGTCACACCTTGAATGTTCCCTCA
**WSSV**	**LAMP Primer**	**VP28.31_LF**	**VP28.31**	**initial design**	**ATTTTGCCCAAGGTGTCG**
**WSSV**	**LAMP Primer**	**VP28.1_LB**	**VP28.31**	**initial design**	**GTGGGGCAGAATCTCA**
WSSV	LAMP Primer	VP28.1_F3	VP28.110	initial design	GGTTGGATCAGGCTACT
WSSV	LAMP Primer	VP28.1_B3	VP28.110	initial design	CCAGTGATGTTGATCTTTCTTGATG
WSSV	LAMP Primer	VP28.110_FIP	VP28.110	initial design	GACTTTCCATTGCGGATCTTGACTGATGTGTCCTTTGAC
WSSV	LAMP Primer	VP28.110_BIP	VP28.110	initial design	AGGGCCGAGCACTCGAACACACCTTGAATGTTCCCTC
WSSV	LAMP Primer	VP28.110_LF	VP28.110	initial design	TGCCCAAGGTGTCGCT
WSSV	LAMP Primer	VP28.110_LB	VP28.110	initial design	GTGACTGTGGGGCAGA
**WSSV**	**LAMP Primer**	**VP28.31t_FIP**	**VP28.31**	**optimization**	**CAGACTTTCCATTGCGGATTTTTTGATGTGTCCTTTGACAG**
**WSSV**	**LAMP Primer**	**VP28.1t_BIP**	**VP28.31**	**optimization**	**GCCGAGCACTCGAAGTTTTTCACACCTTGAATGTTCCCTCA**
WSSV	LAMP Primer	VP28_110_BIPt	VP28.110	optimization	AGGGCCGAGCACTCGAATTTTCACACCTTGAATGTTCCCTC
WSSV	LAMP Primer	VP28_31mod_FIP	VP28.31	optimization	GCATCAGACTTTCCATTGCGGGTGTCCTTTGACAGCG
WSSV	LAMP Primer	VP28_F3	VP28_VP28.1	initial design	CTCCGCATTCCTGTGACTG
WSSV	LAMP Primer	VP28_FIP	VP28_VP28.1	initial design	GCCCAAGGTGTCGCTGTCAAACTGAGGTTGGATCAGGCTAC
WSSV	LAMP Primer	VP28_LF	VP28_VP28.1	initial design	GGACACATCAGTCATCTTG
WSSV	LAMP Primer	VP28.1_B3	VP28_VP28.1	initial design	CCAGTGATGTTGATCTTTCTTGATG
WSSV	LAMP Primer	VP28.1_BIP	VP28_VP28.1	initial design	GCCGAGCACTCGAAGTCACACCTTGAATGTTCCCTCA
WSSV	LAMP Primer	VP28.1_LB	VP28_VP28.1	initial design	GTGGGGCAGAATCTCA
**NA**	**Cas12b sgRNA scaffold gBlock**	**Cas12b_m13-T7-Handle**	**NA**	**Cas12b conserved handle with T7 promoter and m13 fwd (5’ TGT AAA ACG ACG GCC AGT) and m13 rev (5’ CAG GAA ACA GCT ATG AC)**	**TGTAAAACGACGGCCAGTcatataTAATACGACTCACTATAGGGGTCTAGAGGACAGAATTTTTCAACGGGTGTGCCAATGGCCACTTTCCAGGTGGCAAAGCCCGTTGAGCTTCTCAAATCTGAGAAGTGGCAC GTCATAGCTGTTTCCTG**

**NA**	**gRNA template primer**	**Cas12b_sgRNA_fwd**	**NA**		**CATATATAATACGACTCACTATAGGGGTCTAGAGG**
TSV	gRNA template primer	TSV_cr9215-1	NA		ttgttaccaattcaacatcaGTGCCACTTCTCAGATTTGAGAAGCTC
TSV	gRNA template primer	TSV_cr9216-1	NA		tttgttaccaattcaacatcGTGCCACTTCTCAGATTTGAGAAGCTC
TSV	gRNA template primer	TSV_cr9217-1	NA		atttgttaccaattcaacatGTGCCACTTCTCAGATTTGAGAAGCTC
TSV	gRNA template primer	TSV_cr9218-1	NA		gatttgttaccaattcaacaGTGCCACTTCTCAGATTTGAGAAGCTC
TSV	gRNA template primer	TSV_cr9219-1	NA		tgatttgttaccaattcaacGTGCCACTTCTCAGATTTGAGAAGCTC
TSV	gRNA template primer	TSV_cr9220-1	NA		ttgatttgttaccaattcaaGTGCCACTTCTCAGATTTGAGAAGCTC
TSV	gRNA template primer	TSV_cr9221-1	NA		cttgatttgttaccaattcaGTGCCACTTCTCAGATTTGAGAAGCTC
TSV	gRNA template primer	TSV_cr9222-1	NA		tcttgatttgttaccaattcGTGCCACTTCTCAGATTTGAGAAGCTC
TSV	gRNA template primer	TSV_cr9224-1	NA		tatcttgatttgttaccaatGTGCCACTTCTCAGATTTGAGAAGCTC
**TSV**	**gRNA template primer**	**TSV_cr9225-1**	**NA**		**atatcttgatttgttaccaaGTGCCACTTCTCAGATTTGAGAAGCTC**
TSV	gRNA template primer	TSV_cr9226-1	NA		gatatcttgatttgttaccaGTGCCACTTCTCAGATTTGAGAAGCTC
WSSV	gRNA template primer	Cas12b_sgRNA_rev_VP28_25	NA		ATCTGTGCATCAGACTTTCCGTGCCACTTCTCAGATTTGAGAAGCTC
**WSSV**	**gRNA template primer**	**Cas12b_sgRNA_rev_VP28_27**	**NA**		**CATCTGTGCATCAGACTTTCGTGCCACTTCTCAGATTTGAGAAGCTC**
WSSV	gRNA template primer	Cas12b_sgRNA_rev_VP28_28	NA		TCATCTGTGCATCAGACTTTGTGCCACTTCTCAGATTTGAGAAGCTC
WSSV	gRNA template primer	Cas12b_sgRNA_rev_VP28_29	NA		TTCATCTGTGCATCAGACTTGTGCCACTTCTCAGATTTGAGAAGCTC

WSSV	gRNA template primer	Cas12b_sgRNA_rev_VP28_30	NA		CTTCATCTGTGCATCAGACTGTGCCACTTCTCAGATTTGAGAAGCTC
WSSV	gRNA template primer	Cas12b_sgRNA_rev_VP28_31	NA		CCTTCATCTGTGCATCAGACGTGCCACTTCTCAGATTTGAGAAGCTC
WSSV	gRNA template primer	Cas12b_sgRNA_rev_VP28_32	NA		TCCTTCATCTGTGCATCAGAGTGCCACTTCTCAGATTTGAGAAGCTC
WSSV	gRNA template primer	Cas12b_sgRNA_rev_VP28_33	NA		TTCCTTCATCTGTGCATCAGGTGCCACTTCTCAGATTTGAGAAGCTC
WSSV	gRNA template primer	Cas12b_sgRNA_rev_VP28_34	NA		CTTCCTTCATCTGTGCATCAGTGCCACTTCTCAGATTTGAGAAGCTC
WSSV	gRNA template primer	Cas12b_sgRNA_rev_VP28_35	NA		TCTTCCTTCATCTGTGCATCGTGCCACTTCTCAGATTTGAGAAGCTC
WSSV	gRNA template primer	Cas12b_sgRNA_rev_VP28_04	NA		GGAAAGTCTGATGCACAGATGTGCCACTTCTCAGATTTGAGAAGCTC
TSV	gBlock	P215L25	NA		CCTAGGTCTAGGGCGGCacaagggaccttacgatgacccttagatccagctacgatgcgtgcaaactcatctttaacatctacataaacgcatagatctaaacgccgtctgaatgcatctgcactatgaatagacttggggacatacgtgcctggatttacgtttgaggttgcaaaaacaaaagatggtgctatgaatgtatttgccttatccctgacactggacatatgtacttgatatggatcactgttgttcaacctaataaattcgaataactcaggCCCTATAGTGAGTCGTATTACTCCTATTTTTATAGGTTAATGTCATGATGGTTTCTTAGACGTCGGAATTGCCTGT
TSV	gBlock	TSV-9098-9375	NA		CCTAGGTCTAGGGCGGCagatcgcgaagaatacgtctgataagcttattgggtacatatcgagccactctacggacaatgcgaccaatgactgattgcttaactggcggttcttcatctatacccgcatgggttcgcgggttacgctcatttactgtgatatcttgatttgttaccaattcaacatcactagcttcagtgaccacggtatagttacctggagtaaatttcactggagctttggactcaaaatctgactctaatttcagaatttcaattgtctcgtccacgaattgCCCTATAGTGAGTCGTATTACTCCTATTTTTATAGGTTAATGTCATGATGGTTTCTTAGACGTCGGAATTGCCTGT
TSV	gBlock	TSV-9807-10157	NA		CCTAGGTCTAGGGCGGCactaatgtctctaatagaccttagtgagcaggcatttatctctcagagtaaaaaccctgccgcgcaacgcgcggttagggaaaactgttgagataaatgcaccatgaatgacaaattcatggcacctcaacccacgcgtcacaatgacgggtctacacctgaagggtgttaaattaagccaattcggcaggtccaaaagtgaaagctggacatcctgccaattgagagaaagtgtggtcatcaccagcacttgcaagcacaaagtactcattttccgtcctttccaaattactaattgataaatcacttaattgagggaaagacttggaatcaataactaaattttctgctCCCTATAGTGAGTCGTATTACTCCTATTTTTATAGGTTAATGTCATGATGGTTTCTTAGACGTCGGAATTGCCTGT
WSSV	gBlock	VP28_gBlock	NA	synthetic target for WSSV VP28	CTCCGCATTCCTGTGACTGCTGAGGTTGGATCAGGCTACTTCAAGATGACTGATGTGTCCTTTGACAGCGACACCTTGGGCAAAATCAAGATCCGCAATGGAAAGTCTGATGCACAGATGAAGGAAGAAGATGCGGATCTTGTCATCACTCCCGTGGAGGGCCGAGCACTCGAAGTGACTGTGGGGCAGAATCTCACCTTTGAGGGAACATTCAAGGTGTGG

WSSV	gBlock primer	VP28-1_gBlock_B3	NA	primer for amplifying WSSV VP28 synthetic target for VP28.31 primer set	CCAGTGATGTTGATCTTTCTTGATGTGTTGTTCCACACCTTGAATGTTCCC
**NA**	**reporter**	**FAM_5bpDNA_Reporter**	**NA**		**/56-FAM/TTTTT /3IABkFQ/**
NA	reporter	FAM_FluorDNA_Reporter	NA		/56-FAM/TTTTTTTTTTTTTTTTTTTT/3IABkFQ/
NA	reporter	Fluor_DNA_Reporter	NA		/5HEX/TTTTTTTTTTTTTTTTTTTT/3IABkFQ

> TSV | consensus genome | tsv_consensus_20201004

TTTAAAAGTCGTGCGTGGCTTCACCACGCACGATCAGTACTATCAGTTAACCACTCTTGAATAT
GCTCAATGACCCTATTCAACACTGGTGTCTCTTAGTACATTATTTTAGCACTTAACGTGCATGA
GTTTTGCCCATTTCTTTCAAAAATGAGTATTCGAGGAGACGTCCCGCTCCCCGTCTTATTTCAA
CCGTAGACTCGACATCTATTGGTGGACATTTAATTCCAGTCGCCGTAAGTTGCTTCTGCCCCGC
GCTATATTTTCTTATACTTATGGTTCTATAGGTCTGGTTTAAAACGTAAATAGACGGCCCACAA
ACTATAGAACGCGTACCCGGAACGCCAATCCCGGATAAGTCCCTGGATATATAGATGCACCGCA
ATATAAGCCTGCAGACTGGCTCATATACTATGGCCTCTTATTATCTAAACATTAAAACCCACAA
CCTCCGCAGAACCCCTGTTGCCGACCGAGCCTTCTATGTGATGAACGATGATGGAGAAAACCGG
ATATATTCGTTAATTGGAACTTTGCGACGGGCCCCTGCCTTTAAGGTTGGTTCACGACGCTACA
AATCACACATTCCATACAGACGTGAAGCCACGTTTGCAGAATTGTGCAACCAGTTTCATGATAG
AGTGTTACCGTTTGCTAACCCTCGGGTCTGGAAAGAAGTTATTTCGGAGAATAAAGTGCAGCCT
GATTCAATGCTTAAGGCTGCATTTGACAACTGGGAAGAATGGCCAAAGGCTAAAGTATGTGAAG
AACTATACTCTGAGTGTGAATGTGGATATGTGGGCACCTGTTACGTGTCAGTCGATTGGTTGAG
GCCTCAGGCAACGAAGTGTAATGATTGCATTTTGAAGATGAATAGGAAAGTTGAATACCCATAT
CACACTATAGGAGTTTCAGGAAATGTTGTTACTAATACAGATATTGTTTATACAGGATATGCAG
ACGTCTTCAAGTGTGAACAGTGTGATTTGCTGATGGGTGCTTGGGCACCAAACGACATTCCAGC
ACTGACGCACAATATTCGAGCATCACAGTGTGTCCAGTTTAAGCTCCCCACAGAAAATTTAGTG
GCACGTAATTATGTGTTGTTGTGTGAGGAAATTGAGCGAGAAAATATTCCTGTAATTTTCCAGG
ATTATAGTGAAGGGAATGTCTTTACGTGTCGGATTGTTAGTGGAGATCTAACTGCTGTTGGTAC
AGCATCCAATATGTATACAGCTCGAGATGTAGCGTCTAAAAATTTGTTAGATCAGCTACATAAC
ACCCCCAATGTGCACATGCACTCTCTGCATTCCCTCCCGTATGAGAATTTCCCTTGTGAAGCTC
TGGAATTTGCAGTTGAGCAAGGTATTATCCCCCCTGTGACCTTTGATGAGGTATTTGCTAATGA
TGAATACGTTATTACTATTTCATGTAGCCTATTAGTTGTTGCTGACGTTGGCCCCACCCAAGCA
GTTGCACGAGAAAGAGCTGCAAAGAGATTTTTGAAGATGTATGATTATTCTGCAAGTTATCCTA
GTACCCACATGTTTACTTTATCCACACTCCCCCAGAGATCAGGTGAAACGCTAGAGTTGGCTAA
TGCCACATTGAACCATGTGAATAATGTGATTGACCGACACGATGAAGCAATAAGTAATGTCAGG
CAAAATGTTGAAGTGAAGTTGACGGATGTGTCCCGACAAGTTGGTGCTATGTTGCCGAAAGTAG
AAACTGCTATTGACGACGTATCTTCTACTCTATCTTCCTTTAGGGGAGTATTAGATAAGATTTC

AGCATGGATGCCTTCATCAAACCCTAAGATAATTGACCTCATTAAGGAGACTTTTGTATCACTG
TTTTTTGCTATTCTAACTAAGTCTTTGTATCCTATAATTCAGGGTATATCTAGTTATGCTCTTC
GTAACAATTTGATGGCTAACCATCTGACTGCCTTGTCAGAATGGTTAATGACACTCAAGTATGA
TTCACCGGATGAAGAGGAGATGCCAAGTACACACGGTTTCATGGATGACTTAACTAGTCGCCTT
CCAGGACTAAATGCTGCCAAGGCGCAGGCTGCGACTATATATGAGTCTATAGGAACAGGCTTAT
GCGTAGCATTGTCTGGTATATTGTCATTTATAGCAGTCATGTGTCTTGGAATCACTGATTTATC
TGCTGTTACATTCAATAAGCTGCTCACGCAGTCTTCATTGGTTGGACGAGCTTTAGTCGGTGTG
CGTAGCTTTAAAGATGTTTTCTTTGGCATCTGGGATTATGTAGACAATCAAGTGTGTGAAATTC
TTTACGGGAAAAGTCGCAAGAACTTGGATTTGTTGAAAGAATATCCGAGTTTGGACTCGTTGTT
GTCCATCTTCAACTACTTTCACGACACGGTAGATGCTAATGTGCTCATTAGTTGTAACCGTGCA
GCATGTGAGCTGTTGGTTAAAGCAGATAACCTGTACCAAGGTTACCTAGATAAATCGATAACTC
TAATGCACCGAGAGATTTCGTCGCGACTCAAGGAGGCACGCAATTCAGTTAAGGACTTAATTGC
AAAAGCTCAGGTCTATCTGACATGTGGTGATGGTAGTCGAGTTCCCCCGGTGGTAGTGTACATG
TATGGTGATGCTGGGTGTGGCAAAACAGAATTGTCGATGGCGTTACAGGATCACTTTGCAACTA
AGTGTTTTGGAGAAGTACCCAAGAAAGACGTGATATATTCCAGGAAAGCTGAAAATGAATTTTG
GGATGGTGTGAAGCAATCACATAAAATTATAGCTTATGATGATGTATTGCAGATAGTGGATTCG
GCCCAAAAGCCAAATCCTGAGTTATTCGAATTTATTAGGTTGAACAACAGTGATCCATATCAAG
TACATATGTCCAGTGTCAGGGATAAGGCAAATACATTCATAGCACCATCTTTTGTTTTTGCAAC
CTCAAACGTAAATCCAGGCACGTATGTCCCCAAGTCTATTCATAGTGCAGATGCATTCAGACGG
CGTTTAGATCTATGCGTTTATGTAGATGTTAAAGATGAGTTTGCACGCATCGTAGCTGGATCTA
AGGGTCATCGTAAGGTCCCTTGTGAACAGAAGATATGGCTCCATCAGAATCCAGGAAAGACGCA
GCAAGACATGAAGCAGGAAATTATTGCAGGAACATACAAGATCACTCCGGAGACAGCTGTGTAT
GAGTTGCATGTTGACACTACATTAGCAGGCAATGCTCAGTCTAAAGTCTGTGCTTATGATGGTT
TGGTGTCACTGATTGAACAGGTGAGGAAATTGCGTGTTGCGGCCCATAGCGATAAGGTGGAAAC
TGACGTCCCAGTCCTTCCCACTAGACTGCACGAGTTATCGCAAGAAACTTTTCCCAATACACAT
GCCGGTGTAGGATTTCAATTTGCAACTGATTGGTTGGGCGATTTCGATCGGCCAGTGGAAGCAT
TATCCTATTTAAATGAAACATTGGAAGCTCATTTTGTCTCGCGGAGTGCGAACGATGGAAGCAT
GTTCATCCCAGCCAGTGAGGTTGCTGATCTGTTGTGTCAGAGACATAACAATACGAATTTGAAT
GAGGAACTGGTGTATTTGACGTGGATGACGCAGATCACAGATAAGGAGTTAGCCTCGAGTTTAG
TATATTTCACAAATAACGGAATGGATAAGTCAATTTGGAAACAGAGCGCCGAGCGTTCAGCACA
GGCCATTAGTCAGTGTAAGAATGCTTGGACGCGCATAAACGATTTCTTGAAGAATCATTGGATT
TCCATATCTGCCGTTATAGGATCAGCTCTCCTAATAGGGGGAGTGTCGAGTGCAGTGAAGTGTG
CAACGAAGTGTAGGGTTAGGAAGATATTGCAGGATGGAGGTTCGATCATGCAACTTGTTGGTGT
ACGTTCATGTATGTACGCATGCCAGTTATGCAAACGCATCAAGAACGGTGATTTGCGCCTACGT
GTCCGAAATCGCTCGGAAGGTGTTACTACGTTTGTACCAGGTGATGTTAGGCGAGTTGCGCGCC
ACGTGATATCCGCTGCGGATGTGTGTGAAGTGCCTGTTCATCACTCATTTATACAGTCACTGTG
TGATGAGGCTTTTACCGTACATTCGGATAAGGAAGAAACGTTCTCCATCCTTGATTTCACCCCA
GAGGCGAAAGGTAGAAACCCTCTCGAAAGTGCTGTAGTTGAATCCCATCAGGACTATAGAGCTA
AGACTGCTGTGGTGGAATCCCATCAGGACTTTAAACCCAAGGGCGCAATCGTAGAATCTACCAG
AGACACTGTATTTACTGAGTCTCATCAAGACGTCAGGGTAAAGTTGCACCCACAAGTTGAATCG
CATCAGGACTTTAGAGCCAAAAATCCGACAGTTGAAAGTAGAAAACCAGACTATCAGGTGGAGT
GGACCGATTTGAGAACTGAATCTTCCGACGACAGAAACGCTCAAGACATAAGTAACAGGATCCT
ATCTAGGAATTTTGTGAGGTTATATGTCCCAGGATCAAGTCTATATACACATGGTTTATTCGCG
TATGGACGAATGTTGTTGATGCCTAAACACATGTTTGACATGTTAAATGGCAGTGTAGAAATAG

TTAGTATAGCAGATAAGGGTAACACTCGGGTTCACGTCAAGATACAATCCCACAAAACTGTGAC
AAGGGGTGGCTATGAAGTGGATATTGTAATATGTGAAATGGGAAATTCTATTTCAGCACGCAAG
GACATAACTTCATATTTCCCTACGGTGAAGGAACTTCCAGGATTAACAGGTATGATATCTTCTG
GGCGGATGAGAGTCTTTTCGACCGCTAAGTTCAAGGCATCAGATTCATGTTCGTACTTGATGCC
ACAAGACTTTGTAGCCAAGTACATTGCTGCAGTTGATCATATAACATCCAAGTCCCCAGAAAAG
AGAAGTTATTTTATACGACAAGGCTTTGAAGCAGAGAGTGATTCCATGCAAGGCGATTGTTGTT
CACCTTATGTACTGTTTAATTCAGCATCGAGAGCTAAGATTGTTGGATTACACTGTGCAGGATT
CGATGGAACAGCAAGAGTGTTTGCCCAGATAATTACTCAGGAAGACATAATGGCCGCCACGCCG
ACAACTCATGCAGGTCGCGTGACTACTGAATTTCCCCATACATCACTGCGGGATTCTCCTCTCC
CTAATTCAATGGCCATTGGTTCCGTTAAGACAGCACCCAATCCAACAAAATCTGAAATTACTCG
GAGTCCTATCCATGGATGTTTCCCCGTTCGTACAGCCCCCGCTACCTTGTATAGCCCAACAGAG
AACTTATTAATCAAGAACGCAATGAAAGTAACAAAGAATGTGGAGTTGCTGGAAGAAGACCTAA
TTGATGCCTGTGTTCATGACGTAAAGCGAATTTTGAATGCTCCAGGAGTGTCTGATGCGGAGAA
GAGAGTTTTGACGCATGAGGAATCCATTACGGGTATTGAGAATCGTCAGTACATGAATGCATTG
AATCGAAGCACGTCAGCAGGTTTTCCCTACAGTTCTCGCAAGGCGAAAGGGAAGAGCGGAAAGC
AGACGTGGTTGGGCTCTGAGGAATTCATTGTTGACAACCCGGATTTAAAAGAACATGTTGAGAA
AATCGTGGACAAGGCCAAGGATGGCATAGTAGATGTTAGCTTGGGTATTTTTGCGGCTACATTG
AAAGATGAAAGGCGCCCTTTGGAGAAAGTACAGGCCAATAAGACACGCGTGTTTGCTGCTTCAA
ATCAAGGTTTAGCCTTGGCACTAAGAAGATATTACCTAAGTTTCTTGGACCATGTGATGACGAA
CAGGATTGACAACGAGATTGGTTTAGGTGTAAACGTGTATTCGTATGATTGGACGCGCATAGTT
AATAAGCTTAAGCGCGTTGGTGACAAGGTGATTGCTGGTGATTTCTCAAATTTTGATGGTTCAT
TGAATTCTCAGATTTTATCACGAGTATCTGAAATTGTCACTGATTGGTATGGAGATGATGCAGA
AAATGGTCTGATCAGACATACACTACTTGAGTACTTGTTTAATGCCACCTGGCTTATGAATGGT
AAGGTTTTCCAACTCAACCATTCTCAGCCTTCCGGCAATCCATTAACTACTCTCATCAACTGTG
TATATAACATGATCATTTTTAGATATGTCTACCTTCTAGCTCAGCGAGAAAACGGGTTTCCCAT
GACGCTCTCTGGATTTACTACAAACGTAGCTTGCATTTTCTATGGTGACGATTCATTGTGTAGT
GTGTCAGATAAAGTGAGTGAATGGTTCAACCAGCACGTAATAACCCGATTGATGGCTGCTACTG
GACATGAATACACGGACGAGACTAAGAGTGGTTCCCCCCCCCCATACCGCTCCTTAAGTGAGGT
TACCTTTCTCAAGCGTGAGTTTGTGCTAAGAGATCATTTTTGGATTGCACCCCTATCCCGGAAT
ACGATTGAAGATATGTGCATGTGGAGTAGAAAGAATATCGATGCGCAGGATGCATTACTGCAAA
CAACGCGCATTGCTTCTTTTGAGGCTTCGCTGCATGAGAAGAGTTATTTCTTAATGTTCTGCGA
TGTCATTAAGAAAGCGTGTAGGAACGCAGGGTACAAGGAAGCATGTTTACATGAGTTGGATTGT
AAGAGCTTCCTTTTGGCCCAGCAAGGTAGAGCTGGAGCTCATGATAGTGAGTTCCTAAGTCAGC
TATTGGACTTAAACTAATAGCACCACCCGATCGTAAACTCCATGTATTGGTTACCCATCTGCAT
CGAAAACTCTCCGAACACTAGGTGCAGTAAGGCTTTCATGGAGTGGTTTGCTATTTAGCGTACG
TGTACCATAGGCAGCCCCAAAAACACGTGTGAGGAGAAAGTCCCAGTCACTTTGGGCAAAGTAG
ACAGCCGCGCTTGCGTGGTGGGACTTAATTAATGCCTGCTAACCCAGTTGAAATTGATAATTTT
GATACAACAACCAGTGGAGGACTAATTCCAGGAGGTAGTGTTACAAACAGTGAAGGTTCTACAA
TCTTGATGAATGATATCCCAATCACTAATCAGAATGTAGTGCTGTCTAAGAATGTAACAGATAA
CCTGTTTGAAGTCCAGGACCAAGCTCTCATTGAATCTCTCTCTCGCGACGTTTTACTTCATAAC
GACAGTTGGACATCTAGTGATGATGAAATTGGCACAACTATGACGCAGGAACAGCTTGCAACAG
AATTCAATCAGCCACACTTATATGAAATTTCCCTACCTGATGACATTGTACGTAAATCGCTGTT
TATGTCTAATAAATTAGCGAATATTGCATATATGCGATGTGATTACGAGGTTACTGTACGAGTA
CAAGCCACGCCCTTTTTACAAGGAGCATTGTGGCTGTGGAATAAGATGAATGCTAAGCAGACAT

CAATTATTCGACGCACTCTTACAGAACACCTACGCTCTATTACATCATTTCCTGGCATTGAGAT
GAACTTGCAGTCTGAAGCTCGAGCTATTACTCTTTCGATTCCTTATACTAGTGAGTTGCAGGTT
TTTAACCCCAGAAATGTGAATAACCTAAATTCTATTCGGCTTTCCGTTCTGAGTCAATTGCAAG
GCCCTGAAGATGTAGAATCCGCATCTTATTCTATTTATGGCAGGTTGAAAAACATCAAGCTATA
TGGGCATGCCCCATCTGTGACATCTTCAGTATATCCGTCTACTCAGTCCGGATATGATGATGAT
TGTCCCATTGTGCATGCGGGAACTGATGAGGATTCTTCTAAACAGGGGATTGTCTCAAGGGTTG
CAGACACCGTTGGTGCGGTGGCAAATGTAGTAGATGGGGTAGGAGTACCTATTCTATCCACAAT
TGCCAAGCCTGTTTCCTGGGTGTCGGGCGTAGTGAGTAATGTAGCTTCAATGTTCGGATTTTCA
AAAGATAGGGATATGACGAAAGTCAACGCATATGAGAACTTACCTGGTAAGGGCTTCACTCATG
GTGTTGGCTTCGATTATGGCGTACCCCTGTCTCTTTTCCCTAACAATGCCATTGATCCCACAAT
TGCAGTGCCTGAAGGATTGGATGAGATGTCTATTGAATACTTAGCACAGCGACCATATATGCTC
AACAGATACACTATCAAAGGTGGTGACACTCCTGATGCGCATGGAACAATTATTGCAGATATTC
CAGTGAGTCCTGTCAATTTTAGTTTGTATGGTAAAGTTATTGCTAAGTATCGCACCCTATTCGC
TGCCCCAGTTAGTCTAGCTGTAGCAATGGCCAATTGGTGGCGTGGAAATATTAACCTTAATCTT
CGCTTTGCTAAGACGCAGTACCATCAATGCAGATTGCTGGTGCAATATCTCCCCTATGGTAGTG
GTGTTCAACCAATAGAAAGTATCCTTTCACAGATCATCGACATCTCACAAGTCGATGATAAGGG
TATTGACATTGCTTTTCCTTCCGTCTATCCCAATAAGTGGATGCGAGTGTACGATCCAGCGAAA
GTTGGGTACACGGCAGATTGTGCCCCAGGCCGAATCGTCATTTCCGTTCTCAATCCACTTATCT
CAGCTTCGACAGTCTCTCCTAATATTGTCATGTATCCTTGGGTGAATTGGAGCAATTTAGAGGT
TGCTGAACCAGGTACGCTTGCTAAAGCAGCCATCGGCTTCAATTATCCAGCAGATGTTCCTGAG
GAGCCCACTTTTTCAGTAACGCGTGCTCCAGTATCTGGAACACTGTTTACGTTACTCCAGGATA
CGAAGGTGTCTTTGGGGGAAGCTGACGGTGTATTCTCATTATACTTTACGAACACTACCACTGG
TAGAAGGCACAGACTAGCTTATGCCGGACTGCCTGGTGAACTCGGTAGTTGTGAGATAGTGAAG
CTACCTCAAGGGCAATATTCAATTGAATACGCAGCTACCAGCGCTCCAACTCTTGTTCTCGATA
GACCTATCTTTTCTGAACCGATTGGCCCCAAGTATGTAGTCACTAAAGTTAAGAACGGTGATGT
TGTTAGTATTTCCGAGGAGACGTTAGTAACATGTGGTAGTATGGCAGCGATTGGTGGAGCTACG
GTCGCATTGCAATTCGTGGACGAGACAATTGAAATTCTGAAATTAGAGTCAGATTTTGAGTCCA
AAGCTCCAGTGAAATTTACTCCAGGTAACTATACCGTGGTCACTGAAGCTAGTGATGTTGAATT
GGTAACAAATCAAGATATCACAGTAAATGAGCGTAACCCGCGAACCCATGCGGGTATAGATGAA
GAACCGCCAGTTAAGCAATCAGTCATTGGTCGCATTGTCCGTAGAGTGGCTCGATATGTACCCA
ATAAGCTTATCAGACGTATTCTTCGCGATCTATCGCAATCTCCATGTATATATCCATCCACACA
TGCTGGTCTGGACTACTCCAGCTCAGACACATCTACAATGTTGACTACAATGGGTGAACAGTTT
GTCTCTCTCAGAATGTTAACTAGACGTTCCAGTCCCGTTGACATTCTTAGAGGCGATTTGGTTA
CTTTGCCCGGAATTTCCTTTGGCACAGATAATTCATTACGCCAGAGCTTAGTTAACATTATTTC
ATATATGTATAGATTTACTCATGGTAGCATATCCTACAAAATTATTCCTAAGAATAAGGGCGAT
CTATATATTACTACAACGAGCCCAGATTCGATCGAAACTAGTACTAGTGCTTATCAGTTTGATA
CTAACCGTGCTATGCATTATATTAACACATCCCTGAACCCTATGGCTCAAATTAGCTTGCCTTA
TTATAGTCCAGCAGAAAATTTAGTTATTGATTCCAAGTCTTTCCCTCAATTAAGTGATTTATCA
ATTAGTAATTTGGAAAGGACGGAAAATGAGTACTTTGTGCTTGCAAGTGCTGGTGATGACCACA
CTTTCTCTCAATTGGCAGGATGTCCAGCTTTCACTTTTGGACCTGCCGAATTGGCTTAATTTAA
CACCCTTCAGGTGTAGACCCGTCATTGTGACGCGTGGGTTGAGGTGCCATGAATTTGTCATTCA
TGGTGCATTTATCTCAACAGTTTTCCCTAACCGCGCGTTGCGCGGCAGGGTTTTTACTCTGAGA
GATAAATGCCTGCTCACTAAGGTCTATTAGAGACATTAGTATGATCCGGCTAATAGTCGCTTTG
GATGACCTCCAAAGGGC

> WSSV | consensus genome | WSSV_Consensus_Genome

atggcgtacattgaccaaggggcgtttggagccaaatctgtgatgcagcaacaataccttcttc
caccctctaataggctctccaaacaacaacctccactggcatcatcttctcttcaaccttcctc
ttctaataaacctaggagtacatcaagagtaacagacatatttgttgtgataacttgtgttgtt
ttaattgctgcttttataagcaactcatttagtgttacaaaaaatgtggtaaaactttctaaag
aacaaaccgagaaaatactagaaaaagatttgcctgataaggtgtacaaattgtttgaaaattt
aaaggatggaacatttggtataggggaagatgaggaggaagaaagggaggagagggaggaaggg
gaggaagagcttgaaacaaaaaacataagacttaaaagaaagtggaaagaaatgactgaacaag
gggatcaaggaataaaagtaaggaaattacatggcccgagaggagaaagaggagaaactggtcc
agcaggagcagttggccctgcaggccctcaaggagaaagaggagcaattggaccggcaggaaag
gatggagcagttggccctgcaggccctcaaggagaaagaggagcaattggaccggcaggaaagg
atggagcagttggccctcaaggccctccaggagaaagaggagaaaatggacgcccaggaagaga
tggagcagttggccctcaaggagaaagaggagcaattggaccggcaggaaaggatggagcagtt
ggccctcaaggagaaagaggagcaattggaccggcaggaaaggatggagcagttggccctgcag
gccctcaaggagaaagaggagaaaatggacgcccaggaagagatggagcagttggccctgcagg
ccctccaggagaaagaggagcaattggaccggcaggaagagatggagcagttggccctgcaggc
cctccaggagaaagaggagcaacaggtataccaggaagggatggcgtggacggttctgtgggcc
ctcaaggagaaagaggagaaattggacgcccaggaagagatggagcagttggccctgcaggccc
tcaaggaagaagaggagcaacaggacgcgcaggaaaggatggtgcagttggtcctgcaggccct
caaggagaaaaaggagaagctggtaaggacggttctatagggcctcaaggaatacaaggcccaa
gaggagagactggaccaccgggaagggacggcactgcagcagaaagaggagaaagaggcttccc
aggaccaccaggcgaaactggaccaccaggaaaggatggtgtggatggttctgagggccctcaa
gggaaaagaggagaaacaggacccgttggacctaggggtgaaccaggtctagctggcctcccag
gaagagatggagcaattggccctgcaggccctccaggagaaagaggagcaactggtctaccagg
aaggaatggtgtggatggttctatcggcccccaaggaagaagaggagcaacaggccgcgcagga
aaggatggggcagttggccctgcaggccctccaggagaaagaggagcaacaggtataccaggaa
gggatggtgtggacggttctgtgggccctccaggagaaagaggagaaactggaccagcaggaag
ggacggttcagttggccctgctggccctcaaggagaaagaggagaaaatggacgcccaggaaga
gatggggcaactggccctataggtcctgctggtcctcaaggagaaaaaggagaaaatggacgcc
caggaagagatggagcaactggccctataggccctagaggagaaactggtgcaatgggaaagaa
tggcgtggacggttctatgggtcctcaaggaagaagaggagcaacaggccgcgcaggaaaggat
ggggcagttggccctgctggccctccaggagaaagaggagaaactggaccagcaggaagggacg
gttcagttggccctgctggccctcaaggagaaacaggattaactggcagcccaggaagagatgg
agcaactggccctataggtcctgctggccctcaaggagaaaaaggagaaaatggacgcccagga
agagatggagcaactggccctataggtcctgctggccctcaaggagaaaaaggagaaaatggac
gcccaggaagagatggagcaactggccctataggtcctgctggccctcaaggagaaacaggatt
aactggacgcccaggaagagatggagcaactggccctataggtcctagaggagaaactggtgca
atgggaaagaatggtgtggacggttctacgggtcctcaaggaagaagaggagcaacaggccgcg
caggaaaggatggagcagttggccctgctggccctccaggagaaagaggagaaaatggacgccc
aggaagagatggagcaactggccctataggtcctgctggccctcaaggagaaacaggattagct
gggctgccaggaagagatggagcaattggtcctcaaggagaaaagggagaaaatggacgcccag
gaaaggatggggcaactggccctatgggtcctccaggagaaaggggagagactggtcctatagg
tcctgctggccctcaaggagcaactggtcttccaggaagggatggtgtggatggttctgttggc

cctcaaggaaaaagaggattaatagggcgcacaggaagggatggggcaattggccctgtaggtc
ctgcaggccctaaaggagaaacaggattagctggcctgccagggatagatggaaaggacggttc
cgtgggtcctcaaggagcaattggacctataggcccacgaggagaaagaggagaaactggacga
ccaggaagggacggtgaggatggttccacaggccctatgggcccccaaggactaagaggagcta
cgggagctccaggaccgcaaggagaaagaggattaaagggacggccaggaaaagatggtgaaac
aggtcctccagggcgacaaggaagggatggaataatgggtcctaggggtcttcgaggagaaaaa
ggagcacctggtaatgatggtctagagggacctgaaggaagagatggtgcacctggtcccgctg
gccctattggacctcaaggaataagaggattaaaaggtatccagggacgaccaggaagagacgg
agaaatgggaccagccggcaaggacggaatagaaggccctagaggtcaagatggaacaactggc
gctaaaggacctagaggattaagaggttttcaaggaagaacaggagaaactggtgcacaaggat
ctagaggagaaaaaggcgatagagggctaacaggccctcaaggaagagacggtccacccggtga
agaaggtcctcaaggtcttagaggagaaaggggagcacctggccctagaggtcctagaggtatt
cgtggccgttcaggacctcaaggaagtaacggcgtgcaaggacctcgaggtccccgaggaacaa
aaggaagaacaggaatacaaggcctcactggcatagaaggtcctcgaggtcctagaggtataca
aggaaaggaaggaagaatggggaaaattggacatcgaggagaaaagggtgataaaggagaccgt
ggagaacaaggcatcgctggagcagacggggaaaaaggtccaagaggtttacgaggaattcgag
gccctattggtgctcctggtaagcctggcacggaaggggttagaggtcctagaggggtgagagg
tgttcctggctatcctggcgcacaaggggaattaggtccccaaggaccaacaggtcctcaaggg
ccagcaggtcctcaagggccgatggggcgtacaggagatactggtcccatgggccctcctggag
cagtgggaccaagaggagagaaaggaggtagaggaagaaaggaaaaaatggccctaaaggagc
ggacggaaaagatgccgtaaatatcatacaaaaatattcaatcacccatgctcgtgcagagata
atgtgggaaggaaatgaaatcggagaagcatacattggaagatcttatggaactgatacaatcc
ctgtgatgatagaaaatagaatagggatgacaaatgaggacaaaaaaaacgaatattgtataca
agtaatgacaatgcactcaataacaactagaggaagaacatcgggtgtttttgtggtaagcaat
aagacagattatatccttttagttactttactgatgccagaaagtgtttcctgtagaacagatg
tcagtacaaatgcgaggtcagagagggtgaatgctgttagagaaagagaaagcaaatcgtacag
atttattaggccgtctgaccaatctataggtactcattcacgttcaaaaattgccgtggtaatg
tatccagacgcaagcatgagttactcagttgatacattagacgctgatgtggcgcgaagagaaa
caacgtctgtgcttttattagcagaaaccatacacggggaaaaagatagaggtttctatgctga
tagaggaactgtagggaggttgatggtacctcccactgaagaagagttattggtattgcaagcg
gatccagacatcaccccaacaatcgcagacccgattacagtagcagaagttggaaaccctgtcg
tgaggataaaaactccttcatgtgatgagttgaataaattcatgagattctctactaatttctc
atttttaaaatacgtcaaacggtttatggcgcagagagcttcatctaatagtaggaatgatatg
tccgctttcttattagatttgatttttgaaaaattgatagagtttttattatcaacaggatgga
atagagaagctctgggaggaactactggggctattcttatgatcaggacggcaacttgtcgtct
agcgcaatcttattacccccctccctgaaaccgttggtatggactttttgcaaacacctgggttt
gactacaataaatctttccctaacaatgaaaggtatatatatgaatttcaagctacaatgtaaa
taattggttaataaaataaaggtatatttttaaaaaatgtgtttatttttccccaaccttaaac
agatcattgccaggagaaaatcgcatacttaacagataatgcctatttgtatcatcgatgtctt
cgtcaaacgttgctccaaacacaagtgtgttgatcctatttttcttcaaaactgttggtttgaa
atataatacaaatttgttagctgaaaattcccttcctgtagcatttagacctggagacagtgaa
acagagtccactgttatatccctctttgtattattgtatactcttgcatacaattctaattctt
tagattcggctttgtttacatttagtgtgtggtctccgtctccaagtaaagatgtgaggattat
gtctcctttctttttaccagttcagaatcggacgttccttggccagctacatatacctttcca

tagttcctggtttgtaatgtgccgtttacaaagttaaagtggtttcctctagcaacaccgttaa
tggtcaagtttattatttcagattcaacagggtaggcgtctttatccttcttgtccaatttctt
gttaagttctatgttggttacaactatagatataaccacgagtatcaatgtcaaacccgccagt
atagcggcgtaaacccccacatgtgcattttgaagaaagttgtacaaaggtcatgagagaaaa
aaacagatattaaagtttgttatatttatttagtcgtagaaatcaccactgagatatattgtg
agcagcaactgccgtttcagcatactcgaaagaagaatagttgcgatcctttgaagccggcatc
ttgaaatgatgtgcagtattatttctgctagttttgttgaagtactggatagcctttactgagt
tgaaagtttcacgggaacagcttgaacagttgatggaggaagaagttcgcctgcactggcaatt
cacaaaggagtgctgattattgttgttatgatggtacattttgaaggtgagagcacgtctttct
tcaaccaaaggtgtttgtctaatgtggctcaggtacatccacgctcaccttttataccaacgtc
ttacagttccttcagatcttccgtcatggctttaatcttgttggttatccctcgatctacgaag
aatctaatatctccctcagatctgttcaaaactttccacgactccattgggtccagagtataat
ctgacctttctatgtttttgacaactacaggatctggttgaatatatgcagatgatgcatcaaa
atctacttcttcgccgctcatgcctgtttctagtcttgaaaggagtgtgtccaaatcttcctct
tcttcgtcttcttccatttcttctccactatctaaaaattcgtctatatcattatcatcaaagt
ctatgtctatatcatcatcatcatcttccccaagagatgaagaaggagatggggttcgtgtcgg
gggaggtggaggagtaggagtaggagtaggtggaggagtaggagtaggagtaggaggggggtgga
ggtaacaaaccttctccaggcacagttcctgttcctccacctcctgtattggacccagtattta
cactcggttgttcaaattcaaaaacttcctcctcttcttttccgtcttctacagcaccttcctc
gtctcttccgatcggcctattttcaggttcccttcgccctaatacagctccttcggtgtcatct
tctttttccttctctttatctttctttgtttttttattggatgttgtagtagttttatttggtc
gtgacatgaaaatcacaccaaccaccacacccacaaaacacacaaaaagtatgagagataataa
aaggaacatcattattatccttatttttttacattcaattatggcaccagaaaaataaaatgat
ttgtacaaacacgagttacccctccacacgcctgacctctgccgcgaattcttcatacccttcc
atattctttgctgcaaaggtcacgtcatgttgatgagctaccatttcatatcgcatatctcgcc
aaaaagatccatccgcaacagcggccgtgcattcttcttcaatggcagaagtcatgtcagctgt
catttttttaataagatcacgtagtatttcctccctatacaaatctataaccttctgcctaaag
acaggcctctctgctaaaagagaaggattttctgtaagctggtccatggtgtaataaagtgttc
atttatctaacttttaaaacccaaactcttctaaaatatctacagattccaggtactctgccgc
cattgttgtaatcattgtatttgccttgtctatcactgcttgtcttttatcttccacatctccc
acctcgtcttcgtgtttttttcctttcttcttctagaagccgtctaaagacagcttctctaattg
aaggtattagcccctcaactacctgagaagcttcctgttttacatcattcacaaattcgggcga
ttgtagtacgagcgtgatcgcgctcttgtagatgtcttccaaaatgtccatttttgttgtttag
acctagatagagagtgggagacgctattaatttttttgttcatgtggctcatgacctcatgtcc
gatatatgtattggttgggtgtaatacaagtctctacaaagcaatagaaggggtaaaaacaaaa
cacaccatggtgctaactctttcctgtacgactaggcgcgtagcgtctagcaaggggaatttct
ctaaggaagatgcggtgttggggaaccagttccccattttaaagaaatcaaacaacttgtcaat
tgcaagacctccctcaatagaatctttttctgcatcagtggaaaaaatattcaggaatggaac
gaaagtgggggagaaaaaattttcgacatatctcagaatgaagaagaatggatggatatcatat
ccttagtggaaagtgtatatgaacctgtattttctaaatcacttaaacctgataaattggcaga
taaaacatgtctaaccgcggctgcctttgcagcactagcttctgccgtggatgaaaaattgaca
atcttatcaggtagtgatgggagtgtgcttcaacgtacaacaaaggttatgaaaaaggacccca
aaaaaatagcagaatctcttttaaataatgaaaaatggacatctattttattggacaggttaaa
aacagccaagaaacttctaagcagacgaggtgcactgaaaagcgccgaaagagtagaagtactt

catcggttgaataaactcaaggaggctcctcttccccaccatccgagcctatttgataattta
gtggaggaaaaacatcagcagtatctgctggaacagtcatcgcatcagatatgcatttcaaatt
ggttgaacatattttaaggtctcctttagaaaatggggtccctgtggagataaaactgaaagc
ggggaagaagaagatgaggaagaagaagaagaagaaaagaaacattccatatcaagattcgtgc
ttcaatttatgaacggacacaacgggcaacattatcataggcccgaaagtgcttctgtttactt
ttgtgattattatgactatttggcctacaggaatctccctaatgagtacaaattatcgtcaatg
catcctggcacattcaatatggaggatttacctttccgccctttcgcagtaccttcaacttata
agacagaattagagtacaaaaggtttgtgcaatcaacaaatcttccccagctaagtttcgacta
tggggagtttttatgttactgtatcttcggagcagattggtacaaacacctgggggatgtggta
gattctctagaaaatagttccatgatatcatttgattctcagacattgagtggtgtgtataaga
acactgctaattacaaaaggttggggaagaaaagaaacggaatagccgatttggccgttaggag
tatggcagaatttatccgcactgaagcgcataaggcattgacagcagaagagatggaagaagaa
gaagaggaagaagaagcggaagaagaagcgatggaccaggagcctgcagaagtagacttccttt
cagtgcctcatttacgccgtaaaattcgtcaagctgtttctgtgttaaataactttgtggagaa
cgatctttctatattggtttctaacttcaagaatgtgttaaccgatgatactgtatcaggaaca
gatacggacaactttggttctagtggagaatttgaagcattatcttcccatttattcctttcaa
gaatattggatgaagtgcacattcttaggaatactgatatacaaagaaccctattttcaacgca
cgtgtctctgtcggataaatctcccccctagccgtgtccgtggaagcaatgtcaactttaataat
aacgctgggaacatttcttccctgcaaacgtatggcggtatagaagagttgcctgaaaatgtac
tagtcggtttgtccggaggatttgaagataccgacatgtattccggagaggatgttgttgtcgt
atgggatggttgtgatggaggaaaagtgctaagtgtcaccttcaattgtggtgataattttatc
cagctccatgaaaaaacagcagaaacttttaaggatgatacggatttagttgaacgaataagag
atgtgcttcagactgcaagtaagaccggaaaccttaacaaaaaagcatattcaaggaagaacat
ctacgctgttttgcgtgaaaatggcattgagcgccccggggacgattttacagaaaaggggatt
gctctcaaggataaaacaaatcaacccccctcccccctgcaagaagtgccaagataacggttgaag
gagtcaagggatttttcagcggttttcgtgacattttggagacgagggcgctcaccacatatag
tgcagaaaccttcagagatttaggccaaggcatagtaaaagagaccgaaggactgacagctgca
acagtggcagaaacatccttctctgaaggtttagctgaaagtttaaggtctgatgcgaatctag
gtctagaattttcagaggacgccaaaacggttgtattcaaaaatgacacctctcgttctttatt
ggaagaaactagggcattaagagcaaacaatacttctttttcgtcgtttgcaagggacatgggc
gtccaagttagtgccgatttagatgctgaatttgctgcagagatgagagaaacataccccgatg
cagcccttgaacaaaacttgaaagatctcgacaaattcgaagagactataccagaaagtcaagt
gaagaaactaaagaaaatagacagttatttgacagagaatccagaaagggctggcaaagaaatt
aacgacactgaactgtcaaaggctacagattcagtattggggaagaaactaggcaatgcagtta
cagtgttgatgaacaactttggaaaggttacaattgtagtaggggcttctgtggtggccgggtt
tttaggtccagccgctgtcgccctggtgcatgcgtccagaggggcacatctcaacgtcgtggac
cacaccagccctaaaggtgtcatcagttataaaattgtggacttttcttgtgcagatagaaaca
ccggatgggctaagccaaccaagcacccgttcagggaagaaatagaccatgttatcgcactaga
tgcatcattcttaactgaaaatggagcatatgtattccctgaagacggaggacccaaatcgaaa
tataaggcctacgcaccaatctgtggaacaaaagatgctgctcaaggagaatgtggatcttggg
caacattcgacgacccgcattctgtattgccttgggtggcaagcatgaaagatttgcctaaagg
acaatccctctcctgcgataaaggatgtccactttaaaggcagtttcttccgttcttttgtcc
ataggaaaggatgttgcagaggctatttttgaggttgcagaggacgccgtggtggggttggcga
gcaaggcaatttcagctgtaataaataaccccttgttcatatttggagtgcctcttggatttgg

tatagctgctacacgcctcaatccatccaactggaaaactggcctcattgtattttcaatacta
ctagtggtcatactgatagttcgcttctttgcagggtcgggcccgctaaccctgaattggtttg
gtgcaaagaattcagctaaaggaaacagactgaacaattcgaagacgggggaggaaatcgttc
aaaaatagtattggcagaaaggacaacgccaatagtaaacttcaatcgaggaggaatgaaact
gggcccatgagattagaggagcttcctgggcatgaagatttgcgcccagttttcttccctgcca
caacaaattattccaaatctgccaagattctgggctacaaatctaaacccttcaacgactttta
tacaaaaataataaacacagacatcataaaaatggataggtaaaataacacattaaatgtatat
tttatatattttattttagtaacaataaagattatacatgtataaaaattgcattgttttatt
cctatacaccaacaatagatatagacttatcactaagattataagtttctgatgacgttttcc
atcacccattctgattttattcctattcttctccaaaacagattgcattccgtgtccatcaaca
tccgtgttcttcttgaagatgtgggttatatatggctccgacatatcttgtactgtcgattgga
tagtttcagctagggaatgtttttctggtccaaattctcctctcccataacggatgcgacgata
agcttcagcagaagccagcaggcaaaagtgggcccagccagcctcgtcagtacacatggggtgc
ttcagagttttgttctattgtgcacatcaacatatgccataaaatcggaatgggtcaagtgca
tgtcctttaatttacccgtttttgacaagtaagtgactggcaactgtttggcagtaaattgttc
attgatatgagaagtggagtatttttcttgtttagaagcatgtcgtatatagagagcgacagct
aaggcagttattgctagaataaagaaaacaccaacaaccataggccaattcattcgtttagtag
ttattacaattttgttccacatggtcataatttctcagaaaagttcctcaacaggggaattata
gtacgtgatagaattgcagtgataggcacaggcggatattccgacggtcctcttccgagaatat
tttgtctagattgaagcattgcagcaatctcctgtttagcatggacaacctgtatatcatcacg
aggcaaaagattgtcctttgatccttttgataaagcttcgatgtttataaaggaacctaaaagt
ttccacattatagcaatgtgataaaagctgtccaacgaagcatatacagcagctgggaaagttg
tccatattaattgcccactttgtaacgcagaggctaaattttcaaccccgatcttgttgctttg
gcagatgttcgccatccaatctagttttgttatcgtatctagaactggggtattcttagccagt
ttgactgcctcccctttcggttctttggggaagtaaactggctcctttttcagtagagttggttt
ttatgtttttctctgatgcagattctccaaaatataacagttggcgaataatggagtcaacttc
aatagtatcactcttcactaattctatgtccctttcgattagttggcacacgtctttatctagc
ctcatcctgttagagtttcctgtttcggctgctggtgctgtcgcagacgcagttgctattagtc
catcagatgcgtccagtatggaattgggaggaggagctgcaacagataacatggcagtaatatc
atggatgatttggagtagtccagttctaatacatcaaaatcctgttcttccccttcctccatt
ttgttggtgtccatgtttgaggagggatggttgttgatactacccgagctcatgccgtacaccg
aatgacgagaggaggtaggagcaggcccttttttatagccctaaagggccattatcgacagtact
attatagaagttatttttatccctgatattatacaagcagagaaaaatttcagtctacttgcag
gagacatcttttaataaatttaggagtcttctccaagttttcttcttattggttttctgttt
cttgtctgttggtttttgagtcttttgttgtttagcttatgtatttggggttgccgattacgg
tgcctcttcttcctttattcctgtcagcaagtaaaatatccttttcgtacttgataaatctat
caacgtactttttattagcctgtttctggtcgtggaattccgtgaagaagatcgtagaaagtt
tgcatcagcagatgtggattctgtatctgaatcttcttcctcctcctctccaagaagagaatcg
acgttagtgtctgattcagagtcatattcttcttcttcttcttcctcatcgcttgaaggga
tggccgaagacgtgggagaagaagaggaggatgaggaagaagatgtagaagtagaagccatttc
tttgtttgttgttgttacctgcccatgttcctcctcgacatcatcttcttcttcaatcttattc
agtgcacggttcaattgctgctgataatgccttgtgtttgttacagtgctagaccataaagccc
tcctggaagcagccgatgctccagggtccatttctgatcgcgtacatctaaccttagcaataac
cagaaatacgtgctttttttttactttcagccagccgtgttagtcatctacggaatggtgtctt

tgtctttagatgaggagtcgtacaggcaaaatttgggtaaaccttcaaactcgcctgtgcagtt
atcaaagcatatttgtatcaaagaaccttccgcacctattaaatttccaaccataaaagagtct
atgtgttcattaacaaaaacactatttctttagtgagtgttagtttctttctattgcacagta
ttatgtccacatcgtgcccgattgtgttatttttatcattgatgacagaaatgtcgtaagcact
ttctccttcttcttcccccactccctttctgcttgttcctcctcctcgtcttcaaattcttcg
tcgtcgtcttcttcttcttcctccccgccaaacctctccctcgtttcttcctcttcgtcattta
ttatatgtctcctaaaattcttgtccaataattttgtaatgaaaacacactctggcttcttcct
gacctttaagaaacttgcatccatcatgctgttcattctcatatcacaaaggccatcatacta
tcatctctggccatgatcgctttgacgctcaagaatatatcgtacataatcttagactcttttt
taggtgagaaaagttggttttggaactgtctaaaattaatgaaaccacgggacaatttcttagc
caaacccagaagatgagaacctccgtacccttctctgtgggaatgtgctagtgcagcattatgt
atagtaaaaagcaaaccaggatttctttagccaaactctgtataacattagttgccgtcacta
caggtatgagatttgtcatatctaattcaggtacatctaaacattcactgataggaatcactcc
ctctttctctccttgaccaacaattctcaccgtcactagttgatgtgcatcttttatggctcta
tcataatcatgggggaagtttgatagagctaacgttgtagcattttcttcttgcaaaggtaaac
acccttcgtcttcatcattgtcgggaacatcttcatcaaagtccatgccgttattgttattata
tgccattatattagagaaaacatccatctttctattcctaggaccagaagggagcattagagca
gttaatgaaggtgcatcactttctattttatttgtcttggaagtaacttcgttatttttcgtat
tcaaagtgtctatcagggcagatgctgcatcacgcactcgcttctcgcgtgggagaagattgtc
acaatcagatttaaagtggactaaatgcagacacaaattagaaaaaacgaattgagatgcggat
ttaacaatggacaatatttgattatgaggaagtacagacgtgaaagaaagagcatgcccaagat
cgtctgatgatgtgattttattttgggcacctataactactagcgctatgtgcatcgcgaggac
aattacttcagtgaagataccattttctaacgtcaaaccttctacgaatgcaacccctcctcta
tttgtcctcatcatcatctttgaaaaatgttccatcaagtgtacctttgtcatactggctgtga
caatctgtacgatattccccatagtctttaggactaacccttgatcgataaattgttccctcat
aactgcggcagcaatatctatggaattattactgtatctatcggcatatgttcctgcctttgaa
tctttgatcctcttatccaaactatcaaaaaatcttctctctgcgggcgagtctgtagagaata
tgaggaactgagggactgaaagttgaccgtttctgtgagtcctaccgatagtctgtaggaaagc
aatggcgttataaggtacatctaaaacgcaatggtaacgtttagcgtacatggagttattagaa
gagtcatgtaaagagagaccggtattgcctttaggtcccagcattataacgtcgacttctttcg
tgttgttgaatgcactaatacatttgtttgtgttggcagttttagtgttgtttttaaccagaaa
caatcccctattcgttatacgcgaacacaatttcctgtttgttatttcagcgttactatcctcc
cctattgattgtgtgattgaatctatagggttgcctgctagcgccatgagcggttccgtgtcag
ggactgaaggcacaagcataacgtgagctggggaagttcttctattggttttattctttaagga
gattgtgaaagctgaagccatcatgactgcacggcacaaaagtctatagcctgagaatattgta
ttagcaacaggcgatgaatcgaatgtccccacgtctacgatgccgtattttgcgtcactttcat
ccttggcgatcgtttgtaagatgcgtgtagtcaaattcttgagaaaagaatccccagtttgctc
tagtgacatgaccatctttttttgattcgtttgttgtccttaaacgcttcactgcatcggtgaca
gaagttgccttgatattcaatagtgcacctttacatgctgccacagaagtacttgctgtattaa
tgaagtattgtctcctcaacttttcaaaacactcgtcgtcaaatacttcaccccatccgtcttc
ttcattagcaccgtcgtcataatcttcgttaaaatcagttttacctttggaaggtttttccttt
ttcttgatagcatcgtataaatcttgagcaaccatatcaatgctgcttatgtcaacttccggag
acgttattattcctcctcctccattagaaagcgcatcctttttctgtgagtaggttttatagc
atcttgtactatacttcttagtgttgttttcccatcatgtgcgacatccctatcgcgcacaacc

accaccttaaaacgcctgtcgatattttccattacttcatataaatcatcatcatttgcagcac
gtttcctctttctggacggggaattaatctctgctactaatttttcaacatcttcctcttccaa
tattcctccttcttggtgttctcgtataatctttgtgaaggcccttctgacatgtcctcctact
ttttcagaatcgatgagtacttgtctagcgtttaaacagtacgaagccaattcgtctatagcgt
atttttgaagaggggaggctttacaattagtagtagagcagtcgactccagccatgctaatact
cctactaaccaattgtcctctatttcttaaaagtttaataacctgttccagaaacactgtcacg
tactcgctgtgctcagccatagcatctggagtagcactggacacttctttgaaggcttgtatgc
tcgtgtaggctggcgcacttttccttagtatgtggtcaatcatgtgcaaatcagcattgctctg
gaatggcgtagcgctggccataaccgtgaacgtatcatactttaagatctctaagaaaatacgg
aacgcatcagcttgagataatagtttcaagtagttggacattgtaactcggcccttattattgg
atgatttcatgcgcttcactttaaagtttctgtcgtcactcattgcactcttgaacctattaat
aaggtctgacactgaagggtttgcccttttttgtatgttgcgcaaaactctagtatcttcttcc
tcccaagtctttgccagttcctttctaaaagagtcactaatattttgagtcttgtggaactcgt
cacataataatgctgtgacaaagttgtcaatgggcataaccttgttggaatcgaggtctgttcc
tcctgttataaatttcagtacaaattccagattcgtgcgtaaatcagagtacgtcataaataaa
atggtgggcgtttctgttaagaaatctcctattgatcttgaagaacgcttactatgaaattcct
taacatccctgagagtgaaaaatctaatatccaccgagtctttagcagacgatacagaattttc
cacatctactgtcatggacccacttttcccgtcgctcttgaaagacgttggtttattagcacaa
ggcacttgtagttttctccatgaaaatttaggatcacgaagacctctagaatttgtaaccactt
ctcgcatgccctgttgacagctgttgaaaagaggcttagagcacgtcaaccatataaaaaaggg
gtgccttttccacacgccactgttgatggccgtaattaccttgtccgaatcttgaccaaaaatt
gatggccctacgtgtttttgtacatctaagagtgccttttcttgtagtatgacactcatgacga
aggctgccaattctcttgtttttcctacaccagtaccatctccgatgataaatcctccactcgt
tttcttgtccactttaaaattcctttcgaccttatctttcacaaggtctataatggacatgttc
ttcttccaccctctccctttgtagaattgaatcagggctagacgcactgctagtgcctgcacag
aattaatgtccatcggccgaggaagaaatttggggtaggcatcggttacattttgcagaagcc
caccgaccggaatgtttcactctcataaagagcgaatgaaggttctgggtgggagagggtgcca
atctctttagcatcttcttcctccagaaataaaagtttgtgagccatggtaattcaaccagaaa
atcaaccaatatatgttgtaacaagaagttgccttaagaagactcttgtgtactttttttacga
tacttgaaacgagtacgcctgcacggatcaaaaatttggtatactggttagctgctatttttgg
caaaataatagtttttattttcccatgctaaagagaaccaggagttacgtacagccatactagt
ctctacaagtaatcgtctataagcctcagaagtatccacaccgtccctgaaccacgaagtttct
tgcacgtccgccttcttatgcccccactcgacgagtgacttcttgaatagagagaggggtgtta
ttgtagctccttcggggggttgaggaaatgtttgacgcatcaaacgctagacgagggtctgactg
tactaccgggccactcttggggttatgatccaaacgaaacgattgcaaaccccacgcatcgtcg
tcaatatccactctgtactcggatgaggaagatgatgttccgaacaatttttcacttccgggag
ttacattggagcaatcaaaatagctggggattgtttcgctgtcaaatatcctattgtggtctac
tgaaaaaatatcgtcggtagctccttcttcgggaagaacagagtaggatgtggggaaagcgtac
tgcaaaggattggacactttcaatgtactggaagtttttgatgcaggttttccttttaggtaat
ctaaatcaaacatatctcgcgctagcacagtagcagcataaggtccgtatgaacgcattaataa
cccctctttcaatacaaaagtatcctcagattttttatgatatgtcttagaaattctttcctc
ttcattaaactatcccaagaacttgatttgagatatatttcacttgtcctcatccctctcgcgt
gtttctttattacgtattctgtctgagacaatctcctctcatcgttaaaattacgttcaaaaaa
gtcccttatttcttccagcctttggtcacctgtagaattgtactctggagctggagtacctcca

gggggagttctgatataagaggagggtttattttgactcaaaagtgtagagaataaaagttcag
atccgccgtgaacactacccgaaaccatgcgactatttcgcaaacattctgtggccaacatact
atgttcatagggtttttgaggtatgtttccggccaaaacattagctgctggctcaatgtaaaag
ggtgcagtacagaatggcctatgctgccccgaggaaggtttgttcaggacgggagctaaacaac
ttctatgtaacaatgaagacgggcggatgattacatggggaatattttctagaggaggtaaaac
agtgctaggtattggtacaaagagcacattcttagaagtatttgttgcatccaggtttaaaggc
gtgatgatgggcgcttgggccagtgcaattttcttggcagtagtttcgtcgatacttgatttag
gtctttgctggtgaatctggtcccctcttttagattggtcggccaagaaaattcccatggcagg
aacagtccccactaacggtgtcctgtcatcgcctattattgcatgcctgtttgagtcttgggga
ttattaacgcccggtacacacttcattgtgctcacatcaaacgtaacaaacgaagggtctatgt
tgggcatcataacacaggggttttccatacattcatacatacctctgaatttcatatcctttgt
tacatcttgagaaagaaggtcgttatttagtacatccttcttattattacagaaacaggtgatg
gggtttttgtataaaaattcaggatcgttgcatttaattttgaattccgccaaggaagaagatt
gacgcaatttggagatgtaagaagagttgcggaattctgtatgattccgtatttgttgtttctt
taatacatcataatagattggtgtcgaagatgaaggaggtggtggtcctttgtgggttgaagga
ttgaaagcgcaagcagtcatcgatgttcctcccgaacctccgaaatatgttgggtatttaggtc
tgcattgccagcccccatcatctgtcagaacgactatagatgtgtgcacagaacactctttaac
tacacgttctccagtggttggggatgtttctatgtggtcaccgggcacgtttcctgaccaacaa
taaggtttaggatcttctccagatttatgtgtgataaatgttcctccagcaacaaatggtgtca
tcacacgaactgctctcgaggtataacacacagatgtgcaagtttgtccctctttcaaaggaac
tgggttggaacagtacccgccagtattgtaggccaaaatatccacatcttcatcatgtccttct
gatgtagatggaggaggaggaggagcgtcaccacttacgcctgatgcactatggtgatggatat
catgtacaccaaataaagaatcgtacaattcagtatcgtctttttctatattatccttcaaatt
gaccatcctttgtctaaacttttccccgatacagcatctagatatttgttagattttcgatg
gtggcctcactcaatctatctatatggtgttcaggcacgagaagagatgaggatctatttgcag
ctaaatcatgactttcgaaccgagaattcctttccttcgtttctgacggaacgttccatttatt
ccctttaacagtcaaattcatcatgtccaggagagcggggtgaggtgggtaattgcttgcatct
tccaccccgtcatgcctacctctggcttctaggtattcttctagtgtcttttcagggttagctc
cttgaactgctttcaatttacttttcatccctaattcgtgtatagcttgaaaattaacagacat
tggtttggctacattataggccattgcagcgcagactagcgcaaaaatacagcctaacacgaac
acggtcacgaataacgttatttgcgcggtgaagcgcatacttcggttggtagggtaaagtaacg
gatctcttcgtcgttcatccatcgttcctgatattacaatggatatcttcctagataaatctca
tacaggtgccacccttttagggtcttataagacgcagactgtgaaaaataattctacgttaaaa
aagcttaaaatgggcgcgcctaccaacgctgattttacacgcacggtatcaggagtagcttctt
ctctctatcttgttaaccctggagctccttccgatagagaaaagttagttttggcctcttctta
ttctgactcttttgtgtacaactacaaggatgcagtggtgaccgctgaggctcccaagtggtgt
ccctttaacgagccagctcttcatgagcacatcatgaacagacttgaaaaagctggtctaatta
acagatctcgttttgtgtgtaaccctgttaaatcggctggagaagtatgcggatttcgctattc
tggaggaagtactccccagaacttaattttcccgattggagcatcagagataaattggttggtc
atacttagaaatgctgctcgttttggtacagtggcagcatcggccaaagacgccatcgaacgca
ttcctgatctaagagaaggtggtacaagtaaacatgtggcaaagaatgcaatgaggagacttcg
tgtatggcgagcctttaactggatagcggaagcctccagatcagcgggcatgattcgctacgaa
ccctttcggttggctgtgctctatacgatcacgaggtaagaagaaggcaactaaaaggaagtt
atgagcgaaatgtactatttcttggtgaccatttctgtaaagaatcaactcttctggctgatat

ttcaagagggggaagaagttctgatttctggacgatcgttgaagctgtcatccggtacaagaat
agacatgctcgaacaatcagtaatgaaactaatgccatccctgaagactcttctataaacttgg
agtgggaggatgttctagtaaaaaaccaacgcgacacaaatcaaggagatgattctacgttaga
aaagaccttagaagctgccatcaaagaacatgaaagcataggagaaaaaaggaaaaaacatatc
ctagagtttattaaaacatgtctaacagaggagcaacgagaaatgattttaagggagttggag
gaaaagggaatttgtccccggcccatctaacaaatctggccgatgcgatactggctaataatgc
caaagcaggcatatgggttattatgcagagtcttttaaagcaaatcaatttctctatactccat
ttaatagggtacgaagctcaacgacttttaatgttcaagttgtatatgcctgcattacttgctc
tttttatatctcagaggggaattggtgacgtgtttctgaatggtgtgtttaacctagaagtgat
gaaagaaagagctgcaaatgcaaaaataagagacatggtttctcgagacgcctacaagaacaat
actaacgagtctaataatttgggcaattattcccaattcgatatcgctacaatttatggtcaaa
tgtctgattgtaatgcaattcctctatctattaatattggagtacctctccataaatcgaggat
gaacatgcaagacattgaaaagaccatacaacacatagtggatatgggtttggcaaagatacgt
gtagcggaagaaaatagagtagctagacgtcttttacatagaagaaaaatgcaagaaaaggcag
ccagggagcgagcagctgcgagacagagaagactaagaggtgaagaagaagaagaagaagagga
ggaggaggaagagggagaagaaatggaagaagaggaagaagaagctggaacatcaggtgttaat
ggttctggttatgatcaagaagaagaagatgatggtgaggaggaggaggaagaggaagaag
aggatgaagaagatagtgagggtgaagatatgaatggggaaaataactcaagaaaacgtaaaaa
cactggtaatacttcctccactcaacaacctccccaaaagcgtcagcgaggtaaaaatgcgccc
atttcaaccaagggaaaaaggttaaaggaaagagataatattggaggatttttattagccacaa
ttcaaaacgatgaccgccaagtgaatgtggagagcatacaaaaacttttgacggccaggcaaag
aaaatatgttaaaggtggaaaatgtggagataatggactaccagaattgttaattgaaaaggtt
acaaatctactagattcagtgttccatttcagaaaggggtctattcttaacagcatacacgcaa
ataggcgatcagaaactggcgtatacaccacaaaggcaaactgtatttgtgattactatgaaag
aaacgtatcaaaagacagtagtaataatacaccccattcatctgaatgtattgagcgggcaaga
gaaaggatgcttcctgtgccgaatctaacaaacgcccttgtcctgtagactctaacaaccctg
aagatgtggaacaacgtatgcgggaattgatcatggaccctccttcattatcaggggtagaaga
ttctctcgctatcgaaagagtacttcaaaatgagatattattcacaagtcttgtcaccaatcca
attttcaatgccgtgttaggtgctgaaaaaggagatcttggaagatttatcgtattaaataaca
ttgtaaaatttatgaatatgactattgcttgtctagtggacggagatatgcctatgcttctaga
ctcaagaggcaagacaaaaaaccttctagaaaagggtacagtgaaaaacacgagaaaattttc
aaaccaaatatgactgcagcagaattgaacgtggccactgctcaatctgcaggacaccaataca
tgaacgctggtcattgtccagaacctggaatcaagcaaatgttactccctgattgtataatgaa
actgaaatccatcgccatggaaaagggtcgaggagggagatcggcccttcacagacagaaatgt
gaccatgccttttgcaagatgctgaagtgtttattcttcaatattgacccttcaaatgctgcag
atacatttattgaccctgcgtcacgtgccaccttatttaggcttgatgatctttgtaggatag
gaaaaaatacaagaacattgactgggtaaaggatttactagaccctgtaatgaaggaacaaat
aaatgggtgggaactggagaatacactaacattggacgtgactcaaacgtggccgcccctgttg
atttttacacaattttgaaatacacaatgattgatgatggtgtaataagtgttccttcacggaa
accaaatgacgtgtattacagtactattgaaagagcggatgacttactcaccgagagcagggac
gcttcatgtgaaagctaccgcccgaccttgtttgacgccagagcggtgttggaagtcaacggag
acggacgtgtcccttacccttccagtgaaaccgtagaagacttgggaggagaagaagaagaaga
ggaaataacaggcgttattgacgacagtactgaaatagaagacgttcaagtacaggacagtaat
ctattcgatgtggaattattcgatatccctgaaatagaacaacatcaacagggtggagaagaag

aggaacttccttcagcaatatctgaagtgtttgcttcattaccagctgataacgattcctcctc
acccgcgcatattccctcatttgggaattctgaggaggggaaaaaagtccagaaccgtacaat
atttttgattctgccctcgaccaattattggatttaatagatagcgatggaagaaacaataacc
ctaaaagagtcgactggaacagtgtcaccattcaagaatgaaaatattaccagaatagcgtcta
attacgtccgagcctttactgacacgtggtctcatttggtcaacatttctggagctcctctaac
tgctgagaagaatccaagcgctatcccagctaacgaactgaatagatactggaccaaaactaac
gtgttatgcaacccactctttaaattggaggaccacataacgagggatgaagatactggtacaa
taacactaaaattcaaaatgtatatagatgataaaaatggactatatcagtctgccgttttaat
gctggctctcgattcgttcgtttcgcttgcatctttttcccatggagctgatttagtttccaat
aaaagtgaaaacaaattctgcgtaaaaattccccacgacactcgcgctgaatctttactgaata
atgttggattccccgctggattaagtgggcctttaagagatggagcattaactacaaggctgc
aaacttgagcggtaaaagcggtatagatggcctttcaggttccatgttgacagtactaaaaaat
aacaccaataaaagagcaactgatattttacatttggtgaataatgtttctgcctctgcacaac
aacttgacgattctgaaatgtctcgtacttttaaccaccaaaagaaagttggagtttgttatga
tataaatgtgtccagttcaaggcaagtgaaccagcgtaatttactccaccatcagaatataata
ggacaacatctgatcgaatttagaaccaagcaacttgaacgcgcacaaaataaaaaagtcaagg
aagaggaaaatggtgagcatgaagaaatgacaagtgaggaggaagaagaggaagatgaatatga
agaaggaggttgcctatcagatattgacgaggaagatttctatgaggatggttacgatgaagaa
gagggcgatgacaatagaactagaaagaagaagaaaatggaagaagatgaagaggatgaagaag
aagaatatgacgatgaagaagatgaagaagaggcagaaacttgtggtgctaatggtgttattga
ttgtgaagacgatgcaatcattttccccaatggacaaaattcaaaaaggaagaaaaatggtaaa
aaaacaaacattaaaaagcggtcacggaggaaggggagtgctctgctaacactttatcctttg
tggaaaaatacgttggaaattgtaagagcctaggtataaagccagtagggtgtccgccccccatc
cactgaatttacttccctatttatgaaggggagtgaagctgacagctgttataatacttgtcag
tccaccagaggggctagccgtataaggtcactactcaataaatactctgttaaagatttgatgc
aggtaaacagcccttcgagttggaaatgggctaaccctcccgaccgacggttcgtgctgtttga
taagaaaactaaggaagaagttgaggttaagtttgaaattgaatgtgaaaaatccgagtatttt
gatgtcgtatctgaactccctagtaatattaaagtatggttaaaagagacggcaaaaataataa
aacatttggctctgattgaagactttcttccagctatgggtgctgctaccccaaaaattcccct
caatttgattaaaactatgacgagcattttctctgttagagatattgttggatttaaaatacca
gaagaagtgctcagttttattcctatagaatggaagacatctatttctgcaatggggctcctct
ctgtacaatttgatcgtataatagaagtgatagatttaatgataactaatggcgcctttgcgac
gtcatgcttgaacaacgcattcttcttggaaagaggagtggtgcccagagatgggagtaacacg
tggctccacacggaccttgtgcaactctccacctccatatttagaagtattcgcaacagaggag
tgaatattggcggtaacaacaacactggtagcaattcttctagttcttcttgtggagggaataa
gggcgattatggagtacgttgtggattgagtataagcaagcgtggtataaccctgaaaccacca
cctgcagcgatgactaattcttcttccccgtcatcctcggccatgatctcattgcctcagccca
cgcgccagagcatagatctttcgataacgacaatcatccaagatttctcagaagtttctgggaa
attgaggcttaatggattacagaaaaacatgtctgacaagagcaaagacgtgtttaatgatgca
atatacgactctggcgcattcaaggcgctcctaacatgcacagtcaacgataaaagtagacgta
aaaggaaaagaaggactttattggcatctggagagggtgtggtacgaagaaacctgatggtgag
tcagggcaatgacgtcaatgatgcccaccagttccaggaagaatgcggaataaaaattgggggc
ggggcttctagggtgtataaaagagcccagcgccgaggttcggcagtcagttccagaagaagag
taaggaacaaaccccagtttactatagcagtctctgacgaagacgacgactgcgaagaagaagg

cgacttttcttccgagttaaatccaacgcactctcaactacttctattccaacaacggcaacaa
gatagctgcacagaagacgacgacgttttagtgtctgtagaagaatataacaacagagtaagcg
gttcttccaccacagccggagacagagttcttgcaaaggatcttctctctactgtatctccgaa
cgaaaagaggaactctgccgccctcgccgcactcaccatatcccggcactctcttttcaacgct
ctatctgcaaaaacaaagttgggagaaaatggacgtttcttcctataagagcactattgactac
cacaacattgaagatatggacgatctccagcgcgccacctacaaggatcgtatggagacggaat
tggtcctcgagatggctaagaaggagggaaggtacgtccgatcgttggccaccatggacgaatt
ggaggtacctgaagaaccagccacttgctacacttgcggctacacctttattagacgcagggca
cccccaccaaaacgcaagtcaatattcagagagccttgcgcttacccagaacttctccccgatg
caccatcccccgtccgtttagaagagcttgtcgacgtgccagaaggagcgagtttttcacctа
ccctccctacgacgacggatcttctacatcgtcttcacaagccgaatgtgaagatgattatcct
ccaccatacgacccatcagaaaatccacagaggtcccaagtgtgtgattattgtaccacacgtc
aagtcctcagttctatgacggatcacgccagggccaacctcataaaaaatctgaagagggagaa
gaaggccctgggtcttggccgtcgcaacaactttagctactagggttgtacaagaagaaaatag
gattgattgtatcgatgacgaactttgcatgtttttgaccattcttttgcttgctgagtgtaca
tagctttatatttgccaataaaacagaccccgaatgtattttgtgtattctttattactgcct
atttttatggtttaacggaaaaaatttttgaaaagtttttgagatggagatgaagggaaaaaga
gggcgctagttcatatactgcccaataggtggtcgggtccagaaacgtctgtctgttccagaaa
tgtgtttagagatttctggacaagtcatttctggaaagggttgtaatttattatagttggtata
ttatttctggtatatatttctagcccctctctgtaatctgacgttgggtcgacccagcggtcca
cccttcgaacttgacataaggcctagtcagcggtccacccccctaaactggagtgagctgaaaaa
tttttgaaaagtttttgagatggagatgaagggtaaaaggctagtaatagaaggttgtgtaact
cggcataacccagaaacatatatcaaaagggatggaaacaactattctttttatacagtagttt
tattgtaccaaaaactcagaaatctcatcacatgacaaatctgaatgtcccattccgtggccct
caataacgtacgacactggacctgggaactcccacctacatgttttacatacagaaaattttgg
cttagatgcattatcaacaaacactttaccacattggcattcattctcttcagtttgtcacac
atccacaaagaggccatattctccatagaatcagatccatcaacaaatatatagaatgttacac
ccttgtcgatccagtccatggcgacaatagtaaagtcttgtccaaaaacactgtttccgaagat
ttctttgtccccatccatgtattgggaaaaagtacagtatggtccacagtccttgatctcctcc
aaccagtcgttcaaaaaatccctaataaagaaagacaggcaattcgacttgatctggaaaccaa
tgttgagtttcttcgtattttcttcattgttggaggggaagaaatacgccaattgtgtatctgc
ttcaaatgctccacacttccctttatggatcaggcggttctccataatctccttgccagaattg
gggagtaggccttcgaagaacaggatattggggcgggtaggaactttgccagtcaagtccatgt
tggcaaagagatcagcagggtcttcaaagactgaggccatttttttggttgttgctgctgagag
aggagcaagtagtagtgtggttactagcaaggagctcacagtcttatatatagactaacacccc
ccccccaaaaattctcccacatctttcataccggggtcggctagaacccagaaacggtttgact
cacatctggtacattatttctgttatacatttctagccccttcgtgtaatctgacattgggtcg
acccagcggtccacccttcgaacttgacattcgatcgactcagcggtccatcccctaaactgga
gtgacccagaaaaatttttgaaaagtttttgagatgaagatgaagggggaaaaggttagtgaa
tacaggcagttctgccttgctccagaaatgattctaaagatttctgtgcattatttctggtata
catttctagccccttcgtgtaatctgacattgggccgacccagcggtccaccctcggaacttga
cattcgatcgactcagcggtccacccccctaaactggagtgacccagaaaaatttttgaaaagtt
tttgagatgaagatgaagggggaaaaagagggtgctagggtgcgctccagacatctctagagtg
gctagagcccagaaacatcccaactagtttctgggacatttttttctcccactgacgcagactat

ataagctaaccactaagcatatttgcacacatttcatccaccatcactgccgaatacactctt
gtgttgctccgcatagcctaacaatggccccaaactccttccagaaatttgctccagttatcaa
gactgagaagaaggaagaagaaagggatgaacatgacgaccctttacggcagattgattttaga
gatagaaagacattaatctgcctcactgcaaactgtgtttcgaggaagagaaaagctggatctg
cacatgatcgagtatacaaagtactacgctacgggaacccatacaagtaccgtcgccccaatag
aacacatcgaggattggccctctcaatggatcaaggtgaagtaggaacatgcctccctctgcga
cccatggaagagactgaagaaaccccatcgacaagtgcggagtggcgttcctgtactccaact
acaatgaaggcgatggcatgacccacctttacaacgacgaagagtatataaagaagtgcaaaac
aattgaaggaggaacaagaacgtgggtaaagaagaaccgccaagaatacttcagacaagctcta
gagacattgatgatgtcccattctataaaacaatattccaattttatttttttcaaggaggata
tggaggaaggatttgtgcacaaactccacacatttattaatatggtacaccctaaaaaggtgtc
tgttttgtgatataataaagattgatgtttttataaatgaaacgtattatttataccaaaaaat
ttattatgatgtatttcattgggatggcttttcactcaccaatctggcctatgtgtgtgttcat
gctagcccctctttcacccttcatctccatctcaaaaattttagaaatttttctgggtcgct
cgagtttagagggtggaccgctgggtcggcctaatgtcaagttccgagggtggaccgctgggtc
gacccaatgtcagattacagagagggtctagaaatatgtaccagaaatagtgtaccagttataa
gaaaatgtgaccctttccagaaatggcttgtccagaaatctttgaacccatttctggaacagac
gtttctggagcatggtcgacccagatatctgttggcggctgtcaatagtagcctatgtattcaa
tggtcttttaccctcttctccatctcaaaaattttagaaatttttctgggtcgctcgagttt
agagggtggaccgctggctcggcctaatgtcaagttccgagggtggaccgctgggtcgacccaa
tgtcagattacagagagggtctagaaatatgtaccagaaatagtgtaccagttataagaaaatg
tgaccctttccagaaatggcttgtccagaaatctttgaacccatttctggaacagacgtttctg
gagcatggtcggcccaggcagcctcacagttacctgttcacacccttcctactagcaccctctt
tcacccttcatctccatctcaaaaattttaaaaaatttttctggctcactcgagtttagaggg
tggaccgctggctcggcctaatgtcaagttcgaagggtggaccgctgggtagggcccatgtcag
attacagagagggtctagaaatgtataccagaaatagtgtccgattattaaaaatttaactttt
caacatgtggcatacaccgtatgaacgtatagccaaatatgtaccacagtcaaaacatttaggt
gagaaagataatacacatgtataaatttcaaatactttatttgcatacataataaaaaatcata
cagtttcggactcatctgtccacacatcatttattaggaactggtatctattataccctattct
tctacgtttacaagatacttgttcatcaggcactgccattcttttacaagacactattggttca
accaacatctctttaacttgatcagcgtcaggtcttagggaagtacgttctctgcatacaggac
accgggtaatttctccacgttgcgtactcaacaccaatctgtcccaacatttttcatcaataca
atgtccacagcgtgtcgtcctgatgatattggtactactgaaatcttctaaacacactggacaa
ttatcgctgtcgaaacgcacattagcattcttctcatgttcttctagagcccttttaatttctt
ccttatccccagaaattatgtgttcagcccaaggataatttgaggatgcaatataccccaagcg
acaattcagtattggccaagaacgaggtgctttattgacatcgagaccggtggcagcaacaagg
ggttcttcttcttggagagagaaatctaaagcagctttcaatatggtgggtatactaggctcgt
atcctctctgggcaaactgtgttaatgcggtatcattgaacatcaataatgctgcttcagttac
acttaaagtactagatttaccatacaacatattaatctttgccccatgggctccaactagtgca
tcagagacatcgtgtttgtgattggcgatggcaatcattagggggcgttgcaccatcgctcgccc
ttctagcattcactatacttctatcgaaatcgtatattatattcttgtatttttttagcaagagc
atcaagaaaattgcacgaggttgcaacagctgcagcatggaatacgttttcattattttcatta
ataaaggaaaaggccaagctgtttgttgttagaatttcaatacctactcttgtggtaaagggggg
aagatattgcagcgcaaaccattagaggtgacatgcctttcgcagaaggcttcagaatgttaag

tttttcttgtttgactagtttgctagtatgggaatatttgttaatgatacaataacacaataga
gtattccccttttcatcaaaataagtgtgtttgagccacggattcttggtaagaaaagcttcaa
gatcttccatgaaacacgagacttttacacacaatctaacatagcctttaacttcatgagggtc
tgttatcacaacgtttttatgtgatacgatccttttcacaataagattatcgcacagtttgtcg
gtctttttcttaccataatctccctcaaacagatcttgaagtaattgtaatcaatctcttcaa
agggcctactgttaatcatccacaacagtattgcaccacacaaaccactacccactgcttcgtt
tattgcaacatttttagaatattgtccatcatttcggcagagaatttggcatcgtgtcaaat
atactgtaaagggtttgaaaatgctggattgtttcagccttctctataaacacatcaacaaact
ctactgccatttcagcaacagttttgtagtagtctcacagtactcttctaccatttcttcttc
tatcgaggaaagaaaagacttgataatcacactcctgtaagcactagccctttcttcgttggtc
caattcaatctagctatagatctagcgtccatgttcatttcttctctgaagtcagttgacacgg
atgatggtgatgtttctaggcaagaaaaaggtctcccgataataaaattgccattggatatcag
tcgttttgcctttgtaacacaaggagattcgtccacaaaatacttgtatccgaaagatatgtca
aaaggttcaagtggtgcagatttttttcatttcagccacgtaatcagaggtgatattgacgattc
ttgaaaagagcctgaatctaataacactcgaacatttttcaacgtagaaaacaataccacttct
tgcagaactagtagacttttcaggctagccaaaacaccgtccaacttcttgatccttctcata
accttctctctttcttcctcagcctgttcctttgaagtaaacttgaatccagttctgctgtcat
caccagtgccaaacttgatgccgtgcgtctcgcgtctcaaaaatccattatccatagagaccag
aagagaatatttacgaacaaaaagtcgtcgtggatgttttcgtaaaggcctctgaaggttttg
cagacggttgtcaatgcgttgataaaagtcattccctcgcagatgggggaagaatcagacttgg
tattgttgttgataaagaagtagataatatctctaaactcttctttattgtctaatttcttgaa
actacttgaaggaacaggaggagaatttctggaggtaattatgtcattcagaagggccaattcc
ctttctgtgaaaacgtccagaaatgacatatatggttcaatgttttcaagtacttcttcaagca
cctgacggtatcgtggagctgcttcagccatgttgatgatgtctcacatacgactgttgagttt
atccatgcgtacgcccgcttttatacaaagatcccgtgtaagaaactccctccggttcagttca
ggataggggtgtgtcccagttttacatccaaagttaatatattttttttaatataacaaaaaaat
cgtaccgcttattggctgctataaaagagggagcacctgctcacttggacatcattaaccatca
tcaatatggagggagaacatcaatatttgaacctagtcagggagatcctagaaagaggagtgaa
gaaggacgatagaactggaacaggaactctatccatttttggaccccaaatgaggttctctctt
cgagacgacactattccagttctcactaccaagaaaattttctggagaggagttgtggaagaac
tcttgtggttcatcaggggcaatacagacgccaaagaattggccaagaagaagatacacatctg
gaacgctaatgggtcgcgggaatttttggacagtagagggttatacgatagagcagagggagat
ttgggacccgtatacggattccaatggcgtcattttggtgctgaatatgatacctgttcttccg
attatactggaaagggtattgatcaattggccaatatactaaagaccctgagagaaaatccaga
tgatagaaggatgattatgacggcatggaatcctatggatcttcaccttatggctcttcctcca
tgccacatgactgctcaattttatgtggctaatggagaattgtcgtgccagttgtatcagcgaa
gcggagatgtcgggttgggcgtgcccttcaatattgcatcatactctcttctgactcatctgat
ggccagtatggtgggtctaaaaccgggagagtttatcctcactcttggtgacgcacacatttat
aatacccacattgaggtgttaaagaagcagttgtgccgcgtccctagaccattccctaagttga
ggattttaatggctccagaaaaaattgaggactttactatcgacatgttttatcttgaggggta
tcaaccacacagtggaaacttgcagatgaaaatggctgtttgaatcatgttaaggaatttcctt
gttactcatttattcctagaaatggtgtaatcgctgttgtgggcggagcatatttgtgtatata
agagcccgtgttagctcctcgattcagtcacaagagcgcacacacacgcttataactagctctc
tctctccactcaagatggcctttaattttgaagactctacaaatctctttgccaatatggactt

gacggctggcacaacaacagaccctacccgccccaatatcatattctttgaaagtctactcccc
aactctggtattgaggtgatgaagaggcgtctcgtacggcaaggaaagtgtgggaattttgaag
caagtggaggtgctatgtcgtatttctggctcgaagataatgcagaagatatggagaatctcaa
cagtggttcccatgtcaagacaaactgcttggcattattccttcaagagtttatcagcaactgg
attgaagagactgatcgacatggacagtactgtacttttccccaatacatggacggtggggatg
gttcacgtgggggatattttacttcgctagccatgaaatggatggctagggatgtgactttctt
tgtgtttgttgataggaataatactgtagaaaatgcggcatccatatggatgtaccaaaaacta
ctagcaattggtgcaaaggtagtaaaggtgattgttgacaatgcatcaaacccaatgttttctg
tatgtaatgcgtgtaggtgcaagtacccaggcccagtgtcatacgttattgaaggccatggagt
gggtcattctgatttgacatgtgatgagatttctggattctttgtataataaaaccccataaga
aacaataatctttttttattcaacacccatgattttagtttttatagtatataaaatcaagaata
aagtatgtagatatctacttttcgatagctccttcacctatatagaggaggtggataaaaacag
aagatatggattctaatacttctattttaccgccaagcaaacggccagggttaaatctgttaca
ggttttagggattataataacggtagcattaatagcttccgtttcatcctttatattttatagg
gtaggtaaacgcaaatattacccttcttcatcctcttcctcagaattatctgatgtagataatg
gggtagaaggaggaggaggaacaacaacgacaccaactcaaccttcacctgacggtggagatgg
atacgtagatctttctcctcaaaagaaggctgaactaagaactagagttgcaaacgtcatcttt
caagaagtgtcaaaggatcaaggagtggcctttagacgggcaatgaatgattcaactgataaga
taatggaagaaactgaggcgagaatcaataacttttcagagccattcagagaagcaaccgtaga
acgtgaagtgtttaaggatgacacagacaaaaactttatcctttcaactctagatttaacagag
gaacaatttaaggacattgttatggctgaagtgaaaaatcaattagaaaattttgactatgaag
acatgacccgtctcatctttgataacatcccagagactgattatttatggacaactcatttcga
tccgaaaaaatatgacacgtactctgaaaaggtattagggttctcagatataaatagtatagaa
agaatatcctctacattttataaaggtaaaaaatatgaggtaactactggaaatgtagctgtcc
tcgttgattttgaatctgaaacaataaaagagaaggcaggaaatagtctcatccgtaatgtcga
gtttattgttgtggacgaacagacctacaaatctttcttccctgcattcaatcaagttttcttc
tcctttaaagtaaataaggagaaaagggaagttactgtatccatcaataatggatgtgtaggta
tagtggccaatattactcctctaactacgccagttggagcagcttccggacactacatctatgg
cactagcacagcaaaggaaaagacctatctatttgtaatagacaagtacgataccactgaattt
gtttgtggtctgagtaacaagtcaactcctctcatggctctaaatattctctttatgagtgata
ctgttttcccttcatttgacgaagcagaaagacctctgacggatgccaaggcagtagaaatttt
aggtaaaagactaggtgtaggaagatacacaaacgccaacatcagaaatactcagtgagatgga
aggggttattttggataagatagaaacaattgcaaaaagggcctctcctagttatgggtctatt
gatgtgggtacggctattttgcgccgtcaattcatggaaaaaattaggggtaaaataaatgaag
aaaccacaatggagaagattatgggcacaaaggaagaaagagaggacactataagaagtatagt
ggctaatgttatcaaagagaatactgttaaagaaaatgtaaccgaaaaaattagagcaatgaca
gataaggaattaaatgacaatagggaatttatgcatgattttggaaaaatttcaactggagatg
gaggaaccttccatctctttgaagatacaccgggttttgaaagtgctttaaaggcagaatataa
aaacgttccaggagcaactactccaaaatacgtatctatgaacagtttacgtatcgatgcgatt
aatggaaaaatcgaagaggtttataatccttcacctatcatgggtattagagaatacggcacca
ttcgcaggggcaggtacgaagaaaatgcaggttcgaaagaattggtttttatgaccaagattga
aaaaagacccaataatgtagctgaaaatctcattattagagttgccaaccagcagtataatgtt
atgaggatggtgtttttatagactacgaaacaaagaagggggtgtccaaggaggaaatgttta
taccatataatgttcagaaaacaaaggctcttaaagggcgtagtacttacttttcattcgtaag

gaaaattcctgatgaaccagaagggagtatcataatacacgcactagggttttattgaggaaat
aataataataataataatggcattacaggaaaaggatataactatagggaatgtttctgct
gccctacgagagttgatgtactcacccacacatatgcagcatcacgataagctaaacacattcc
tggacagaaatgttgaatcatcttcagaagagaaaataagacaaattgtggataaaatacgatc
ccaaacaacatctgacatatctgaaacagtcaataatgtcacaactaatgggactgcattttcc
cttttcgaagataccttagaaggtatggtgaaaaaaaatataggtgataaccttcagagtgggg
actttattgatggccgtaaaaagctcaatgacatgaagagtctagctactggagccatcttatc
tagacagcgagatttgttgcagaaagtataacaggaacaaaggactggctcaaggctataatg
ggttgtggtattataaggtatactgtatttgtcaataaccttgcaagatcaacactcgataatg
atgatgacaaggcagcaacctattataacacccctatatatggcgggtattgtaaaatggctat
aaaggactatgaaataccagattcgtacagcaaggtcgaagcggaacatacagttgaaggaaga
aagatgacctttaatataaaatggagaggcgataccataaacaacctaataacaatcatccctt
cagtgacaggttatcttgcttccatctctgaagacgcagatgtgcaggcgccattacttttaaa
ctgcaacaactgttttatagaggcagatatgagtagcctctacatggatgagaaaaaaacagag
gcatcatttaccctcaacttaccggaaatcgaaggagctgatgcgaatgcagtctatgaaatat
gtatagtagtagtttgatggaggacaaataaaaaattggatacatttcatgtagttttatttaa
ctcttaatcttatatacatttcatgtacatttcattaaaaatcactatcagagctatagtacag
aaactggtgctggagctgattaatcctcctggccatgacggcagatgtcctccgtgaagtgttg
cgtctgccgtctctgaccctatcaaccatcctacctgaacctgggttcaaattaacatttggtg
catcaaaggggggtgtgaagaggtgaagaataaggagaaggtacaggactagcatcatagtccct
cacaatccttgttcttccccttctaacaggtgtggatgcgggagggggcatattagccgtgtct
agcaactccaacatctgtgcctggtattcaagtgcgttagacacaaacctctcagtgtcttctg
cttcattgtcattattaggaataacaccataatcttccatgagggcgtcacgccttcctgcctg
atgcctctcaaagagccgttttctttcctcagggtcatcagagaagagtttgtgaacccagcat
ggtttcttagcctcccctacagccaagtcaggcacaaaaactggcttatttttacattcttat
agggaagaagagggggcaacaccttgacaggagcaggagtggggggtaaaggagtggtatattc
acccttatatgaactaggtttatggaactcctggcaaggtattttgccctccctgtcctctttt
agaggggcggccttacagcctccaaaaaccttcttagctatttagtatgcattttctcaatcc
ttttcataccttcagacaacatcatgaacccattcttatcaaactgaaaacccctcaagaaaaa
cttataaaaggacttttcattctttgcaagaagacatcccgccattagcctaattgcactcaaa
acatcccctatctccccttccccatactttatttcaggaaacctattgagtgctgagcaggtac
tttctatagatgctctcagagcatcattttccaaggttgatgagctataatcaaccttcttaca
gctggcacacttcttaaccatatcctttatttccattcttttcttatgtttcttcatcatttct
gatgaagaggaagttttgattgatgtcttaaagacgatattcttccctttttattgtctttcctt
tcttgctgacgatacaccctcccttcttgctgctgctgctgccgctgctgctgctgctgatgat
gtcagttccggccattttttaatgactctaaaaaacaaggggggtaacacatgtaataaaatgcat
tataatgtaacattcttttattttaataaaaggcagtagaaaatttgtacatcatttacaggca
ggcatatacaaaaacacatcactttatagattgggcagggaagatatttgatcagcttccgaag
ggtcagtatcaaacccattcaacaatgaactgtgtacatgtgcaatttcgtcatccaacctagc
attattagaggctgacttaatgagacaggaaccaattagctgcttattagcactccactttgaa
gcgtacttacgcttcaccttcaatttcttagagcacaaactcttaccaattacatcattgacag
ttgtatggaacaggttacctctagatatacaatctccaagcttattctcagtttcctgtctcct
ttctctaagagcagaaagttcagccagatacctattggcaatctcattatactcctcatcaata
ttatcgagtctacacatatacctcttgacatctacacacagtctcttactctcagaggaaatat

tagacatcctgtcctcgtcacgcccataccttggcctctttctactgttactactactactact
acttcctgaggaagtagagggctgattcactgatgacgaagaaggtattacttcttcgtcatca
gagtctgaatcaacattaaaacaaaggttaacattaatgttaagagaggttgaggtaggggcgg
atgaggtaggggcggatgacgtagaagcactaggcatattcaagtcaggcaaagacattgtgtc
atcatcatcatcatcatcattaacatcataaagtgaactactaataccactaatagtatcaaag
gtaatattgttacttctaggggaacagtcaagtcaggaagtgactctgagtcatcattatcat
ctacctctgggggacaaacactacagtatcctcatcatcttcaatgaaattgaaggatgtcac
accagacaacaaactaggggagacattattatcaccttcatcatcatcatcatcattactcaag
cattgagtaatgtcaatagtattgggagctacatacttaagccattcaccttcatattcataag
gggggatatccttgaatttacccattaaagctgcctcatgattatcttcaaaggtctcttcact
gaatgcatgcatttcctgtctagatagagtttcaagcatgaaactcaacttggtataagccctc
tccatataaacatcctcattctcattaacacctttggcattggggacaaagaaagtaggtgcta
gaatagttgacatcttattaagggtatcctttacaccttcatcattaacatagtcagcagacaa
caaaaatgagtgaaaagaggcatacttgtacacctcttcatacactttacggaagaaccacaca
gggggtatataggccctacacatgggacataacaggtgattttcattcaccatatgctcttcaa
atctccaggtgaaaagacactccaggtggacacttttcctgcaacacggcatccctccatttga
ccagttatagcaatccgtgtccgaatcgtaatcacccatacagatgggacaggtaggcacctca
tcaacggcgacggtttcagccatcttgttgttgttgtttactttaaagagggagaaaagcc
aacgctatagcaactcatttctttactttaaacaggttcattacagtccccttaaaagggaata
tgtgcttcatggcaagggcatattctgtgattgaagggcgattttgatagaaatcagtgtctgt
tccgctttgcaacaacatcaaaaagtgcatgtttgacatcaaattaaaggcagggccttccatg
ttagaacaagcaacaacatcttccagtatactggaagccattacactgaaactgtacgtcattg
aataggcacctagacagtgtcccctaaagaactcgggaggtgtgtgaatgtactgcctgattag
ttcttccgtgttcctaaaatattcatcgttgtaacaataacctagagggtatgagagtcccaag
tcaatcatttaggttggccagatatgctatctataacaaagttatcagcctttatatcgacat
taataattccctcatttgcaactcttgtaattacattacaagtctcagccaaaacaaaaggcat
gttcacctttagtttttcaaacatacttcttacaacctttcgggcaatgaaagagtctgttatt
ggtgcgactgtatcaaccccctttacctctggtaaacgtcccatcctttgaagcatgacaaccc
catacaacaagcgtgcgtcccttgactcataatactccccaccatctaaactatccttgacaca
cctaatgacatcatcaatactgatacctgcaaatggcatctctaaacataacccctgaggacac
acaccagtcaccccaactaccccccttaatattctttctgtatacagaaaggccattaacacact
caaaaacaaactcttcatagttcatatccttcataaacttgacaacagtcccccctattcacaat
gtaatagaccccatatttaatctcaaaaaaggtctcccagggacagatgaaagtgttaaagtcc
ttcacatcttcaggcacttcgaccaaatcagacaaacaaccacgagcagtcttacggaagtttt
cccagttctcacactggtcagaaacttccttaattagcgtgctagtcttttgtctaattcgctc
acaggcacttgaaggcaattggaacacctctacagccatatacattatgtccataatctgggag
gacggaatattacgactagacaactccttaagaatgtgtctagtgtcaaaaaatctcatctctt
catcgcaagaagaggtgggccaatagaagccaactttagggtcctttccatagaaacaggtatc
atggacatacttgctagacttaacaattgacctatggccaattctcaactgttgacaggtatca
taattgtcctcaagactacaactcttctcaatgcctttaaagtagtaaggggaatcacgttcaa
tccactcattatactgcctcttacactgcctaatcaccttggacgcacacacgtttgaaaaggt
gcgagtagtgggaggagagggagcaacttcagacttcttgaaacgtttagggggagggggcgtg
atgctaaggttgatgacgtcatcccaagtcttgatagtgatgttgttgttgttgttgttgtaag
cgggaacagttgtggcttcggaggttgaaccagacgcgtagttgttgttgttatattcttccac

aggccgtttttattcccctgatggtaaacatactgctggtggtggtggtctccaccagtattg
atggtagtagtaattacagtgggtccccccattgcctatggaagaaacaacaacaaatacacga
ttaattcaacgcccggcacactaataaagtgatgtgtttttatataaaaaatagcccataata
tttaacgttacgttaatcaaaaaaaacaacaacaataatagtatattattggtacttacttttg
atgggtttggagcggagactgaagtaaatccagaaaacaacagttttactgttgggcactacac
tgcactggtgacgtagtagtagaagtagtagtggtaatagtccactattgtcactgcactgcac
tagtagtagtagtagtagtagtaagagcgttggataagctcgggttggctgctagagctggagc
tggaactggagcttgtatgcttggctgtgcctctgctgagactgatgcttctggcctgccccgg
ctcgcttatatacaagttgtccccccctcacccccacttccagaaatttgccgtcgaacgccagt
tctccaacagagtggggtccagatctggctacgggttcactcctggccataccctgtttagggg
tctaaatccacccccggcactaaatggggaggacctaagggtgttataacaacatgtaagcgtt
gggtaagaagatctggatctggatgacccaccttgtccttcttatcctatccttatccttgtcc
cctgtctaccaccacctcaccccatatctctcaccccatctcacctatacccccatctcaccca
tcacctctatattcccccaccttattcactcgctccagtttcaacaccctgttcttgccgagcc
aacccataaccagatctggaccccagtcttccctttttatccctaaccggcaccatttatgcccc
caggcgctagcggtgtatataaggcggcgcggccaggccagaagcatcagttctctgcaagcca
gcagaagagcaacacaacaagcactctctctccttctacctagaagagacctgccaatactcaa
gctacaagaatggcctctccagcccccgccgcaccaagtccttacaccatgttggactctaagt
tacttagttctgaggaactaaaggaactaacttcatacgtctcgactagctctcgccggtctga
tatgaagaaacacttgctccatctattcgaggagcacgagaagatcttccaattcatacaaggt
aagcacaagttctcactatacactttggactttgaaattttctatgttatgctgaatattttgt
tggttgaagtgaaaaatattctaagtccaattcctttactctttgacagaaatctccaaccagt
acggagactatggatgtttcacaatggccccgcctcacctgaacgctgcagccgatctcttgga
taaggtgatgtccggacctctatctcccgaaggcgcccaaacttcgtccccggctgcttgtgtt
ggtgcgaaggttgtgaaggcactggtgagcttttgccagaagacccgcttcaccaccaacattg
tgatgagagaagttaaagccatggagttccaaggagacgattttaactactctgccttgtgtgc
aagtatgccccaacgccccgtgactgagaggcagatgttcgcccttatgaagagtgaggacgaa
gaaatgggagtgtctgcaaacttctctccagtctctgatgacgtcatcaacccttcaagcctcc
cctctggacaagaagtcgactcatcaacttccgctcaaatttctggtatgtttcaaaacgtgtg
gagtttgcttgaagagtgtggtagtggctctaatagtaatagttcccctgtctctaggacagtc
ttagtttgcaccctgtttataatccaagtgttcaagtttttggtgactaaagtgtctaatgtga
acgtacttaaccagttgtttggacatgttgtttttggatcacttgatgtggctccaagtaataa
taatagtgtcccatcaactgttgttaacaacaacaacaaaccctcgacctctaataatagtaac
aacatcagtaacaagcgtgttggtggtagtaataacagtggcggcggaagatcaaagaaagtta
cagccacagccaaaaatccctttaataatgtagatggggacaatcatggcatgtttgccggtgc
ccctgttgatgttaatttggatgactttgtttttccccaagttgaaactcttacaagtaagagc
accatccctaaagaagaggtaaatgtagatgaagatttgagtaaaatgtgccgtaaaactgccc
ttacccccctagaaattcatacctttaatgtgttcatctctgagattaacccctccaaatatga
ccgttcaatgttttgcaagggatttttgactgcatgggataagtttgtagagggggatactgct
ggcgttaaacgcttccgtaactatatcctcactcgctcaaactatgcctcagccgccagggccg
tgtatgaagcgtcaattaaggggactgtttattataatgacaagtcaaagtttctgttccacga
taatgttaaccctgatctggacaagagctggggtaacaagaatgggaagaaacctagactccca
gctaacttgatggcattcatgggtattgacattgtaaaggtgtgcgctaaggggattcaaaagt
atatgtttgcaaagcaattccaacatccggaagtggaagaacttgtgcctcctatggctgtata

cgcaaaggttgccgcaggattgaagtcggggactttgtttgatgactgggacctgcctgaatac
gaaaattgtcagtttatcaagtatgacacagaagggtgcaaaaagcacagtgagttatacgcca
aacaacttctccgcacaggacttaatcaatacaataaactggaagagggacagagtgcattccc
atttgcaaatattgtgacggtaacatccgcctctagtgatgatattcacggtgacacaatcatt
gaattgatgtacaagacaaaggatggcgtaaagggagtctcaaaaattgaggacgaaaacatca
tcaaggtgaatccagcagaagaaaagaagaataatagagtacaagccgagaagaccctgtattt
tgagattgattccgatgatgaggtgtgtgagagaacagaggaagaattcttcaggcctacatct
gttgttgctgccccgacaacacccctcgtaccttctaatgtggaggaagaggaagaggaagaag
agcagatggaagaagaggaggaagaggaagtagaaagggaagaaggatctgataaggaagatga
cggagacgcaccagcacaggaagaaatggaggaggagaaggaagaagaacaacaacaacagcca
gaagaagaaagcaatggtaatgagaaccaagaagaagaacaacaacaacaacaacaaccagaaa
gagaagaggagaataaggatgcagatagtgacagcgacagtgatagcagcagcagcagtagtag
cagcagtagcagcagtagtagtagtagcagcagtagtagcagcagcagtagtagcagtgaaaat
gaagctgaaaagaagaaagaagaggaagtacctgccaagattcagaagagaaagaggctaagtg
aaaggccatcagaagctgcttcctctcccaagagaatgagagtagaagaagaacaacaacaaca
actatcaccatcattggacatactccagactgcagttgatgagatgatggaagaaattcctgcg
cctgagcctatcgttgctacaacctcacccaaggcagcgacacttgcactcaagacaggattta
gttactcttcattcgtaagaggagatgacctttcagtagctggtaatacttcccctactgaacc
agcagctgtgcccgctgctgccacttgcacttccgatgttggaaatgacttttggacatgttg
gacggtttacctggcgatatagtaatgcaacctggcgaatgcgacgtgaccgcaaaattctttg
agggcatcaccctaccagatggtactgataatgaatgcacaggtttcgatgatcttcttaaagc
caccgagactgataacattataaccaccacatgctttacctccccgattcacccttctagcaac
tcagcccccagaaaggatattgataattgcagttctattaagaggtctagggcaggttcacttt
ttgacactgatgatgatagtgaaacaaatgaggttgaaaaggaagcccctaaacgtaagaagca
cttgaaaaagaggcgtaacaagtcccaccgtggttcctctggttctgcttcttcttctcattgt
atgagtagtgatgaagaatcagaggatgaaagggatatgaaatcaacatcaaaggttcacaagt
caccaaaagctcatgttaaacattcccctaaatatgatgctgtaaatagtgatgtaaataactc
atacaacaatgttaatagtacaacatgcatgtcgtcatcagatagtgatgcagaagcacagcct
aaaagccataataaaagccactctcgtaaacactcttcttcctccacaagtgataagaaacaga
accaacaatgctcaatcaatactcaaaatgtcaagaagactgttgtacagtctccacctagttt
tagaagttttagtcctaagaaagatgagcttggtgatttcttgtcacgcaagcacacaaagcca
gttaggccctataacaagaagcgtgataatgttaacaccactaataatgtagtacagaggtctg
cctgaccgactcaaatgatactcaatcaatgtacaataataatcttagtacttaacaagaagaa
ctatatttttataatatttttacatgtcttaataacaaacaaaataaaagaaaaccaatgtatt
atatgtttaaaatcaaccccatttgcatgattaaactaactatagtgtgtaaggaaagaaaaaa
aacatgattatttctgccattaaaacaacaacaaaaattctaagcttctcctttctttctgtgt
ctttgcagattcgggagtctttctaaaccacagcaatacaaaaaacaacacaaattctctact
gctctccctcaccaatcccccctatcgcccttatgctccttctcctctctatacttcctccccat
tcacttctgactatcaattttctgattcatttcaacaacaatgccaccaatacaattactaccc
cacacccccctccatctatgtcctcgacgacctctccatcgtctttgtgggatgatgacgatgat
gatgacgaagaagacgaaaaagatgtcaagcaagaagtctcgaaccgtccccccatttttttcgt
acatggaaactgtatcttttagtgataacgatgaggacgataacaagggagaagaagaatgttt
tggatcaaactttgatatgtttggtgattcagataacatgccatcaacttctactgcccctttc
cctcctccctctacaacaacaccacttcctactcctcgatccatcatggatactgattcggatg

aatgtgacgaagaaggagcagcagcagcatcagcaccgtctattgccgcctcttcttctatccc
tgtcgggatctctgaagctgaattgaaaaaaatggaaaagaaaaagaggaaggaaattaagaaa
ctcaaaaagatgatgaaagatcctctccctcacctatatgtaggaggagaacctcctgtcgcag
cagattataaaacaagggcaaacatttccctttataaagttgaccctagtatcgatatgtgcgg
tgtcgcccctcctcaattttgcgctgaattgcccaccccatccatagatgtgtatacttcttcc
tatgtatttcctcctcccacacctgccatgcataataagaaagggttcaagaaatgtcaattcc
ttaaggggagaaaggctttgaggaaatggattcacgagaatgtatgcatggcccctcccggtaa
aaggggaggtgtatttttggctcacttggaacaaagattcttggctgaacatggagatgaatac
aaggtcccaaggatgtttgtttcaagagtattgaacaaagctttccccaatctgattgctcgtg
cagacacactgtgcagtgatatgacattctatactaacctttgttggatagttaatggagttgt
cgtatgctttgataaagatgatggaggaatacatggcgatgcgtcagagtatgcaacaggagaa
aattttgatactgtagtgttccacaagagggaagaacaaaagaccaatgggagtgccagtaaga
agaggcgtctcacgcctgacactagtaatatgggaacaagcactgatgtgcaagaattccaaac
gatgggaacaaatactgatatgcaagaattccaatcaatgggaacaaataccaaccccatagag
acttcatcagtgggtgtgaataccaacccacttcccaaccctcccccaagattggtaattactc
ctttaacgaatgatgtaccagaattggacatgatgtggctttattcgccttccagaggaggtgg
aaattctagaatgagtgcaaatacaggaacatctcccctgtctaacaccccaattcctacctgc
ttcacaggaggtgcgaatgtagtagtgcctaatggatttgtccctcccacgttccccttagaat
gtgacgaagatgatccaagtattcccaattcttacaattacgaagaggataaagtctttcatcc
attttatgagtatatggccaaatatctatcccctcttgttccatcatataacaaggacagact
tgtaatgttgtccaggagtggttcaagggatccttctctcttgcaaagcgtagaggaacagtcc
ccaaattctgtagtaacatttcccacgctttcttttgtaatatggatgtatgtactgccatgtg
caaatgggcgaagactgtaattagacatggacaatattgtaatagatgtatcgtaaggaggtca
tgtacatccatgctcgcatatcactacattgtttgcagagacgcttcatgtgatgttcccaagt
gcagggaaagggttcgcaacgacatggatgactgattgattggttgatatgtgacattttctgt
atattgtgtaaataagataccaataaactaatgttttatatatgattctatttttttaaaaacc
tttaaaaatatacatataaaatgatgtatttttgaaactacactctggcagaatcagaccagac
ccctgacctaaagcagaccacaggggagtcttagagaggggtgtgaatctggctaggggttcat
ccctcaatgttaatcacacgcaagtaaaaacaccacttcctagaaagggagggaaggggtgact
tggtgatatcataactggggaatttcctctccagatatctggctgtacacgtgtgagcgcttct
gggcgcgacaagaaaaaaattagtgatatcataactggggaatttcctctccagatatctggct
gtacacgtgtgagcgcttctgggcgcgacaagaaaaaaattagtgatatcataactggggaatt
tcctctccagatatctgggtaagaaaaaaaattagtgatatcataactggggaatttcctctcc
agatatctgggtaagaaaaaaaattagtgatatcataactggggaatttcctctccagatatct
ggctgtacacatgtgagtgttctaatctcatttttttatatagaaaatataactgaattagctct
ctaaacttttcctcatttcttactcctctttgtttgtatgtcctactaccagtgtgcatataag
aactggagagatgggagttttaaataacaacccttcttaatccctttcatgtttatatttaata
aatataatcaccatggatatttcaaataagacattatttttagtagtcggtacctttttttctga
ccacttgtgcatcttgcagtccaactcaaatcgtctggaacctcatggtagcttcatttgttgg
atttctaggacataaactacttaaaaacattacacctgtcaatctggatcttgtcggaaaaagc
ttcgtattctctgcaagtttaaccatctcagaagaagcccgtttattgaggattggaaacgtat
taagggactataatggcaacaattttgaagagtatgaagaagaagaagatagtggtattgaaga
ataataataataatggtttgaataaaatatagagatacatttatattgttttatttgcatt
atatataaaaaagcactacaaaatttgtacacatacattgagagaaaaaattgatacaatttct

tcctttttttttactggtatctgatttcttgatattcgagagagttagtagtagcagaagaagta
gcaccaactccggcagcagaagttgtagggataggggcggtggcaccggtggcagcggcagcgg
cagcagcttcacggcgttgttgttgttccaggataatctttgcgcgtttattcatttcagtggt
gcgtgtaaagatgcctctgttgtattggaccaccttattcctatattctccgatgtccttatcg
cgcttctcaaaaaacttggccatttctccacaacgttgacgggctgcccatgcagtcatagcca
taaaatcaaattggtcagaggtgatattatggttgtttagaaggtccacagcgtaggccataca
gctagcggcacgttgaatattggagtacttgctgtttcttggtttaacggtatggatcacctta
cttccatcattcaagaattctgcctcttctctgtgcagaagagatgtgcctctgctaactgtcc
cgtcgtcgaggacaaaagcaggtccaggtatgggtgtttcgcaatctccgtaagaagtgtcagc
aaccgtgaagtgggaaggatatagtccattatctccaactccagaagtgagtgcagcgctataa
acttctccggtgatttggcgagtgactggattacttagccctgcgtcaatttcagacatatttc
cttcttcgggtgtgggaaggaagggcctaggtggggcgagagtgtcgttgtttctttcagccgt
agtagtagcaagagatgtgacccaagaatatttcttgttcttagactgtcttccagaaccgtcg
tcgataacaagagatggttcgtcctcgtcactgctacggttgccattctggttcttcttcctct
ttgaatccttcttcttagacttggacttgttcttggggccaggaacaggtataacatccacatt
gggtgtagaagacatcctcgttccagtagattcagtcagagatgtaagtgctggcacgggaggc
atggccgtaacagagataccttgtggtactcggaacattgcagaagaagacgtagaattagagt
tgatacttgtaacagcagaagcagaagtccgagaggcgatggcagcagctctggcggcagcaat
catagggggctgtagtggtgcaaatcggcagagtgttggatgaagtcgtggcagcagaggtagag
ttgatgtgagacatcttgatttcttggttggttggttggttgtggactattgtgttctcagaga
ggacttgtagtcgactgtgctctacccagatccacagcccctttttatatccgattttcgggggt
tgccagatactaatatactcctccacgccctcttcttgcgccatatctataagggtcgaaagtt
gtataccttgagttgatccggttataaccgccattgttgggtctttctgagaatcgagagttgc
gatcacggcatggcctcgtgggggaattttcattgacgtaagcagtaacgtcagaagaattgtt
ggagcggggttggttaaagtgcctctggtgttggtggcggtgttggtggtggcggtggtggtga
gcagcagcaggggcgtcgtagtgttcgtattgctcacaataatagtccttgggcacttgcattc
ttcttggatgaatatatctttctctttcttcgtatctgggttcgtcctctctttttatgtgttc
agtttttatgtgagttactctgtccatctccattttctcagaatttcttcccccctccattgacg
tcatccgaataactcaactggcgccggtgatactgatgacgtcttattcttcggaccttattat
cccctccttctgaatcatcagatgattcagagtcagatgaatcagacgaatcggatgagtcaga
ggaagaagaagaagaatcggatgagtcagaagagtccatggagccactcccaattccactacta
aattcagcttccaattctctgacgacatctatatttttagtatctttttcttccacatcttgat
tcgtattagggtatgtcacaccagtttcgttgctttgttctgcgaggatattttcgaaaccttc
aagaaaaggaatgtcaataaaaccttccatttctacgatctccccttcttccaattcttccttt
ttgttctcttcttgttggtttcttgagacggtggtttcagtaaaatttgggttatctgtagatc
caaatccaccagttccacgcacgcttcttgcttgatttttgttgtcctttttagaactagagtc
aataatcgttctctctcccgtggtttcattaatatacacaatctgttcttcctcgacatcacaa
tatcttaagaaaatcaactgggcaatgctggttccctttctgattggcacactttttttctgcac
tatggttgcgcagaatcactttcaattctcccctataatccacatcaatcgttccagtaggtac
actagtgttgttcttccatgtcatcccagaacgtgacacaatttgtccataacagccgtcgga
aacttgtctattccatatccagtagagatcttggccagtcccataggttcgatatccatttctt
cagagggaaataggtcgtaggcgacagaaccgggcgtggcacgtctgggcggaagtgcagtttc
ctccccgggagggcgaatctcataaacacgacagatgcagatgagtccatggctggatacagg
gagtacacagtggagacgggatgtaataagtgttcaacgtctggaggacaagtgagtaccttcc

tcggttcctcccacccttttataatcgtgaaaccccacacccccgctaaggcagtaaaaaaa
ttgacccaccatcaccccttgtccttgaaggaggaaactagaagataacttttatcatggcaac
atttactgaacaggatcataaaaatgcgtttttatatgctaatgagaagctgaggcaggaaaga
atatacagacttaaaatgtctgagccttcagtttatgcttttattgacataaaagaaatagaaa
atggttgggaaaaagaattcgggcttttagtacaaccaggacagaaattagctcctttcaggga
tatttcttatgactcaagcaaacttgattgtgacgcattttcttgcataccttcagatatactt
cattctgataatgaaaaaagagtaggagagtgcaactttgccgaacacacctctgtctcatttc
ctgtcaagaaccctgagggaaaaacattgcgccatttcacggcatgtggtccagggtgttaccg
gaggtacaagcaaagagacccccatactggtttgccagtagccagaggcgtcctgatgcaagat
cacgttgaccatgaaactggaaataaaatgtgtgaatatctgaaccagagcttagtcatgtggg
ctgctgtcccttggatacgacctggagaccttactgaaggttacaacacaacacacgtgcctgg
ctttgcattcaaggaggatgacgaaagggattcaaaaagagttaaatatgaaaatgtggtcatt
tcaaaggcttattgtgatttctttaaacagtattatgacgcagactctggctcatgttatcgat
ctggatggatgaaatttgtccatttaatgtttgggcagtattttactaatctttcttacaattt
agctaacccaaaaccttacaatttaactggaaatacatggtctgatgtagtttctgtattgaca
gataaccctattgtagatgcgggtgctgctccgtccagatctgaaatggatgaaattatcacca
agaagaagttcaacgtattcccttctgagcagacatctgctcgccagaaagcagagaatataat
acgttctcagtacggagatggtgtagaaatagacccatcttctgtggatgctttaatgcagttt
gttaatagggaaggtgtagtaggaacagagaaaaaatccgaccgcctcatgcgagtggcagacg
ctgttatggatgcggctatgcgccttcaagtcatgggtctggacgacagtcaatctagacgatt
attgttaaaaaatatgattaaaatgagtagaaacaacccagaatatgcaagacatttttccagt
tctctcaaattaattggagtaactttggccataaaaagatctgttttctctaaaggcgcttcag
ctaaaagaaaggaaacggccattaataatggcgaacagcatagaagaagcagatggtcacccga
gacggtgacagaagaagatgcacttttatttgcaagagaaaatatcactgaagacccaaaacac
cctgcgccctttgtggacattttacactcaccagatatcaattcatctatcaagagcggttctt
cttcctcaatatggaacgatatactttcaagaatttcttcgaccagaaaactggaagaaaaggc
gagtgttttcgttaaaaatcttgtggtgaaagtagtgagacagttcttggacattttagaaggt
aaactgttttcggacggatacgaatgggacgataatattcctctgatgataggcgtggaccaaa
tattgagagaagtcattaaggcggccagtaatatgtgcgctagatttgcctcctcagctctgga
gtctagtttggttactggctttatagactcggcgagtgctatcacttctaggttagccgtacaa
ctggcagccagaacgttctccgtattcttggaggagtctgttatagaatttgtagttgccgcaa
gccttcggctagcgatacaggcatttgccgatctggcaactcttgccgcgtcagccttaaccgt
tattggaattgttatattcgtaatacaagtacttgggttgattttggatcttgcgctagggtta
ggttggtacgatcacatttttagcccagaggatttaaagaagcaggttctagtgtttaggagag
agtttgcaaaggcaggaaatgtagatgtgggtgtagctcaaccagtcacccctgaagaaatcgt
cgctatcaacgttttccttcaaactgaagaaaacggggaagaaaagaaggaagaaggagcacga
aaatcaaagattgattttcttcaaaaatacttccattctactcctttgatggaaagaaaagta
agtttgtctacatacaagaagcagctcaagaatacttgggaggaagaacaatgaacgcttttgg
gcagcgtataataacagctgctgatgatagtgacaccaccaccaccacacaagagggaaggagg
gatgacgaaacagtgactaagaaaatgaggagtattattctagaaacaggtcaaactcttaagg
attactcgtctgctgttaactataacgcctcccgtctagattacgtgggagaggaatgggtaag
aaatactgccctaaaagaagagacaaggagcaacactactagtgataacctattcaagaaaact
gtttctcttgctagtatggccggcgcatttctggttctaggaataggtgtattggtagcgtccc
atattacacttttacgtttcaccaatattggtctagcttttgcgttcgcaggtctcctagcatt

tattgcacttatgagtatatcatatataaacatgaatgctatgggtgtagtgaattcggacgca
atatacaggtctactgctctagttggagatatcaaaacagaccccagaagagtaggaatggtcc
agcgccacgtaggtgtcggggctaaatacaacatgattacagatttcgtctctccaatgttaga
cgagatcgagagtgactaaaaagtgagggttataacccccccccaacaaaggctttaaattag
actcaaaccaaattcataatcattgtgaagacatcgacggtcgaggaaggtccgtgctcaaaag
gtcccaaaaatattcctcaatcaaaaaataccatggattcgttgactaatacagtaacactcct
cgtgaatgaccgtcttggaaatcatcgaacaaacaaaccaatcaccgaacaagatgtggaaaat
actcttaaccttaacagtctagaaagggcaagtcttttgaagttatattctgttttcatcaaag
aaatgcagtcctattctggatgtatacccaagaacaagtacacgaatgtgcaagaaatattcga
agatggactaattactttcgaatggagagatggaacaaaagtacacagatcagtttcaccaagt
tcccctatacctctttctacaaaaaaatcgcctcggtcctcaccttcccctcctccatcgatgc
cttctattaaagaagaagaatttgaagaagaatttgaagatgatgaagaaatatacgaaacaga
tgaaaatgtggaagatttcataaatggtgatggagaagattcagaagaggaagaagaagaagat
ataattgttgatgacgaagaagaagagaatgaagagggagaaaacaagtacgttctcgcattct
ctaatcatctgaggcgccagactgccgccgctgccgccgccgccgcagctgctgctgctgctga
cattgagaagaaggacaaaaaccacgcagttagtgcgcatgactacacactgtccgccctccag
caacagcaacaaaaactgctccagcaacagcaacagcaacaacaccagcagcgctcctcatctg
agaaggtcacctccacacccaacaaattcaacaagtttttactgccgagtaatggcttctcaga
acagaccgagctctttgtttgtttcgatgtggataaaattgcgcaatataatggactcgtggag
ctagacatcttacccattgttgctgaatacattatcaacggccttggtctgaaatgcagtatgg
aaactcccccagtgaaaccgtgcaggagaaaggaagtgaaagatgtgtggtgtcagcctaaaac
tagctttgaaaatgatgctgtggaagataaacatctcgcattcgcagaatcgcctatacttcaa
aggcctagagatttccctatccctaaaaaaatcaccgcctatttttgtttagacgattctgtag
acattaaaaaccccctggggttcgtgtccgcttttgaaaagtggatcaaactttcgagtgtccga
atattcgcgtcattttaatgaattctcaggagttaaaaatgacgatgacacgtcttcaaacact
tgctttatatactctcaaaaaaaccccaacattgaaattgtatcaaaattaaatattgaatttg
aggtaatgatggagggaattataacccatagaaaagatttgttcgagacgggcattttgagcga
ttcttcattagctacggctatggcattttgccacccaaaagctagagttcgaaatgttgcattg
ttttatttttctgtatatttaccattctctaaaataactcgcaaagaaactataaaatgttcag
agacggataaggtacatattggttcagacgcgatcttttctcccccaagtgataatcctaatat
aagtgctcaccagaacaataacaacaataataacaataataccagtgtgaatatcgaggacaga
cctatccgaaataataatataagcagaaaaatgaccatcacaaactaccaatgtatggcatgca
aggaaagatgcacaaacaattgcactaacggtaactatcccgatcgtggtaaccagcacttgtc
acatagtgtaaaaggggaagatttctttaagattttaaataatagtaaagtagattcattgaaa
aaattgagcagagtactgattcccgctcctccctctggaaattatacatctaagttttgtgata
gaagctctatgtgccatagcttcttttgtagagggatcgaaccagtgtctacttctttctcatc
ggacagttttgaaaagactaaacttgttttgtatggtaaagtcgttgacgttatcaatagttat
tctgccataaaaacttcccataataatagaatcagggtctttttttaactctgaggaaaaagata
ataagactatcccctctagagccgaaagtgcaaaaaatgcattcaaggatatacttgttcacga
atgtaataaagaacgagctgtttcatattttgagcaaaacaaattatcctctaaagatgggcat
ctatctaacaagtggtggatcgaacttaatgacttgaacattatgtttgagaaacacgtggaag
atttttacaagaaatgttctaaagtaaatgatgcagaatctttaaaggatatttttaatgattt
tgaaaaaacttgtgataaatacaaaactgccaagagggcaattattggagcacaagacccttct
acttctactccctctaaaaaggagaatggtatcactaggattattagtacattatccgaatttc

attcaaaagatgaagctacagtaagtgcccttctcgacaaaacaatgctcttgggatcgaggac
aataatgtctggtgttagatgtgttatacgtaacaatagtgtgttttcgggctttgaaaataag
aacactaataataattgggaacttgagattagacactatgtcatctctatgggaggtgctgcag
tgacaaagatttccgatgaagatttggaacaattcacgcctgtaagaggtgctgtctctgtcac
tacagcacctaatgataagctacctgtaggggcacatcagacatggaaggatgaacaaacacta
aaaacaaacactaaacgtaatagtctatatgactcttacaattcaaaaaggaataatagggata
ataataaaataaaaaatcgttcattaaaactatcagattttaattggagaacacccaatatctc
tattcaagaatttaatgcaaataaagatgatgttaacaagaagaggtacgcagaagtcgtggcg
tcagctgctccaaagtcaccttcaccaacgagcagcagcagcagcaacagcaacagcagcagcc
ctcctctttcaccgctctcaccaacagtgaagaatagtaataataaaccattgtatattcctcc
ccataaaagaatgaccactactgctgtttgaccaatttatgggttattttataatgttgtgtga
tattttatacaataaaaatatataagaaaacaaccaaatgattttgtatatccttgttctgtgt
tgggcggaaattcctccgcgcccattatgatgaaaaaattgctagaggccttgaatcctggtca
cgacaaggttaacaacaaaaaaaattacctgtatgtgcaagtcatatcctccagtcccacgcat
gagaacctgttattgcaccacgattcgtgtaacaaagtcgtatctgcacatgtgcataaattcc
ttcacgctaaaatgaacgacaaaattgccgggagcaatgggcgtgagagccggttcctggaaaa
ccccacctccgttccacgcactacctcactattgcaccatttttggtgtataaaaaggcacgg
agaggtagctaaaacatccactcgcagagacatcaccgtcaagatgacgtctcctactagtgct
ctccgcgttgctactactgccctgctgtttgctgtggtggttacggctactgtgatttctgaag
ctgggggaactaagatagacgaccgctgcgccttcccgccatgtgactacttcccggaacctga
atgtcgtggaagaagatgtgttagagctgctcgaaggggaacaacaacaataacaacggaacc
cgtgatggagccaaagtctttggcgaaatcgcaaatggactgattgggcgtaagaggcgtgaag
cagtatctctaacaccagtccatgaagatatgccagatttcttcccctaccctcaccctgagca
tcccattggagggcgtctaccacgtgaagcagctcctccacttaaaggtgcacttggacgtaaa
aggcgtgaagcagctcctccacttaaaggtgcgcttggacgtaagaggcgcgaagcagaatcct
tggaggaagaacttgtgtctgctgaagaagaacgtgaaaagcgcgaagcagctcccccacttaa
aggtgcacttggacgtgaaaagcgcgaagcagctcccccacttaaaggtgcacttggacgtaag
aggcgcgaagcagctcccccacttaaaggtgcgcttggacgtgaaaagcgcgaagcagctcccc
cacttaaaggtgcacttggacgtaagaggcgcgaagcagctcccccacttaaaggtgcgcttgg
acgtaagaggcgcgaagcagaatccttggaggaagaacttgtgtctgctgaagaagaacgtgaa
aagcgcgaagcagctcccccacttaaaggtgcacttggacgtaaaaggcgtgaagcagctcctc
cacttaaaggtgcgcttggacgtaagaggcgcgaagcagctcccccacttaaaggtgctcttgg
acgtaagaggcgcgaagcagaatccttggaggaagaacttgtgtctgctgaagaagaacgtgaa
aagcgcgaagcagctcccccacttaaaggtgctcttggacgtaagaggcgcgaagcagctcccc
cacttaaaggtgcacttggacgtaagaggcgcgaagcagcagcagcagctatgcctcccccctga
agacgatctcgacttcttttacgcacctgttgctttgcctctacatggagtatggaaagcacca
gaacctacaggttaaaatgtggctgaacatcacatatacaagttaattggataattgaaacaat
ttgacttttagtacttaatgtttattaattgaaacaattggactctttcctacctaaggttta
ttaattggtacattgttaacacttgctctttgctcatagcagacatacctgctatttttaacgc
tttgtttaacgtgctttttacaattgatattggtacatgtttgtgacctcttgaataaattttg
ttagcatcaatatttattttatttttatcctttttcttgtgtgttagaattagaaggagatgtgg
aggttgttgatgtagtagatgctgtgcagagttgctcctggtgttgctcctcttcttgattcac
attgtcctcttcaacatactcctctaatccaaagcgctctcgaattttctcctttacaccctca
ccacaatctcctatcgaagactgtgctaatataatttttgtagtagaagtagagaataatggta

caacgttattgatgttgttacttctgcccatagctggtagtattttgaaggcatgccttaaaa
aagggatatatctttgtccttttctatacaaaaggataatcgttcatccccatgaaaaagtgtt
agatggggcaatgtacacctatagaacagtgttatttcttctatatcttccagtatttatacca
gaccctcttgccccatagtttcgtatctccattttactattcctgtctctctggtgaacccatt
ctgagccaggtgttctgcccataatttctgatgttttatcaacaacaatatcagctgaaggggc
aaaaggccccccatcttccagtgcactattcccattagaaggaagtggactagctggcataggt
gacaataatgaagggagtggtgaaaacacaggataatcatccacaccctctgtcactagagatt
ctagatccgggggaggaggtgtaggcaacataatttccatctgcatttcttcccccgacgtgga
cggttgctgttgttctactccttctacctctacttctactcttcctccttctttattatttct
gcttctagttccttcattaccatttccacgcctgttgctagtctcattttcttattgttatttt
ttatgcgttctttagcttgctttgttacttcatatgtaatatcagatgagtagtatttgcctgg
acaaataaggttatcactcatggctgtataattagtatcagaattgccatgtgcattatctttt
gttataaactctttgggtggtttatttgcaccagccgtgaaagtatttgccagaagagccgagg
ctgatgaagagttttgcttaacatacttttcatcgatagttttattaaataatgtaactaaact
ggccgaacacttactatcccggcaattcgcacatgcattattattcttcttttattatccatt
gaggcctcctttgcagtttcaattaaagctgatatttggcccatgattttaaggctcgcacaca
gtgccatttcctgtcctcctttctctagttgaattgatccccattcattaaatttttcttgtag
agtcaggagactactcttgagaaaagagtgtaaaagtgtttgagtagttatacaatttttcaac
gtttcagtgattgggtcgaacccgaatttgatctgatttcttacactatccagtaaatccttgt
ttaattcttccgtgtcagaggaagggaaagaaattatatcattagctggaattgattcagaaaa
gtgaatatttttaagtacatttccgtctgttgaagaagggtgctttagatcacacgtgctaagc
attacatctaatgataagaaaggggtaactctttctcctgtatcgagatttcctacaacatttt
ctggtgtcaagagatcgtctgtttctgtcactcccaccaatctcttgtaatcttcctcagcaac
agtttggttgttgttatagaaagacccataagtctgaaaaattctcctcgcagtctcattatct
gcatcattagctcctcctcctcctcctcctcctgccacatttaaaagatctaggtagctactca
tctgggagggaagggggagggttaagggttgtatataaagaggcttgtctgaagacgcgagtgat
cacaacttcaacgatggacgtcgagttcggtttcttccacggtctgctctccaaggcccttctc
ccagatgaaaaacatcaacccgttataaggcgtctttgtgcggatgattctagaaataagggag
aggatggctgctgctcgttctgtggaagaagaggaacaggagagagcaatactgcgtgccttga
acaactaatagacgtttgttcctttataggaactgtctcatctattggtacaattatcaattct
aatctttcaaccagttgttctagactacaaaaaacgtcagacagttatgcggcattatcccatt
ctagttttctggatgtggtatatccaagtttgaagaaaacaactgaagacgtattgcctcattc
tttacgtgccatttggaataaacaacttccaaagttgtatgaaaaaactcttcaacccatagaa
gaagaggatattggttataaggattatgttgtttcaattgaagacgacgacaatgttgatgatg
gtgaccaacaagaacaaatgattattgatgaagaatcttataaaactattggagaaaaatcaac
cattgaactgataggcatgtataacaataacaagtttggtaatgaatttataaggattccttta
agagaaactgcgttgcacgcacaatctctgaggtacgacactgaagctaaatttgttaaccaca
aggactctatacctctattttatgaaaacagcacgtgcacatgtaaggaacgtcttattgattt
ttctgagagacaactacaacaactaaaacaagatggaatggataaaccaacggacaagtagaga
ggatcttttcaacacgtatacagggaatgcagtaatacgttcggcagctaagcaagcactggct
attgaaaaacacgcagcagaaagaagaggagaaaaggcatggacgacttcagcagcagcagcag
cttcttctaattttaataatgtacaacaagattatactgatgatgatattacacaagtgtctat
tgcaaacagtgttttgaataaccccttttaaagagatatgcaaaacttatagataatttagca
atatcttctttacctcctgatatagaggatgatgtcattatacacactagagatgcctccaact

ctacagtcagagtagatggagccaatatctatttcgccataattgacggtgatttatgtgtata
ccctaaacaatatatatctgataaagtgctgtgtggttctctcaaccgggaaaaggcactgttc
tataatagctccaagaataagtggacgtatggatgtaacctaaactttgatatcgttgacgctg
ccatcatgaaacaccccgactacaaggaagagactacatctacaaaacatatacgtaaaatatt
gggtatcggagcatcggaaaaactgaacattacccactatttaaactactttatccaataaatc
accatgtctatatacagcccagaaatgtcagccatgatgaatgagaatgaagaaaaaaacgttc
tcagtaataggagggctgtaatattgatctttttcataggtgttgtcattttatcactcttgtt
cttaatcagactagatatagatatcaaatatgggaatgtgggcgagagagccactattgtaaag
aagactagtcccatttttaaggaacctatccagacagccatcaactttaacaacaaaaagattt
cccacattgaagatggaggagaagaaagtgatgttatagcttcaaggactgttagttctctcca
gagagatcctcgtgtacttttaaccagttcatttggattgtataataggacagattatatgaaa
aaataaaagtaccaaataccatatcaattgttttatttcatcattagttaacatctttttaaat
ccacaatcagagctaaaacatagtgcagttatacaatacaataatacatttctccctccaatga
tacaatgttgatgttctcctcccatatacttttcaatcaaagccgagtcttgttccttggataa
tttatccatgaccagcttagaatttctccagtacatgggacgaaacactaacctcatactagga
ttcgtatcattgtgggaagggtttatacaacttcttgatgtctttacatcctcagtaggcattt
cacccaataaattaaacacatctcttataatagaaacagactcagaatcatactttaaatcttc
tatgcattcacctcctatttagatttattaaagggtgcaaagaaagggggtttcttccttcttc
ttcttttggacatcatttttacctaatacacttaaacctatctgttctcttacaaaggactgaa
aattgatatctatataatgtacatcttcaaatttttggatattgcgattaacatttttgtatga
ttctggaacagggatcttctttgcagtcgtcatcattctggaacacgtgcaggcaggttctggg
acaacacccatggatagggcgatagcattggccagtttcactagccacatttttgtttgtggat
taaaagtcttcttctttccaaaacgaagaaagaagacgctacatacgtctccttgctcgtcacc
aattacacgtattgtgtccctttttgtttaatattttgtaggcaaattccagtagtacttccttt
ccttcaacaagaggtattccaagttcttcaaaatccttttctaaataatccacactctcctcta
aagacaaggaagaggatgtgcatttgggcagcaagatttctgtggtgcacatattattattcaa
accttgagaagaggacatttttctctttactgttcaggacgaggagaattgggaggagttatgt
tagttagtgatacgtcttgccgctgctgatgctggaggtagaatgaagaccacagcgtgagggg
cgaccatggcttagatatggccacgccgagcactggcatgaattgcccctgagagggtagccat
ttagtgttttcactcggtatttttccctctttattgagagttgccaaaagtttattatagttgt
ctgtgcatgtcgataccttgtctttaaaatcgtccaatacttttggaatcctctcccagttctt
gcgttttatttgcttgattctgggagtctttttttagttcaggcatatactgcaacattgtgtat
tctagtgcagcagacataaaattagttctatctgtgcatattttatcctcttctgttttaccac
aaaattcactatgtattatgggcattaattgctggtacacaatagtattatcctctagaccgca
catgttcatatacacgataactaaaaggttcatggtttttacactataaggaacaccagagaat
ctttcatcgtcgatgccttgaactggttcatagagcaccacgttactattgttcccgtcttctc
tcttattggcgtccattaaaactccacttagacgtgataataaactattgtcattgtacgttat
attgtcaagagaaatgcatttgtcgaattcgttaacaatttctgcccacttttcttcatccatc
acgggttgtttattgttaagttttgatgatacagaacatcttgaatgttcatcctcttctatat
tatttttagcgagcgatgacgtcatttcgttcgtggctgcagttacagcttctaaaaagtcaga
atctttatactcgaacgctttccttgcctcatctaaccagtcgtcagtttctagatctgcctcc
tcttcctctttttcaccttcctccatttttttcctcctcctcctctacatcatccatttcattga
attttttaaagaaatctaaaaagtcactcgtgctgttattttgttcagtttttaagattgacgt
ctgctcagtagatagacctggtattgacgatactattcctcctcctcctccatttcttctccc

aatgcttctggattcgtatctaaattattaccaaaaacatcaatcctttctttagtcaatattc
ccctatcattaattataccaccttcagtgtggctctcaaaaaatccacgaattgattcgaaaac
tgacgatgataatttttagtaacaggtgcactttttctggttgcattaaccttacttttctta
cgggtagatttttgagggtcattaccacttttacgtttattattagaaaatgggataagatctt
cagtatcagattctacatccgaccctgtatttttactctcattattattagcaccggaaagatg
tatactgatatctgataaggttgttattgtaccaaaaacacgttgattaaaggaccccattgtc
aaatatgacatgacatttgccgctgcatttacagccagggctctatgaagtgcgtgtttcaaac
taacgtcagtttccaaatcaatcaagtgtgttggaggaaaacacaactgaagcgggtctcgtac
aaaatctatagcttgagacagatagaaaaatttgattgtccagaaaggaagtactagtttctta
tcacattttctacaattacttttagacaaattccccatagaaatattaccaacactatcatcac
agtctttattattcttcctagcttcttgttcactcaaaaaaatctcccatccgcccacacaagt
acagtacgtgttttcaaatactgcaacttctttccttacccatgtgtcgtactcacagttacta
acaattgcactattgtagtctgatcctttagttagcataatcaatagtgtactttcaggtgaaa
gcatgagcggcgattctgccaaattatatatcgtatttggctttgttcttcttttagctgcaaa
accacccaactcgtttgcttgattttcacccgaagaattactgttcatgcacctaaagaacttg
acatggccaatctttaagaacgtttcagagtccttaaattgtgtaccagtaagacgacacacgt
ttttatgatgtccaaccatcaggttccatttcaccagcacatcagaatcgtgggagaaaagatt
catagaaagtgtttgattttcatcgttggtgtgttgcttcaaaacattcaataaatcttcccac
ctcttggattcttttctgttaccagacatattataagaatcgtactccaatatcttcctctttt
ctagtagggagaagtgtttcccaggagtctccagattcaaaaggcatgatgttaaacgtacgat
atcatcctctgcttcggtgaatgatgcattagaaaggtacatgcatttgctccccgtagtcttt
agaacagggatcctcattatgtccagtaatgcagatgccatcagaccttgaggaagacgcatca
tcatccgctcgcacgagtagaatagtagatcgttggtcttcctattgaggcaattttcattcct
tgattttgccttttccttacttctttcttgtctgatctgtcgtttggctctcgtcatcatcttg
atggctggagcctttccatccgtaaagagtacagtatacctccctttaaggttttagacttga
ggagattttcatcactcggatcatcctaaaagcgtagtacaagatgacccccctccatctggct
agcaaaaaatgcatctttgaatctggatttcaaaactgctgtcggggaagaattgaatgaagta
gtaggcacgttcctgaacaagaaactgttagtcaaaaacttcctcttcttcataacatctgtgt
gtttgttgatccagtaatcgagaccctcatagtcgtccattttgtgattagaaactggaggagc
catcatcatgtcttcgattggaggttcagttagccaatcaacttcctcttgggatgaaggatt
ataataggcactctttcaattctagccttaattttctcgtgtttatattttctagaaaagaatt
cagaaataaccttcttttcatccctgcagtaaaaattttcataaatatgtttcttgttctgcat
ccgtgaatgcatagtatcttcttccctgataacattaaaaccgtcatgtaaaacgtctattatc
tgttgatttgtagtattgataaacaggatcatctgacccatatttttctgtcccttcaacatgg
tggttgcaatttgcctgcccaacatgaccagatctgatacattataagtgggcaaatcaaccat
gtcgagtgtgtagtcacaggtaggtgaagaagaggaagatttcttgcctcttccaaaatccagg
aagttcttggagttgatgttggcaagagaagtggctttgagaagtgagctagttgtgcaatctt
cttctcctgaacaataatcctcgtcgtcttcgtcatcgtaacaatcttcatatccagttttctc
cttgatgactctcccattctgtgcaccgtacgcatttcgtacactttttggcttctgggaca
cccatttctgttgatgttactcccacgggatgttagacgataatgaatgctcactgatgggcgc
cgtaattttacacactctgcgtagtctggtgtttttacccctcctagaagaacttcgtggagc
tgctttaaaggttgtagtgcttggaatctgcagcaaacgcagctctctacagagacttctcgga
gacttgccagaagcgagtttcctcttcttaataggggtactttcttcctcttcctgttgttgtt
tgtagagtgttatagattcagtagggaagtagtttctagatcgaagaacagtgtagtcttcttc

ctctttcacagtctcaatagaattggacacctcgttgaacacttctagggccaattcccacaca
ccttgttccttaagtgtctcgaccacgtaatcaaattcggattcactcaataactgtaaggagg
aagaatctccatccatgatacgaatctggaacacctgtctcctccttgactgttaactgatgtc
tgaccccttgtaaccactttactgatgttttataccctctcccgacggaagatgtatacgtcac
aatttttttaaaaaataacgttatacaccaaaaatgagcgatacagggcagatggaagaaaat
aggcctgctacccagaaaaggagacctggagatgaagaagaggaagaaactggtagtagtaatg
ttccatattatgccaactttggcgatgacgccacgtactccatgtacactggagaaggaaaaag
gggtaaatttgtattagagccacctaaagaaagaagtgtacaaagggtgcaaaaaccacctaaa
gaaaaggaggaaagggaacaacgttctaatgttcggacacggagacctggtcaagagtttgaac
agaaagtgctacaagatcgatcgcgagaacggtcagaaaaacttgggcaaatttggcagagaa
aggattgcaagaacggcaaaagaaatatactccaaaggtagcacaaacaatgacaaaaaaaata
atcaggtttcgtgaaggaggaagaaaattcaaggcgccgcaacagcagacatctgacaaaggtg
cagcaaccaatgttcttgaaagggaagaaattgagatggctgcagaaagagaacaaccagtaga
aattacaggagatactatattaggtgggctaggagaagaagatgacgaagatatgggagaggat
gaattaactatacaacattcatctatggctgtatcacaacccgttcaacaaatcgttgtcagtt
ctcctataccgccaaagcccactaggcccgctcctgatattcccatacaagaagatatagtggg
gaaaaatattagccagttaccaccattaccacttgatgattatgaggacgaagaagacgaacat
ttgtacgaagaagtgaatgatttcttagtggcaccaccaacagcagcagcagcagcttccacaa
gacctcccaggcctaatattcctcctccacctcctcctgttgttgctgttgcagacgaaacctt
gaagaacttggcttcaattgcagccttggaaaaggaggccgaggaacaaagagcggccgcagtt
gaaagggaaagagaagtagaggaacaaagagcggccgctgctgctgctgctgctgctgctgccg
cccaacgggaagcagacgaaaaaagggaaagagaagcagaggaacaaagagcggccgctgctgc
tgctgctgccgctgctgcccaacgggaagcagacgaaaaaaaggaaagagaagtagaggaacaa
agagcggccgctgctgctgctgccgctgctgcccaacgggaagcagacgaaaaaagggaaagag
aagtagaggaacaaagagcggccgctgctgctgaaagggaaatacttgcccaacaacttcaaga
aatgaaagaacaaatgcgcataaaggaagaggagaggcggaaagaactagcagataaggaggaa
gaaaaacgtcgagaactagcagccaaggaggaagaaaagcgtcaagaaatattagctaaagaag
agcaacttgaaaaattgaatttccagttgggtacagaaatcacgtccaaaagagcactcgaaca
aatgttagaagaagagaaggcctcacgctcacggtccgcagccagtgcacagttagcgatccaa
gcaatagaatatgaggatgaacttcctcaggcagtcgaacctcaaggacagttagttcctatgg
atacggatttgtacggaaaaatgtacgatctcaataagaaattagaagtacagaataatacatt
aacttctgcatttgaagacgtgaacaaaacaaacgaacagaaccaattggttgctcaatccctt
gaaaaatccgctaaagccattgaaaaattaactagtcaaaaacatcttcctgtggatgatcctg
cttttatgcagagaataataacagagaggatttttctttaaagaatctgggaaatgtttacaa
aagagttctcggggtctattttacattaaaaaggacctttttaaatcgaaggcattaattaca
gataaagaatcaagggatctggaggtgcgtctaacagatgtatcgacagatctcagggctaatg
atctcaatacaatactggaaaggttggatgtatccgttaacatacgctctggtggaacattata
cactaaatttacagaggcagacacggcattagcagatcaagttccttcgaggattgaaataagt
aacagatcaagatctgccttattgccattttcatctgcaggtttggatactaattttactaata
gttccgacaagtacaatgaaatagtgaaccaactaagcagtataaatgaggctatgaatatttt
gaaagaaaatattgtcccaacattgaaccaaatcaaaattgatgtcaccaatctattaacagtt
tcaagctctcgtcaatatgctattgaagaaagggtgtattctgatgtgtcccgaatggattctg
aaataagaaaattcctcgctataatgaacagtaaaatatccccttattttaaaggcgattggac
ggatgaaagacaacgctctattgctgacagtatttcctctcagataaaatcaaacgataaaatt

aaagagagtgttgctacactacacgatatcaatacaacctcaagaatacgtagtaatcccctcc
tgcacaaatcctcagttttatcatctccagactttttaaatgctgttaacgactttagaaattt
tctcgatatccaaggaggttctcaatttacttatgatgtcctttcaggccaaaatattgatgac
ctttcactggcatcaaaaaccactgaaaaggttacagaattgtgcctcgaattatccataattt
tagacgtgatccataaaaatgctttgagtttaaatttgcctgcaattacttaccccgcaggga
aacatctatggaagaaagtggttcattggctgttgacattagacaagaaattggtaagaacata
tcagattctagcgctgaacttagtcgcacactgtcagaggcgttacaaatttttcagcaacaac
agcaacaacaacagcaacaattccaacagcagctgttacaacaacaacaggaccaacaaaatca
acaacaattattacaacaacaaatagaagaacaacaacgggttcaggaacagcagcaacagcag
caaagggaccaacaacaacaggaacaacagcaaagggaacaacagcagcaacagcagcaaaggg
aacaacaacaacaaagggaacaacaacagcagcaacagcagcaacagagtgaccagtttcgaca
acaattattgcaacaacagcaacaatttcagcaattactacaacaacaaggaagaagaagaggg
ggtgacgatggtgatgaagaaagagaggaaagagaagaaggggctgaaaaggatgattgtgtgc
gtaaggttgcagaatcagtagcgacaaaatatactgctgacttgactaccttattccaacgaga
agaaaataacttccaatctaaaatagcatcagcaaaattgggaacccttgtctttgccacccct
ccttcacctatcatgaacttgacaaaattgagagaggaatattccacattcacaacccagtgtt
tttcaaaactaacagctgaaaataatagtattatgcgtattttccccgagaggattgtagaagt
atgcaagagtaagaatctcaatttaatgggaaaatacttgtacattataactaccgcacaaaca
gaaatggaagatcgagtgaagaatatattgtctggtattttcaatcaaattgaagagttttcaa
acaatgtaaaacaacaacaacaacaacaagctgcttcagcttcttctactaatcctcctcctcc
ttctactccttctactactcctcctgttacaagcatgcaagtttgtgagttggatgatcaacgt
accctagaaaaggctgccatagtagaggcaattactctggccaatgctgtacttcaaactacaa
aatccgcttcagctccttccacggcggctgagcgagaaattgctctaaagctagagaatgggaa
aacatctatccgtatggaaaaagtggatctaagttcaggagctactggtgtttccgaccaacaa
aaatggatcgacgaaagtacttccaaacaagaattggaagatttcattgcagaagaaaactttg
tagaaactgcacataatgaaatggatattggattaattttggatgccaagaagaacgatccgac
ccgtgatgccaatcttaggctcgtgaaacctcatggaataaatgtgcagtctttcccatattac
gtactccgcacatggctaggagaaacagatatattggatgaagatactgtacatcctgaatatt
tccgccaatacattgatcgcaattggaaggtggaagaacatgagcgtgaagatacattaaaggc
actgggtgtttctttatcagatacgttggcacacatcaaggactactattctcccagtgtcaaa
aatgatgcatcaaaatcagtaccatttgcgttgaacactctattgtacaacatatttgctatcg
acggaggaatgatttctagcctttcaagaacagcctttatttaccgtaaattttaaggcaatc
tatgacagataaagaggttgctcaaggacctgttcggtctcaactgtgtgaagcgacaatagcg
tctcttttcacggcatgtagtaaccttcttcgatcgtctcccttagccgataaagtagaaccac
gccttcaagaaaaattagcggccgctgctgctgtagacacgtcaaccggagacatgtttcgtat
acgagtttgtcatctcatgtacaattttatagtggcttatgtgaacctatgcaataatcgtata
aactatacgttaaatgtattgagagcgtcgggtctggcaaacaaaaaagtcgtggccggtaaaa
caactaaagggcatacatcttcttcccaccggtttggatcttatgatgtcacatatgattttc
agtattgtacaagattcttcaactacaaaaacagaatatctctttactttagagaagggtttc
aatgcatgggaatcgtgtgttgccgccatggcagccttcactgccgacccttctctctccatct
cagatgccgaccaatccatactcttcccactagaaggggagagattgttattgaaaaacatga
aaacgatgcagaaaaaaatgtcgatatggttcaagaattgtggaaggaaactgcactcacactc
atggcaaaggaactaaattcatactacaactggttcatatacagtaaggataccgatatggaaa
agttggcgagagtgtgcaggatgattatcggtatagttaaggctgttctcagattaacaaataa

agccgagagtttagtagacactaatgctctttctgatattttcaagctacctgtgattccaatt
gatgataccaaaactctggcaataaatattgtggtgttcactttgaataatgtaattaaaccat
ggatggtttcgttcaagcaaatgtttagacagaaagacgggggagtttcttcagcctatttctc
gtttcaaaatatccaacaacaaaaacatcaacaaactgcttccattttagatgcatgggcttgc
gcgcccggtaagttaacaaaggcagcacacgtgtttatttcaggatatgaaaatcatatcaaat
taaaaaaagatgatcttttatggggtgcttcaatgaaattccctgccgacggacgaggcactgt
ggtcgaaggatgggcccaacaatacaataacgaaagcgtattggaagatttaccgacttttca
attgaagtaaacgcacctgcctctggactcttaataccgccagatcctttactttcttccatgt
tcggtaaaggaaatggtggaagcagcagcagcagcagcaaggataatacaattattggaaaggg
aggattaattttaaaccgccaagttgttggacaagaacaagcaccaccaataaacacgtcgtcg
gatactaaaaaaataagacgtgatgcaaatattgaaccaataatagggacgccttatagtgtaa
ttaaggcaagtaaaggagtatcaatttcagtactggatgatttcaatgaggatagtccagaaga
tttcgcccttaaaacttccatcatcaatgatgccatacgagaaatagggcaacgcatgacttat
acaagacctatatttgatcatcaaacacagaaaaatatacactacagttcacctaaaattattc
tcgaaggatcagatttaaagaatggacaacgttcaggacaatcttgggctccttcttcatcatc
cttgactctggcctccgattggaatctaccttctctggagcttttatatagagaacttgccaca
aaacaagtagagaaggaagaagaagaaaagagcgaaagggaagaagataaaggacaaaaactta
atgaaaaattatcatttgtcgtgaataaagctatcggaactatccaacaacaacatcaatattc
tgaaaggggaggaggaatgaagaggtatcagcaacactctgctgatcaagctagtaatggtggc
atagatgatatagaacttatgaatagtaaagatgctacttccatgagaaaggcaaaactggcat
tagccgttactaataaaattgcagcagcagcagcaagggatggggaaaattcatcagctaaacc
gtcaaactttggcaatagattggatgaagcaataaaccctggagcacttttattacgtagagga
ggaggagtaagaggaggacaaacaccccagagttcaatgctaacaatgttccgtcctggacaaa
ctggtggcaatagtagttggtggactactaatacaccccttattcaacgcacaactagtgttgg
gaataatttagttgtgcttgtacccaacctattggattcacaccctcctacatttaattaggaa
taaggaattatacacatttatttgtatgttttacatgtattttattcaacaataaaggttatga
tcatacataatcaactggttgttttattacatagtttccattactgacccttccaaaaatgaat
tttgtacctccgaatacaatttcttccctcggtttacaaaggaaattgcacggagcgaatctgc
tcacccattcaccaacaacaccctcattaatcttcccatgagtgatgtgaggattcagtctgtt
gtcagcgagttcaacatttctccctattgcatctgaaatgtgttccattattcgtctacgcaaa
tcgagtgcatcatctccaacacatactttggatacgagaatgttgttgttcatcccaaacaact
caaaattatccccagtgtcgattttgatagctggtgcaaaacaactcccaaaatctgttttcat
cagagtattgtattcttccatatttcgcaaatagaaaacagaaatggtcacatgtggaggcaac
tgagaaaatgatttggagacgtgtttctcagataataattttcccgtttctgaatagaaaggaa
gtaactcttcgctgtagagagcaaagaacattttcatttcttcacctttaaaatcaacttgatt
tatagtgaagattctattgttgtgtttgattgaaaattgtttcaccacttcaaacatgccattg
atttgtttgtacatttgcttgactgcgcatcttctttgcacctgtggttgagtagatgaagggg
agatttggtggccattatctgggtggtatccattcaatatgttgtagccggcacaagggataac
tgggtccacaaacttgaaagcctctctattaaaccctctccctagaggaataagtccacaacga
atttcgtctgcgtcttctgccttgtccattaacatcatatttagatgcacaacatatctcagaa
tcccattcaaggtgtgtttttctggatagaggcaaataacatcatccacatattccccatctac
ataaagtccagtaacaacaatctttaacgctttagataaaacaatagtttctctatgagaaaat
attgccttgtttactttggtggagagttgttcgtcactgggaatgggcgtagaggccaagagta
gagccttggaggaaaatttcttgtccataggaagtctgtgccggatattgtctaccatgttaga

catagaagtattatcactaaagtcaaagaagactactccatttctggagagggtttgttgaag
gatagatgttcttccatggtggtggtggttgtgatggtttgaatgaggtgtggtacagtca
ttatatacacgtgggccctttttaaaaggggagggagggggagagggttgccatcaagcaata
tcttcgtccagaaacacctggtccagaaatggccataagatacttcctctatttctggagctat
tacatttctggtgtaatctgacattggcccgacccagaggtccaccctcggaacttgacattcg
gtcgagctagcggtccacccccctaaactggagcgaccctaaaaaatttttgaaaagtttttgag
atggaggaagagtaaaattctctagtgaaaacagaagggtataccctctcatttctgggtcgac
cagtctccagaaacgcctgttccagaaacacacaaaagttaatgtacgtttctggagctattac
atttctggtgtaatctgacattggcccgacccagcggtccaccctcggaacttgacattcggtc
gagctagcggtccacccccctaaactggagtgccctgaaaaaatttttgaaaagtttttgagata
gaggaagagtaaaattctctagtgaaaacagaaggttataccctctcatttctgggtcgaccag
tctccagaaacgcctgttccagaaacacacaaaagttaatgtacgtttctggaaccaacaattt
ctggtgtaatctgacattggcccgacccagaggtccaccctcggaacttgacattcggtcgagc
tagcggtccacccccctaaactcgagtgccctgaaaaaatttctgaaaagtttttgagattgaag
aggagtaaaaaactcactatatgaaaggtgtgtagaacaccacatcctttctaggcacggccat
gtccagaaacgcctgttccagaaacacgcaaaagttaatgtacgtttctggagctattacattt
ctggtgtaatctgacattggcccgacccagaggtccaccctccgaacttgacattcggtcgagc
tagcggtccacccccctaaactcgagcgaccctaaaaaatttttgaaaagtttttgagatggagt
aagagtaaaattctctagtgaaaacagaaggttataccccaccttttctggatgcgactagatc
cagaaacgtatgttccagaaatacccataagtcctttcctctatttctggaaccaaccatttct
ggtgtaatctgacattggcccgacccagaggtccaccctcggaacttgacattcggtcgagcta
gcggtccacccccctaaactcgagcgaccctaaaaaatttttgaaaagtttttgagatggaggaa
gagtaaaatttcttgcctgaaaggtgtccagaagtgtaaacacacacacgcattctcgcacctt
gtcaccatcattaataaaaaatgtcccctcccgctccccccctcaaaatcgggtataaatagagc
tcaccagcacaccacagacatcattctcaagacatttcaagtactgagaacatcctctcctctt
gtgatattattcaagaaaacctactgaaatcgtccctaaaatggattttgaaggaactaccagt
tctacccccctcaaaaatgtcccagttgtattcatcagtgaagaaagttgcagagcattcctttg
ccaatcttcatgacaaggctactcttgcatcaaaggttattaaggacctggaaggggagaggaa
gaaaatgtctaccccaaagtcctcttctgatggacaaaaactggacaaggctatgttggacgat
attatcaacgagtatcaggccgttaagagcactgcagataattccattgaatcgaccatcaagg
aaattgaaaatgtacttgaaagtgtgcgcagaaccaagattgaaagtgaagccaagaacagtgt
aacttccagcccagaaaaagtgttttctgtcgaggatttagaaatctactccaagggcgagtg
tgcaaaggtctcaagttaaacgccaactgttcaagaattggaggcaagtatgcagtgtcaatga
gtatcaaaaaacacaacgtctcctcatttgagaacaacaacaaccaagttttctctgaagaacc
cagggattgttttatgcttgaaacaacctatcctcttgttgggttcgaaacttctacagaagat
ggaaatacatatgcagttttcttgactggtgttgggctagaaagatctctacctaaatatgtac
cagttttcgacatgaatgcaggtattcaaaccctaaacatgactggtttgaggatggccaagct
tcctgttctgtgcatgtttggacgtacagaatatgacaacttggaagatttttacatcacttca
attgagacgcagtcttttgacgaagaggaaaatgatgccagaatgaggtgtcacaccgaagatt
tggagaggaagaagcgcatgaatgacgcaccagcgattacacctcatgtggccgtgtacgacta
cagtggagacgggaaagaacaattgctctatatgataaccgagtatgaaaacacggctagttgg
tgcaacgcaaacggtgtggtcacatctgacagtggattttctaacgaatgtgcaattagtgata
tgaatgacttgtgctgttttgctgactgcatcgatgttactgttaataatgaagaacatgaaga
acgttctatgaatattgtggtcgaatctgacaggcgtctttttgatgctagtccttcccccatc

aagacggaagaagatggagaaaattcatcgtcatcgtcttcctctccaacagttcctcctcca
caccatacgaaggtaacgcagttgtggagggggaggaagaagaggaagaaattgatgaagacga
aagtagcaagtatgaaggttcagaagatgctcttgttatgaagaaattagccaagctttctact
atgaaacaaatgagaagggttaagaatgaacctgcactcaaaattacttctgggggtaacaata
gtagcagtagtatcaataacgaagatgatggtgatgatgacgatgccgttgacgctactgcatt
atgcccccaaactgaagctacagtgaaaaattccttcatggccccaaacgacgagagaactgaa
aatatttgtatgaaactatgcaaaattctcttgctaaaatttgtaataatccatcatctatga
gcagttaccgtgtattcaccaacaaactccaagagtgtttgaataccatggacgatagtatccg
tcgccgtccaaccatttggactgaagaaagtcaacaatttgctaagggtttgttgtttgatgag
gttgtcacatcaattgtggcacatcagatggctcaagatatttgcaagtctgaaatatttggag
gaatgtttaacgccaactctaccaacattaagggtaaatatgaaggacaaaagaagagtctgta
tggaaacaagcacatttcttcctcgtgcttcaaaaccaacacggaatctaatgtgaataatgca
ctatttgcgtgggtgaaatcgaaactccattctggcacagtcatacctaacgtattctccttca
aaatggcatcagaaaagccctcaaaaatgaagcgcaagcgtacctctagtgcttcatcatctaa
cgatgaacaccaagaaccatcaacaaaaatgatgaaaaatgatgaaggggaaaaggttgcacaa
gaatcatcatctccttcttcatcgtctacaccagaacaacaacaacaagctggtcatgacaagg
aaactatcaatttaattcccctcagtttcataaaaatgccacgcagtaatgtcaatggctcggc
ttcatatttgtctgaaatattcggtcaacgtctttgtggactgtctgatgcttccagcacattt
aagagaatgtgcaagacttttgaagatcttgaaaatgaaatcatgaggagctcattcactagac
tgactagatatgagagggaggtaactcgcttgtatgagaaatgcaggtctcaagctgtagatat
tgaggaaaatgaaatggatgttttgtctcaccaaggggaattgtttgccgagttcttggaggac
ccgatcgcttactttgaagaagtactggagaatattaagagttggagcctagaaaacgttaaca
cccctaagcgcaaaaacaagtatgcaaaggtactggtgagcgttaatgctattcgtaggacata
tgaagaataccatgcgtttagcaagtttgtaccaatgttcttgttcaatctgattaagagagaa
ttggaaggagacaactatacccatgacgttcacttttcttccacttgcctgtggtacctgactg
taatgaccaggaacaggatttgcgatgtgctccagtacatcaacaacaacaataatgataacga
agaaaccgatattgtggaggaagaggaggaaggagaaggagaggaggataaaatggaagaaagt
atggacgtagaacaacagaagcaagttcgcaagggagggagaaagaagggtcaaaaattcaaca
gtattggggatcaagtcattagaaaatttgtgaaaagtttgtgtgaaaattcgatggtagtttc
tattgcaattaatagtttgatctctggaataagctggatgaacaagaaaatccctcccggtttc
ttgaaggattctagcacaatcaataccccttgatgaggtctcaaggtttgtgtttagcgatgtca
aaatcaataggaaaatcaatggaacagatgataaatatgaaactgtttttggagtcagtacgcg
tgtggattcacatattgtaggcccctttagtatacctgttgatttttcaagcgcaggactagat
aaggcctcatgtggcaaattgtacgttaacaccatagacggaaagggcattttgacaatttcac
ccaaatatgattcattaaacgatgaggatgttgattctactacaacagacaagctagagaagga
tattttgcatttgtctaagcatgacacctttttcaatattaataagaataaggttcttccattc
tataatatttctcctagctcttctctcactgaaaagaaaaagacaaaattcaataggaagaaga
tctcatctggtatgagcaataataatggcatgtgtgtacaaactccttctagttcaaattcagt
ctcttccgtctcgtctattgtagctccttcatcttctgttctggctctatcttgctccctttct
tctacaaagaaaaagagcatctggaacgagaacatgtttttgacatctaggaacatgtggaggt
gtggatttgttgtaccacccaaactttgcagttttattgttaaccatagacacgctgtaaaact
tgtagctgaaactgcacctaaaacaaagttgtgtaggaatattattgataggaataggaagatt
agatttaacggtctaaagaaggtatgcaagagtgttagcgcctttaccggcgagtctacatatt
tgctcaataagaatatgactgcaacttcacctagtgatttgaacctatgtatttatacttcatc

tttaaatgacccattgtatacttgtaaattgacccatgaagagtaccaggacggaaatgctttg
gatgactatggtgcagtttttgtaaactatacatttaagagtatcaaatcctgctcgtccaaag
atgaaaccgctgacgacaatgctgctgctgctgatgatgatggttctactacttctacctcttc
ttcaactgatactgatgctgctgcaatccaagactttatgcatgtaatgattaagaaaattgat
gccatgaaagacattagaggaaagtacaagaaatctttggcaaagaaaacaaagaagcattaaa
tgtatacctttattttaccaataaaaattatatgttttacatgttgtgttttattcaaattca
tatggttcatccccatttaaatcgtctaaccccatatcccagtcaaacgctgctgctgctgctg
ctggttcaatgtctgagccatagatggattcattgacatctaaaagttcatctctaaaatttcc
tggtgtacgcggttgacgaataattggtgcaataaatgcctcctcttcttcttcttcttctcct
ctgactcccgtttccttgacagcttgacgttgcgcttttattatctcttcaacgttcctccctg
gagtagcgggaggaagatcaaaatcaatactgccaagctcagagtctatatcagtaaaggacat
atcagtagcgtttttcttcttctgctcttgctgtagacggaggaggaagaggagtaggcttgggt
agaactggtgtaggtggaggttgaggggtagtagaacgagagtcaaatcttacacgggaaatag
gtggagaaggagcagtattaggtataggaggaggaggaggggactagaaatgagttgaaccgg
aaagggctgctgctgctgctgctggggaggaggaatattactgggtgattgttgttgaggagta
gggggaggaggaatattattgggcggctgtggtggttgctgctgctgctgctgctgctgcagct
gaggcggcggctgtgattggttgcctaaaaagtacagcgggtactgttgttgtaattgttgagg
agcatatgggttataatactggttatattgataataaggatttaagtatccttggtaagtaggt
tgttgaacctggaaaggtgcctgtggtggaggaagaggaaagggaggaggttggaaaggggaa
attgttgttgttgttgctgacgttgctgttgttgttgtttgggcacaaacactccagtcgataa
attaaatgcttgacttgtttcctccactgcactctttatcatttttcaaccggtcttgctgaa
gctcttgctaaaacaccagctgcacatttctccagcatggagatagaatgctctgatttactcc
tcatccctcctccaaccaaaagatccatagtcgatgacatttttacccacacgttattatttat
agctctaaaataacgaggtattgataggccacgtacagtggcgttagagttatcagaagctaca
gatacaaaattcttatcaaagttcttgttcggtaatatacgtgtaccaaacaggttattacaca
cagtagcaagttgttcaaagtatactcgttctttcacagttcgctgtatacgatcctccacgat
actgctcattaaattgtttatctctgttgcatttccatcgttaagagatttcaatatttttgaa
cttaattcttgccactgaatctctcctgaccgtatatctttcctatgggaaattgcccaacaac
tattagccagaagagctttcaccaattcagcacccacatttgtgacaacaaaagtgtttgttct
ttttcctccgaactttttcttcccttgttcagatgacaataaagatgatgaagtaggaaaagcc
ttggttttattggcctttaaaaggttagctaaaagagaccttattctgtactgtatagccttct
ccttgaccactgcctcgtctgccaagtatgacacgtaatcgtattgtggcgcattttcactcct
tacacctaaaatagagcccgaagttatagcacctctcaatggtcgagacacggtcatcattttt
ccagcaggcactgctaatgcctttgattctgtgtcacttctctttctttttttagagggaggaa
atatgtgctttttcatttgttttgcccgcttcgatacagatgtagtaggtgtaacatgttcgtc
ttcaacattctcctcttcttcctcctctccatctttttcttctttctcttcactctgttcttcc
ttttcttcccgctgctgttcctccccttccccttcctctttcccttcctcttcttcttcattac
ctcctcctcttccaccaccttcttgatcagtgtctctttttccctcatcattttctaaatctga
acaatcgccagcaacacaatcgccactccttgacagaattttactagtacgaatcttacgcctt
tgcctattcttactagaagttggcgcgtccaatttagagacctcctgacccatgtttcgttgga
atctggattcggcgtcatcctcctttttttcgttgttggttcctaattatatgctttttttaaagc
agtattaggtttatgtttaggtggcatatttgtaaggtgggataaacattacaagaaaacaaaa
atattcaagacattattttattttgtaggcaccgctgttttagtatgtacatcaaattcaaaac
catcttccattacatatatctcaatctcttcttcttctcctcctcctctttgtttggacaaata

actatcactacatttcttaaagctagacaacatagagtacaattatacaccttttctaaagca
gcctttgtagacttacaatcagaatacaatagtttatggtcaattagtatatggtcagggttag
ttatttcatcttcacatatccaacaaaaaactgtagggtacccatctcttctaccgctacaatc
ataacaagtcatggtaatacatccacctaattttatgttaggtgtcttacatttagggcatttt
acagtcttaatttcctgtccttctttctcaaccttccattctaatgtcctcttttcatccacag
tgaacactggatcctttattgattcagatgtcatgttttgacgtacaatatttacctgtttaac
aatttccattttgcaccttggttaggtctgatacccgataaggcacgcctatctacacaatga
aggacaaaagaatgggctctatggaacacacattctggccgctccaacaccatttcccctataa
gattcttgacaaacattttcgcccataatcctaatgcactctcatggttcaatttttcatagac
ataaaaataggttgcaattagaggtatactcacacctacaacttgttcaaaatctctatcatca
aaacccccaaaaacactgtttaagtaatggactttaaccctattctgaaacctctccctggtgt
tgtggattacagatatgtcgttgagaagccccttcatattctctggcatacgatcagtcgagtg
ccagttggctagcgctagaagaaccttgccctccggagactctgggttccatcctcgaatcttc
ttttcgggagttctttttagtatagaaacagcctgcctacaatttgtcacctcttcatttggag
gtacaaaatttgacgccaagaaaccgcaggacaatctcatgggccatttatggagtccgggacc
agtatcatatcttactagctcatcggtgggagggtcatcaaattcagcatcttctacatcctgg
aagtaatagtcttccagttgttcgggaggagggtacggcaatactggaggaggtacatccactg
ttacaatgtcttccatctcattaggctggtcacatacattgggtagtaaacactgggtacagat
cagggaacatttgctatcaccagtttctccctccaccatcttaaagagtttaacggcggtctg
aatctattatccaccacagtaaattttgaatccttgtctagtgcaaagtccttgtccatcttac
acatctccatgaattcggcctgaagaagttccacagtatagaagcatttctctgctcgcttgat
gagttcggtgatgagaagtttgttggctgcaatgtattctgggtcgacattttctcctgctaaa
atcgccctctcgaataaagatgaaaattcgtgacatctacctataaactgtaccatatattctc
cacaacatgggcacctaaaaacgtcctttagtttttcactaccagccgagaccttttcgtacac
atacattaggtgttttatagtacaatcagggcatagttttgcagaacacgtttcttgtcgtgct
tcaaaagtgtccatgaaagcatgtacggggaaataatcgtaagaggctttcatgttttcatgag
aacagttgaagaattttggtttcatgtccaaatccgcgtagcacaacccacatgttttattggt
ggtattcccactcaatatctttgtaacgtgtgcttgtattacctccacttcgtcagatggttgg
tacaccatcgccgcttgactttctccccaagactcttcttgcagtgtctggctaaccatgcatt
tggcgattgcttcttctagatgcatattttcagcgttttcatgttcgaggatgaaagatcctag
ttttctggctgaaggtatgccgtgtctagataccagggacagtggtgtattttctaagagcgaa
gcgttcggggagattattagaccagaaggcgacattacggatttctttggctcctctactcttt
cagtatctgctgccaaaagatagggaacattgtagatagagtatctgtacactatatcaatcat
cttggctatttttgttccaaaaagaatatggtgtaattcttggggcacatattccatagcgtca
tacacgggcctggcagcgtacatgcgaagatgacccttattgtagtggttgttggtggatggac
cacttaaaaatgcttctgttacggagaaggccatatttcccaaatcctgtcattgtttcttcc
ttcatgcatatcagaaccaaataaaacactaaacgttctagcgtctcttattttttgtccccact
tgcaaagcagagaagggccacatgtaccactccttatgaaacacatccttcaaagtgttgagag
aaactaccacatcttcataaatgacacttttcatgtggcggtgcttcttgttctgatctcgttt
gatggcaacaaggagtaagaaaatgcgtatcaaatccatatctgatatcatctcggcgtacgcc
tcacacaacgcattcttctcctccactgcatcgcctgcatttccacgtataatttcatccaaca
tcaagcgcgtatttttcatgacaaacatgccactataaatacgatgaattagatccttgtatgc
tggcccatcttgtttttttgcaactctttaaatgtccaatgaattcttgttcgttttggtcaaga
gttgtaaatggaactgggtcgtcgttacttttcatcttgatgttcatgtcgagggctttcttca

ttaatagagatatctctttcaaaagaccggttggattaatcacaactgtttctccctccaaaag
ttcatccctataagaattgcggttgcagatgtccagtatggcctccagaagatcaccactttct
cccactatacccacaattcgttcataggtggacctcttattatttctgcctcccattaggtcgg
tgatcaagattgcaacttctgggacagcagctgcaactatggatgttacaccttccatgttgaa
cgagggaatgaatgcgataaagtctggggcctccatcttatatacccttcagatgtgaaaaaa
acaacatgaggaaaaaagtaaaaaattcaatatacatgtatgtctttattggctaggaagaaca
catttcacaagcaccagggttattgatagaacataaaagagcagcagcaggatctggaacaaca
atagaggacttcttctcctcttcttcttctcggacaggagcagaaaaagctgcaacaggagagg
gagcttccttcttgacttcttggagtacattcttgtcgactgtgaactggacagctctagctgc
gccctttgtgcgtagataatagagagtcttgatccccttctcccatgcatacatagtcattgac
cgcaccttgctgagttcgggttcttccacaaacaagttgagggattgagcttggtcaacaaaca
tacctctctgaatagccatgtccaaagtagtacgaggattaatttcccatacagttttgaatag
ttccttggtcgatttagggatattaggaagcgtctgaatagatccaccacttgccataatcctc
tgtttagttactgaattccattctcccagttttatgagctctctaatcacatattcgttcacca
cttggaatgatcctgaaagtacattacgattatacatgttggacgttaaaggctcaaaggattc
agagttgcccaggatctgtgcagtggatgcagtaggcatgggagcaacaaacattgaattgtga
acaccatacttcataatgtcccttctcaattgctcccaatcgtgaattggcaaagagttgaaat
atatgtccctattcttaatattttccttccccatgtcaaattggaaaatccctttgctcaaagg
actaccctcaaacagctcatatgtttctccctttttctttggcaatttcacatgaagcttccaag
gcaccatagtatatagtttcaaaaatcctcttgttaattagtgccgcttcttcagattcgaagg
ggattctgagtttgaagaacaaatctgctagtccctgcacacccaatcccattggcctagtttt
catattggaaatgcgggtcttgtcaactgcatagaaattgacatcaatcaccttgtcgagattt
ctggtcatgattttacaaccctcttcatctcccggtaatcaacatagggccttagggaaggga
tgggagaatacttcacaaacttgttgactgcgatagaagccaaattgcacactgcagtttcctc
cgaatcactgtactggacaatttcagtgcacaaatttgaagacttgatgatgccgacattttct
tggttagactttctattgatggtatccttaaagcacacataaggtgttccagtttcgatacgtg
cagaattaatttggtcgaataatgcacgtgccttcaccacctctttacctttgccttcagcctc
atatttctcgtacaacgccttaaactcttcgccatggacgtcggaaaggccagggcactcgtgg
gggcacatcagggaccaattcttcccagccttcactctctccatgaagagatcagatacccaga
tagctgggaaaagatccctcgtcctcaaatcttcattaccggcattctttctgcagtcaataaa
gtccttcacgtccagatgccaatcagaaatatagatggctgcagctcctctcctcttgtctcct
ccctggctaacctttttcacagagacgttaaagatttggaggaacgccatgagaccagggtggg
taccactccatgacgaaatggggcttccttttgccctcaaatcatgaaagtggatgccgagtcc
tccagcagtcttggagatgattgccgcctccttaagagtatcataaataccctcaatgctatca
tcttgaaggcccagaaggaagcacgaggaaagttggggtgtgactgttccacaattaaacagtg
tgggggaagcatgggtaaaatagtgcctcgacatgagatcatatgtttcaatgacagatttgat
gtctgatccgtgaatgccgacagccacacgcataatcatgtcctgagggcgctcaaccaagatt
ctcttctttctatcagtgggggaaccaatcttgatcaaataagagtattctagcgtcctcagtc
cgaaacaggtgaagagataatccattttgtaatcgataacagcatcgagaatttcagcattggc
cataacattttcatagtaggtatcattaactactgatgctggtttaccagttccagggtggatt
gcgtgcctcaatttctgagttgttgcactaaaactattccactctttggtgtttttgtggatgt
tcgaacagatgaatcttcctgccagttttccaaaatcagggtggtcaacaatttttgtctttgc
ataatcggccagaaaatcgtccatttcttggaaggagatggtagcgggaagacggtccatgatg
tgagatgcaagttcttgagggttaattgcgttcttgtcaagcttgggcacatactggttgactg

gaagacaggcattttcaatcctcttgattatcttttcaagactaatttcttgcttagtgccatt
cctctttgagatgaatgattgttgctggttagaacccattttatgttattaaattgggatgttg
ttatttggagtattttcctatacgtcttctcggctcgaggagggtgaaagatgtgagtgaagag
gcttggacggtagctgtatttataccggcccggtgaaggtcgctggatatttttgaaggagggg
aagaaatatatagtattatacgtagttctttgatgtagaaagaaatcagaaggtgaggtgacag
ctgctgctgctgcagtattagtagtgcaggtgcaaaactgttcctcaaaaacttttaaaaatt
tttctggatcactcgagtttaggggggtggaccgctgtctaggcctgatgtcaagttccgagggt
ggaccgctgggtcgacccaatgtcagattgcaccagaaccgactgttgctccagaaatgtgcat
taacctttgtgtgtttctggaacagacgtttctgggcatagctgcgcccagaaaggaaggtgc
aacatactgttttcgctatagaattttactcttcctcactctcacaaaattttgaaaaatttt
ctgggtcactccagtttaggggggtggaccgctgactaggcctgatgtcaagttcggagggtgga
ccgctgggtcgggccaatgtcagattgcaccagaaacgactgttgctccagaaacgtacattaa
cctttgcgtgtttctggaacaggcgtttctgggcatagctgcgcccagaaagggaaggctgtat
cttactgttttcgctagagaattttactctttctccctctcacaaaattttgaaaaatttttct
gggtcactccagtttaggggggtggaccgctgggtcgacccaatgtcaagttcggagggtggacc
gctgggtcgggccaatgtcagattgcaccagaaacggaatagctccagaaatgtgcattaactt
ttgtgtgtttctggacaagtcgtttctggtctaggaggcaactagaaatataagcttgtagcct
agaatttagtacaccaaggataaagaaatataacaaataccatactatgttttatttgagtaat
atagtatccaagttaaaataacaatcacgcacttgtacattaatagtctgtaaaagatgggcaa
acgtacacctgagagtattgtcccgacaatcgtacatgaaccctctagtgcacgtatcacgttt
tcgccctattaccattgatgcacaaatttcctccttcattctaatagaggcagattgtttgtca
aaataacactccctatagtaacaaccaggatttccattggattgacactgtagttgttcgaacg
gttttcctgtataacatttcccagctgcgtggtagtgacgcacaaaactcatgcaagaatgtgc
gtgggtagtttcatttctaagaatctcccacgcactagaggcacagaaaaaatcaactgctgcg
tggcagtgacgcacggccacacacccgcattcttcacaccataaaggacatgattcgtgttag
tcgtcacatctctcagaaaccccccatgtgcattgacgtcatggggaattgctattgcaccacct
tatggtgtataaaagtggccctgggtggcatcggttagtagacagaaacaaaccgtcaagatgg
tgtcgtctattacccacctctctctgttgttcgtcgtggctgtagtagcttccgtcgtttttac
aactgaaggagctagtgtgagagtgaaacggtgtgctgttagcccgtgccccgacgttattgac
cccgaccaccgctgccaagggcgactgtgccgcaggtctactcgaggaggtgacgacgacgacg
acgatgacgatggaggaactttcgatacagtagggtctggtatacttggacgcaaaaagcgtgc
cgcacctccacctgaggatgaagaagaggatgatttctaccgcaaaaagcgtgccgcacctcca
cctgaggatgaagaagaggatgatttctaccgcaaaaagcgtgccgcacctccacctgaggatg
aagaagaggatgatttctaccgcaaaaagcgtgccgcacctccacctgaggatgaagaagagga
tgatttctaccgcaaaaagcgtgccgcacctccacctgaggatgaagaagaggatgatttctac
cgcaaaaagcgtgccgcacctccacctgaggatgaagaagaggatgatttctaccgcaaaaagc
gttaaactacgcacgaaagtgacggtggttgaagaatagactaatattgttgatatgttaaccc
ctttttttcatgaaatgtgtacacacctgctatatatacgtgcatatttgaataaggaataaag
tttatctgcatctgtattttttttattagtatcctttttgtgggataaataatcggtatttgtgt
ccaaagatagtggtgccatgatgccatatacaaagcacgtgatactttccaagaagcagagtcg
tatgaccattgacgtcaatgcgtgggcagaggcggaggtggtgataaagcgtttctgagaaaca
ttgggcgtatgacgtcaactacattattcttcctcctcctcctcctattgcctctgccagttca
gtattttatttttcttcctatacaataaaaagtatcagatgaatatttttactgtttttctgtt
catccctctttcctattgtaaaaaaaccaataactaactaatcatggataacttgaaaggggaa

tttgttgcgcttaaaacagacctcacccattacaaaacacagttggatagatctatattggtat
ttgttgatgttgttggtagattatatgttatagtaaatagtgaacaaacagctaaaaaggaagg
tctagcaactagagtggcaaagcaagccacagagatacaacaattcaaggacgaaataaacaac
aaatataatgctctaacaaatactttggatgatatcatctacatttttgatcatggagggagtt
tcaaaagagcaaaacataaggccataattgaagcgagggaatactctaaaccgctgagggaatt
agagtgcatgtttacgcgtatagcggacatgttaaccttgacttttatgactgtgtacaccaat
atcattactgaatttagacactctagtgaacaagccactaatagtataaatgtcaccctcggac
gtcttttcttgtgtgacgacttgtgcaatcaattaccaaaagaagaggaagaagaggaagattt
gaaacagaaattcattactttccatgcgaacctatacatgctggacacacgcctaaagaaagat
ttgataattttcaaagatgtcatacaacaacttcacgtgattttgcaaaaggatacctatgctg
taaaagaaggtgtggccattagatgtgcgaaacagatgaacgaaataagtcaatacaggacaa
cctcaaggataattacaatacattttcaaacattttgaatgaaattgtctacatttttgatcac
gggggacattttgaagaagtaaaacacaaagccataactctgactagaaattacttgaaaacac
tcatgggattaaaatgcatgttcaaacgcatatccgaaatgttgtcattgacttttctaacagt
gtacactaatgttatagcagaatttataaacgctagcaatatttctgatagagagatcaataat
tatcttgtccaacttgtaacatgtaacgaattgtgcaaccaactccccaaacctaaacaatacc
gtcccctcagtttgatagataacatagcttatttttctctttctgtccaaaaacatctgagtgg
gtttctttagtatgtggagtattattgtcttgaagctcataagtattcaacctctcctactggt
aacatctcttcctctctataatcccaatatggatagttgttgcctgatatcgaggataacacca
gaactggctggcaagttaacctggatcttcataccagaaaacaacttcaagattgtccagaact
cactcccagacgaccaagttatctcccaattcagatatttcgaccatagacattgctatacgtt
tatggagattttgatggcaaacattaaaatccaagacaggaaacaaaacaccacagccatatgt
gaattgacaactggaagagaaggacttttatgtagaagaaccatacctgtatttttgggttcag
aggaaaaacgagaagagttattggggaatctccctgaaggtgcagaaattttcaggcctagaga
agttatgcaagtaattggtactctcttggacaagaaactagaaattgacgacggtatagcttct
gtaaaggctgccctctgtgctggttcatcatcgttatacctaatcatgagccacatagtgaaaa
tgaccttttctgctatcacaaacatgaaggatataaacgaagaatatttcgtagactttatatt
tcgtcataaacaattcctcaaccctgaattcttcaagcaccttatatctttgctcaagaattcc
aggaaggaacatgttgcccatctagtaagacgtctagaacactttctcatgctatggacccttt
ccaagatgaggttcacagaaatggaagaaaactacttcccaatctccagcgatagtgattacgg
catctgtgaaaaatgtgcacgaaaaactcccaaatacaagctccgtatttttagggaacgaaaa
tgctgcgatagatgttgccgtctttatcaccaacaaccgcctccggaggtgtataattgggatg
gaaaaataacccaacaatccaataaaggctacattaatgcaggcgatgaaattatcggcatgct
aaactcaaatgataagggaaaaacattccctcctatacctaagatggttgtacgaagagtggtg
gacggtgtctacgggcaaggaactatcctgtcaaagattttgaagttcaggcaggcaaatatcc
ccacgtgtctattcgtgacatgcaataaatgcaataggattttcaggctcactatcttagggcc
tacaagaaacatcctttgcccaccttgcagaaagaaaagtgttgcagtaaatacacaacagaaa
ggagaaaataaaccttcgtttgtgcaaaaaggaacaaaacgtctacgagtggataccggtagca
acaagaacacgttagaaaaattctgttcctgggaaagattcaatactgaagttttgctcccttg
gcttggctacactattgagtctaagtggcagaactgggaatcttttctgggttattcgagtacc
agatataaggaactgtgggcctttgtgaacaaacaggaaatatcttccatgaaagactcctaca
taaaaattgaagacatcgaccagttattgaggagtatcttgcaagaccagaagggtgtatttga
gaccgtctgcaaaataaagagcagagatggtttgtgaattggccacactgattccgataccgac
taaaaaggctgattgatggcaaccccccccctccagactcagccgcatgagtataaatatggc

cacttctcacaccacagcatcattccctcgtcatcggtcctaccgtcaacttccattattactc
caataataccaacaaccccagaaatggagtcaatcaaactgttcaccgttgctggtctgaatat
ggagcaagccaaccaagtggctgaagaaatcaagtcagaatataaaaccgaggaggaaaagagg
attgcccaggaagtgtttgacaaattcaccaaaaaactcattatgcaagtagatacgtctaaac
acttacttacaagagaaaaccccaaccgttttgtatcccgccccattgtccatgaagatctctg
ggaaatgtacaaaaaagaggttgcctgtttttggacattggaagagattgatttcgaaagggat
cctaaagattgggagaaactcactcaagatgagaaggatttcattctccagattctggcgttct
ttgcatcctctgacggaattgtaattgaaaatcttacaacacgtcttcgtcaagtggcgcagat
tccagaagcgaggagtttctttgacttccaagttggaatggagagtattcatggcaacgtctac
ggagaactgattgatagactggtgcccgacgaaaaagacaaggctatcttgtttaacgctgcac
aacacttccccgccatcaagaagaaggagcagtgggctattaattggatgcaaagcaataacga
tttggcggaactaattgttgcctttgctgcagttgaaggaatcttctttagtggtgcattcgca
tccattttctggatcaagaacaggggtattttgcctggtctcacctcctccaatgagttcattt
ctagggacgaaggtcttcatcgcgactttgcatgcatgctgttgaaaaagggttttgttgatac
cccatcaagagaaaggattcttgaaattgtcactgaagccgtccgaattgaacaagaatttctc
acagtttccctgcctgttaaattagtgggaatgaactgcaagttgatgagccagtacattgaat
ttgtggcagataaactattggttgaaatgggactagaaaagcactataatgttaccaacccctt
cccattcatggacaatatttccctcgagaataagaccaactttttgaaaagagagtcgccgag
tatcaacgtgcccaggtcatggcttctatcaataagatcaagaaggaccaacaaacccaagaaa
ctggttctcctctcccaattctgactgcacctcctccagtctcttcctcatcatccgaacaaga
agatgttgaagacggcgtcggggactacatcagttatgacgattttagttccactattgtgtc
aataggttgtgtattgtattattattgttataatatttttaaaaaataaatgttctataagact
aaaacaatgaatttacttccaatattcctgacaacctttttgttgcggtagatgcatgctctt
gctctaccatctgccttttacctgatgggaagaaacaacccttggtttttgattctgtattaga
agaggtggtataccctacagatgtgtgtgggccaaagggagctggcgaattattcactggtgtg
gatcttttgaccctctgtataggaggtaaaaacaatggaggtgaatggtcaggaaaaggtcctt
gtccaaggatcaataacgctgtcgttgaacgagattactcccttgacgaggaggattgtaaagg
gtttagaaaggggttccgaattcctggcactgaccattttcatactgtcttttccctttgttgg
gtagacagagatatgcacgccaagtgggtgcgcaacaaaataaaccctggtatagtaactgatg
atgaagatttggtagattctggtattaggactaaatttaaatactcttctaaaatttttggtaa
aggattcaatccgagacctctttactccctcgactatcaagagaggattaagatattaaagtct
cattttaacaagaggacgggtaatttctttgctcgaggccacttggctccggctggagatttt
tcctcgcttcagagagatgggcaacttttgctctagagaatgcagtacctcaaatacagaacca
taacaatggtgaatggaaagatattgaaaatcgtgcaagaactacgccaggtgccgcgtgggct
gagactggaccaatatttaccaacacaagaagaaggaatatctagacaagaagaagaagtaca
tccctatccctcatgccctctacaagattgtgtacgacaagaataacaaggaattgttccgtgt
acagagtgatatgtcttggaaataaaatacataattaaagaatttatattgttttattttgtca
tttatttgatacatttgttgtatgtttaaagacaatacagataattttcctccatgacgaaac
actggtgatatgtctggagctctgatgaatgtcttgccagatgccatcctagtttttctcctgg
ggaaggtgacgtccgtttgagagagggttcgctgggtttcaactgcagccaagaaaatatccct
ttgtccagtgacggtaaattgtgtaccgttatttagtgtgatgacattatccctctctgattgt
ttcaaagcttcataatcttcctcctcatcttcatagtcatctgcaacattctcagtgtctaata
gaacaataacagagttgggggaaaaatcagtcgtgctacgtccagactggccagtatttttact
gctccatggtgtaggatcatgctttctaggcttgtctgaatgtagaccattatagttggctctc

cccatcatatcatacagtgtacacactagtgtattaccaatgcgttttccactcttattgttat
cacaataatcctcatcttctccaacaagagttgagtacacactggaatctgttagaattgtatg
agaaactaatgcctctttaaaaatatcgtcactctcttctaaccagaaatctgatacactatca
ttacatttatttgcagtattatcaataaatgaattgagtaaagggtgtaaaggttccacgccaa
ataaaggtataggcacaaactttgcctcatccgtgcgaggtaaaaatggcctgtcttcgttgta
tgattttgaaaaggttgttgcaagaacacaatttcttgcatttttttaatgcctcagctgatata
cgtagacgtatactgtttaacttttgctctgttgtctggttattaaatcctccacacaacgagt
acatggcattatcgatattctccctcgcatgatcctctatgaccaaaccagccttggtcattag
aaaatggtctgggtctttaccgagaggaagaatatctgcattgaattttgaaattatatcattg
acaacacgttcacagtgcacagatctctttatgaatttagaggctagagatatgaaatgtctat
ttaaattggaacctagcctattccaaggaagtgaattttgatgtcttttattatccctctcgtc
agaagggaacaaaaattgaagtaatctggtccaattaaccgcagctgcagtttctctctggacg
aatgtttgacgttgttttagtagctgaagagacacttgggttggcttgtcactactagctggaa
gagggtgttgaaagccacagacgccaaatctgaacgtcatctttagattggtgtgtagagattt
tgtgtattcctcttcgtggatttttcttctcctttcattgggcacaaactcctttgtctttaat
tcttccaagaagtcttgtacattgtcctcattcgcgaacgttaccttcccatcttgccctcgtt
tcatcacgtcgttgttcttaaacaggcgcgcagaaagctgcatcagggacatttcttccaactt
tttagggtcttcatggatgtccttcttcctcttccccgaagagataaaagaccctataacattc
tcaatgcaatgaactgcatactgtctcaattttctgtttgtcatccctgaaccaaagcctatta
ctggaacaaccatgccgcacttgtggttgtatgtgagcccacaagcaactgtcaatttcttcag
aaggatctcgtatttttcacttccattaacatcgttcgtaaaactctgtttatacttgtgagca
acccagttcactataaataacaaggcataagaagcagacacctttcgtcccaaaagtttagaaa
tttgaagggagagccagtctgccatgtagccatctgattcgtgttcggcgataggtgtcctcat
cattgtgacggatgctttctccactctatctgctacaaatttcactgctttaacaatactttttt
ctgcacgcatttttgtcattagagtttattcttgcatattggagttcacaggggtaatattctt
ccccgtcattatcttcattggaggatgaatttttagttttgttgtcgttctttatatactgttt
caattttgagaagggagtatccagtacgttttctctacatgcactagccgcagaaagagtgaaa
ccgagcaggtaaagttgtacacatgaaaaggcacctccaaaaatattaccaatgaaataagcaa
atattaatcccctgatgtggttgctagggaatctaaagttgtgctccagactataattcgtttc
aacgttaaagtacctttccaatctacgaggcactttaatgtttatgtctggaaggatttcaaaa
ttctcactaaacatacccgacaacttgtttgattttcccaatacaatatctgaccttatgtatt
tttcataaaagtcaaaatccgtcatcgttaaatcagttgggggctttttttgcatttttaagagt
atccgaaagatgcaccaaaccagttctgtagttgtagtttgccattgcttcatcgggcaataat
ttgctcacttcattgtatgcatcgacaactttttctggagcaatttttttcagcatataaacgtt
gccatattttcttatcaggatctagtagttcaacgtgtctagattctattgtcttcacatcgac
aagaaagtatccttcacgtatactggtgcaggccaatctctccatcttcttgttgtagtagtct
atcctcttcctgaagcaagatattatggaagcaactccatccaacatgtggtacagggctgatt
gggtacaccactcgtcactatttactccatcacgagcagtgaagaaatcttgaaggaaactgta
tatagagatgaacgaaaacagtcttctggatgatgttttcagtctcatgtcagttctctgcata
agtttctcgtgccgtataactctgtctacgaattcccatgtcaggttttcatttacgtattctt
ttaggagtgccttttctgcttctatagttgtagtagaagttgaatccataatgtaggaatgagg
gaggtactttggcacgcacgtgtgcactgaccgagacacaacggtgtactgaacaagggttaaa
gacgttggctgttttacacctctcagaggaatccctctaccagaataagtcctttcattttttt
aattaacacacgcaaaactgacgctgaaatcgtttcgcaataatgggagaggaagtgggaggag

aactttcgcctgtggtcgtagggatactttcggtggtgttaaccgccataattatctctactat
atttgccatctactacttttatttccgtccaaaggaagggttaaagaaatcgcgagtcagtaaa
gaccccttggctatacgcgtagatgctaaccatgtcctctccaataattctccctacatgatgt
tcaatacaaacattaaggctagaaatacactaacagaatttgatcccaaaaatatgaatgaaga
ggaacgagcagaattttcaagatccattattttactgggttccctaaagatgtggaaaaggaa
caggtgcagaagaagaaggaggaagaagaagaagacgaagaggaacagacgacagatactaacc
ccaaaaataagaaagtggaatatgtaatgctcgaatctctgagaggggataaggctggattttt
gatagatgagaataagaccatgtctatacaaggttctcctggtacaaaaactgtctgtaagaaa
acgaccctaccaaagtttgtcccaaccccctaaaacggaaaagtatgttccttcagatgaaagat
ttttcaggatggaagaaggaacaggagaaaaggcagaacattattccttcacgtctgttaagac
aggaagaaccttaatcatgggctcttgtggctaccctgtgatgaaggatataagcaaaggtaca
agccaacagtacatgtacacatttgaaccaataaaggaaaaaaagtaatgtctttgtttatttc
cccctttttagaaaaattggaacaacaacaatgtcgttggctgtgacagaagattacgggcac
aatgaaaagttgatcaaacggttacaaacctctgtatatcacacccctctattaggtgcagacc
atgtaatgaaatccatatcagactacataatttctcgtcgattcatgaactacacaaatttatt
aaaacaagttgaatatgttttcgatgaagaaacaggagcagttatagctaatatctgtctgtta
aaaatcctagaaagatgcgcacagaaaggaggaatatatgatgcaccagaagatgttgcattct
tcaattctaagatgggggaagtaacgcgcctatttactattataggaggtaggcccaatatgac
ggtgcgggttaattttaaacatgggcagacaaataatcctgcctatggttatctcacagatgat
aatgatactactactgttactcctcctgttactcctcctccatctccagctgcaagaagatccc
ctttttcacacgcactctcatatccgagtcgtcttcagttgaccattatgtattgatgcatga
taacccaaaaagatcttcatttaaggtgtatgatattcacgcagaaacctttccccataaagct
ccttctgttcctaccttccccccctaaaacctcgtttgaaatttctgacgtgactctcgattgtt
caatggagatttttcacgagacagggatgttttagacaatgttcacgactatattgctaacga
ccccgtaccattttagtggatgttgtgcaccgtggatctagtctccgttgagtaagtacttta
gttgcagccggtctcattagacctgaaaaataccctttcatttgttagaaaagtgtgcttagatc
aaaatggcatccacttcttcatcaactaagaaaagagtacatgaagaagatgaaaatctcatcc
cacaacccaaaaagaaaaaatcaaagaaagtactaccatttcctgttgacaagtatagagctgt
ggataaaaaggtggtaaatctcatacacaagatattagatcaagaaaaggaccacctttctagt
accgaactgcaaatgataactgaatgtaatggtgcgcgagaagatctgcttaaacatcttctag
acgaaggagaatttaaccctactataattgaagtagtatcatccatgcctattgaaacaatata
cgaaatactctcttcttctgctgacgacaagaagtttgtacagatatcattatcaatgttgatc
cacatacttttcttcgctgataagggtactatgtgggtatccaacgcgtgcgttcaaaatgttt
tggggaacgactataaagtggaatttgaaaatatacgtaaaaagtatctgatattggaagactt
actgaacggcgtttcaaatcattggtctgaacatggtcctctttctcacatgctccattcttca
atccctattgtacaagacatgttattgaacaggctggtgcgttactttagcacgtatgatggag
atgctcaattcgatatatcattcataattaatagtgtcttgtggggaattgataaaagtgttct
caacgaattgacacaattgatatcgaggggtgttttcattgtgtcgtacgtaccgatgcgtgta
cgaacaccttcaaaggacagtaatcggccacaaaatactccttcacaaaatatgtcagcactag
gtatgaaactcaatacattttcatccagaatctcagtgtacagaaacaatacctttaaaaaact
aaccgagttagtgcataactttgattacggttccaaagatgcatcatcatcatctcctcctcct
ccttcattatcggacagcgtcaacacttttgtgaggttgtacaccaactatgacatattcttaa
aggtgatttccgactggaaaatgccttatgggttctttaagaaaacttttgacgtcctttattc
taaggggttgatgacattatcagtgtctgaatatacactcaaaaaagagttggttacgtttttg

cgcgccttgaaggaaagggaaattttaatctataaaatggagaagagagacattatatgtatac
tgaaaaagtctttgttcggatttaatttcaggtgtttaaaacaattactccctctcttcaaaca
ctttttaaaaattgaagaggttaaacatatagcacgttttgtctttagagattacagtctcatg
tgcaaaactcaaaaagatttgcagagtttccctgccatacagtctgcttcacttttcatggaag
aattcccttggcttgcaaaaacttggatcgacgacgatgatgatgaaggaggaaagggacatac
cctattaacatttgctatagtgcacagatatcccttaataagccaacttatttcacacccaatt
ttaaaatcgttagtgaatactacatgtagagacaagcactttactcccctcatgcacctcgcca
acacgtctataatgtaccaatgcaatacactcttgtgccttataataaatggagctaaaccaga
gttcataaacaagttcaacgagaatgttttgcatatagcgattgaaaatgttaactatggagtc
atcactgaattgagaggaacattatccagcgaacaaattgaaaaaatggtcaatgtaagaagaa
tgatggataatacaacacctttaatgatcgccttggcgagggagaatattgtactcgctcagct
ttttgacggtctttacaagcccaaaataaaggtccgtttcggttcttcaaagaggctaaggata
ccagagtttgtcctcttaaagggcctaaaggaatcagttgcatatttggaaacgaggaatatat
cctacgatattaacatcataaaggatgcagtaatggacaacagtctttttgaagaggagtacga
aatagcagcagcaggactgcgaggcaataactgcgaccctgaagcagacgagaagactatgaac
acgtggaactttttcaccaaaaattcaaccaaatgggcaagctctattttccaaaagaataggc
agaaatttgtaaagattgtggatggtatgaataggacatatgaagactctgaatgtgcaatatg
cttggatagtctggacggggatcttccttcagggagaacaacgtgcggtcattgcttccacaac
gtctgttggttatccttgataaggatgagcgggccgaataatggcagccgcgcaagaggaggag
gaataaaatgcccgtcctgcagacaagtcacctgcctcggaaaaagactaggggttgccgacta
tgatattgaaacagaggaagaacgtgacacgaaaaatgtcgtgccttcggtagaagaaggaaga
agggaatggaggaagattggtgttgacagatatgaatttcttgtaggtggagtgtggacaaatg
aaataaaactataaaatgaactagaatattggtattatttttacaccttacacatgttccagaa
acgtctgttccagaaatgtgttttgagatttctggacaagccatttctggagtaatctgacatt
gggtcgacccagcggtccaccctcggaacttgacattgggtcgaccgagcggtccaccctctaa
actcgagcgacccagaaaaatttttaaaaaatttttgagatggagaatgagtaaaatctcttcc
ctgaaagggaggtctgaagaggctatgacggttctaggtgcccgccctctccagaaacgtctgt
tccagaaatgtgttttgagatttctggacactccatttctggagtaatctgacattgggtcgac
ccagcggtccaccctcggaacttgacatcaggccgacccagcggtccaccctctaaactcgagt
gacccagaaaaatttttaaaaatttttttgagatggagaatgagtaaaatctcttccctgaaagg
gagatctgaagaggctataccggttctaggtgcccaccctctccagaaacgtctgttccagaaa
cttactaaagttaaagtacgtttctggacaagccatttctggagtaatctgacattgggtcgac
ccagcggtccaccctcggaacttgacatcaggccgaccgagcggtccaccctctaaactcgagc
gacacagaaaaatttttaaaaaatttttgagatgtagaacgagtaaaacactctagtggattag
ggtgttaacagcctaccctttctggtcgcagctagacccagaaacatctgttccagaaacttaa
taaagttaaagtacgtttctggacaagccatttctggagtaatctgacattgggtcgacccagc
ggtccaccctcggaacttgacatcaggccgacccagaggtccaccctctaaactcgagcgacac
agaaaaatttttaaaaaatttttgagatgtagaacgagtaaaactctctagtggattagggtgt
taacagcctaccctttctgggcgcagctatacccagaaacatctgttccagaaacttactaaag
ttaaagtacatttctggagatgtgcgcacgtatgcatgcatgcatacgtacctatctgtatttt
tttaaaagtaggggaaaatgctatttaataaaacaaccaccattctccgctcactatacagctg
accttcgttgagtaaccatgtctagcggaaaagtaacctacgaaatcgttgaaggggggattgtt
gaacaacaagtaccttctagatggaggtgcagcaatctgtctgcagtctaattgtgttgcaaga
aaacgtcacgccggttccctccacgataacctcttcaagatgctaggatttggcgacccctata

aacagagacggggaaaaacaaacagcaaaaatctggccataattgaagatagacctcaactcgg
gtcagtatcagttgtccaacacccgacagaaccagaaaggttttgctccatgacattcttattt
gctcagtacaatatgggtaatggaagaaaatgttacttccctaacgacaaagagtatgttgaga
gctgcaagaagcacgaaagggtccacaaatcttccacagaaatgaaaagattgcgcttgtatta
ctttaacaagtgtcttcacgcgatcgccaaatcacctgcaatgaagaagtacaacaagataatc
ttccctgccagaattgggtgcgcggcagctggaggagattgggagaagtaccatgcttctattc
gagatttctccacaatcattgataaggaagtgataatagtgtctcaaaggatgtaattaaaaaa
taaaaccgtcgctggcaaccccccgccaccaccaccatcgaggcgggtataaataaggggcgctg
gcacatgggtggcacactcgcatcatgtcttccaaccgattcagtcagctgaggggcaacgagg
agatggttggggactattcaagatggacaactgtcaagaacaggaggaacagacagcaacagta
ttcccatagtttccgtccccaacaacaacaacaacatcaaaaaagaacatcaaccaattctcct
cctgctccacctcctccattccccatcattagttggggagccctcggcagctactcaatgtatc
gactggatgaccagtgcagaaattgcgatgaaactggctattacaatttccactcttatgatag
aaagagggaaagagttcgctcattaaacaacactccaagtgaaggcatgtggcggcgcacaagt
agatcttccccttccttaataagaagaaggacgttgacgaagctccacctcctcaatcaaacc
aacacatgtacccccctcaacaagtacagtttccgtgaatatactccttcatcaaagcttgtgaa
ttggcgagacccttcacaagaaaaacaggacaagatcttacaagaggaagaagctcgcgcccct
acacccactccccaagaaaaggaaccagaagtagaaactaaagatgatgttgtcatcgaggaag
aaactgcaccagaaccagaaccagaaccagccccagttccagacccagatattcccgcaataac
tgcaactactactactactacagttgcaacacgtcacgacgattcttctacagtatttctcaga
aatgttattctgagtatcgtgttttggtttctgggtgtttattctgcattatttgcaaaatgta
ttagatctaagaaggaataaataaaatggtatatgaaatttaaatctttattgtttctttccaa
ttactccattgaaattgtcctccttgtgagtgcgccttctggcaacactcccatgtccacaagt
gctaaaggttgtatagttccatccctctttggtgggaatggtcctctatctgattgacaaacaa
aggggaatctatccttcaaatcaccctctgaaacagaaacagtttttctcttgtataataata
tctgctcattaatccttcatatccaaaatggtgatggtgtgcaaatttccttagattaggtccc
cctacgcttctaaagtagagggttgaaagatcatcaaatggttctgggcctttaactcttaccc
cctccaatgttgtttttgttccactaagatctgaaggtctcatgaaatctgcataggttatttc
cacacctcttgtacaagcactctgagcagctgtaggtgcgcaagattcgaatcctccatggtag
ggtgtcaatacaggaagtacatggactcttctcatgactctagggactcttttactcgcttcat
agaaaccgaccatgtctttttgtcctccctctacatactttgatgatgatgatgttctaacgcg
gagattttctgtagcctttccttgcttattgattgtgaaaacttccgcatcttcttccaaatcc
acataatctgaaatagataatctcctcttctttgtactaccggcagtgcttcctcctaacattt
ccatattgctgttaactacttccaccacaacttcactcccatcagttggttcaatgcttgcgcc
ttcatttattgtcgcagaatcggccgatattgttggcgaatctatgtctgtaaagcaaggcgtt
gcatacaggctcactatatccttgacaccctcaacgtaggggaaaatgttcccacgttaaatg
tagaggcagtaactccatgagaagactcgaaaaagtccactatccttctagcctttgcaccctc
ttgacccgtttttgctgcatcccttataatgtggaaaatttcagcctccgcaggaggcacgccc
ggcaagcgttcataatcctccattaaaatgtcattagatacaatatctgtcataccaacatccc
ctaacaattctgcgttcctatcgaacgtacccttcctttcgtccaaaacagcagcaggtacctt
ttcttgtcttgtggaataaggagtgtaagccgtactcctacgcatggcacaaaatttattaggg
gccaacataacattggaagatatatcccatatattttctgttgcagcttttaggtttatctttc
ttccattagttttagtttctccgatagtatttagtattctagcgtctccggattgagctctcag
gagtgctgcagagagatgaacatcgtttttagggagggtagtcatgaggggaaaacgtgaatgc

ctatttcctctgctaggtgttgccgctatagggtcagttttgttcctaccccctccattcactt
tgggacagaataaaccgttggtgcaattacagttctccttttctcccaattccccgattttatc
gaatatgtttttactattaggatcttgcatctcaaaaatactcttttctccaatattggcagga
agtagccgtgtttgttcctctacataactctttatagaaaatttagataacgaagaatgtgatt
gcgggcccaggggtttggggaccatcattgtagtgtactgagagagggcacccgagcacgggta
tccgaaagattggccagtttcttccctgacacaaggcggggagtacacgccgttcaagtagtgg
ccggccgtgtgggggctcagaccccaatctgctctgaaccgattagggatcaattctggcctaa
taaacgtttcagaggaaagagtcctaaccctgagtagcgactcaaagagagccataggaggagg
gatgaaattgagggctccttcagtttccttgacttcacattgttgccataattcatccccttca
tggaattttagagggactattccatgcatggacccgaccgtgccgttcgagactgattcttgga
cactcttttgaatgagaaaactggaaggtatgggtaaaggaaatccccctaaaactcccaaaaa
tgatgtagcttgagcagccgcagacactcccgcaggaggagcgagaggcttagaaaagggtcgt
gctggagcgtacgaattgaaggaggttttaatgtctgttgtcgaatttagaatagctctaatgt
ctccctttgttactcgatcataagtgttgttgatggctgtcaagttgtcggccgaaccagggtc
tcctctcgcgttgaccggcgatctgcgttcgaaccaggtgtgttcggggagagaagatgcagga
atttccataggtcttacaagaggtgaagatgcaccattcattaaaactccatctccagtgagga
ttgctcctgcaaaaatatcggctttttgataacgtttttgcgaccctttcggcctcgcgcat
acccgtaccttctattccattatttagtcctcttcccgagaaaaatgtcccttcattagaagct
atgcgccacgcagttggagggtgagcaaacatttcatatgcttctttttcccccactttcttaa
acactgcagatgaaccagccttgtaatgatcaattattttgggagaatttgtagacaatatttt
ctcactgaatcttgcaaaggtggaactttttcttagcaccctgcgctgtaacaggttgcagaata
atacctccaatttctttgaatttggcattgtttggttcattgtatattgatcttgtattgatgg
gcaggccagacctctcttttccttcgccgttcaagttgggggaaaaaatagtccagaagtaaatt
tgtaacgtcgtcaaaactattactagaatttgtaaccatcatagaatttaatgtactgttcgca
aaagattggtctctttcttgtgccgtcaaaagagagtgcttgaaaattgtctcgatgggagtgt
gggccattcgcttagcggtaggtctatagtgtaccaagggcaatatcttatttactgttttatc
atttttagcagaaggtttttcctttattactctatggagtgaaggaacgacgtatgctgttgcg
catatgccagagtcgtgagattttctcagtttctgtaaggggttatctttgtacgaatcgtacc
ctgccttgtcagaatctttaaggaaggatgtttctatggtcgtatcgttgtgcgcatcccttct
gtcagtctttactcctgaaggatcgcctgctctcatgtgaaccgtctcggggaagattttaccc
tgggacgctattatgcccacagcatgatctatgctatcgattcctaaaccgggaatattgctat
ggggtaaaggcacgaccctgagcgatgcgtttggatgttcagtaaaatttccccctccagctcc
aaccagcccattatcgtttagtgcatctccgttccagatgtgagcggccacacttcctgttcca
ctgtagggcactacacctcctacagattcactgactgtgagttttccttgttcgtcagaatctt
caattcttgagtgccaattgccggcggcgtccatggcagattcgcgcaataatcctcccgtaat
cagatggcctccgttaaacgctgtaatttcaggtgctcttccaaaaagagaagcggcataagaa
tagccgagagccgacatcctcgtatcgtttcctggtctagggaggcacgcgtccttagtactac
cacctaaagtttcgtacctttctgttatttgcagcaataattttgcaccctctacaaagggttg
agcataatctgtttttgccgcatcgggaagatctataccggccacttcttccagtctgatttgt
gtactttcatcacaaagacagccctttgttccagtatctcctacaaccccatacctgaacatga
cagggcagcctagaccttgttccttttcctgtataccaccttctttcttctcacattttggggc
gttggtggcagggtctctctgctgggagtagccaggttcgcacaaacaatccatagtagaagtc
atactgcttatgtcgaaatcgatatcgtccacatcatcaataattgagaatgttgtagggtgta
taggtcgccccactccttttcctccacacagtacaatatctgtgcacccaagaatgggctctaa

tcttggatctttattgagaagggaaggatcctttcgcaccacagacgaatctacacaccctctt
tttttggtgatataagtcgttcccttttttcctcctccagttatgactcctccagattcgtagt
gcaaataagcctcctcgacagtagtaaaagggttgacgtttctgtaggctgcatctgtagcaac
atcatcgtcgtgcttgtcgaacgcagaagaggctgaaaaaatagtatcggcgagagggacacaa
actccgtcatattcatcatcatcttctataccgtattcagttttcaattctctactggatttga
cgcaagttcctcccccgagagaacgacagctaatgcattcaaaatcgttgcctcctacgcgaca
tttttaggagttttaaaacagtcaaatgtgttataatgggaactaatttcccctggtaataga
gattctacaaacctttccctatgatgtctgacttgactccagccataccgaagggggcgtccg
tatcgacatataaggggtcgaaagggttgggaataattccccccatttttcttttaatcctttgac
gcgtaaagagtttcgagacatggctcctgtatctacaaaagagctcgcttaaccacagttgcac
tcacccgggtacaataagacaaaaattataaattgggaagaccgatacagtctttcatgacaaa
ataccccgagaacaaaagattgttgtctaggaacaaagaaacattaaaaatggttgcccgaagc
tccaagaccaaatcccgccgtggaagcaagaagaggtccaccactgctggacgcatctccaagc
ggaggagcccatcaatgaagaagcgtgcaggaaagaagagctccactgtccgtcgccgttcctc
aaagagcggaaagaagtctggagcccgcaagtcaaggcgttaattcttccctgtacaacaacta
tgttatttaattgatttttttttcttctgaataattggaaataataaaacatccattgaaactta
tgcagtatttttattcaatttttttaaccaactataaatccacatgtggtataaaagtttaaggg
tacagatatattataagatgatgtaacagatgcacggtcaataacaacagggcctgaagaagtg
tctatgaatttcttgtagaattcttccctgtctgtacttgacgctacattggcaaataccagag
ggtctactagtacacctgagctagtgccgggttctattttagacatgacattttgaggatttt
aacacataatacaatatcatcatggtcgaatgtcgtcctgttccagagacggcgatgtagtgct
gaatttctatccactaaaactagaccatcgtcatttttcaaatgcttctttccttcatttctag
ctacaattttcatgtagaatcttactctttctgcatacgacaccaccttgtcttgattcccaac
tatagcaaatagtaacgaggaagtaaattcattgtttgcaccattattattgtagaacccgttt
gcactaccaattcctccacatctagggtctagacgtaccagaaattgtccatcggctaaccgag
taacagggtcgccgctaggccatgtggctagccgagtctgtagaggggcaactggttgtctcaa
ttgagaataagaacccactgcgcgtaggcggccagtgtttctccagttcgtgtatctgtctgca
ggttcgtcagggtatttatttgctccaaaaacaacaataccccatttcctaaaatagccgcaa
gtttcttgtcttctttatttaccagaagattttccttttttggttccagactcgtctaccacgta
tgtagtatttgaagtgtgcacaatttggtctaatgtttcttcagaaacgccgtctcccatgtac
attttagcgtacactttctccccctgtgtatacgcttcactaggtgctggcaataactgtcgat
gacgttcttgttccttatcgatttgttccctcaaccagttttctattgaaaattcagtttgagt
atctgtattgacaatgtttgtagtatcattcgttcttatggaagaaatgaaaatgtgccgaggg
tactttatttctcccccgtaatcagcgaatgcccattcaggtatgcttgtaggtaggggacaa
gactagtctgtttcatgaaagggtcccttatccctctattagaaatgggaggaagtgtagtgcg
acagtagactatgcccttgttggttatgtcggtggcgggtgtgtcccttacaggtgccgtaaag
gttgagggatcattccatatagccaattcgttagatggtacagacatgtctgaaaactgtcctt
caaaatgcccgtccatcgtaaagggcatctgtatgcctttatcgtcacctccaaatacttccat
ctttacactgaaaccctatcgtctcgtgcaccgtcaataaatatagggcggctggttattctc
gaagaaagaacgttttcttttgcaagtatagaaccagacacgggatcggagactgtgatgtggt
agcttttatcttcaaggacaacatcatcatcatcatcattgacgataacatcagatacggacaa
gacagtagttttgttccttgacgtgatactgtgaatgggttggaatgggttatcactcgctttc
actaggccgttatgcgaatctaaaacagggaaaacagcgtacagtttagtaggttctgcgggtg
ccttaaatgtaggctgttcacctcccagtaatgcagtttgaacgggggtaataatatctccacc

tcgtacttcagtcaatggaccaactgaaacgtttaaagcacgaggcagtagagatgttgtgcgt
tcaccggcatattctgtggcgggggaagaaagcacagccgttattcgaggttctctattatcat
tatcagttttcaagatgatacatggtcttagttggtaggagaagaaatctggcccgttcgtggg
tccaggccaatctggtgcagttgcagcatctactctaaagtttacgctagaaccgaagcacccg
actctcgaaggtgtattatgtacaacaactttgttcgtgttagcattcacatcagtacttataa
ccgagaacaatttatcagattgagtaacttttgatgctggaaatacaatattgtacccagaaga
tgtggttaaaacgtcagacgacgttttgaagcccacaatccagcccaacattttccattgcgct
tctatttcactttcaggtacagacacacatataaaggtccgagctgggttagatggatttctaa
ggtgggttatctctgtggcgtttagaatcttgtaaccctcggattcatttccctccatattctt
taacgtttcattgtatagtactttgtttaatactggtgtgttaacagtgtttgttttcgagtac
acgaacgacactgaaggtttgttaatatctgaaagttgattttcgcgaggtagtttagctgcta
aattggacgctatttcagcttgtcgggaaagacggggaaaattagcagggtaatctgaaacata
tttttcattcaatgtatcaggagtgtattccacattatcatagtttattgctttaggcatatca
agcctcatgagggctacatttctacaagcaaaaacttccgttatttttggtatgggtgaagata
ttgttacctgagaatcgaataacacggtcttgtttttatcctctgttacaatggaaattttcag
tctattcccagtgttgaaaatacttccctttgttgatagtgttataccgtcagctagagaattt
acttccacgtgtttgaatacggcttgttgtttattgccattgttgcgagagaacgcattcgggt
cattggtgataggaggccaacctgctgggacaaattcagggaaagtttcgaatagagacggacc
actatcgctgctgccacccccgtcttctccttcatatttttcatccactaaatcaacttttatc
tctgccttaaagggaagagggacattatctggtaatattccgtcaagaatatgtatcctcacga
tggtatcttttccacctttttctaataattcttctgtacggatattaatcattcgaattggcag
caatgatgatgatgatgaaccatctctatcaggagccgattgaggagaaatatcatcaccaata
tcataaccgatctgccgtgcagtgcgtttcattatactgtgtaacaattttggatcctgcacat
cagtgacacgttttaattcctcagaggatatcctttcaatatttttagcgagtttttttgacgc
cggttctaacccattcttcaagttcctttctttttcgggtcttttctaacagcagcggaatca
tcgatagccgctgctatattcccgaatacagctttttctatttcgcaggttttcttatgtgta
tcacaccagatctttcatcgggcgtaaaattgttctggacaattttcataccaatataggaaat
aatagaaataaacacaacgagaagcgtgaattcaaaggcaccccaccacgagccaaaaataccc
gtgactgctgtttctccaaacattgttattgctgttcttaacggcagggttggttgttcggcaa
atcaaggaaacaaaaaagttacccgggcatggcagggaatagaacccagttcgtatcgtccctg
attgctaaatgtatatcagacgtggaacaaggcatggagtgttgtggcagacaagcacaggatg
cattaatgactcgcctagccaacttaaaattgggcgattctcttaaagaaactgatgttaattt
ggaatacttgagatacgcgtctacgcccctccttggggaattaaactacgacaaacaacaatat
gcggcaacagttgacatcaacctaatggctcatttctcctacgctgctttgggtatagaaagta
tactgaattctatacggagagttgtagtggctaatcatcaacgtagaaataatggaaaaaaacc
ttctgaaccaatctcacgccctcacccgctgggaggggtagaacctcctctatcgtcagagttg
gcaaatgcaataagggacaagttcatcagcatgggggcgttggacagattgaattcagcaatag
tgacagcggccttgggggctattgccagtgaacgtgaactattcttacgtgaaaatgctgtaaa
ctacatgtacgatgtagaatttgcagaaagagatgctgctactacagatacagggaatgtagtc
tatctttccaccaaaatggacgaagatgaagatgacataataaagcgttcagaaatattagata
aggtatcaaaacgacccgcaaaggaaggtatagactggcgccccacccctgacaattcgttccc
ttaccaattgatttggggcgatgattctgtagatgatactgttcttatagatctcatcaccaat
gcgatcgtgcctaatatttttatggcaaaatttatcctgttcatatgtaaccatttaagggcag
ttattaggagtatgagggaaattttatacggtaacatttcttcttcatccgataattattttga

ggatggacgtaaatggtgcttctggttgaacctgtacaatagactggaatggttcatgttagta
gttagatttgtaattttcctccactcaaaaaaggagtccttttcaggagctgacaatgttaacg
tgaaaagacttctggtggtagttgtggagagttttcctcccgttctcttggacactgaatgggt
caagactaatataacgtcatggcctgttattaataacagcaataataatagtacactccctgtg
acagaagacaccttaatgagactagcgataaggacgagtagcggtgcccgacatcctattttcg
acgaaattaactccttgacaacagcagtgaccaaccgtattaccttccagtctgcagaattctg
cacaaagattttgctcgggcgagctctggacgaagaagaagctggaacaaaaatgctagtaaaa
tcagtcaaagagacgggagaagaaaaggataagaacaatacgttctcttcatttggtttattac
tgaagaacacaaaaaatgaagaattggaaataaacataggcgataacgatgatgagactacaga
tgtggcttgttgggcacgtacttcctcgacatcctttatccgtaataggacatatgcgtttaaa
aaaatatggggccttgaggatgcaagtgatgtagtcgagctgaagcgagagagtgacgccatta
catcctttgtcaccgataagagcagtcctctcctatttccgtatgtgtccgactggagttgctt
actattacatccctgttgtaaagcaccggccataattaaaagtgtgtggttacaaatcctgaaa
gatttttcccaggaaaatataaaaactataaatgaaaaggtacaatctctttcatctgagattt
gtcagaaatcaaacgaccgttttaaaaataaaaaaattgctgccgaacacgttcgcagtgtaaa
aaagttattaaatacgataagcaacagggagcaagaagcagcactatctacagaacactgtatt
tggttaacgattttgtggaaacaagtcgttcagaacactctcaaccttctggagaattttcccg
tataaaagggaaagtactcaacatgttcactcacttgacccgagccttccgcaagatgaacaa
cctagtcaatcgcagcttcattgacgttcaccgagtggtggccgagttatcctatccagaattc
gaagaggatgtaaagaatccggaatcatccatatatagaactccgatatccctcttccaaaaca
aggatattgttacaatagttggtgattacatcctctctccgaagacggactcattccaagttct
atacccaatcaagaaggtcatcgaacacttcccagtaatcttccactgcacccacaataatgcc
cctctctgggtacaccttctggacgaacgccatcatcgcctgctccagagcctgctgacgtacg
agattgtgaatgccaagtacaggggtattgttgtcatcccatactacaggcgccccatcaacta
tcaaactgggaagagtctactgatgagcaaactggcgtccgtaaaagttctggacattttaatg
agatgtggatcatacaaattcatctcattaatgtgcatgatcaacaagaagaacaacaccaact
ttcttcactgctgtgcaagtaaatggggagaagttggaagcaagatgatgctccacattgctga
aatgttctttgccaaccctactactagccaacacctatccgacgctagtagtttccctgatgct
gcagcagaggacgacaaggggaaaacacctgcccatctagcaatccaagaagataatgctgatg
cactcctgttcctgatctccctctacggcgcaccctggtttcaagataacaactcgtacatgaa
atctgcccttgaactcaagtctaacaagtgtgtcaaggtactatcctttgcagctgacaagtac
gagattttacccaacattaacaacaatcaactagaaccagataccatgtgtggagtgtgtgcaa
catctgtggaagaagatgaaaatgaagggaaaacaacaagtctttcctggtaccagatgaattg
caagcattacatccattgcgaatgcctgatgggaatgtgtgctgctgctggcaatgtacaatgc
cccatgtgccgtgaggatgtgggcgacgaagtactggaaagatgccctcctacaatatttagat
ggttaaaactggctgagagatctgaacacaatcgtgtactttttgaagcaaaaaagcaagaatt
ctataagcagatggaagcaatgaaacctcccagagttgttgttcctcctcgcaggacatttctc
accccagccagaagaggcgaacgagccatcagaatcgcaagagaaattgccaccaacgccatcg
ctgaagccacagctcaaggagatgtcaactcctacttccctgttctcattgacggagcggaga
agaatatgaagaagagggagaagaattcttcaattctgaagaggaggcgcttgcttttggaaga
ccatttctggaagatgaggaagaagccagacaaatacagatgcgccagtttgctgaactgtcta
gacgaggcgtttctgtcaatattattaacaatgataatcctcatcgacacatctctacagtaaa
tattgtgcaaccagtttatggagttgaaaagtcacctgctgcttccttcatctacaacatgctc
aagaatgacgtctttgagtctatacgctcaagagatactcgagttggaggagaaagagtgcccg

tcatgaacctgtccaatgacaagagggcattattccacgcagcttcttccatgctttgtgactt
tgccacagaaacaaactctcaaattgttggattggactttcaagcagtgtatgatccccatcac
atatccaactatatcgagacgtttggtagtcctcttcacgcctacccaggagccgtcacttttc
tggacggggcccaggactattatgcagagagtatcagatacgacaatgatattgtctcattctc
agaaatggcaagtgagttgcacatcaccgaagcattagatgtctttgagggtagtttattatcc
ccactgttcaagaaaatcaggactggaaaatcttactctaactggaacgaccatttgaggcgta
gaaattatgctcgagatattgctgaggaatttgttagggtatgtgaaaactctctagcttcacg
cgaacacccccctgttcatgtacatccctttagagatggagcaatccccattctcattgaatat
atagtagatttcatccaccactgcatcacctggtctatgcaagttaatgcactccattgtatga
gaaagtacattgaacacgagaatacaaatgtgcacctgttaaacttgcgtcctactgatgaaag
ggtggaagttttaagggtatctcaactcagatggagccgcttgttcaatgaacaatacaacact
agaatgtccctcagcaccaaaagattgagcctcatgaagatcttcaaccatgatttgggtgtgt
ctaaatttggtgtatacaaactcctagatattattgaaatgtactgttttactttaatctaaac
aataaaaataatgtaaagaattccatgtttttcatccctcctcctctgtggtgtgtatatataa
ggcgggtagcctcctccacaggcacacttgtccagtagccgttgtcgagtgaacatcatggcag
cagacctcctagagttggctatccaggaaacaatccagtctgaattggaagaaattgccgatac
tgaattcctcaattatcttccccataaaactggcatctgcgaagaagctgcagctaatggacgg
ccatatcttcctacactagaaatgaggaacgaagttgaccatttctggtcccaagataacagga
agctgaaactcttggggcatttttgtggcaacttgtatgtggaggcatttatagctggttctat
agatgctgaaacgtgcgtgggctttttgaggtcgcaagcaactggactaggataccctctattg
aaaaaactagccctgattgcccgtgaggataaatcaaatacaactaattacaacttgtacattg
atagaaattcaatgatgaaacaagtttttagtgctgaaattgataagcgtccttcatcaataca
gaacacttctcacacaaaatcttcccccgtgtacttgaaactgatcgacagaagaaccgagtgt
cttgccctggattggctggacgcatccaagaggacggccaaggaaattggagcagccagaaagg
tttgtttcctccagaacctgattgttgccatattaatacctgcatacacagaaacgtttgttct
tgatacaggcaacgaactagaacagcaagtattggatgatgcatattttaatgcggagaataaa
gataaagtggatgaaatgtgtgtagtggccatattgagtactttgcacaatttatttgttagga
aaagtcttccccatcatttgtacaatgcacctttccgtcttcctccctttggacaacaccctat
catcaacattgaaaattcctcattctttaatgaagatacgacacctattctagcgtcaatttct
ataccatcaagtatggtaataaaacaccatacgcgaaaaaatagtagatggagatgtcccaata
acttgatgactgcagcagaacggtctatatttttacgtggagttttaacagtcagtggagatta
cggatggttttctgtaattgtaggatctactataatgcctagtgtacttttctatggcgaccga
aaacatctaataaatacagtcaaatctaataacttttctgccataacctgttcttattggaaca
agtatatggattgcagatcttatgggtttgagattatagacacacctgaaaataactgtggttt
tcgtataagggctgcaattgattgctcgaacacagatttccattcaccggtaacgcgagtcaac
aagaagaaaacgagcattattaatgcggtaaagaaccctttttttattagacacacagaaccta
agtggtacaacaaaaatgccatgtgtggtgaagtattggaaaatgttggcgtgaccctcgaaca
acacgtccgtgttagtgatgagtatatggacagatttggtagtctattacttggacgagaaaag
aaatggacgtgtaattatctagatagaataaagtctctagaaactatttctaacaatctcaagg
gtaaaattgacaccatgtgtaaaattctagaaaccaagtataactacaaatcttcttccttata
ctataagcaaataactgctacgtctgatgatcctataaagatgaagattatcgcctctataaac
aagaggaggtatttgtgtaatattctagaatttgctataatttcatcagagaaaaaggatgagg
tggaggaagatcacactaaaaccggcaatggtggttgtgcgttttcaaagtataagaaaaaaca
actcgaacctaaacaacatttaattgttaaagtgaataaatacattgaggctttttcgttaatt

aagatgctgagaaatgattgcgaacgcaacaagtgtaggtttaaagaggctgaaattagagagt
gcgccaacgaactggtacgagaactgtatagggcttcggccagaagttatgtccacgatctggt
attgaagcgaactaatgtccacttgacatggcaacgcccttacgatgaaaacgctaacactatc
atgtctttaatacctaaatgtaagctacacacagtattgtacgataaagattcgcgcgatgtta
agttgttgaatttctgagaacgagggatggaaattataacccaataagacactctatgctgga
gttagtatacggagaggagtacgctaaagatgtcagtactgttacctgttttgaatggttaaaa
tggtgctcgaaaaaaggtgtgattaaatatgaagactttttggatcgttacgagaaaacggggg
aggaagataaagacgaaagggaattctttagactaaaaaaatgtagtagagatcacactaagga
tataaaaaaaatagaaaatgtactaaattctgatacactttattcttattctctcgataaaaat
gtgcaaacccacgcatcttctagtacagttgtaaaaaatgacactgacggaaaaacgtctatgg
tgggctgggattatatttttttcaatcggtaaaggagaaaaaacaaccaaaaaacgaaaactgga
aacgatagatatatcgagtagtgacgacgacgatgaagaagaagaggaagaggaagaagaagag
gaagaagatgaaggaaaaagaatgaaaatgaataactgcagcagcagcatcaagaacaagagca
agaacaagaatgggagaatgtgttgcacagatattctcaatgttgtagaaccttctctacctaa
tactttatcgttcaattgtgtaaaaagtatggatgtgttgaatttgttatgatgttaaaaataa
taaaaatattgtacattttattacatttgtttgttttgacacctgagcggtttgggcctgttga
cgtgcctaacttttttgtcaccctcatgaatacaatttgtaaaggtgctgaaatgtacttgtttt
ttatccaaatttcctgtactgaagaatattgaaagaagacttcttgaagaggaccgataaaaaa
atggccaccttccagactgacgccgatttcttgctggtgggggatgatactagtagatatgaag
aagtgatgaagacttttgatactgttgaggcagtcaggaagagtgatctagatgaccgtgttta
catggtgtgcctaaagcagggatctacttttgtcctcaatggaggcatcgaagaattgcgtctt
ttgactggagattcaacgctggagattcaacccatgattgtgccaacaacagaataaaataaag
acggtgacgggagactaatatctttcttagtttcccgtcacggtgaaaatgttggttatttctt
ccctatgtttaaaaatttgtcttggttaaaaaaataaaacgaaaactgtcaatatattgtttta
ttgatatacaatatcccttttttacacagaaatggcatgtctctttacacgctgttgttcttgag
tgttgtttccatcttcttcttcttctacgtcgctatcaaattcattgtcgttttcccaggtcct
ttttactccccttttgctcctgttcactggtagactgttctccatttctcgcttcttttgttcc
tccctcttctttaccttctcaaaccacttttcggtcttgtttctcgtacgagcttcctttgtgg
tcaaatgtctttccttgttgtacctagtgacaattttttgatgtgttctccatcatggaatggag
cttcctttgttgttcaatttcaaattgggtcatattttcttcatcaagttcttgaggcgaagag
atgtctatcctctgcccatcttgatagacggtgccatcattcataaagattcggatatcagaac
ctgtcacttttttgtacaatacccttcaaatcttcatcacttggaggggtctggaaaactatact
tgatcggttacattttaataccttgaacttgttttggtgtttgatactgtcgttgcaagcgata
ggtgcgtcacagccagtcacggccatgatgagtctgggcctcatacctgcagtatccctttttct
tgtttatgggggagaaaatgtacctcaaaccattttctgttgcagtttcaaaggaaccactttt
gtctgtcctgcacaacaagtagaattctgtagaggatatcgagaagacgagacaagagccgctg
tcgtatctgaaggtcgcattggaagatttcttgtgtacatttctgagagattctttttgttcct
cagagactttgaaaagttgttcagtttcgatatacgagtttttgtactcctctttctttactac
ttgtgtttctagttccttttcaacttttgtggtctttactacttgtgtttctagttccttttca
acttttgtggtcttttcgacctggttttctagttcctcttttatttgtaaatttttcctagcca
actcttcgcgtatctggcgttgcttttcatcactgtataagataacagcgtaggctagtgtcga
aaggtacatttcaatgcttgcagcagaagggtgtgcgctccttgcaaaaacgcccgccaccata
aacaagggaacaatcaagcaatgtgaagaatggctcttgaaaataggattggctatatattctc
ctttgtaatgggtacggtgacaaatttcaaccaatgggaccatcatatccttgaaaccatgttt

ctgcatagttctctccattgactttaaagcgtgcgtgacgttcattgtactgaaacctaaaccg
attaagaaatcggaacttattatcatacaatcgtgtttttttctactgcttcagaaagactat
tgctcatgtacctgtataattgactgaatttaaagtccttgtcttttcgttttaatttttcctt
gactattgtattgattttacgcacgtcttcttcttgcgttgcaagccattcctcgtcagtaacg
tgcccagcatcatctttgaataaatgtccaggttcaggagggcacaacttatccgttcaattc
ttaaagagtcatcttctaccaattcttgtaatctttttaaccctcttgcgaatttgagttgttt
atctttcttcacatgtacaacaaatgtgtcttcggacaattcgctttgtcttatagattcgata
gcttcataaacacccatgacttgctcatatttacttgttttcttcccgatcatcaggaatgacg
atcttagttttaactccttttctgatacactttcattactaacagggttattgtttttctctcc
aatgtattccatatttatcttcttcaaaacaacaacggaaaatacagtttcttttaagaaata
taggttacaatactgtggattgataattttactcacagctttgttcaaaacttaactacgtggt
tcggcccattttgcaccagatctgttttaaaggaactgggtaaaatacacatgaaaacactcct
tttgaacaaattcagatttatatgtatttgtcgatttttattaatattaataaagtatattttt
aaaaattaggatggggacagatggtgtagattgatacagtgaccgtccctgttattttacctta
atttttttccctataatacaaataattggtatcagtttaggaaaacatatttttaaaatgaag
gttatcatatcaactaaacacttttatttacccccttttcacctcagaaatatcctcacataat
cgcatgcaagacttgcgcatttcttccctgccgtacaaatcaatagcaaacgctaacgccaaca
gatacgtctccacttctagagatgggggttgaacattacgggaaaacatgtaccctagatacac
cagtggaataactaggcaatgggaatgataactcttgaaaatggggtttacaacaatatcaccc
ttgtatttttgagatggcacacttcggccacaggcacccatgtatatttcagaccacttttcc
gcatatttccctctatcgctattaatttttccatgacatcatcagcactaaaaccgagaccaat
caaaaaatctgtgctaacaacaaaacgttccttttccccgaaaggtatttgaggaagactttt
gttatatacctgtaagttggactgaaataaaaccctctaaacaacatccgtgtcttttccttta
cgagcttatttacttcctgtacgtctttgtcttgagaagcaagccactcgtcatcagtagttgt
agacgtgtttttaagtataacgtgtcctttctcaggcttaatagaggataataatgattctatc
ttgagggaattgtcccctgtgagctctcttaattctttcagtccccttcaaaagtaatgtgtt
tattttcctttaattgtacaatgaaaacgccgtctctaaattcactctttcggatagtttcaac
tgcttcaaatgtactcattacttcttcatacttttcatattgtttttctccaatcattagaaaa
gaagagcgtagctgtaactcgtcttcttcaggtgtctcatccaggagatttatcagtttttgtt
cccctatatactccatggtttatttagtcagttgatttatttttctctttgacacagtttttat
ggtactctgggattactttactcaaaaatttactcacatgttgttaaaagtttgagtgtattct
tttgtggtaaactaacaatgaagtacatttcttagtaaatgaggtaggtactgattcgtgatgg
ataacgatggcgtcccatgtctagtgatgtgagtcacataataaaaagggaaataaaaataaga
actagtaacatatttttttattttatccttctcaatatgttgtttgatttctttctaaaaatggc
tgctgcaaaaatggacgctatccttgcggatattaatgggaatgatacagatttatccaagcta
atcacagacgtgattcaaaagagagccaaggctgtcatggatagaaatagggctaaaatggaca
tgaatagaagagtagatgaggctattcaggaagccgtagcggccaagaaacaaaaagcattagt
ggtatttgataaactcgtggaagaaactgacagcggacaaagtgtccctccaacattatcggga
tccgattacgacgcgtgggtagacagagccatgccttcgcatattgaacttgtagagagtgttg
agggagattctttgtatgataaactccctcctttaacgtacaagacatagacgaccaaatcgg
tgatgagatagatacaccaatatcttaccttgccatggtagtggtaaaagtcgactgtgaaact
ggggatatcgaagaagagtacaatcttgctcctacctttggtgtgacacaaaataataaaatat
acagagatgaaagagaccagatttttacaaaggctgataaatctgtgcgtatttttaaacttgc
taaattggatagtatatcaggtaaaagtagacaactgacgtatgcggtaaaaaataacaatgaa

tatacagagtttgtctgtagcgtctttgcagagtttgaatctgacagtgacactactaaatcag
gtataggtattcgtgaatatgacaaacctaaaaatgaattcgaatatgaggagcgagagatctt
tacatttttattccaatacaacccgcagggacgaaattattgttatactttttggtggacgtc
aggtcccgtattatataggactataaggcacaacactctgtttgaagagaaaaaatgttcgtca
taagcatagctacgtcattggtgttattcttcttccttcttttcgtctcaataacaattttaga
tggtgcaaaaacaatcgactctcaacctttaggaaaaggaggaagaggaagagatatcgtacc
agtgagagtggtgacggcatagacggaggaactggaacaacaaacggaggaggaggaggtggag
gagaaggaggtggtggtggaacaaatggaaatggaactggaacaacaaacggaggaggaggtgg
aggagaaggaggtggtggtggaacaaatggaaatggttctggaacaacaaacggaggaggaggt
ggaggagaaggaggtggtggtggaacaaatggaggaggaaatggaaatggaggaggaaatggaa
atggaaatggaaatggaggggatacagacacagacgattttgagcctacgccagcccttctaaa
agaacgtctacttaattccatatcttcaaaacctaaagaatactatgaagcattcgtatctgca
gaggtagaaactgccttacaattatctcgagatgattctacccaaactataataattgatgacg
accaattagaattggacgcctcagacactctacaaggaaaaccaagggattatttgttcaagct
cgcaggtgtttcatcggccttttttagaaggtaccactatccgcaaagcggaagaccgtgcccgt
aatataaatgaggaagaaattgcacaaacaatattaagtcaactaagagaaaaacacatcaacg
atgaatacgatggaaaatatgccacacccgaggaaagagctgatttttccaatagtcttaatct
tgttactaaatacacaaaccatgaagtgggtcttttagtaggagaaactattgaaaaggctttc
cctcatgagattgagtttgagagatgcataattctagtagaggattttaatagtggaactatta
cttcaaacactatgcagtacaggtccaacgcttacaaaatcagagtagtagaaggatcaacaac
agatccaggggaagttgtccctgatgattgtttggtttttgccgtagtagtaaataaggaacaa
cattctctagaaatatctgcaactaacagatgccaagacatatgctttgtaattattcctcgtt
tatccgcaataggaaaaaaatgctaccatggtaataaggaaaggtgatgaaattaaacaagaaac
ctatctgtttgtggccaataagaatgacactactcatttttcaatcatcacagacaaggatgaa
tctgttggaatagaattaaacatgctcattttctcagaaagaattctccccactttgagtgacc
ctgcaaccgttccaagacctttgactgacgccaacgtactgtcagcctacggaaagcgcctagg
tgttggtgcctttacagacaaaaatttattgtccagccaataaaaaaaatataaaaaaagattg
attgttttattttcaaaaaaaataaaacaccatgggagataagcaaaaggtggaacagcttta
agggaattaaaggcagaggctaatgatgattggctatcggctaatgttgatccaattgttgaga
gatttgtcacaactaaatctgatgagacagcccaagttgttaaacaggccgttgatgaaaaata
cgatgaattattagaagataaggttgaagaaatgagaccggatataatcaatgaagcatccgaa
acatatgataaacttgctgctgatatgataagagaggtagacactagtagtgttattgctcctg
caatagctggcacagtggcaagaactatcaataatttaagagataaaaggaaagaatatgaaaa
gaggctatggacattagcctacaaaccatggagaagatatgtacaagcaattacagtgatggaa
tttcgtttatcatataaagacctgactgtccatgccaattccgatacatatttaacattccctt
ttttaagaataaaaaagatcgcctatattaataacgaccgcgccagccccgtgaactgctcctt
gtcagtgtcttatcccaacaagtccgagtggggaaatgataacggagttggacgtaaggttgac
atacacatcagaagaaatgatttacaggaaaaggatctttacctgtctgttatttgtatgttag
atactgattttcaggatatgataaagctgtggaagttgatgcccataaatttcattttgaagc
agggaacaggactatgtttttacccaaaacctctaatctatttaatcgatcgcatatagtaaac
tctaaaatatgcactatagtgttccctcccgcttctgccagttctgcttctacaacagaactgg
ataatgtctattacaggatcacatgcacatgctcctagagggggggggtggctgtgtccttgta
taaaaagcgaggttggcacagactatcaaacattcagccgtaaaccatggaggaagaaagtcaa
cgagtacagaggaggatcggagtcatcccagaagaggctgcctcccagatcctgaaagacacca

agttaagagtatcatatctgggcgtgggacattggggatactctgtttcagttataaaaagtgc
tctccagaaaggatgcaggaggaacgatgaggatataactgcgtggtcaatcagggaggcttat
ctctattaccatcttggattgaattacattgaaaacgtaaaacctgctgcaaaatcattaaata
caaatatggtaaacagaattaaaatcatagctgtggaggataccagtcctagaagtatggtggc
ttctaatgagtgtgtgagaactctagaaaaatatgaaaaggggaattttaggcaacccagttac
ttgatggatgctgccatgaggctagttcacgcctccagttctagagtgtgctcccacatgagag
ctttgtgttgcaaggaagaggacagtgataaattaggggtatttattatgctaattttaatga
acttgaaactcagtgtgttagtgcagttaatttttctcccatagaaagaattaaacacgtcttc
agggagattgaaagtgtaaaattggggaagaaaagtgtacagttattaaatttaagaagtgtag
cagcttaccatgtgttaagatattatggggataaagtgaaagatacaaataagaaacatagtgg
accattcaagcgaaaagagtttgagcagttttggggttatgctttaaatttgttactcagcac
gtaaaaacagaccctgaattacgttgttattttaatgagttgacatatgccataaattggagaa
gggatttttctgctcaaaagggttctttagagaagaaagtctattccttacatctattgtgga
attaattatagccatgtgcataggtgaccgtaaacagtttgccaagattcagaaaagggattta
aaacgtttcaataaaggagaagaagggagaaaagaagaggctgctacatttgattggatagaag
gacatgttaaacggatgcctcaaatgcctgtatgggttctggacaaacatacgaacaaaaacac
acatggagtatcttttgccctagagagtagtatggtttctggaggagacaagcgctggtcccca
ggggtgtggcttcattcttatacaaagatgcgtctagattctccccctcccccagaagtgggcc
aatttctggatcaggcttttaatacattgaagcgagaagctgctagtcattgcgtaacgaggaa
tatttgtactactacaggatttataaaggcttctagttttactgctaatattaattctgaaccg
atggaaattaaggaagaaataaagaaaagaaaaattgagattaaggatgataatactactgcta
ctgttactgttagtgctactactagtagttctattacttctactccacctcctacaaagaaaca
aaaaacaactccaagtggaagcaataaagtagactctatacaattgaataatctaccaacttta
aatatggaggatttagatagagtactagaagtacacaaccaaaactctaaaaagggtgtagctg
ctacagttttaatgaaggatggaaataaagttgtgtttaaggagatgagaaaaagctttggttg
gggttctcatcagaattttgttcaagtactaaaggatgaagatgtgtgtaaattggactatttg
ttgccatgccctgatagcggaccatacagaggattatacagatgctatttcaaaatagtaaagg
atgaaatttcgagtacagctgctaggatagaaaaagtgaaatggggagaaaatgccatgtgtta
tttcatctctgggtgtgtgactagacaagaaggaattgggaaaattataacagatgtgcgcctt
tcacatatgggaccaaataaacaatatgtgtatgataactataggcaattaatacacattttaa
tttttagattgttgactggtgtgagtgataccaacacatcaaacattctcgtaggtgatggagg
aaacttgttctctgtggacgagaattatgtgggcgcaaaggatccgaggacagctctcgaaaat
agaaagataaaggaactacaattgctactgaagacttcctttaaggtaaacaaggtaacaaagg
aagatatagatagctgtcttccatcttggctatttgatacatcaaaaagtgataaaataatgaa
tggtgtatgtaatattggtaaaaatatgggaattggtcccactactttggatattgtaaagaat
aattgtacttgtattttaggggtcgtgaatgacctgctttatgacaacaaataaaggaataata
ctttatacaatttttcatttccttcctttttaaagtgtataaaatttttctaagacaaaagtga
gtttgagggttggtcataatctagcacatttcatccctttttccccttcttcttcttcttcttc
ttcttcttatttgaaagaccctggttaggtccctgctaagggtaggctgtaacctaccatatac
actagagaattttactcttcctccatctcaaaaacttttaaaaaatttttctgggtcactcgag
tttagagggtggaccgctgggtcggcctgatgtcaagttcggagggtggaccgctgggtcgggc
caatgtcagattgcaccagaaacgtctgttgctccagaaacgtacattaaatttctactgtttc
tggaacaggcgtttctggacacaccagcacccagaaagggtaggctgtaacctaccatatacac
tagagaattttactcttcctccatctcaaaaacttttaaaaaatttttctgggtcactcgagt

ttagagggtggaccgctggctaggcctgatgtcaagttcgaagggtggaccgctggctcgggcc
aatgtcagattgcaccagaaacgtctgttgctccagaaacgtacattaaatttctactgtttct
ggaacaggcgtttctggacacaccagcacccagaaagggtaggctgtaacctaccatatacact
agagaatttttactcttcctccatctcaaaaacttttaaaaaatttttctgggtcactcgagtt
tagagggtggaccgctggctaggcctgatgtcaagttcggagggtggacctctgggtcgggcca
atgtcagattgcaccagaaacattgcgtactactcccatgtccagaaatatctagatcctttct
ggttctcgcccaccaccagatatctgggaagccttaggcgagtcatgtttcttgcaccatgtat
ttctaggaatttcgctgacgtcaataaactggtttgtcatcctgttttatccagtttgctgacg
tcaatagaccatatttgggcatctcgcccacacagaaacaggatatgatatcatagatttctgg
gaagagggtgtttttgtgccgatctgggtgtataaaagagcgctgcggagaggcagaaacatc
agacagacttgatctgtacagctagcagcagcagtagcagcagccaagagaagatcggacgcaa
accattctcgcagccatggcagcagcagcagtctcaggagaggggagaatctctgcagatctac
tcctgttggaacaactcaccccggacggagacgtgataagatacgactctgagcagtacaccaa
acctaggaagatctttggtgacaaaagtgtgatagagactattggacattttctcatccacaac
cacaaccaaggtgagagttaccaaatcgcatcttctgtgttggaaaaattccccgctctactca
attgcatatggaatggagagtcgggaggaatggctctatggaaggcattgtacagggctaaaaa
gtatagacttctcaactcactttggtccacaagataaaaaattggccttcggttgccgtgatc
cctatctacgggtcagtgtgtgacagagaagaaaggcccatcatcatgagtgagattattgaca
aggaaactcttcagaccatatgtaagagtgatatacgttctcttcttggaatgatgaacgccaa
gcatggcacattgggaggtaatttttacacttctatgcccgctcaactaaaccgtttgaaaat
ttccaatatgaagcaatgggagctaatgcagtgctaatggcagctgaagctatttatgatggat
tcagagaccatggcttaaacccatcagaatatacttttcctggtttggaatctgctgatgtgta
cggaaacaatccagtggaaattgcaatatcaggagatgatgacaatatgttgttgaacctcatc
tgcaactatggtgtatcttatgaaaaaactcgcggtcgggtaaatagatccttgttagatttt
taaaaatgaacacagcttcaaagtgtctcagtgttttaaagtttgttgaaaaacactttaaaat
tgagtcaaatacaccaaaaggagaatttgaagaaaaggccgaaacatgtgttaattgtcttgat
agaaataatgtgttgactaaagggtctgaacaagaatcttacaagctttcttgtggacactttc
ttcatgtaaaatgtttgaggaatatttgtatagtatcacaacacctaagatgtgaaaaatgtct
aaaaagatttgatgagagtattttgagaaagtgtacacctaacttgaattggtggttgactatg
ccggcaggtgctggaaatgaagaagaaatatgtttcatgagaaataagaaactggttgatgatt
tcagaaaattgttgtcccctgtctcaattcctcatttcttcaaaaatagtagacagcgaaatct
tgatatgttgtgccccctacagtgaccacacgataataccaaataaagaagatccaaagaaaaac
gaagatggaaacagagtgagggtcaaccacacagccatcagtgaaaagcagaacaaggaggaag
aagacgcgaggataaagcgtgtagccgtcaggacatttacagccatcagagaaaagcagaacaa
ggaggaagaagacgcgaggatcaagcgtgcagtcgacatggctgtcgcagccatcaacgaaaag
aacaaggaggaagaagacgcgaggatcaagcgtgcagtcgacatggctgtcgcagccatcaacg
aaaataacaaggaggaagaagacgcgaggatcaagcgtgcagtcgacatggcggttgcagccac
caacgaaaagaacaagaaggaagaagacgcgaggatcaagcgtgcagtcgacatggcggttgca
gccaccaacgaaaagaacaagaaggaagaagacgcgaggatcaagcgtataattgacttgactg
ttgatatgaggattcaacgtatagtcgacatggcaattgcagctgccactaaaaaggacaagaa
agaagaagagaaaaggacaaaaagggaacaagagttaagggctgatctgagaagggcaatggat
atggtgaacgaagtacagaagaaacttgaagacatggaactagaaaaggggtgtaataaggatg
aagccaagaatactagtaatgttgttagtagcagtagtgttgttgcctattctaaagaaattgt
accttgtttaggaaataataataatgctgtcattggtatgactagcaccaactattctgccaac

aatactaagaataatgtatttggttcacctcataaattttccttcaacgatgcatctagattct
ccaatattgtagaaactcccaaaatgtctttcaatttctcgttcaagacataaaataaaatgtg
taacatgcaatactgtttttttgtaaaacccttgtgtctggataaaataccacacatctgtgaac
atttcttgcacacgtaacactacgacttttcttcaactcacacatctatttttttgcacatgcaa
gtaaaacatttcttcaaacctctacagagagatggtatataaaggctttgtatctccatcattt
atcactattcgtacgatgacgtccaacagaccaacaacatcccctctttcgttctctgaaggct
tctcgctttcaggggataagtatgatacgtatgaggatatcttacttgaacaattcaactgttt
caagacatcttctccttcttctgctcgtaaaagtgaaatagaggataaaactttaatctttcaa
cttaaagaaggagaaaaattccatcttgcaaagggcatagaagagctccgtgagattctagacg
ataattctgcaacaattgaacctattatttctccaacaacattcaatgacagaaacgaattact
aaaccacgagggagatatatcctcaagtcccctatatactcagataatgaagcatatttcacca
gagcatgatatttatgaattggaccttattgttggcactgatttgctttttggtctaggtgtga
atctacgcaacgtttctaaactgatgaagaaaatatcgtatggtactttaaatgtagttgatgt
gtgccacagaaaatttttcaacaataggattatagttaatcccatttcttcgtcattctcaaaa
aatgtgtgtattattcctctattttctgcagctgaagaattttcgtctctgggggaatgcaggg
atttattcaacggtatttgtgatgacgtagagagatatatcaactcttatttttttctaccctga
aaatactactactactactactgctccttcgtcgcccgaaatggaaattgcagatgaggaagaa
caatccccaaaaactataaagagaaatgacaacgcaagtagaaactggtctggtgtctgtttga
tttttgaagtatttaaaaacacgtactacattattaatagaggagatagaggaggttcttttga
aaaggctgtgaagagtgcaatttcttctatcaaggaaaagagatgtaaaatcacagatattaat
ggtaataaacctcgattggttatggtgataactgggtgttatacagaattgtacttcaaagatg
cacttaaacagattggagaaaacaggcgcaaatttttgaaaatgaatgggaattacttttctct
gattgatgaacaagcagatctaatcgaattcgcgatgagtgtttctggtgccggggagaggatt
tttgttaacggtttggggatgttccagaaccgtaaaatgatacctgtaattgatcctctcacat
atgaaaatgttgtatgtggtgagcatgatatacaaaaagaagatgctattctttctgtaaggag
agctattgcagactataatgactttgtaagtaagaacaagagagggaagaaacgcagcgcagaa
gaagaaaatgaagatgaagatgcagacgctagcagcagcagcagcagcagtcctcctccttctt
ctcctcctgcacataaaaaatcacgtcttccggatgaaggcgaaaaatgtacactctgttaatt
ttttcaaacaataaactaaccaccttgtatatttttgtttgtttgcaacacccttttttatgata
ccattaagacacgatggcggtaaacttggataatgttcttgtgaatatcaacaacaaggatgaa
gatcttacaaaactcgtatccgaggcaataaagcggcgagctaaaactgtatttgacactaaaa
atcaagcagggtttgacatgagacgtcaagttgaagctgcattatatgaagcaatatccaaaaa
gaaagaaaaggccataaaggcattcgatgagctcatacaagaaagaggtgatgaaattacacct
ttgactacaatgcagtatgaagagtgggtaaaccgtacaataactccctcattgacgactgaaa
atttattaggtgatgttgagcacgccgatttttttactggaccgaatgacacccgtaagcgagga
agatattgaaggtttcgctgcttctacttttaaggaggtatcagattcaaaaactgcaacagtc
atagttaaggcagattgtgaaacgggggatatcgatgaagtgtataatcttgcaccatcattcg
gcgtcactcaagaaattaaaatatataggtcaaacaattcctcggaattggataatgtcgcaga
ttctttccatatttataaaatttctgcaacagatagcgacagtggcaatactaaaaaattgttg
tatgggttaaggaataaaaaaagcaggttatacgtgtttgtgtagaatttttgcagaaattgaat
cagatgggattatggccaatacaaatatcggtgtcgctgaaaacaacagagatgaaattgatga
aaacgaagaaggtaaatatggtttttttaatacccaaacaaccagctggtgcaaaattgatcatc
tacttcttttttaaattgttggacataaaaaatgatattttatacaatgcaacccttccttgggt
ttctggtcttttctgtgcttattgtaattgtaatgactgtattagctgtatacactgcacctca

aataaagaaatcgaagaagagaaaaattgaagatgaaaatgaggaagaacccgtaaagactttg
gaagattttgtaaagggtcggctccttaacgctgtcaaggaaaaacctgcagagtactttgagt
tgctaatatctgcagacactgaagcagcattaaaaactgccgaagaaacagcccttcgagattt
tgttattgagaacgactctgtcgaaatagatgtggaggaagtacttgaagagaaaccaagagaa
tatgtcttcaaattggcaggcgcaacaagcgaaacgctaacaaacacaatcatcgcagaggtac
aaaaaaaggcagcattaataacagaagaagatatcactattaaaatgttaaaacaattcagggc
tgcgaacaaagataataaagacggggaagcaactcctgaagaaaaggaagattttaccaataat
tcagatcttgtggggttgtacttgaacgaagtagtagaaaaaacaacaaatattgtcattaaca
aaatattccctcatgagatggtttttgaaagatgtgctattttaattgaagattttgatactgg
tgttgtgactgatcaagccattcagataccctccaacaaatacaaaatcagattagtcgaaggg
gatgaacctgaagtattccctggtgactgcttggatcttgcagtttcagttgataaaataaacc
acgtcttgaaaatttctgcaaagaacggatgtgaaaacaactgcttcgttattattccacggtt
ctctcctgtaggaagtgtttcttccatgatattgggcagcactgaccaagtcaaacctaaaaca
ttcttattttagccaacaaaaatgacagtacacattttcaattcacaatggataagcaacatt
ctgtagggtgtgagttggacatgttaatttttcagaaaggaacttgaggaatttacccgattc
aaaacctagacctctaagtgatgcagacatattggcctcatatggaagcgtctaggaactggt
gttttcacaacagaaaatttggtagacgattaaataaaatttataaccactttatctgtagact
tttatgtcctttgtgcaacaaaaataaacatgtcttcttcgtcttctgaaactcctaagacttc
caccgatactggggaagaaaggattaaagacattgtaaatgctctagataataatggcgagtgg
ttgtcttcctatattgatccgattatcaataattacatctcacgaaaaacggcagaaactgtcc
aaaaaatcaaccaagaagttgatgaacggtacgatagaaaaatagccgacaaaatcaacgaaat
aaaatcatccatctttacaagtgctcagactatgtatgaccaatatgcaatagacacatttcaa
gaaggaaaaggagccaacgggactggaccagtcatggggccagtgaacacggttatcgatacaa
ctttaaataaaatgaggggaaatatgctcgaatacgctgaagatatgtgggacggagatgactg
gaaacgattttccagttctatgacaacgcttgaatttgatctaagttactctgatttaactatg
atgcgtggttctgacgggtattttgcattccctttccgtggaacaaaaaagataaagatggacg
gttcaagaaagaaagaagacccaattaattgtatcatttcagtaacatatccaaacaaagtagg
ggatgagtgggaagagggtaaagaacgtgaagtgaattttaacctagaaagagtagacgactat
gaaagagatatccatgtttcaattttgtgcatgttacatgcacaacttgataatttcgaacaag
cattaggagaaaatgcaaactctttttattttaaaaggggcaaagagtcatgttcttacccaa
gaaatctaaactgttcaatagacctactgtagaagattctgatatgttttctataatattccca
cctgcatctgaccaagattttgcagatgatatttattatcgaataattgtaacatgttcataat
aaaatttgttgaaataaaatacagtaatacattcaaaatcttttattatttttcctttctaatc
caacatatataatggtgatacatttacaggtgatacctgaacatatttataaaaatcaggaata
tacataaacatgctattgtaagttgtattcatatccctggtgatcatctcgtgccagatgcccc
tcagcgagatggagcttgctgtaatctttgccctgtggagtgcttgtttgtatgcgtcttgagc
cctcttcatctcatgtgctgtagtttcatcccccctcacaaggctccatgataatattctttctg
gccgaattatacttgtaatcaatgaggtattgcttgaaaggcccagtcaagatgtcgttggttg
tagtctggatgaggtcacatttcttggcaacattcttaaaggcggcagcagcagtcttggcaga
cattgaattcgcttcaatgcttccaaaaatatcctcattattctcgtgcaccttcaagtagttg
ccactattttctcccacgcctccgtcaacaataaggttgcgaacgtatctgcggctgtgtgtct
tgttctcaacaacttccctctttagaagattttcaaccatcttagtgtacatgtcagatctcca
attgttgttgatgtcgttatgttgcccagaaaagttccctggcaaactaattctgaaggtcttg
tagcttccataaggagtagtgttattaatgtggttaatgggcatagattcacagaatggaatag

ccagtgtatggacagtagcgtataccttggcagtcacattatcttcacatagagcattaataag
agcactcttcttgctctcacaataaggcttcacagacttgtcgaaagaggaccagaaactctta
tcataatctacctccaccattgaagattcgtatgtagtggaacccatagaaaagataataagat
tgtccacatcgacaagtactgagtagtactggctgttttgcagatcaagtccctgatcctcctc
atcttcatcctcatccttcttctttgtagactcgatgtgcacacaatcctcagcagcctcagtt
ccggcagggaacaacttaagagacttgaggatagtggacatgttgaatgagttggtgatgcatc
cagaggagtgtctgcatagagatagacagacaggagttgatctgttctttgataggagagaaac
atccatctcttcctcagattgaggatatttggtcactgaagcagatggtctgaagctgttgtct
gaagccaggtagagagggttcccatcgctctcgtcgaattcgcactctgttacatccccaaaca
caacttctgaacactgggaagttgcaccatcatcatcctcatcttcttctgaatcttcactgct
actgctgataacactttccttgtcatcttcatcctctactactgttgctgtatccttatcctta
tcctcctcatcctcttcctcttcttcttcagcagcagctacaacagtgacagatgaagacgaag
aggaagaagaacacactgatgaggcgtcgtcatcatcatcatcggagtcttcttcttcttcctc
tgactgctcaccctcttccttttcctcctctacatcactgagaccgatagtttttctaataaga
ttcttgatgacatttccaactgaactttcaatagtttcattgttgtaatcgtaatcaacttcct
cctcgttttcatcttcgtcttcatcctcactgtcgctgtatgcatatgctgacttttcgtcatc
ctcttctccaaatacagtattgttcagtgacttgaggttactctccctcttggccaaccacttg
tcgttcttgaagcaacaacatccatttctcatagattcagtgacactttcttccacaatttggt
tagtggtcttgaaatgtgttcctgccttcttggtagacgaagaactgggagttgacatgaccaa
tccagttgacttgattgcactatcaatatagtttcctccaagttccttaaacagtgacttatct
ccattacttccaaatatctgcctcttcttctccaaatccatctcgtccacatatacagcatcag
agagcttcaatgtgaagagagggcctcccttatcaagggccttcttgttgagtgtttcaatagt
cacgtgctcatggaactggagattcatacaaccaaggaatagattggccagttttgtagtatca
gtggcgattgcagttttcttcacagcttctccctcaaccttgacaatgttgctaaccagatcct
ttccttcaaatgccgacttgacctttgagacacacccatcacaaaaggtggaaggggacagttc
tgtgcgtccagagttcatcacactattgaacagtaccgatttctgcgccttcttattcttctcc
aattcttcgtcctcatcttcgtcattctccagaaattcagcaacagatttggccagaacactca
cgtcaagatgagctggcaccttgcccataagagcgtccagttcttgaagagattgctgcttctt
ctcggcgggtgtgatttctggaggagtctgctgctgctgttcttcttcctctcctacaacttct
tccacagtttccgtgtgtggactcctgatagtgttcctcctctgcttagagaccattcttcttc
cagcagcaaacttggccttggaaggagttggtttcttggcagaagtagtagtagtatcctcaaa
ttcataaacttgatctggttcctcagagaaagtgacagttggctgctgctgctgctgttccttg
attgactccattttagaatattgggttattaggattcagtagttctgttattcctagacaaca
cgtcttgtctgtacgattagtcgagatgatgtgatgtagagcccttgcccgtgcctcaatatat
acccctcagtagaccttgataatcacggttgccaaacaacaacggttggaaatcttagtagaaa
gaaccaatggaacaagtcaggataataatatacagtataagcttttggggaacaacattcattt
attgtcatagcgctaacattagaagtacaagaacaggtgattctgcatccagctcattttgttc
aacccacttcatttgcaggggacaacactcttctttcttgaaaaatatggatgaagacacttgg
aattcttcaataggccctggggcgttgctggaggaggacgcagaagaagaagaagaacagctgc
cgttgaacgagtttatagtttccagaagacaacataatttagctgtaattggacgaggagactt
cttgtaatgagagaaccacaaaattcttcttcccgtcttttctggtataaaagtagtcccataa
tatgttcctttctggactaccacattatcttcgtttaagggaacatatctgaccgactttctca
tcaagggctgatctccatttcttgttgtgcttagagccgatttgtattccccgtcgttcacgtt
atagttgcatatagaacagtgggatgaaaggaacttgtcggttacgtgttcagtttgtacaaga

taactggacgcatttgctagttctggagggacgtcctggtttttgtttattgttgaagtaggtt
ttatctttttcctatttaagcacacaccctttgaattaatgtgtatgcgattcgcattgatgta
tatgcagattgtgagtgctggagaaaatggtatctcaaaatcggtagctggttcaataatttct
ggttcaacttcctgttgttcttcttcctcatctttctcttccttatttttgacacctagaactc
taaggttggcagtactaattattctggctgcagccgtgctggtagcagaaggggaagattgttg
ggagataacaggagacatgttgaccgtgccgtgtcgtgaggataaatggctaaaggtatatacg
gcatgccctttttccgttactgtgtgcgaggtcgttttctaggaccgtagatgaccatctgcc
ttcctctctttttctggacaacctttaattgccatggtgttatgagaggtaaactcgcctcata
gaatccgcaaacttcctctaaagggaacaagtgtttccccataggggacaattcaaaaatataa
ttccaacatattaagggtgttgttttattttcctttttcatcgtactccgttattgttttactaa
aatttggaaggtgatttttttgatagactatcggacattatatccgctgcaagttgacgtttatg
ttcaatgacataatcattcgaatcaaagttgtcgtgtgttgacaccataccattccatttccat
ttggctagtttggagagggatttattgttgcgcaattttagacgtgcagaaacgttttgccata
ttattttagaaagtacaggcaaaacaacatccctcacgtcaatactgtccattaatatatcagt
agaatcattgaaccttataacatctctctgtatatcttcgtttgatggggccaatcgaactaga
ggaactaaatccccagttatgagatcggtgaaaataaagctctcgtcatcaacaatttttttgg
agcctatgtacatctcacacttactataaaagtcattttttataccctggtttcttttcccacct
agaacataagagtattaaaactctcgacaaattatacatggcacctactcttccttccatatta
ctgatagctctatacatattaaccacgtattgtataggtgcttctacgtacaaatctgaaaatc
cactagcggcagttccttcactaaaggctagaacttgttccatagtcttctcttctgaaaacat
ttgctgaattattttagtggatagtttgtgccgcttattgaattttgtgccagggtactgggac
ccgaattcgttgaaatgttgttgttgtttttttccctacttctttagtttcatgccaggcggggt
gttcttgaagagcagccacctccaggaacaaatcacagatggctcgtactccatcacatgtgca
ttttacagtttcagggaaatttaatcctgttattttataaaacaactctctgtctattattttt
cccattcgtaggcgattattgacattaaacccacaacactcgcctctgattcccattataaagg
ctaaagaatatactataatatttcccgcacttgaacccacgagtctaagagttgtatattctcc
atcgcagttatagagacatcttgccaattcgtccattgatttcctgcccgtgaagcgcacatcc
atgagtcttgctcctccatatccaatttcagcgtttattgaagtcctccaacctttatccttgt
ttaggcgctgtatctttttcgccttctctgccaatacattcataaaagtgtaaaatttaacgtc
tcttacagattccataatttttttcctcaagtgttagcggcggcggtggcggcgatccttctttg
tagccttcaaagataaaagagtgttattcaacttacagtgtttattggcccagcaattcttttc
tttcatcaactgcatagtagaaggtcgatcggaaagaatgaccaatttggaatccaaatcccta
ggtttatctagtccagcttctatgtccatactctttcctcctattatcatatagtcttcaaaca
gtgttttattagcagacatgttaatcatttcaattacagtctggaggcatgtttcatgtctatt
gaccagatcttcatcatcttcatcccatgtcacacccaccagaatatttctcctttcttgagt
ttggatacattttctgtaatctttttcattatccgttgtttcttggacgccacactttcgtagc
atagcgtcggtaggggagagtcgcaatccacaaccaagtatttttttcatgattaatttgttgag
gattgtgttctttgaattgaagaaaaaaatcattcgttctgtaccatggagacaactatggata
atgtcgttcagaataacgacgtaacaaaaccaacaccagatgttgctactgttacaacagcaac
tgagaaacgtcagtcatgcaaagagaaaaaggatcaacttaaggccgaatgtcctcaagtactg
agagcactaaaattgtccaatactttaaaggcaaattttggaaaatccatgtcggctattttg
ctcaacatttagtggacatgacaaacgctaaacactttaaggacccaaagacaaagaagatttt
agaactggatggaagtagtagcagtgacagtgaagaagaggaagaaactagttcttcatccaaa
cggaaaagaggtagtggtgctagaagtgcttcttcaaagaaagaaaaatgccccaatactatca

aaaattggctcaatgatgctcaaggtgtattccgccagtttgcagatatcatcattaatcttcc
ctcttttgatgatcttagagacgaagtaaaggatgaacaaactgagctaaagaccatatatgac
ttgtatagacaggacatggaaaaggtggtggaagaagttttagggcgccaagacctgtttgatc
acaagtcagaaatagccaaaggtttggcccgtttcgatacccacgtctcgttgctcccttcgga
taggtcggctgttctagactcgtccatatccaaagagttggaaaaaaatagcaagggcccgaac
agtaatatttttgacacactaaacacactcaaggaagaaatcaaagaacttttatgtcatcatg
tcaaatatttattgcaaaatcttacaccggaggatgcaaattttgtgttcaatagttctgtaaa
gtatgtaaagaaatcatatcaatactacatacaaacatcagagatggaaagtgatgaatttaag
tcccttctcactggagtcaacattaaaatattggagaagataatctcgtccgataataatgttg
ctactccttacaaacacatcactaatcccaggaacattatttcgtctttacaaaaagtacgtga
aactaaacctgtttcaaaggattatccgttcagagtggatacggccagagatattgtactactt
ccagagacgggtggcatttctgatctccctatcaagcccgttacattattgcagttggtgtcct
acatcaacgctctcttttccctcgagcgccgaaatgttttcaccgacggctttttaatgcagc
gtgcgtcctaatttcccagtgcctaacgaatgcgaacctattatctaacgactttcctaaaccc
attgaactggcagctaatgttactcgccataatcttctgagtatgaaaatgcttcaagaaggtt
catccagtgaaaagaagagtaaaaagaaggagaaaaagaaggataaaaagaagggcggtggtgg
tggtgacgattctgattcagaaacagattcttcctcatcatcatcatcttcttcttcttcttca
tcctcctcctcctcatccgaagacgaagaagaagaaaaaggagaagcagtagaaaaaggcaaga
aaactaaacgcaaaacaaaaaagaaaccatcaaaggacgacgatttagatacaattagtaaact
gattctaaaaacaggaggttacttccacgacacgagtgaactcggcaataaaattagaaattta
atagacaaggatgattttgcgggcgtagcccaatatgcagtaacaatcactgagatgcaatcta
cgccaatgaatcaaagattagtatctagtcttttagatttgataatgagactaaaagaacaagt
aaaatatagtgttgatactgaaagtacttcctccaccgccaaatctaataatgctttagatagt
gctaaattgacatctcaacaagtggttacaatgatggtggattctggagctgaattggcaaggc
tagctgccttctttttcgtggtggtggataatactgtagttaatcgccatgaagcattcattct
aacatcaaaacttttaccctcgaatgaaaatagaggacttaaaactgttgtagagtcattcttt
aaaaatttaacaattagtaacaaagtctctacctcgaatgaagaaatgatgtcggtgatgccgt
tcgaagacgaacaacaacaacaacaatgccctcaacatgaacaacaaccggatttgaaaagagt
ggtgggagaagtatttctagaaatgggaaaatcaatagtgaactcattcccttccaataagagt
gtacaattaacggctgacgcgttcaagcaaaactactcgcctatgggaagacgcataaatttgg
cggccaagataaaaacggctatatctatcggatctaatatctcgcccaatatactattttctaa
cctcccagaatctgtagggaataatactgtaactggtctaaggctaaccaacctattgaaaaac
atatcccaaagtgcccaagctaataatataatcaaaaatgccaacacgctcgtaaacaatacga
tggaccaacaaaactcagcagccatgtccatactactctttcctccaacatcaaaagaaacttc
tatctttcccggaaacgatccttcatctataaaattacaggatatgactacaatgtcaaatctg
gcacgaggattttattccatcgcagaaggatgtatcggggtcgtacgctcgagggaatttgatg
aaggaggagttaaagcgtacactttactagtggactcaaatactatggacatggcagttaattt
tgccgctcagtctctggaaaaatcaatgtctgaagccttaacaaataatgcgaatatgaaccct
tctaatgtattagaaggaggatcttttgtagacggtgctctttcttacatgtttgaaaaaaatg
gcagtgactgtgaacccacccctctagcaaaatatacaatgaaagatgtatcaaatcgatactt
gaaaaaattcaacaatgataaaaatacacaagatttatataaaaatagagcagaaagagcactc
gtcgaacaggttacaaataagcctacaagtgttgtgcattcccagctagcaaacgccatggggg
tagctgttataggcgcagcatcaatcaagctcatggaagcagaagcagcggaatctgaaatgag
agcagcaaattatcaagcaacatcaaaatctacaaatgctattaatataacaaatacaattgga

atgatacgtaatactactcacctgtgtacgaccatcgccgtgagtgcagccgccgacatgtcaa
agctcgccaacaaccattttatgagtgtattaaacactgcaaataacagtcatagcagcagacg
aggagacagatcgtccctgatgttgcagcaacaacaaccaacacattcagcatttctggaacag
acaagaggaagaggaggaggagtattaggatcaggaactgaacagacaaaggatcatgtggaac
gtatgaagagagattggatattaaacatgatatctccagaagacaaaaatactactactactac
acccagtaatgccggccggacattaggatatggatctaatataactggcataaacacgataaaa
caagacgacaagagtatgatggataaactttctgagatgtctagcttcagaacttaaagtgcca
attttcatataaaaatgtagtattatgtggtcgtttttaaactttaagcgtttgtggttttggg
tcgttcacggtccaagatgtaagagtccccttgtacccaaattataacaaagtgtcagtatggt
caatggaaagtatctaaaaatccacccgtctatcattcaatatcccaactgaaccaaccaaccc
cagaaccaacaacatgtctcttgttgagaataatacccaagaagaaatgattctggaaactact
gttgaaggtgttgtggagggagcagaagttgcccctagaggtgtcaagagaccccttccttctt
cctcatcttcttcttcagcttcggatagtgaagatgatgaaggaggagagcaaccacaaacgaa
acccccaaagaagaaacgtaatatcaatagcggaaagtactggaaaatcgaaacaatcgaacca
gcatctccagaaatgctcagtgctgtaaatgatattgataacgtgtccaaaactattcctctaa
tcgataatagttttggcgttcagtttaaaaagagtgtatctgaagaacagatcaaaacgctact
cactgaaactattgccgtggaatatggaacaatcacaaatgttaaatattcaacctttaatcaa
ctcgaaaggaccggagagcctcttaagaagaagcgctcaaacaatggaaataacaactacagat
actggcaaattcgaattgaagccgctgcagcagagaatgttacacaagctgttttggacgcaat
cgttgaaggaaatgacaccgtgatcaaggcaatcctcctccctgaaggggagggaatcgggctt
caatttaacaagagtgttagctctcaacaagctaaaaatattgtccaggctgccgatattgaat
ttggacaagttgcgcacatgaagtgtaatttgttccacaagatggagaaggccgatgaatcttc
taattcttctggtgaatcaccaaaggttaagaaagtaaggaggaacaagtcccagcctacaaat
tcttactacactttcactatgatcggggattctcttcaagagcgtattgataacgcaatcaaag
ttatcgaaatgtctccagttaagaggccattctccaattctgctgctgctgctgaagaagacac
cactactacgaccactacttccactggtgttgttaatccacgagggattaaggatattcacttt
ttcgattcatccatttctaagggatgtttcactgtgaggaatattgttgcagcaaatggtgaag
ttccacaagaagaattcgtttctgagttgtacactaaccttctaaaggttgaagagaaggtgga
tcatcctactttcaagaagcttattcatgaccgtacaatgaatcgacacattaaggcatggtac
tgcatctgcccctactacaccacaggaggcgtccccccctgcagctgataaggtttccgctaagg
gaattgctacctacagaatatacgaggacaggactggagtgttccaattcgatggggcacatac
atctactaccccagcacaggcagccgaggcaactggtgctattcacaagtctatgctcttccag
agtcctggaactgatattcaaaagttcctcgatgctaagaaggcagaaggtttggagcctattt
catcgggcgaaattgtgtaccgttcaaagtggagccccaatgatagcagggcaacacgctgctt
caaattttattcctcttcagacgagaagatgaacattgctgatgttttgtctattgttcatact
gatggacttttcagtagtgtacactttagaaaggatactatggaatatggtgttgcaaagagca
agagtaaaattatccccaaaactatcaagattaagaagggaggagatactttccatagcgagga
ggatattgaagtgcctgtaaaattcactgcaatcacttcggaggaacttaataggaatgtaac
accaagggaatgaacagcctccgagcacacaagaaacgaaagagcaactcttctactactacta
cttccaccacttccacttcaacaacagcaaatactccaaagaagacgaagaagagcgcttctgc
tgccagtgacccatttgcaaagcttactttagattatgttgatagtacatcatttgtattttac
aatatcagtaaggaaatggtgcagagaattttggctcaggagagagtaaagacgctaaaggcag
tcaagaacgaagagaagatggaaattgtagagggagaagaagcacaagaaacttatagaggaat
cgtaaagatcaagacgaatgcaaaggcatacaatcttgccaacaaaacatctggagtactttc

cctgctgataaggtgtgtctaaagcacacattggaagatttggggatgtacttgattttgatg
ttgtaaggaggataatgttaataagacggtcgcttctactactacatcatctgaaaataa
ggcaagcggaggagatgatgaagaaactccaatggaatttgaaactgacggagagaagctgttg
cacgaattgttgaatgaataatgttgttgtgttgtattttgtatatagagaaaagaataaaata
aaaggttaatataatgttgtcttttacattttcctaaacttgatataataaactatatagcaaa
tattaatgctattgacacatgttactagtccctagtcaacgtgtaattgtacatagtaacatgc
cttttcttaggcatttgaggcaaataggaaaagagtatttacgatgggtgaccgtcgtgataac
gcctacaccaggtgacagtatagagggtttcagcagactgcaaatactcacctaacaggccttg
gtatagaggaatcaataaaaaatcaacaaaaaccttaaaagtttggtgtttcatttttttacat
cggggacagaaatggggttaaaaacaaatatccgagctgcaatctgacattggctccctaccca
gaggtccaccctccgaacttgacattcagtcgacccagcggtccaccctctaaactcgatcgag
ctgaaaaatttttgaaaagtttttgagatggagaatgagtaaaattctctagcggaaacagta
ggttacacactaccctttctgggtacgttcatatccagaaacgcctgttccagaaacagtaaag
agttaatgtatgtttctggagcaacagacgtttctggtgtaatctgacatcgagtcgacccaga
ggtccaccctccgaacttgacattcggtcgacccagcggtccaccctctaaactcgatcgagct
gaaaaatttttgaaaagtttttgaggaggagagaggaggtaaattgatacagatagaggtatc
tttagtagaggtggtggtccttctaggagctggtgtctccagaaacgcctgttccagaaacagt
caaaagttaatgcacatttctggagctatcccatttctggtgcaatctgacattggccctaccc
agcggtccaccctccgaacttgacattcggtcgacccagcggtccaccctctaaactcgagtga
cccagaaaaaatttataaaattttgagtcggagatagaagggaaattctctagtgaatacagt
aggttacacactaccctttcggggcgcagcaagtctccagaaacgcctgttccagaaacagtca
aaagttaatgtgcgtttctggagcaacaaccatttctggtgtaatctgacatcgagtcgaccca
gaggtccaccatccgaacttgacattcggtcgacccagcggtccaccctctaaactcgagtgac
ccagaaaaattttataaaattttcgtcaagggaaaacaacactaagttgcttgtcaattcata
cactagtgcaggttggttgagatagagagtgaaggaggctatccagatatctggatcaaccaga
atccagaaacgtttctatccatttctggaacagccatttctggaaagggttacattttattata
tttggtatattatttctggtaccacttctggcacattcgtgcaatctgacattggccctaccca
gcggtccacccttcgaacttgacatcaggccgacccagcggtccaccctctaaactcgagtgag
ctgaaaaaatttttgaaaagtttttgagatggagaacgagtaaaattctctagcgaaaacagaa
ggttacacatacccttttctggttacggtcatgtccagaaacgtctgttccagaaacagtcaaaa
gttaatgtacgtttctggagcaacagacgtttctggtgcaatctgacattggcccgacccagcg
gtccacccttcgaacttgacatcaggccgacccagcggtccaccctctaaactcgagtgagctg
aaaaaatttttgaaaagtttttgagatggaggacgagtaaaattctctagcgaaaacagaaggt
tacacctaccctttctgggcgcagctatatccagaaacgtctgttccagaaacacacaaaggtt
gatatacgtttctggagcaacagcgtgacagtagtttctactaagccttcacttgtacacattt
cacttagtataatttgtcatcacatggtcgacaaaaattttataaaaatttttctgggtcgctc
gagtttagagggtggaccgctcgctcggtctgatgtcaagttcggagggtggaccgctgggtcg
actcgatgtcagattgcaccagaagttgttgttgctccagaaatggtgggggagtattcagagt
gtgtttctggaacaggtacacatacccaactgatcagataaaaattgggtgtatacattttctg
atccgagaaagtaaaccaactgaacggtgtaaaacgtactgaaacattacatcacccacagtcg
tcctcctcccttctaagagagaacatatcccgtacccaactacctcaaacaacaacaacaacaa
caacaaccatgacatccattatggacgatctctacatgagctgttcttcttcttcatcgtgctc
aagttcatctgaagaggagaatgaggtaggagtagaaggaggaggaggcagaatcggaccaaca
gaggccaagaaaaagatcctccgtaaacgaaagagatcttctgttaaaagtacatcatcttctt

cttcctcatcatcatcatcagacgattctgactctgatagagaagaaaaagaaggaagaaaact
atacgtggatattgcagatacaaggaaaccgcccaaagtaagaaaactggatactccttcacaa
actttagagaacgacctctacatgtctagttcatcttcttcttcctcctcatcttccgactcat
catcgtcttctggtgaagaagaaagtgacgatgatgatgatgacgattatgacccagataatgt
ccatgttcttggttgtaagaaggaaaaatctccccaagacatagaagctgaaaaggaaaaggag
gaagagtatgaagaagaattcaagagaatggcattaccttcacggataaacacatctgtagatg
attgtgttatacctgatcggattttaaccctcttttctacacttttgaagaaaaatagtttcca
gttctctcagccagtttctttcctccggttggtgatgaagcaagtaaacgaggccatgaattca
gcattctcttccatgttatccagttctggtatgaggttggtggaggactctttgggtgacacat
ctaaaatttcctcctttataacacctcaaacggatactagtaattcatcttcatcatcgacgtt
cgtcaataattgtacagatgaagatatcaagaagcgtaacattgcaatgggaagagtagcagaa
ttgctgtcaaatattgcagccagttctaatgaggagaataatttccgccctgtagtttccttaa
tgcgaggaccaacttgtggaggttctaatgcatccaacaagaaactcaatagtaacaggcaaac
tattccccaagtattaaacaaggttatatttttagagaaatacacagtgttatcgcgctatat
ttatcgtccgtctgtgtgcagcgagcaatgaataatgacaacacaaattctagcggatacgctg
aagggatggttactaaaatcttgaatattattggtaaaattccttataatgaaatgagtagaga
aaaattcatatccgttggaagagatgcactatatttgtaccagaatgtgatcacggatatgact
ggccccaaacataacaagagactccgtatccctcaacaacaagctgattttgttacattatag
ccatgttggttaatgatgttcccattacctcagatttacttttaactggaaaggcgacaaattt
agtacaatttgcttctgctatggtggatcctgcatatcgcctggctgtccataaaatggcgtct
gttttcaatagtagttattccgtatataaagtcctagatcttgaccataaaatgttattaaggg
ctaatttaatcctatctattttatcagctagaaataagtgtctcagtgaaagaaaacctagaac
attaactcagagcgtgtatttgttcctaaatcatcttttgcggaacaaattgaggtctagtggt
ttgaccagtgaagagagttctctaggaacagccgtaaaattggtgtcacagcaattaatgtatg
agggtgtgactcgtcaaaccatcgaggacggatgtagcatgattagcggaaactttgaggatga
agacggtgtaacactgaaatgtttgggagccgatgttaaggacgtgaagactgttggactatct
gcattgctatctgataggcttagaaaaaacattagaagaaacgtccctttctactgaaaaaata
tggaaactactacgctcgctttcattgcaatgggtttgcttttcgtggtgttgatggtgtacat
ctttatacagtacacgtcatcaaataaaaacaagacagaggaaagaaagggtagtaaattatca
tcatcatcaacttcttctacttctggtccaacatttaagagagaagcgtctcctgaattaagag
aagacataccactttctccacgatcttctggtccagaactgaatctaactgacgctgaatttat
agaccctgaactcttttctgaagccagggtcagtcccgaccgttaacggaaaaaaggtgcaaac
cggcaactcttctccctcctttcgagtataagtagaggcgttgcaccgagccaagcatcattcg
ggctcattcaactgtgctgacaacaactaagaatatacattgaaataacccaaataatcaactt
ttcaagatgtttggaagttctgctaataactttaatggtgacaagaaatcttcctcatcatcat
cagctgccgcatcatctgacgatcagcagttaggtccccttggactgtctactgctgatttcaa
gaaagttgctgccattcttgccaacagaacagagtccctatatctgttgccagattctcccaac
tttaagaatgtgattaacaaccctaaccagatatctattgtgccgtttctgggttcatcgaagg
cagctgaaagtggtagtgcaaacaagaatgaaaaccaggctgaaaattcttctaaaggaggaag
tgatggaaagaaatcatcgcagcagaacaagtttaacctattgaacaaggtagaggctgaagaa
atggcctttaaacgtgtggctgaactcattgcggatactcctccctctaaagataatccattga
gggatgaccctgatgctattccatcacgcaacccatgggtcaaattgactcagaagaatctgga
atatcttttctgggaagcagtaacaattgaagtctccaatgataggagtatccgtagtggaaga
tatcttcaagccagtgaagtgggggagaatccattcctaatgaccatcagtgttgacattagaa

tcctgcaaagaatggcacttaatgtcgtgtggttcttcaacagattcttccgcatggtttctgg
acttggagtagaaaacagggccaattccacctatgtagcaactagcgatgctattgcccagatc
tgggtagagatgctcctcaagaactttatttctggagaaaatgtgccccaggcactgaagtatt
tgaaggaacattatgagcatgtttataacaagatcagtaaatgtggacgtcagccatcctattt
tgttgttgaatttgaaagagtggacaacacgattggatttgtcaattctgatactgaacataat
ggatcatcatacatggaatacaggtgctttgacacaatcaggaagaatgcatcatctgggccaa
gtggaggcggaaagagtggtgtcctgtcttctggaacattcttcattgataacgaaatggggaa
taacaatagtagtgcagctgcagcttctgcccctgctgtttctgctggagtttctccatcactt
tctccatttagcagtgatggagatgatgatgacgacgattgtagtggtgatgacgtgtgggga
agaagatgatattcaacacatcaggagacggatcaggagaatcttctgggcagaacggtggtgg
tgcatcaacttacaagagatttaggtgtggagaaaatactgcttctctttctcagaaggaaaat
gttcgtctcatggcgatgccaaagggaaatgaagataaacaactactcaagaacattatcaact
ttctgaatagtgcacttaactctgttgaaaaccatgtaatgtgtacagatgaaaatatcttcga
tgaggatcaagccgagcattatacgtcaaacaaggaactatacaaggctattgtttgctccaac
ccagccaatgtgtacagagtaatggttgaactgtttgtcaaccttattcttccccgtcttagga
acccaattgtgagtgacattgaaactgtacaaaatcttccttcaaacaatggatcggtgagaac
aaagaagatggttgaacatggatgtacagatatgcgctatgacatccctccatatgcaaaggga
aagattcgtcttagtgccaagagagcttgtgagtgcagaaaactgtgcaaggacgtgaggtgct
ttgacaagtctagagaggcaaatctcacccctagccagaaggcaggaagggaggtggaggaacc
ttttccccgcaaccacaattctcacaggagtaacgctcacgactttactttctatgacaagtac
agggcaaggatgaacaagctcaagaaggattcaaagaagaaggtaaagaagattgacacccttta
caacaacagacgattttctcctgcaagataggaacgcttttgatctacttagaaagtgcttcct
ttctgcctctcttcatcacattttctgtcctgatgtgcttatggtgcatagaggagacagcttc
aatattaactttgcaaacaacaaactcgagtgctataatgaacgtaacggaattgaagaagtta
cttcctcacaaacggtcaacgccaaggaagcacttgaggatattacaaaaattaaaatgaagag
aggggatgatattatagatgttgtgaagagtaaaggactttctttgagggaattctctaagaag
gttagtaagattgtgagaaggtttaatgagatcacaaaccaactctgcaacaactgcaacgtta
actcttctaatggagatgtggatttccacgtctttacttctgtgtgtgtctacatccacaacat
tattcctgtgctcgaagatatctccattttttgcagaattgggtgaagaattgaccaagcttgtt
aaggagtgtagagacgtggctggagaggacaagacatatgatgatattatccgcaattacgaaa
ttactgtaaagtactttaagctctttaatgcactcgttaaattctgtcacaggaattataatgt
ggcagtaacctctgccattaacaggagagggtacatgtgcatggtgagcaaccttgtcgggtat
tattgtaagctgtctgataacgctatccagtatcacgaatcactatgctctttgcactctagca
tctcttatgcagactattatacgtctcgcaataacaattctgaagatggaggaggaaactcttc
ttcagaaaagagcaatgcagatgtagccaagactatggcctctttctatgaccagttcgataag
agtgaagacagcaagaaaaataagaacaaaacttcaaatgagatccttataaaaatgttccaaa
tggatagggttttggatggcatggatgatgatgatgatgaagatagtgatagtagtagcagtga
gaatgaagaggaggaggaagaggaggaaattgtaaagaaaccagcaaagaagaggaaagtggaa
gatgttgatagcaataagaagacactgccaaaggaacctgccgttaagaaggtgaagcaggaag
aagatgtggagatggaggaagtgaaggaagcagcagcagaagaagaaaagaaagaggaacagga
ggcgaaggaggaagacgctactgagtatgacgacgatacagaagaggacgagaaagcagtagca
tctgatgaagatgaagatgatgaagattctaaagctattttctaaatatcatgtgtataaaatg
tagtattttaaaggtataaataaacacaatataaagttaaacattgtttttttatttgacacatg
tacatcatcaatttcacatggttatatttcttgtagcttgctcggcagcaataagagcaagaga

agagtcatataaaacagatgtctgtgaagcagccatgataccccctcgatatattattaataagg
ggagttgctgctatattaacagcataataaatcaggttcttccaatccctagaagcccacacat
tgggcattttatataaaaatcaacgtctactattgacctggcaacagccactatacctagctc
atcaatgttaacaatcttcccctgaacacccattacaatttttgatttttctgaattctctcgt
atagcagcaatacggctagcttcattatttaaccattccaattttacacttgaaacataagctg
gggattttcatcagttacttgagatacaatattattccattgagataaaagttcctccaattg
agttgtagtcttgtccgcttgctcttggaaactttgcatgacgatagccgaatctcttgttgca
gtttcgatgatggaaatacagttgagccattgcatgtcagtaaggatatggcgcggattaactg
gaacaaacagattctgtgcaggagaaaagttcaagtcttctggaatcttgttgaggttgatatt
gtccttggtgtgtttccatgggataggtttctgtgcctttgaagcaataaagtatgatgggatt
ctactgtatttctttagtgtttctgaaatgtttgtctttatacggtcctttcttccatcatccc
taatgtaattcaaaagtttagatgtgaatagagaagagtctctaatttttttgcatttgttcatt
gtgaacctttatatcctcgggaacagtattgttattattagcctgcctctgttgacgctgctgc
tgttgttgttgttgagtagtacttccaatggtatttaatttgtccaatattgaacgtaattcta
caggcacccttctaccagttccaagagcacctccaccgccgatggccgctgcagcttcttcact
agcttcatcatttataaaaagtctccccgaagaagcatagcgtaaagatccatacccttcattt
ccagtatcaatagtaatcccattttgttcagctgttatcctttccagtatttcttcaatggcct
ttctaggatctagtgcaacatataatccatcgaccataatatgagacacgtgctcgcacacttc
tttaataattttcatctcttcgttgctgacaagcatcatcattctagatataatagtagcaata
acattcctaaatttgttctgaagaaatagttcgatctctgtgaacgtaggctcgaaacgggcag
gcacattaactggaacaaccgcttgtgtttcttgttgttgctgctgttgttgttgttggatcgc
tcttcttgttccaccacctactatattattactgcctctgagttggtcggctgcaatagcaatg
atgttttgtacacctttaccagattctgcaattacttccatttttgagtcttcgcctgaaattg
gtacagaaggaatcttgggtgacaacctttctagagttgtgggtaatccagtatcatactgaag
aactttccaaataaattcgaatccagttcctgccactgtgcccactttactggggaaaatatcg
atcctccctgtagatagaggactaacacattccttatagatcctcattgggcttaaatgttgtg
tcgatttagtttttgaggcttcataaagcacagcagacgagagaaggggttcaaagaatacagt
ttccatgcccaattcctttaccgaatatttgacttgtttagatgcatgaacaggaagatttgtg
ggctgagaagtcgtaccagggaggaatgcgctaacatgttcaattttcccatcaaccgaaaaac
ttgtccgtacaccatcaacgaatgaagaagaagaagaagaaagggatccagtgtcgtcttgatg
gttattaatatcaccaatttccatatcctttataccatcatcgcgttcagtattctttacaact
ttaaagtacgacaccaattcacctttatttttcaatccaaggaatccatcttctgcgatattct
gaccatatgtcacatttcttgttagagatttaggtggtacataatttaagaacgattttgaaat
ggtttcagaagaaaatggagcagtggcagttgtttgctgctgttgttgtttttgttccttagag
gatattagaggtacattaatgaaaagttccagatccttattcatctttaaaaaccgtcctgttg
gtacaggaagattaactaccctaatagggggtttctgatgaatatctggaggaggatgatggaga
agaagcgactgaagtggcagactttaagttttcggctacattatctccattcaaaatactctga
ggtgtatcggtcagtgtatagtatctgagagacgtatcatttctacaataaagggaggatctc
tagggaaagcaaggcacacgcctccattatcggctgccgaatgaataactgcataatgtgtaac
aatctttgcaattagatccatcacgggcttcttgaataataaaaatttgtgttgatcaggatcg
gctgtatattttagtagcacttcacgtactatataatctgttagttgtctagcctcctttacag
cccatacgcccgatttcaaaacgcaatcaaaaaaatggaagaagaacaacttgaagtacctgctg
gtgcatgtacatccaagcggtctcatccactttcactggctgtcctctcataatatttcttgtc
attgtttctaaacacgaccgataaagacccaccacgcagttaatgggatcgtaccatctgtact

gccgtccataacttcttcttccggccagtataggctcccctgggaatagtatggaatcgcagtt
agattctcttccaatattgtatatggatgcaatctctcgagcaatatctcctaccgtaatagca
gatccgcccgagaaaagtcccacaacagcatcagagacatttgagaattccctcccttctagat
ttacattattataatatatcagagatgctattatagcagcagaagattccccagaatcgtctct
cagaattaattttaacgtaggaatatcaacatctggtataaaggctgtagtattattttgaccg
aatgtaagaaaccttccagaatcgtgtatttccgacacgttactttttctgtcgacaacgagtg
ttttattatgttcagcgtcgttaagagaggttaaaaaaaggtgcatttctatccagtctgtttt
ctccgatatacagtccatgattgaagtgtattgaggcccaaagaagcaagtaactgggtttttc
atggaaatggaagtaggtgcccctgaagggacaatgttggtatcggtagaagtttttttgcctct
ttgtacgttttctattttcatcgtattccttagaaagggtggaagatacatctcctaaagattt
tcctttagacgataaaatcgattgtgcgatttttttccttttgttcggtcgtcaatgaggcggtt
gaggaggaggaggaggctgctgttgctgttgctgttggttcataaagtttgggttgaggtttat
ttttaaatgatagttgataataccgtgtaaccaaatcctttaaagaattggtatttttttccaaa
aaagcactgggccgtaaagtagtgcataccaaattcagtcgagttaaattttgacaactctggt
atgataatggaagaaactgacttggagtttgaagccatgtcatttaaatcacgtagccttgtaa
gagagggatgttgatagagaattttctttgcatttgggttcttactggtgtcaggaaatgctat
ctggagggggtgtttatccacaccttcacatccattagacgaaatatcacagggtgttgtaat
aatttccctgtgaatctagaggtcgtaagaacaatatcaagtatgccgtgatgggcataaaaca
caccataggcgtctttcaatactattgaggaagaaagtctttccatttcccattcttcttgatt
cagatgggatatattgacaagttcttccatgccgtcgtttcctttaagatctagccgtatttct
ttctcatcatgcattgtccagtccttaatattaatgacaacaggcaaaagccctgccaatctct
ctaaagacgaagcagtttcttccatgttcagcttgatgttgttaatttcgccttcgataaacct
ctcattttttacgagaataacatcctcctcctcctcgaggaaattttttgttctgtcagagatgaa
aatcgtcgtgcttctcgtgctctttttttccactaatgcatttagagattttgtagatatttctt
caatagtcatctctttattcgtctctataataaacacagacgggtgtatttttacccttatttt
tgcattttctgtttcacttcctaaacgcccctcggtgaggtttgcaagggtagaatcataacac
gcaaaagttcccgcattaacaagcggtagagaaatttcgctatcgccatcttgttgttgttgtt
gttgctgtagcccttcagccaataggcaagctaaacggctattgtcttcgttgttcttaccagg
caggggccgttctattgtttgcgtttgcgttgttgacataatttcctcctcgacgtttctcgtc
agcctgtaattcgagtagagtttgtttccttatacgggtgtgaaccaatagtcggtaaaacacc
aatccaccggttacgatgatggatatcaacaatatatagaatagggccttgtcactagccttgg
cagcattgtctctttctgcagctgcagtcaaatctttgtagtctgggcctaattcgctctgtac
gtggttccgtattctctcagacacgtccatgttgttgggtggataaagtataaatactcaggtg
tacataaaaacctctcacatgatggcaggaatcggtagaagagataatagacccgtattacatc
tggacattgatccaaataaggaaataccctataacgttccaccaacccctattatctgtgaaaa
aaaccccttcgtgtttaacatgcagaagtgctcagactgtgctcctttccctccctaccccggc
actgagaagcctttccctccataccctggtactgcagtagaagaggaggagaagcaaaaggaaa
ttgaggagcttctggttgaccaatctttccctcccccattccctggaaataagctgagagatat
ccccagaacctaccctctcgaatttcccgagaagaaggagaaggatttcccttgcgttgacact
accggtcacagcgatatccccttcatcgatctggagaaaaccccaccccgtagtgacgttaggc
acggttaccactacttaatcaaccccaacaaggttggggagcttaaccatatcgttggtaagct
cactgaaaagcaagaaaacctgaacaaattggtgttggatgttgatgacgttgtgattaatctg
tcaagcactttgaaggaacttgagaagctgcgagctggcctgtgcaagttctcaaaaaactaga
cggtaaaggtatgaccaaaccggtataaccaacttttttatataagcctaatattataaatcaac

aactaacaaacgcgaaaactaacaataggaaccatggcctcatcatcatcatcccctgttgccc
tctcctctgtcgcatcatccgtgatgatggaaagagatgaagaaaatacactgtccctcaggaa
caggaatgtgaacaaaccaacacctgttagcgccgcctgggtgcctgttgatgaagaggatgaa
gatagggaggaaatgagaagacttgaagatttttcttcagatgaagaagacgatgataataaat
catgtcattgtgaccatagcgatgacgatgacgatgacgaggaggatccttcatgctttaaggg
tttttcagctggcctgtgctcttttgtgagggtttctttggcttcctcaggaagtcacttacc
aagaaacaggtgttccttcttacaagcgcagccgttgctgctattttaagactagagatgtgg
ctaaaactgaagaaggcgcagcaaccatggaagaaaattcaacagatgtgattactggaggaga
tggagatagtggtattgctgctgatgttgtctctctcgctagtgagggagagggagaaaatgga
tctcttttggaatctattgcaacaacactcatcaagactacaattgaaaatcttgtagatggtg
gagaagaaaccacagaattgtaaaatgtttttggtatacaaataaaagaatacataatttata
taatattttattgtttcctcttaaatgacaatttccttatgatacttttaggtgaaggaataaa
ggtaggagaaggtgtggaagacacaattgtagtgtttccatacacatgattctcccacatgtta
ttattgttaggtgggaatttcttaaacgtaacttgggttatacgtggagcattgttgttgttgt
tactactgctcctggttagggtgtttttcttgtgtttcatctccttcaattcctggatacactt
ctggatagatggagattgttgaggtacttctggcacgtattcgtagacatgttcttccacatta
tcatcctctccctcctcattcttgatattgaagatgggagaaggtgggtttgaaggaagcttca
gatgtcgtccgtttacatatgacacgtaatcaccgccattgcttctaggtggagatggcggcgg
tgttggcagaggaagactattgtcagaagaaaatgacgacgagcagctcgacattggcatatag
acactttcttcctctttatttgtacttgttggttgttgttgaggtgtattggcaactggcctgg
gtgtaggcggactagcataggttggaccagaaaatgttaccagttttgtgacttgaggggtaac
attggccagatcgtaagtggtagatttgagcatgggttcagagatgtccaggtccttatctgga
gtgttgaaccggacgtggctgatgaaactggattcggtgttgctcccgttcctgttcttcttat
tcttattcttctcccacggtgagcaagaaatgcaagtagtagttctgtcgatgcacttcacgag
catgttcatggccagtgcgagcccaatagtcatggagcagccgactgccaatccactcaggaaa
gtgacgaggataatgttgtcgttggtgataagattgtccatctcgagagatgtttcttcctcga
ctaccagacgagatatgatgctctggggccctgcttcgtccttttataccgtcacttggttaga
aaaaacgatagtgtagttcacagtatttgtcccttctggtcttgggattggggttctgatactc
tgcccagcctccagatataggtggatagaccgagattcttcccttgatgcagtttctattccat
ccaagatatcttcaccaaataagtccttatgcaaggacaccagaatattgaactcgtgaacgct
tgacttgttgatgatgcataattcaccttgttcgactgttatacatactccttcaatagaatcg
tatagcccggggttattggcacggtcctcttcaggtaagaaaactgcaaatattgtattaggca
caacatctggcacgtttacagaatgacagtaatgtttatctataatgtcaccagaaaagtgaaa
atctatcatccgacactggagaaaggttgaaggaaaatctttccgcatattcttgagttcccgc
aataacttcttgtttctggagtttgacttttaggattatttgttgccagacttacagcatcga
aaatactttctcccattccttcagcgggattttccatcttacttttgtagtggttttcagttca
ttaattgtgtttcagggaggctataaacaaaactcttacgcatctgttcattcagtagtggac
cagacgtcgagatgacgagacatggtgtgcttgttccaaaaggccgttctaggcatgttatcct
aggaaatgtcgactacactttctgtactactgacaataattgtgtcagtctagacatcgatttc
aaggacaatatcacagaccaaaacattcagttattgaacaagaaattgggtaagaaaacagcaa
agaaaataaagaaggaagatgcacctgaaacaaaggaaaatagtgacgaagacatatacgccac
caaggaattcgaacagacaataaaaggtctacagacaaaaaaaggtgccaccgaggaaaacgcc
atcgcggccgcagctgccgctgccactgctgctgcggtagaaaaggctatgctatcagaaagtg
aaggaaaatcaatggtcatcaacagagctagaatggtgctttctaagcgagacacgtcccagaa

acagttcactgcattgaagaacagggaatctttcttcagtgttttgatatttgaaactggatca
gtgatagttgtcgggcttcaagatccttcgcttacaaaattgtgtgtgattaaagccacgactg
atattgctgatattctacagaaaaacatcagtgtggctaacgtgtctatagtgaatacagtgtc
cacttttaatagattccacttgaactttattcgactcgggaaattcttcgaaagaaattgcatc
tcttacagttataacccagaaacgttccccggtatgtttttcaagctgcgagtgcccgcaaagc
ctctcttgcctggagagactatagggaatactacacaaaggttgcaatgatgcgcgatagtaa
ggatcccaattttaaaatgtctgactggttgaggataaaaactgcattaacatttaaagttggg
aaaattactgtgctcggagaaggagagagtgggtgcggtgatgtttctgtcgtatccaaattac
tatttggtttattccattactttatggacaacaacattaaaatgtcccccaaagaagcacaaag
agtcagagaaaaatacggcatcccgcatctagaatggtacttgtacattgacatgttgctccac
tcctacccgtacgtcaaaccatcggccgagcaagtgaaaagggcgatggtggaccaacaacata
tttctgaagtggataggacatactatggaacaaagaacagtatggacgctgccatgtctgcaaa
tttagtgccttcaaaagaagagagtatctccttcattaaaaaaataagatcacaacaacttttt
ggacatttgtgtaaaccttcaaaagaaactactcgacgtgctatagacacactttctttcgatc
ctataaaccaagacaggtggtggaataaaaatgaccaatattacggtaaagagagatgtgaccc
gttttctgttgcacgtttagtgtctgtttctgaaaatacaaacagtatgatgaatagtcgcatt
tcttgtcaagggaaatggtggctagatgaaaacgaatacaaggataaacttgatcatattgtgg
atttgtgtacagaagaaatagtggaggaatgtgaatcaaagggttttattgcctccccatttt
gaggaagcaccagaaggaaaaaataccaacgccttatgttttattagcgagagcctgtaatcaa
aaaaatggtaacaaaatgagtattaacaataatagtaactatttgtcgggttcaagtagggcga
agaggaatgcaaaactacaggaaaaacaccgtgtaactttagccaggttgaacacgatgatggc
gtcgtaccgatttttgaataattacatctcaacagacattgcacctgattttgccaagttattt
ggtaatgatgtatatagtttattacatttaatgacaaacttgcctaaatcccgtggacatgctc
taacatacaacgaaagggccctttcaagtaatgaaagtacatataaaacacctggaaatgcata
ctttagtactctatttgaaaaatccattataaataaccaagaaactgctaataaaggtaacaat
agaaaacgtaaattttctcgaatcggacaagaaaagagctcttttctgtgcaacgcgtgtggtg
tcaatttgaacaagggtagtgatgaaatcataaagggtatttgtacaagttgcgatcaaaatag
taccagttacatagagaatgcattatctgacattaacagagacaagaagattaaacgttttaaa
gcagctgcaacccatccgccagtgaagcaagaattggtagattctttatcctcctcttcatctc
cttcttcttcttcttctcagacgtctaacaagaacaatagatgcacccctagtgattttataga
ttatgtgtacaaattcactgacgaaacaacaggtgctccaaaggtgggcttagtgtttaaaatg
tgtgatattcttgcatccttagcaagcaggagagggatggaagatcgtcccacagccaactata
gaacctccttacattcagctactcaaaataaaaccaatttgaataaactattagtttctgctat
caaggaaacaggagccactgaaactgaagcacagatattcaacaagattattggtagtgaaaag
ggactatcaattctctgtcaacttgtggaaaggaggaacaaagacaataatgtcttcgactgat
ttgtctaagaatgccttccatgactgggtggtatcaaagacagattgtgaggtgtttgatgtac
actgtgagacggacagagattgtggcgctgcttgcgagaacacgtactctgttgacggaaagga
ggttacaaaattctcttgtaaccaacagtcgggaagatgtgccaggagtgtttatagtgcgtct
tctctagaaagagcagccaatgatcttggccacattataggtatcatcaagaaaaatccaaaat
tggaggaagaactccctgaatcatttttgtggtttatcaatcacaatggaggagatttgtttgt
gaataagcgagccgcgtactacgacacgatgcatctaagcatagggaaactggataatgtggac
actcttgcccagggtttagataaacggatggcctcatcattgagagagcacctactgaggaagt
tggactctatactttttacaaattgataaagttaaatatgaaaaggcaaagaaatggatattgga
tataacacaggaggctggcaccgaagaggacaataaagaagaagaagatgcgaaaaaaagaggat

caatctcttagcgttagtgaaattgtggatgttttaacgggcacacatgaccctatgcccctga
gggctagagggtttatccagaaaaaaatatatcctttgtcaagaaacgaattgagagaattagc
ccttaaggaacttttccctgaagaaactacatctcctcaggttttgagtaggcaacatgatgta
tctacgcgtgaagatttatgcaatgaaagtatgaatgcagggagggcagaatccatttttagcg
accctgattctggagagtacgtggctacttgtgcatgtctttactcggaatatttaacagggcc
tgcgtgtaagcacaaaacatacaggtatgttatagactacgacaaatggaaaaggactggaaga
cctgaatttctaactgatcctgtacttcattttaaaaaggcagaagctgtgtgtaaatcgacaa
atccaaacttgagggcaatttatagtccagataataaaggtttcttgtgtgcgcctgtagctga
acttgtaaagacggcattaacttttaggggttcacacgaaccgtctctcattgtcgagagagat
ataaatcaagctgaaaatctaccatccaattcatttggtgtaaactggccctatgtgaatctcc
taaatcgcattcaagaccagtacacgtaatttgataaaaaaagatcggggaaaatgtctgcatc
tttaatattggacgaatacctcaagaagactgcttcagccgttctggatgtagctgactcattt
gagaaaatcaagggagaaatccaatcacctgaggaggctgcggctctttctgttgctctctatg
gagcacctccaaaaccttcagcttcggctgtggcctctatcatcactggagaaagaacatcttt
aaacgacaaatatctatcggataatgtcctattgaaaatgtctgttgctcgcgttggacaagaa
aataatcgcaagagagccgaccaggcagctgatgaaattagaaccatcatggaagatattacag
ggagtttgtccggtgcgtacaggcaatatagcccgctcgaggaagaaaataaggtgcatatagg
catcatgaataacaaaacgcctagcattgtttgtggatattatacaatggacacatctatttct
tccgaacctctttctctaacagattttcaaaccccactgtcattgccaatgtgactaagcgga
tggagagcatttttttcaaaggtcgactctgctaggtctacaagattcgacgcttttgttaatgg
tgttgcgaataatatggatataaagtcatcaatagattgggcaaatatggtagaaaatgtgatc
aaattaccagattctacacctaacccttgttcagttgacactattgtgtccagagacgcaagtg
tagttaaaacagcagttaatgatatatacgcttctgttggaaaatcttattgtcgtcctgcaac
acagctaacctttatgagcgagattgaaaaactgcgaaaggctgcagttgtatgttttgaggca
ctcatgtccgatactagggagagggcattcgtagagttcctattttacgttagctttaaggaag
atgcatcaaataccaattcaaaattgtttgttcagaataagctatcttccatgtctggaaaccc
cagacagcccataaaattggtacgccgttctgctgaggaaacactattcgggctctgtttcatg
tttaaggtaatgcctccagaattcatgaactgtatatttaacttccctaccattccccattcaa
cacaataccatggtctatatggtacatgtttaacccctctacttagaaaatacggttcttcatt
cgaaaagtcctgggctcattttgaggaaattttaagcgaaagagccaatgcagtgaaaaaattt
ggtgtaaacgatacgaggatagattgtctagatgcagtagcaaatctcaccggacctgtgtatg
ttctcattttagatcttgtacgtactctaagtgcgcagagatcgtgttcaactaaatttctccg
tgaaattaaggaaaactatcttttgtggaataggtttgtgtcataataaaaatggctcagacat
caaagatgggaactaacaagaggtgttttgaggaggaagtggaggaagaaaggcaacaacctttt
cacaaagaaatctaatcggaaccacccagttttgaagacaagagttcatccacatcttctaag
aagaagagcaaatccaataaacacaccaagaccaaggaagaacaacttctagaattcgtgaagg
atctggagcggagcgaccccactgttcctgatgagaaggtcaagcaagaagttgaagaaaagtc
ccctgaagctattgctgaatttttttcaatgtttgggatcgctcaagacagcaagttcaagagc
cttcttcccattgaacgcataaagagcatcactactaaaattgttatcgatgcaattaatcagc
ctgtgcgcaagatgttggttgaccacctctatcattttaaggagatgcagaatgttgtggagaa
atataaggacgatagcgacgaaaaactgagcgtcattcttaagagtaagaaatcccccaaagaa
tttgacctctccttttccgattacgttgatcgccttaacaggattctggttggtgtaattaaga
gggtggccggagctattgaaagtaaggaattgttgcagagtaacagcatgatcatgaacagtgt
tctgggtactgttgtgtccaacattccttacaacatgaagattaatatttgtgtgtttttgact

aactttatttgtacatttgctaatgatgatttgtacacattctttagggatgatgagaaatttg
taatgagtcaggtaacaagatacatttcaaaggattagaaaaataaaagatggtataaaattac
ttgtatttttattcaaacaaaacctttatatttgtacaattcccactttacttcttcttgattt
cgtccttgatatcgatcacattcttggaggtgaatccaatgttcattgtagcttcgaaggctgc
gccgtcttcaaatttgctgcacacgtcaatgagggtaattttgaagatgaagatttaatgtcc
ttgctcgagaagtaagtgttcgacatagtatttcccttaatgtttcctgcaataacagttggcc
gaacagtaatgtcgttgatcaagagatcgacgccagtgttgttgaggatcttcaaagtcatgtt
agagttctcggccccagtgccagcgggatcagtccttggggctgtgacggtagagatgacaaga
tcagcagaaacatccttcatgtccttatcggagagaccattcttcatggccacctttccaagag
gagtattgtaggacctctctccacgaattgacattacctttgcccttctttgaattgggactcg
catcatctgatcataattagcgacgacgcttcttccaacacgtgtgttgaatacaatcattata
accatgataacgattagagcaatgattgcaatggacaagattgcaataattgcaacgtccaggt
ttgttaggttgccaaattccatttttctttgttttagatggaagttcttcctttacgtgttggt
tggatccagctagttagttcttctgtagcaccatatacccagaaagggtttgtttttaaaattt
aaccctacatcgaaccggatgaagtgtaccttgccgtgatctaatttcagtaacgatacctccc
actacccctactttcatctccatctctacccccctccctactcctcccttcacctcctcctctcc
ccctttactcgtattggtcgtcttcatcgtcgtcttctgaattggatgaagaattgtaatattt
tgtgtgcttcattcttttcacttgctttgctagagaattgacgctagatttgttattcaaaagg
tccataaaagtcctccgtctagaaggtgtggaagattcttggtcaatatacacactgctagcgc
tactcatgttggaattgatgtcatttgaaaaagaaaacgttcttttcctaccaacatgagaaga
tacactgctaggcacgtccattgctgttgctgctgctggggtggagggagaaacatcttgattt
ttagaaggacggtacattaagtttcgcataggcacaacagatgtagattttccgtgcttaccct
ttttattgtatttctcagatgcttgatttagcttatattttatgcagtacacttcaatggacaa
tttagctttctttaaaagtttgatcactactgcgcttagcaagtgtttcctcttgtccatgaaa
ttgactagtgtatttaatgcattatcaaaaataggggatctgtgttcgatgattgatgagcctg
cattgtatgctacaccaaaccttcccccgccttgtgtctggagatcaaacatactattcttgct
gttattgttactcactccagaatacgcgtcaccgaaaaagtggtttaatgatgtacggtcatta
ctgtccccgtatcttatattatcgtatactcgacgaattgcttccttcttgtccttctctgaaa
acatgttgccgtcttcacttcttttctctgcctcacttccattaccatcttcatcctcttcatc
gactgccttatcaaattgtatactgtttaagacgtaaatgggagggacagccattttagaagaa
caactagaaatactatccaaaagaagaactttataatcttttgacaaagtgaactcttttggcc
aatttaatagtacaattggtatgttcaagaaaattgttaaccttcccacgtccatacatagttt
gttgatgtgtttctctatgattgtagaaattgccccgaaacaaatttctgcccctatagaacct
aaccccatttcttccattatgtttgcagattctttactcatctgttttagtttagatgaggtag
acaaatctgatacatcatttgtttcagtttgatgtttttcaaaatcctcttgggctttgttggt
gtctccgaacagtgccactgatgccttggtcactatgtccgaaagcatattgactaatctagac
caagaaacagcacttgatgttttaaaggatatgaggttgtttaggaacgccagtctttgaccaa
gcttatacgtttgacatggttgattatcgtcgtcttctttcagtatatttatcagttccatgtg
caaatttgcttgagtttgattactataaaatgaagaatgctccaccacctctcctcctcctcct
cctcctcctcctcctcctccagcaatagactcgtcgccttcagcacatactccaccagcagatg
aagacatggaagtttcatcctcgtttaattcagatatactttcaaccttttttaagagaaggagc
aggagtcgatggtcttattttgctagctctttcccccattatataagttgacgcgtatgctgct
gaaaaaagagggttggacatgatttgcttcctctgaaacaactccaatttctcatttgttttcc
tgaacagctcggcagatttaatgtctgtcaccgcagcagaatctaaaaatgccttcatggttaa

ttctgcagcgatgatattttaaggcgctccattactttctcggatggttttactgggttttct
ggatcttggaacacaactttcaccagttcagagcattgaaggttctttgttgtttctagaagag
tttttacccttgttttaagattatagactccatagtcttgtgtaatctttcttcggcctcttt
cccacctaatgtgttgttaacctttccaaccactgtactcaaaaggatggattgttgttgttg
tttgaaaataaggactgatttgtaggtggaggtggagggggaggaagaagaatattggtaggtt
tgagttgaggaggttgatgaggtggtggtggaggtggaggaggaggaggaggcatcagcatcga
atcttggtccatgatgtctctactttcttctcccatttcattgtctggaggaaaagtcacgata
gatggaacaggcggtggtgctatatcgtttagttggtcatattgttcctcacctccactaccaa
gaatagaaaacattctaggtgcttttggtgaaggttcctttatctggggagaatatctgtctc
tttcatgattggagggaacacaactgcatcctcgcactcttgtatatctggagggggagatctt
cgaaccctgggcgcattgttaaattgatgcatagtacttatttcttcatcctcgagtgtgtgcc
gagattcatcttcatattcttcatattcttcctcttcttcaatatattccgtgtgagattgaac
agactctttagtttctacatgctcttcagattttgtgcaggttcctcctcctcctcttcctct
ccctcttcttcatcctcttcttcatcctcttcattatcattgccgccacctgctattgaagaga
agaaataatccattccatcatcattacctccttcactcatagggctggattcaactctatgaga
gtgcacgctttcatttctattgcatcttcatccttctcgggttcatttttttcttgttctata
ggttctttttcttcctcttcctcctcctgttcttcttcctcttcctcttcctcctcctgttctt
cctcttcttcatcctcttcctcttctacgggttctatttgttggttatcatcttctttgttttt
ctgttcttcacctgctgatccgcccatggcgttcacattttcaagtaaaaagttcaacatggga
agaacttgcctatttggccctactggcgagggggtgttcactatacttttccactggttttcag
gtataattgtcgcatcttcttgttgtgccgctagcgccaataacgcagggtcagcctcatcttc
ttccccttcaccttcattgtcatcttcatattcatctccttcttcttcctcgtcttctagattt
ccgtcatattcacccccttcttctccctcttcatcttcctcttcctcctcttcctcttcttctt
catcatcatcatcgtacatgtcattagtgacatctctcacaaatttactttgtttttttagaaga
tggaggatctttatttcttccatttctaactcccgtgtcctcatcttcatcttcgtaatcctcc
tcctcctcctcctcctcatcctcgttcatttcctcctcatcctcttcgtcttcatacaagttac
tataatcaacattattattgatatcgtcctcgttgtcgatatccaacactcccgcaccttcagt
tatacgcagatttgatgccgcttccatgatgaaattttagtatacaatattgtgttaaccgtgg
aaaatcaaacactacatttactaagttcctttcctgaaaaaacgagttacctctatagggtaat
tctcagggtgtatattctatagtttggtcttgaacgtgcttttggtttttgttataactttctc
taaaatctactaacggcaagagcattgacagttgagtgggaagggggtggttcagagtaagacca
aaaataaaatatttttttgattgtgtgaatttcgaaccgtttatattactattttagggtactag
aaaacgttgtcagaaccaatcatgaggtttatcccgatgttgataattgcgcttatcgctgcat
ttgttattgcagctcttcttactgccaattcaaattacctggatcacaatatcaataaggaact
aaatttgacccgttcccttcaacttcggggaacattcacaccagaagatatagctcataacaac
agaattctcccttccaagctgagtgttttagaaagagggtcaatcattttagcagaaatggaca
agtacaaaaatgcacaacctacagtaaataattctcaagagaggaggaacatttctactcctca
acaacaacaacagaccacccccctcatcccaatcatcttcccaagttgaactttaaattctataa
gaaaatgctgatttatgttcctgtatgtgacgagtctcgcaccgacgagaaagaagattttaac
aacgatgaggaagaaaatatattaaaggaggaagaagttagtggaggagaaaaggttgtaaata
tcgtagtagggagtattaagatgatgaaacctggttcaaaaatcaaaacaaagtacaggctcta
ctccctccttgcaagtgcgatagggagtagaaagaaagctgaagaatatattgaacgtctatac
aattcctttttcttccatgacgttgaatgaaggaggacgtctggcttcagcgatttattgtccat
cctacaacaataaaaggataaaaaacaatcgttctaggcctgtgaagctgatccatgcctccag

ggaattgttatctgaaactacggtgagggaggaaatactacggaaatctcccgcttcttcttct
tcttcttcaacgtcatcatccacttcttcgttttcttcaatttttttgtttgtaccttctaatt
gtacttctaaaactgtatgcgattttgtaaaacacatacagtatgaagaagatattaaccgttt
aaggtataacataatacatatatctgaagaaaactatgcttcaagattttctaaaattaataga
ttaactacatgtatacaaggtataagtaagacaataaagattctataaaaatcaacatttattt
gtatcatttataaaattccatatttttgtatccatttcgtagtcgacgggtagagatggtctgt
agaaagaagatgctgctccttttaaaaggttattggacatggctatcacatacatttcttatt
gttttccctattgatgatatttgtcatgacgttgggagtggcatttttttgttgctggtgtatga
caaacggcaacattactgttgttcttaaccacctgagcaaacatggctgtaggggattatcttt
ctatgtcctctgtaggggaagcaacgctcgtgggttttatgattttaaattttataaactttgt
cacaatattatctttaataatatatgccgtgacagacgtttatagaagatgcaagcggccgtct
accaatggatattctggttgtacaaccaatgtggtttcttcgactttacaagaagctaatctcg
ttaccactgaaaaagataaacctgttcaatttgttagaggtttagtccccagaaaaatgatgga
aaaatatagatcggacttgtctcctaagaacgtgggggaatatattttaccttcagaaaaagaa
acagacaaattgaaaagtgattataaaaagggtaagaaggttggtcttttaactgccctgagta
atggtcatgacagtaacaagaggattatagggccaagggatctgattagtagagatgatgtgaa
ggacaaaagttatgtctttaagagattgagcaaagatccgctcgtctactactcttctgcaacc
tctaaatacgttagaaaattttcccctttcagagcaaaaaaattcatgacatcaacacagttgg
ggagtaagctcgtgtatcctcaccctatacggtatggtactgcttttgtactacccacgggata
cgtgatcaacaaagcatacggaatggataatgaggatctacacacttggaacccaccctcttct
tctgtgctcgtcccagactctaataatgatagattaacagtagaatgtgctaaaacggacccaa
cacatagaatcggcatctacggctttggagggtctgatgataatagacgtgcaaaagaagaagg
ttatgtagaaatgttgttatgtaattgtgacaaccacaaggacttgcttaaggctcctctaatt
acagagtattctacaaatccaactgaaattcaagtagatgttgctgcaaaacgtgttttattcc
ctgcccctggttccgagcctgtaaaatcttcccaagtgacatctgctgctcatcaactagacgg
agctactggcgagcacgatatttcccatgagcccgtgaagctatcagatacgggtgactatgca
gttggatcacccattgtattcaagccagtttatggtacatctttagtaaatcttccagaaacag
gatctcctctggcattgaactgcccctgcaccgacaaggctgatggaatatatcaagtcaatca
aaagggagggatattatatagagatatggtggggtatcttaacgccaaccctgtggaagctgca
tcactttcttcctcggactcttcttcgtggttgacaactggtaacaaaatatcttctgttacat
gtgaaggagaaaaaataaagaaaattgtgtaatcatatgtgtagttttattaccactaaattta
ttggtcttcttggtactgtttacactagggaattttactcttcctccatctcaaaaacttttca
aaaatttttctgggtcgctcgagtttagagggtggaccgctgggtcggtctgatgtcaagtttg
gagggtggaccgctgggtcgagccaatgtcagattgcaccagaaatgacagagctccagaaacg
tacattaactttatactgtttctggaacatgtatttctggagactgacacgtccagaaagggta
gggcatagtctactgttttcactagggaattttactctgcctccaccttaaaaacttttcaaaa
atttttctgggtcgctcgagtttagagggtggaccgctgggtcggtctaatgtcaagttcggag
ggtggaccgctgggtcgagccaatgtcagattacaccagaaacaacagagctccagaaacgtac
attaactctaaactgtttctggaacatgtatttctggacacggacgcacccagaaagggaaggc
tataacacactaaatacactagagaattttactcttcctccatctcaaaaacttttcaaaaatt
tttctgggtcgctcgagtttagagggtggaccgctgggtcggtctgatgtcaagttcggagggt
ggacctctgggtcgacccgatgtcagattacaccagaaatagcagagctacagaaacgtacatt
aacttttacgtgtttctggaatagacgtttctggtcatggacgcacccagaaagggaagggtgt
aacctactgttttcgctagagaattttactcttcctccatctcaaaaacttttcaaaaatttt

ctgggtcgctcgagtttagggggtggaccgctcactcggtctaatgtcaagttcggagggtgga
ccgctgggtcgagccaatgtcagattacaccagaaacgtttgttgctccagaaacgataacaca
catttctggagcagtagttagttatttctggagcacgttaaaataagtagggagaataatacat
aacattatattttcaattccattttattggttctatccatctaaaggtgttagaggaaatactt
tcattgtagtataagaatccttactgaaatttttcaaatcatccaaagagaatacaatctttcc
agtataacttagagggttccttggtgtagctgttttcgtgtccctggcgagacggtacttatta
ttgcttcccatctttaaatcttcgcttctactcctcttcatagacctgaataaggctatggatg
aaataggggtctttcctacaggttcgacaataagtcttcctatagatgcgttccgtatcaagtc
ttgcttggccttcttcacattcctgtcggatgcggctttactcccactagttttccatatctct
gattccgtttgcatctctctaaatgctttccagatcaatttttctctgttaacattttccactt
tgacataattattcttcaacatgttatccactaacatcttggatgaaaatgcagataagagagg
taagggcctccctggtacaaaactagtgtttgacgcatcaatcacatttcttcgtgctctaaca
gcattccttaaagcccaaaagcttgcaaaggataataatgatgatgtattttcatctgttggaa
catttccccataagttatgtgtaggaaacttcaaatctccccatcttgccaatagtaaatatac
tgctctgtcgaaattgttcgttattgaaggattggaggtggctgcagccgcgtacaacaggtag
aaagaaagggcagcaaaccctttagatacatggacatattttgcagcttcaaacacataccctt
tcttatccactaggtgcatgttacgcacttcttcgtcctctctttttttgtttcatttttccttc
aaaacctatatcaccacctaaccctcccccttcttaatagacccgaatctgcagagagtatctca
accatcaactcccctaacccattgacattttctgctcctttattattaccaccaccaccaccta
aagcatcgagtactgctttccagtacgtctctagttcctgttctcccatagacggtttactttg
ttcctttaaactgatggtaaaagtgtctagagattttgttatgaaaccagattcacaacgctgg
cagacgaataaagaatgcatatctttcaaaaacacttgaagaattaacctgcttttcactgcag
agctattagttttctgtgttagccctccagagcataaaaataatgcattccatagcacatacat
tgaaaatcgggcagctgagtcgggatcaagaacgcgggtaacagagtagttggagaacataact
tgtctattattgacaagaaaatactccaaagtttctggtgaaacaatatcgttaaacttgttac
atctcttgtattcacgttgaagagcttgaagggcggtaggtgtgcgtttaggccctaaaattcc
gtctatcgcctttggaagatatgggtggtccctaaagagattctcagcagtcatatccgtagtt
ttaattcgttctctttccctctcgacccattctttttccctttctgtgacactttcggatggca
tctgcagatggggattgttcacatatttatccagtttagttgtcaaaggatttggtttagcgtt
tctggtcctggacagcactgccctgtactctgcgtcgatggctgctgataatagtaccatgatt
tccctccctaaaagagcttcatggaaagaacctaccttgggggggtagaatccttcctgtatta
cagaaaccatgttggttaaaacagtcgatatagttggaacaacataagattgtccatcttcttc
actgactgtaactccatcactaaaggacccttcaataaagtcttttatcctttttttcaatcatg
tcagcagcgcgtccgtactctaggtgaggaggcatggcagcaaagggatctaaacccctctctt
tcattctagaggcgatagattgcttccatcttagaatatcacaaagagtatatcctagaaatag
agcaatggctctttttgctcgtcccattgccagatcggccaccgttgaattgtttctgttggcc
aacatttctgaatcaattacccatgaaggttcattagtatcacctctagtggaataaagaacta
gattagctgctgatctaaacacaccgtccataatattctcaaaggcattcaaaaggttcaataa
tggtgcttgtttcaaaaaagtaaagtcacatagttcttccgctgcagtttcggtcaatctccaa
gaggggtcaattatatgctctctaacaccttctatatctctgtagacactcctcatgacggcat
ctttagataggtgtcctttggctaatcttagtcccgaattaaaaaattctgtcgttttattgaa
ccacgtgggtccgaatacgtcagaagaaataacaaccttcctttgtttttcaggtgtattttgt
aaatttgaaatggcagcaactagcccttccgatacataatttagtcgtttttcatcggttttag
tttctgctactaatgccagaacatagtcctttgactctgtcatgatttttgtggtgctggtaaa

gtagttttgtacacgctgcaatatcttcgaccttaaatgtcttcggttaaaggatatttatt
tgttggtactgttttcgtcaaggctgaaaaataatttcaaatatacaatggagtacatggaaga
aggagacatcgctgaaaggcgctcagaaggtgtcgactatattctggacgaaaactctgcttgt
gtagttaatgtgaagagtatccgtaacaggctcggtgccatggacgccgaggaggcacagtacg
cacaggacatttccgcccaacttgtcacccatattatccgtctggcccactgctccgaatccaa
caagattaaggataccattgccagtattgcgggtcttttcatcaacaacatctttgacaacaat
tcaacaaagaacaaacttaaaacgtataatcaattcaaggcagagtcacaaaacaagtctagcg
ttctcaatatctttggctctctagatcctctgagtatgctttctagcttcatgggttctgatcc
agcaaagagtggaggggaaaatttggacaaatctttgggtgtgctctttgaggtgcttcaaaat
tacaacccttgcaagattgacgatattgtccttctggaaatgtgcccatccaagtgcgccgcct
gcaccggtctcaaggaagccatccgccaggaacaacccatggaagcaatgttgttgtttttcaa
atgtatcaaccataataggttcaattttggaagcgacataaagtcagcatacgcctctgaaaca
tgcatgagatactctcaggacgaacgcgcagtcgttgtgcctctgaggagtatcctcctcggct
gcctcgacaggatgatccagctcatactctttcctctttcggggatactatcgagtatgctga
ttcagataacgcttgggtttcttccctgtttgcagccgtctctagaatgcctatggtagacaga
gccgttattgctcacttttacgtgtacacaatgctcagccgacataggcgagtatctggagaca
gcttcaagcagtttgtctataccgtatttgttcgtatgatttactctgcgattgaaattttgtt
ctgtgatactgaaaattcgtctgtagaatgtgatggaaagcacttttttgagctatgttaatgcc
atggttaacgtatccgtgctgggttctacgtttaacgtactaaaagcctaccgttcatgggtgg
tggatcaagcatccgtcgcaccggttctagacattatttccggaggatggaagaagaactaccc
ctcacctgaccacatcaagagggtggcgtacgacatctctcaagtcatcaatcatcttgcatca
ccttctagaatggttaaaggtaacaacaaggctagcaacgttacatctggcctggatagtatca
ggtctgttcgtcaagcagaaaaatatatcccgtttggaatacttgaaaataaagcagggtatgg
tgtaataaacattgccaagcacaacatcagccgtccagcaagagaacaatccaacggaaggaac
tttaattgcaacgctttacacattctaccttcaattaagggctgtgaggcacttggggcacaaa
aggggagcgcagatcaaactgtcaatgtttttgataattttgtcgcatctcatatggatattgc
catgaaaaagcaggggtcggggaagattcttggactgctcactagcatgattgacaggcaaggt
ctgactacttcattccctagtagtgaagcggaatacaagaagagaatccatgatttcacaagat
acgtcatcttctcttcaacacccatcaacgacgaactagtcaattctcgctgtattcttcccca
ttctaatgttctgaactcccctatcagcttgagaaatattgacccagaatcagtccccgacact
cgattccactttctgctcatgatgtggcagcggccaaatatcgatgaacctaatctttctgctc
taactacaagtcagctagaattgttgcttagcaagaaccaaaaatgggacaaactcaccaccag
agcgttcttcaatatcgacaggatcaatttccagatggcagacgctatcattaagaacgtttct
ggaagcggcttcctagatgggagtaaaactgcctcttcttcttcctcagcgcctaacttttcc
aaatcttcagtggtgctgaatgcactgcaaagcagctccaaagtattcgcaaattcattggaga
atctatgcagcatgtacaaaaggaatggagtagtgcagtaaacaatgggaacagaggagtagaa
aattatgacggactcaatgctcagttctctgaagaactgttcgagctgctctacaaattgatca
tcgaggaggatatgcggccatccagcctgatcgcctcatctgaattcttgagcaactacgtcaa
cgccatggatgaacttcttatcagagctaatgcttcttagatagggttttatgaaataaaaca
aatataaattatatatcatgcaattttatttataccttttttgtatgtgatacaatatttta
gtgttatgacagtgcaaccattatattattatctgtaataggtcttggtaaattttaatgaaa
ggaagattaggtgcacaatctattgtcataaaaatgtccaggtcaatttcttcaacaccttcct
caaagttatagaagaaacttgtttcatcattttattagcaataaacaagtacgtttttcctt
agctgtgcttgcagagcttatccaatagtctgatgcctttttaacattataacagttggggagt

aaactacaaaaacagtcctctgttttgtttgtagcgtatacccgtacacggttaccttctacta
caaatgaaaactctatccgggctttttcaatacgtcctcccacagctgctctaccttgttttttg
tagcacgggttctaatccaacttgtagagaatccagattcgttgctgcactaataacattaaat
gtcctctgtttcatgttgacatgtacagacattcgtccttgccaagtgcgcatatcacgatcac
tgataaaacctcctcgtatattatttatcaccaattcctttgagtaccgggcaaggtaatcagg
tgtaggttgaggaggttgttgttgtctagctagttgtgaagaaggtgttgttgtcaacatcacc
atttctgcgtctgggtctgttgatctctgtaggctttcatccccattttcatcagaaggatcaa
ctgttactgtaccaaacaataaaatattggtcaagtctattacgagttgttcgtaagtgatatt
ggtagattttgtgcaacaacttcatccatcgtttccttgacttcgtcatggttcgtccctatg
agatcgaacaagttttcatcagcaattgcctgtccgacatcggggacgtttatgtcaccttcag
ggattgaaacttttccatctgttattttatcctttattaaccatacgttttctgatttgtatgt
gtcaaattcctgatctgctaatttggtgaagaattcatcctcagcttttttcaagagttcttgca
aaatgctctctcgtttgacgttgaagcgctgtctcattgataacatcaactgttcctccccctc
ctcctccattattattattatcgtcgtcagtttttttcatcatcgtcagtgtatttagagtctaa
tttatatttcttactctttttttgttttggcatttgaatgtagagttcccttccctacgcccgag
atagcaaagaagagggcaatagaagctcctattaccaaaacaataaccgcaaaaacaatgtata
ttcctccattgtttgaaagtgccattttttaaacccttcaattcttatctaaaaaacaaacagat
tgaaatctatgtataaatcatcactcgcgttttcgatttcaggcatgtgaattgtaatgagaga
ggccgaatcctcggatgggttgattatgccatttgtatcatgactaatatatataggatcattt
ctctttagataatcacctatgttatcagaagaatcactatttcttagatttgataatatcacta
cacttttttggtctgtttacaataccagcgaacgtggatttgcggttaaattctaacgtgagagt
tttggctgaagaatctattttgaacgataaacttaaatcaattaaatccccttgagcagctcca
gggggagtgattgataacatgtgtttatcgtttattggtacactaaatgaagttgaagagtcgg
taaaggcaatagtattgtacttgaaagaaaatgatgtatgataaaaaccaggtatatatcttct
ccaccgctcctcttttaaaatttcagttatgaaatcttccctgggttccatgatccgtctattc
acatcgtatactttttccataaccactggtattcctttctcaaaaattgcttcacagataacag
gattgaaagcgtcttcgtaaagatgttcaaaagtcattacattagttaaatggctattgatttc
ttgccttacttgatcctctatatattttatagaggcatttttttgctaccgaataggcaattgca
cctaagcgatactttaattggtcactctttcttgtgatccaagaattgtcatggtgagcagctc
ccaattgagcctccactgaagcgatttcagcttcagtcacagcaggctcatctccgttagacga
cattatttactcctccttaaaagcagtgattgcaaacaatatatcgtcccttgtatcgcctctt
tttatacgtttaacagcgtatgttgcagattctccgtttagtatatgggtgtgtttatatctag
atttacatacaatatcaatatctttactcttcccttcaacaatcacattaaagaatatgactac
gcgttctcctgttcgattaaatgcttcaaagcttagcacgtcattaactacgtcactacgagac
acaagaatcactgcatctacatcctcatcatttatggaacgaaacaattttgccgttattttaa
aatctcccaagggaatggagaaatcgtaccttgtaaattctccatcgtcaccaccaattctcaa
attgtacgtagcaacactacaccgccctacttgaccagtttcttgttgaatttttttcgtctata
tctgtgcaagatattgggggccgataccaatgtacaagattttgcgttagttttcgtttagcca
agtaatcagttattatttgtctaacatctattccttgtttttcaattgcctttaaaacacaatc
ctttttttatgtcttctaggcgagatctcacatccagttctgcatccttcatgtaaactctgtct
ccatgtcttacagtgttcttcaacaattcgtttatgtgtttggttatggggtgtatcaacaatt
cagatagattagaaacaccacgtctttctacaattttatcaaggatgaggatcaacctctcatc
gcttatagttgcgccattagacatgctgttgctacttgcttttggtatacaacatcaacaggtg
taaaaaacatacaaaagaaataataaaaatactttagggaagaaaatgtttagaagtattttc

aacctttattgttctggggcgaagattatatacattcaatcttttaacgtagacctttcttcg
tcttcttcttactcctacttctttttctcctcctttctggtcctggaactggaactggggctcg
gtaactggagaagttgtgttggtggtagtgacagatattacctcctctattttggtatcctga
gtacaattttggttttggttttaattgtactgtagagggagttttctgtgttacccttatagc
ctgtttcaggaacaagggtaggtgtctatacccaagggtagccattgctcaagtgatgctccac
cccgtgcttttccccttatatacaatttctcacgtggtcaataaaggaaacacaaaaaatatt
catcaattcttttatttgatcaaagaacatgttttacacagtttgggcacagataatcagggtt
aagtctcaaaccccattggttcattacaatgtacttgacaatatcagaatggtcagattttga
gaagcattaggaagagctaaattggtagagtgtctaagagcatgcaacggtttattcacaacca
ttgccagaaacatggaacagatagtgaagaaacttgattctcatccgcaatatcctctgtatt
tgccctcttgtgggatgaagaggacaaaaacacagggtatttcagattactaaccatcaattgg
tacaccatacaggctaacattacagcaacataatcaacaccagttttatcactctttagtgttt
ttaatgaggaaagtctcctcttacattcagtatactggagagccatttcaaacaacatgtgtaa
atggtggcaagaatacatcaaatccctaacatccattgaaccccaggcatcacacaaatgtttt
gttagtgaagaaccaactcctattaattccccttccttatcatatcctacaacatccatatcag
gatttggtaaactcataccaatacaattgtcaaaattgatggctggcctattctttgtggcagg
agagtgcatcttgatgttaaaatctctcccaaagaatcttgtggtcttaacaaagggcatttct
acaaataaaactgaattgagcatgctatttagttgagcccatgcgccttgtctagaacgagcaa
tattcatagccattgtattcaaagatttaggattaatctggcacatttgaagaggtgatggtaa
tttattgcacatggactcgtaatttgctggtttcttacggcgcaattgttgttcctcagcatca
caactattgtcttcttctacttcagtgatagtagcagaagaagaagaagattctccgtaatttg
ttgcagccttagcacaaagactcattgaagtgtacaaatggaaaaaggtgttagtattctcctc
taaacggaaactgattttacgtctctcaattctaggacctggtttctgatcaagtttttcatt
aaaactgatggaagaacggatgatacgtcactaattctatccttctttttgttgtcactggaat
gcccactaaagtcattccatgctttcttgtgatgattaaattcgtgttgacgctgttttattcc
atgctttccaaatttgatacatctttccatcattaaagatgaatggatatacttcattcctcta
tccaaaagcccgataattttcttaacatcctcattcttattctcaaagacaaatgttgtatcac
aaggaaaaacaataagtcttttagacatagcttcgtcgacaaagaatgagaagatgtaactgtt
ggacatcataataggagacacagtaatgctgctttttgcgccctgttggaaaatgcctctaatc
atgacgctgttttggggtgaagtcatattctttaataaactggagggaaattggcccggaagca
tatgtttggttatagaactggacacatcattcttttgggggatggccttagcgatttcggtaat
aatggaagaagatccgtcactgttatgtttttggagtgcagacaccaattgccttcttttaggc
gtatcgacattgaaacaagcctctttattactgtctcctccaccactagagttagaagcaaagg
aaaattgtgaactttgttgcctattttctccaaaaacacttaaaaagtttgcaatatcactgct
gttatcgtctgttttaggtaaagaagacgtatttttttgatgaattagaggaagagtcgtccttt
tcgtcagaaagcaaatttttatccatacacatgcgaataaaggatccagaattcttagccgaca
tcttatccttctcaatacgcttctttaagcaagaaaggacattttcgcagtcagaagtggaagt
tgacagaaccactgaaccatcagaagtaaatccaggaaaactatattttgacggtttcttaacc
acatcgaataaagatgcgtcataacgttcgtacatgttcataaagtccagatctctatccacta
atagttttgcagcttctgggtcctttattatggacgataacttgcgagaaatagtcttgatgtt
acgaatactgtcaatattaatattcgacagagtagggcccagaagagccttctcggcagcatca
tcgacagctacaaagtgttgtttcttgggaggggaagtaaaactgggtagtttgtttttgaaa
gatcatcagaaatttttccagtgtaaacgcaatggtacgcaatcaatacaccagggggcggatgt
attaccatcaataactgacttttttaacgcccccgttagtcgacaggcaattatcccccaggtat

tttttaatttcgagcatgtcctgggcagacaaagatgacgtgatcatctcggaaacctttcag
tggcagggttgcagactaaaacaggcgaaccgtcgcgatcaaatgatgtattccatttcaaaag
gccattgtttttctgtattatgttcataatcgcatcaactttgtcttcaattttcatattctgc
ttatttttcttgttcttgctcataaatttctcattccattcatctatatcttctttacaatcgt
tccaggaatgaagattattaacctggcagttttcatcaataatggattcaaatgattcgttatc
gcatattttggatccaaatttacctaatatgcacttcaaggcagtttttacttcggatttagag
aatttgccttcatattcgataggtttcttacagtgcctgtacacagcatcggtttcgttaagcc
aaggagaatctgtgcagtctttaatagagaaaggcatggcgcctctatggagcatattatacac
aatatttttctctttaaaggtagttgaaacaacaccagaagaacaattccaccagtgagctttt
ctggcaaagaaagaaatattgataggatcctttatgcggacaccatttttcgtcatttttaccgt
cgtttctctcaatttcctcacattctaccccttcatcttcaatttcgccacctcttttacgttt
ttttgtatcattacaagcatttttcctattcttttcgtgtttgagttcagaaataaaatcgtac
tcttggtcgcatacttttagttttgattcggtcattaaacggttaatattactaaaaacgatct
tagagtttcctccgtcaccgtacgcgtttcttgcttcattgatgtcaacactattcatttctcc
cccactgccgccaaaatcgacattaccacttctggaagcagactcgtccttatcgtcttcgctc
gatgacttctttgagccaaatgaaccttggtggccaacttcgtcaataatcacgataccctttt
tgccagcaaaggaagccaaatttgcttgagtagatgaagaagaatccccagcattattgagagc
cgtaacgttaatatttgtaaacctttcgaaccaagtcctcttacacaaatcgacaagagtggtc
tttaaattgtttccctttgttcccacaaatacaacagcagtttttgtggcaatattcaaggcaa
cggaacaaagagtttgaaccatgtggtgggctttctctccatctcctgaagcaatggctgtgca
catacctccaaagttcattaggccacgtaaaagatacacgacatccattctgggcatgccagtt
ttttggtcaacacatgagatgtaacattttcttagcgcatgaagggctgtagttgtagaattaa
catcatcctttttgcttcctcttcttattactactactactactactactatgttttgaagaaga
agaggaggaggaggaggaggaggaggaagaagaagccgtttcacaatcgctagtggctttagca
agttcgtataaaattgataaaaatatataatcttcatcagcaataaaaattgatggaggtttta
gagaagaactgaatgctgtttttgatacaggttcgtactttacaacgctagtattttccgtgtt
ttcttcgttctcatcctcatcttcttcattttcgtcatcatcatcctcattttcatcggtttct
tcctgatcttcagccatagaccaaaagtttttgttcatattactgacagagtcggtatttttca
tataagatacgtgttcgattaaacaaagtcctctacccattgtagtgccttccatggacgaagc
tagagcagcagaaataaattcggttgagaataaagaattcaaaataacgtgtttttctgccttt
aatttacaccttgtatacatgtccgtgaccaaatttatcccttcaaggcgtcctcctagaagat
aagtaccatcaatcaaaaagtacctgttattatcaacgctaatacaggcagtgttttcagctaa
cctgaaggaattgctgtaccattactaataaacgttctagcatttcccatttttttcaacttca
gccataattttagaaatgagagcagttcctgcatttgtaatacaaagacgtatattttttggctc
ttttgagcatattcttggcaggacacactggattagctaaaaatgagcatttgtatgcttgttc
cgaaaaaatattctgcttgttgtagtctgacaaaaaatcgggacagtatctatcgtggaacaac
tcgccttgttctgactcattgtcaaacattgaaggagttaatcgctcgctgaaacatcccccgt
ttctaatttggagagtagactcgatcgccttcttgttattcttaccctttgatgatgaaacaat
acaggcccaatcagtaaagaatatgcgggcaaatactcctcctgtgcctttgttcaataagaaa
tcgcaacacttcttaaagatttttgttcctctaggagcgacaaatttaccctgcacaggggagt
taaagaaggaattatccaagtagaaacccttatctgttcctgctaaaaagttaaaattatcctt
cttaatggccagactagattcgtccagacttttcccattaatgacagaccgcatagaaattaac
atcctcttagcatccattgaatcttcttgcagaaggcaagttttatctgtaccaccttgatcgt
catcatcatcttcatcgtctgcgcagccagtatcaatgccagcatatagaccagaatggtcctt

attgtaattgatgacacgaagcacgcgcttatggccatcagagttggtacttaaaaacacggca
gttttaaggatactgacaatttcaatttctgatgaaacaaagttgggacatttcttagtttctt
ctttaccaatgaaacagtaatcattgtcttgttgccgataacaagatttgttgaaaaaatctcc
tcctcgtcttatatttgtccctggtgtaaacaacactgaacaactattagtgatccaagacgta
cagaaattaccctcgggatctaaacatgaagacaaaacttcacgggcgtaaacagccttagcca
aactagctgcagaaatttttttcgggagaaggatcagaggaattattgtctttagatttagcaga
gaaagtgcggtgcagattcataagagggaaagtgtccttcagtagttggccatagtcttccaca
ctcaggaaaccagaatggtatactgaaagtggagggtttgaatcggtaacgccacatggagcat
tattctttgtcatttcaaagtggctattctttgaactattgggatgaatacatccccagttaca
caaaacgctaatgtgacgctcgtacaatttaatccacaaaatgtttagatttttaaacagcaat
tttttaagcaaaactatctggttcttaggggacataccattgttacagttccacatcttaccca
aacttttataggtgttgatattggatatttttttggtgggtaaagtattgctgggagaaagtctg
gtcaaatgaagattggttagaagagcccaagataacgtacgcgtctttagaatcgttaacaatc
ttaatgttttctaatttttcagtggaagtagttagagaatcatcataaagagacaataaatctt
catcataacccataggaacattctttaaagagtctttttttctccaaatcttgaattgaaacata
tttcaccgatgaattactactgctattagagttattgttaaagttcttccagggtctctttcca
aacttctttttttgacttgtcctcatctcccagggccatattcttaatgtaacaaattggcataa
cttgagaccagtacgagtcagaaatatgattggagaaatagcgtgtcaggacctttgtttcagc
aaaacctttccccttgtactggataaaagttctcaagggacggccaactattctaccctcatgg
cctgtatttgccaaaacactgccctttccttgcttcttttccgaagaattgacaactttcttgt
agaagttaaaccaaaacgagcaatcacttccttcatgtttcaattggtcttcagaaatttcatt
ggtctggttagcttcattattattattagagagaaggtgacctgatgaggatgatccacccccca
cacccaccatttcctccctcgccaacagaagacatgtaatcgccaggacatatgatgttcatat
cataaacgcttccagtatcagtcaaatcgcggcactcttttcttatcttgttagtttcttcctc
aactgccttattccacctttcaatggcatccattccagcacttaacgcagtttcagtgacaccg
tacaaagcatactctttttttcgagaaaggaacacaaatcttaaaaaagttctcaatgtcagaaa
ttactttcacaatgtcattatttatctgattttgtgtgatcatgttcttaattcggttaagtac
atagccatcgtacgaaaacaagccactttctctgtcttcttcgtttctggcgtccatgtgtatc
tggctgaaacccgtgcgggatctcgagtcctcatcaactacggatgatgatccccgactagaat
catcattgtaaaaaccacccgcgttattaaaacctcctccgccagatttctttgatcgtcccct
attacttccaccctttcctgtcatttgttttgcaccactattatcctccttgttaatctcttct
agagaaggatcgataaatgcttcttcgttagcgtccattactttagcttgaccggcaggaatgt
tgtggaaagaaatgttagtgttgatgattctagaggacttttctacagcagcgagttgggcagt
tgtacaggaattactaaccagattgtagttagtatcgattctagtctcttgcagttcttggcga
gtcttggcgcggaactcgtccaaagatttaacactgacatcattaacgtccaaaatacaacaat
ccaacaaatcatttgaagaagcacgcacagagtctgccgcttttactaaaacaagccttgcaat
acgtttcattaccttcccaacacgtttttcatacatatcattgctttttttcgctatttttgtcg
gccaaagaggaaacactaaccaaattcttaactagtgaaaggggcctagaaagaagcctgcttc
gccgtcccattaggacgtgagaaaatctataggtgataaattgcaaccaattactagtatcaaa
tccattaaaatcaaacgacatgagaatctttcgggtcatttcttcaatgacaatatcattactt
gcaacttgtccaataacatatgcggcaaaacggtaaaagtggagaggatcttcttcaatatcgt
aaaatctaccttcttttatgctagcgtcggccttttttgggagaatctcccttgcctgtacagct
gcaccacttgctagtgattttaaagcatcgaatgagtggtaccattttatccacagatagacgc
aaaggagcagttgcaatttctcgcacactttctccctgcatcatgatacgatacatagcgacca

ttgctagcacacaagagagggacatttctgtcttgagaacctcttctgttgcagccatccactc
tgcccgactgcgcaaagtagacttgtttcgtggataatattcgtcttcaacccaaatttgatgc
ctaacatcattgctaggggtgggttttatcttcaatttcttaattacgttgttatttgttagca
agttgcttgaatttatgaaattttcaacagatctaatgtcgttgtctttgagagcaaactcata
ggccttggcgacctgcgctttccagtccttattgtcgtcattcaaaagacattcccacgtatca
gcatccaaatttgtgtccagatctttaccaaaaaatgccctcgcgctaacgcccagaaattctg
atagctcaaatacggcgccagattcgtccacatttgacacacctgcctttaaatttagcatgtc
actaccgtcccaatccttaactaaacttccccaatcaataggaaggctgttattaaagcactcc
ctaacagtctctagaattgtccacagtctagaagctggactattggttccagaaccgtatcttg
ttatcaaatttgaaaagaactcagaatcattacttgtttttccattcaaagctgtacgcatgat
acattcttccatcattttaaagcactcgtatatgttaagttttgaaacgttatggtccttgaaa
taatttgctgcatctaaacacatctgttgacgtttttctttaagttccttctcagaaagcgatt
gattaaacactttagttggttttagaggatcaacaactgtagaacccagcacaacagactctaa
atcttgattcgcacacaatgcccaaataccagaaaagaaaccattattaacactcttatctgat
acaccatcacctcccgatgaacatgccaatctggctaaacttctaggtctaaacgtgttaatga
gtttgttatcttcactgatcacattttcatagatacccaaaggtaatgctgaagatagacattt
tcctgccaatttacactgttttatggcatcccacctcttattatctaaaatagacctaatatta
ccattaactttgtcctttttcacaaatcctaaagctgcagtaacatcactaaagtatctccttt
cacatgccattgtgttgaaacggagagctacagtcagatctttcagatgtttagaactaggaat
tgggagtagttttatgcacttttcgttagacatggtattgttactactaaggggagaatcgctc
ttgaagttattaagagaagcacaagaacaaaagggagggatattgttgttgctgttagcggttc
cgtcttcactattgacattaggcttgacaatttctcgtacaggcatagtaaaaggcactaccca
attcctcattctcaaagatcttctattaatttctttcaaaatatcaccagattcagctgcagaa
ttgtccaatgttagtacatcacacaaatttttctccatttcctccacaatgacagttgaaacat
acttttggcgatgacataaactggatctaaagttgaatgttttgatgaaattaacatccctaaa
cacatcatcttgtacgttattaatactactagcattgtcagatggtacattttcatcatcacta
gaaccaaattcgcctagaaaaccataacaatcatcttcatcatcaccaccataataatcacctg
ttgcacctcccttggcagctccataattctcctcatcgtcactctcttcatcactttcttcatc
gctttcgttactggccacattgttcaggatctggcgagcagaaacgtcgtcgttgtcacatttt
cttagatattcttgtttttgttgatagagtttctccctcttctccgacttcttgtctgccaaaa
cagcttccttatcagacttgacgctggcaaaaagtcgttggaaagtctccatgggggcgcattc
atacattggtccaaaagacttgtcagattcatcataatcaaacgttttaaatgtatcgttgttc
aattcgtagaccattgtgctttcatcatacttaattttgcacaatttgctgtttgtcttgttct
tgatcctctccatggaattttcgtatgctgcaactcccattctgcagacagattgcttttcaag
cacacttttgggtacatatatgctaagttctcggttaattgcgtcgcacatgtgtctaaacttc
tcaaaacctccttcaaattcaaaatcttcacgaattatttgctgatagtactcttctggatgag
aagtgatgttgtacagcgcacaaaaccgctcagcaagagtaacgactgtccacgcgaatatggt
gtcgttcttgtcagcacccgtggcgaatgttgacgtgatgatggacaagagcttcttgatattt
tccaaagaagctatctggttttcatcagatgacgaagtggatgttgcaccttcttctagactag
aaaccatatctggtgttgttgatgatgttgatgcagcagcagcagcagcagcttcttcttcact
ttgagtaggtggaggaggaatactctccaattcttcaattgtaggaggttttagtgattctttg
tgggtaaattgccacatttctgcaaatttgccctttgatagtgcatcccatgtgttattcttga
gtctttgttgtagaatgtccgtatctctgggcaatttcaggtttctgttctcatatctcacaac
aatttgaaggatggcgaaaggcatgattgtgtcaaacttgatggtgttaacgccagtcaaattc

ctgataatgttctttgtttctggtttcacgcgttcaacacaattcttcttgctcatggctttct
ggggaagaagaaactccattgccagaagaggagagttgtcaatgtcactcagaaagaaaccgtg
ttttctcttgaagaaacacacaatctggtcaagcttatcatcgtcattgatgatgctattatag
acattggcagcaaggggagggatttgtttaggggctggaggaggcttgttgatgttgttctcgt
ctccagaaccgccgccagatttccctccagatcctccttcctcattatcttcttcctcgtcgtc
tccatctccagaatcgtccaatcctttacacgccatcttctcctttaatagggtagcatttgtc
ttggtcaataacaagccacctccttctgccagaaacttgtaacacatgccaattgtacagaaat
cttgatccataatcaaacttttgtttctgtaaggcatttctggcccaagttcagaaaaaaggat
ctttcttctccaatcgtcggtattggccatagaacccatgaggttttcgagatatagacacttt
ttctcgaaactttccagtgttttgcacatccactcttcggctctatctgccttcacgacaccca
gagaagtcaccatgttttcgttgctgatggagagaatgttcttcttttctcccaccaactgcgc
atggtgataggggaacgcagtgtcgctcactttacaatgtccgggaagattccagttatcactc
ccgccaatatcaatcttgaaacaggcaacagttttcttgatcatactttcaacacgttcgacga
ttctgggcatacattcactcagtaattcaagttgtacctttaagattgactttaacaacaccac
attttctgtgtctgaaacagctgcaagacgttcctcttcaaggatggttgggtagaatattacc
ttgagccccatcaaagaggcattggtgatgaaggcaaccttaaacactggcacgtctgttgctg
ttcttccacaacccagaaactctctgaaggcaggagtcacacatagtttctctgcactagaagt
ttcttcctcataaagcctcatcataataataagttccctgagaagctttccagttccaacgtgg
ggtccttcttcatccatgcgcctattttcattctggcaagaacgctcattcagcttgtccagaa
attgatcaccagtaatttctggaacctttttactttgtacatgcctttcttcttggattttgc
aacatcctttaaagttgcaggttgactgtacattatcaattttccatcgtttcctctgttcact
gtgtcatattcaacaacattacatccagcatacagaagattgttggggttgacactgccgtctc
cgccgactctatcttgtacagctagatgccccatttgtttgaccagagggttgccattcttggt
caagttatcagtgactagaatttgttccccttcgttgagaaggagaagaggtagacgtctgttg
tcgtactcgggcagtttgttggagagacagatgcgtagagtcgttggttcctctttcatctggt
tggcacattttattaagtgatctcgcacatcactttcaagaaacttgcctcctcctaggttgag
cacatttgccttaaacatattgacgagtgcagtgtgctggttataagttttgtttttttcttggt
tagttatttttctgaatgtaggataaaataacgtaacttttcttgtgtcgttgttcaccaagga
atcttttgatatcaattacagctacaactggtaaggggggttgtccgacgaccgtctgtatccta
cggtatccagtgtcggactgtgaggcagagagcttgtgagggcgtatttatacaagaatagcag
ctggtgccaactagtgattttatagaacagaaatcatggaccagtacccagaagtgagggatac
tcctcagacagaacaggaacaggcggcggcgcaacaacaacaagcagcaacaacaacagctgcc
gctgctgctgccgctgctcctacgcagtacagtaacactgtttctgcagaaactttatccgcca
tttctgaagatggaaaattggagaggtcaatcgcagcttcgtgctggatcaataaccttaaccc
tgatgaaaaaatggctcaacgtgtccaattccatccacttagttccacgaccacatacgattca
gaaaatgtgaaccctggtagttctgttgtgtttttgaagcctagagccctccccaccgggggca
cgtgtttagcccccaactacattgcagtgcctactcttcgtgctgcgtcagaaattatcgattc
tattgcatcaactagtctataccaatgttcaatgttcaattcgtggaatcttattcccatttt
atgagtaatagcaaacatagtcaattcggtgatcgtgtcatcaagagatcaatgatccgtaatt
gtttttccaaacaaaaaaatgtagaaaatcttttaaaggaattgcgcaggagaaaggttaacgc
cgccaaggcatttctcatgcagttcaacagaaatcagcagtaaatactgcccttgcagcatgg
aacgcgggaagtgctgcaaacctcgaaaaattagtagatttttgcaaattgaagtacagtcccg
atagaaaatacaaagcaggaggtctttttagtgcgtcagcaacagctcagtcacaatcaggtac
ttcttcttcctcagttgaacatacttctaatgatttcttactcgatatcctaaaacgtcacaag

ggaacgtcccttgatttagattcagcaacaaacacatttgatactgctctttctagggtttta
ccgagtttaaggaacaggctagagcagccgtagatgcagccgcagattcagatcacctatccgc
gtcggaccctatttctctattgttcgacacaatagcaggcgtgagggtattctagattcagtg
ccaaatatcggtatgttggctccacgatcaaaatattctgtcgcagagtacttgatggcagaca
gagacgagtctgcagatatcgctgctaagatagggacaaagattgcaacagattttgaagcatt
gagaggagataataataagagaagagcagatacttcagtcgatgatctcaaagaatcactagcg
gattctattgaaaaaacttccattaaaaataccggcgatattaattcggtcacaaatattccca
cagatacagaggagtatgaattttctctccacatcacacagcttttcgcgcaggcattttgga
aactatgggaagtttattgagctgtgctttcggtgtacagttccccttctcagatgaaggtttt
gcggctatcgaaaggattatacgtaaaacagatcctgatactggaaaggtttcagaaatggacc
cttcctctctttcagatcaatatctcttgttggtaggaaatttccaggtttctcccttccacgt
ttcagatcctaaggatatcgttttcggtagacaagttacgcctaatacgcctattttgagtatc
attactagatctaaaaatgataagaatgaaacgtctactattattaatttcaggataggcttc
ttgttaacgataccgtgttgagagacgccacgcaaaacgtctcaacttccaccccttctcaaag
aagagtccccaccgccgcaggtgaaccaaaaaagccaatgctctctggttgcctacctatcatc
aggggaccccaggtagtgaccagggaaagtgatgatatgattagtggtcttgtgggtgattggt
atatctctttgggggtctattatgccatgggttcatcggctgcagctattgccgctggccacca
gagagctctcgcttcagccgagtcaattaattcacccatgatgaaaaaattctcaaagaaggga
ggaaagtatacagaggaggaaaagcgaatcaagaaggctatgcgtaggaatgcagatcgttctg
ctaggattttggctttgttggggcaaactgatgcccagtatgggtatgtcgaacataattctac
cctagattcgttctggtcttcgaatgccgctattcgtgcaaaggcaaaggaggatgctcttagc
cgtgcagaaatcttggcagttaggaaacaattggacggaaaatgttcctcttcaagggatgaat
attccatggtagagagatatcttagagactcattttcaggtcagttaataggtcaggaggagg
atatgaaatgtttgatcaaggttttgatatgggaagatttgccgactttttgagtgacaactct
gctgccaggaacgcttggcaacagtacgcagaagtaatgagaggactttctaagcatgagaaac
gcgtattcaacattgaaggtcttttcagtgctctaaattctttcaagttccccttgttccaga
acaggggcgtaaaaagactgtaggtggaaggcataggcttaacaatttgaaggcggccaataag
atcattaacggcatcacagagatgactctccagtcagccatcgatggtactggaatctctgata
ttattggttcagttagcgatgggtggggaaatactacagctcagccgtctcgcgtcaaagctct
taaaacattgtccaatttcagcggaaacggaaatgttgtgtcaattccagttagtcgagcagta
aagtgtgccgcgggaagtcgaggtggggaaactctcaagtgtgtggacattccttcagtcatta
ttgcaaacctaatctctgataagagaattctagatcaactttgcggaggagggatgaacctcgc
tcacgaaatcacaaactttatcgagacgattgcaggtaaagaacatacaggaaaggaatccgtt
ttcttgtctcctagattgtctgtcattcttttgcgttatatttggttcaacgcagcggttgttt
cccttacggatagcaacattaaaatgcccctcaacacaatgtccgagggcactggcgatgacat
ttatagggactatttggccatcagaggcatggttaataattacaacagtagtctctcctctatt
tcagtcaaggctatttccgataggtacaattgcggtagtggaaatacttcaaccagtaataaga
atgtgaccattaagactcaaggtgaattgttaactgtccttcaacagactgctaatgccttgtc
cgcctttaccaacaagggaggcgtgggtgcaacccccgatgctgccaatatggccaacgttatt
tccccaattgctaatgcggatgtagttaagaacaccaacgtggttgtttcagggctagatagga
tcactgagaccatcaacttcttttcatttttgtctcagatcaaaacaatgaacgagaacattga
agagtatcttaggagatataggctaggagaaggactagataagaaagaattggataattttgtg
tatccaaatattgcagctattgttaagcgagaattgggggtaagtggttccgcattgtccagta
atctcgatactgatcgtccaattactatcgacctgaacactgaacagcctttgatcgtaaaggc

tagcaagggttatgcctctaaccgctacgctaaattattcaacaaaacaacaagaacagcagca
gaacaagctcagatggagcagtataatgcacaaatggctgccaatactattcctcaattagtaa
acaggttgaccatccctggatccatcacggcagacactgccatcaatgtcgttaaagctttcac
agaaaatggagaatttagtaacgcagaaacacacttgggggttatgggtaacgcgattaatgaa
atgcaacctcttttcacggacggattcaacgttgcgaacaagcgtttaacagttaacgtgggtt
cagttagtaagctgattcagaatgggttaaccgtatctctcattcttgctcactcaaaggctag
cccctatgtctttaagcctctcgtgcaagatttcgctaagcttttactggcagtcactgcagag
acttctctggttgtctctaggtcccagaagagtttcttccccattcctccttcagtattttctt
caggtggtcttttcaaaattgatagggaaatgttcgataatatgaagacagattatgtagtgga
agtaattagacagctatctaagaatgctaccgccgccatcgaaaggtgcaatgattccgattca
gctgctaggattgccaagtcaggtgaaatttataacaaggatgttgcatcaaccactgcagctc
ccggaacttcttcctccgctttaaccttgttcgccaataatctccagaaccctgcaaaggtatg
gtctatgggagctctcccccatttcgatatggccgtggtaccaaaacttcatggcatttctcac
gatcaaatgttccgcctatctacatattatcagggtattcataagatggaacttaacagcgatt
gcaaaccagaagaatgggataattctcttcctggaaatagggctagcaaattctttggcctttc
ttcggtgagcgataacaaccgttcattcaatttggcattggatactcttttggcttcacctgca
gagatttgcgatctggtgacgagggaaatggtaaagaccagtaacgatattgtgcataatattg
gatcgaattccaacacggacgcgcttcaaaagagccttcaagttggtgcctcagcagtagaaaa
atacgacgagtctactctttctactaaagaaactgacgtatattcccttgtttctgctctggct
aagagcaaatctcctctatcttcttcctcatctttgtcgtctgagggacatctcacctctaagg
agattgataggacatggaacaccccgctcttctcggtaccgctaaaactacatcctattctgt
ttctgaagacgctctcaatgctcctctttcagccgtgttggactttagaaggaatgttgtggat
gctactaaatctctgtacgaagttgcagctgtttgtagtgtcatgagtaaagaggaggatgtgc
gttcttcgagtagaaagattatgggaatggtggaacaagaatcgcccgttatgcaagacattgg
cattgaccgcattgctagtcttgttagtacagttgctacccccaaacagcatcgcagattctta
cagacagtaaacgattacaaaaattatctcattagaaaagttgcatcgaatccccttctctctt
caagattgggaggaatatcccctactagtggtaacaccgattacaaccttaaagctgtatatga
tggtgttgtttcttcctcatcatcaatgacccccctcgtccatgtctgtctctgacagattctgg
tcgggagtattttctcagtgcctagagactggcccttcaatgtttgccgatgctggtcatggag
gtagtaacatgttccaaatcactgcacctaaactttacggttctagagtcaacacctacgcagc
tctgagctctggcgttgagcggctgagagactctatttcttctgcgactcaggaaagaaagaat
aggattgcaaagagcatcgaagctctggaaacgttcgtaaccgatgtggtgggggagatactt
tggatcaattgcgtaaggcccagaacatgtacaacaaactgtcagatattacttccaactctat
ctatagtgatttcggaaacattgactgcgctaaaatcatgaagaatgtgacgagcaagaaaatg
accgctagacaacaatcagatactattcttagctctctcttgcacgaactcgctggcctggtac
acaaacaacaacctcaattggctactcaatttgctctggcgagccatgttatcaaggcaaagta
tgtcactaatgacctcaataatatccacgagaaggaaacattcagtcaattgatggccgtggcc
ggtgttgccgattactataatgtgtcggcagctgccatgtgtcagcgtctagttgcttccgacg
taacaatgttcctcggcggaaccatgctccaacaaggcctgttcgtttcattccttcttaacaa
cgtacttttctcccaggtttctgataatattaaaatgaacgaattgaacgatgaaacaaagtct
cttttggttaaactggtaggattttgcggtacagtttcagatgcgctaggatctaggcacgtgt
cttcaattagacgtgtacagaacgaagaggataagaaattagacaggagttttgttacatcact
ttattcagcatacagagatttgaggaagaagactgaactatacagggaaactgatactattaac
aaacttttcggacatcaaaactttatgtcttacgaatcttccatgctcaagaggacttccttgg

tacatgacgctgtttccggccctaggccaagaaggtacagcacccttgaggatgtacttgaggc
tccttccacggttcacaaatcgttcatggtttcttacccagagagggcagctgcttctaggcga
gtgaagagggctggactcagggctctggctgataacaggatggaatctctttacggggaagaag
tcttgaacgatatgaggtcttcggcggtctcttccgaaatgatggatatagagtatggtgaggg
aggattcatgatgatgattagtgatgatgaggatgatattgcctttattgattccgaagaagag
tctgaatcatctactgatttctcctcatcagatgaatattccgattcatccgatgagtatgatt
ttgatgatgataataatggccagtctccttattcaactacatcttattcgtatgatgctctaga
ccgtctgaattctgccgctaagcctcttactgccatctacgggtgcaggggagaaggtgaagac
gatgaggaaaatgacctctatgaagaagaacaagaaaggaggagacgtcgctcatcaaagatgg
ggaagatccttagagatcttcatgagagtgatgatgacgacgatgactactttgatgacgaatt
tgatggcgaacgttcaatgtcagaaactattgcaaccagaagagctggccgtattcaatatggt
ccaggtttcctatctcattctaatattcttaaccgtccggctaaagcacgcgctttcttgacac
gaggcaagaaattcaggccttctgcgtacgatagattctttatggaggatgacgattccctcct
cttctctgacgaatctaccacttcttcttcctcttccgattctccattctcctccttcagcaag
gggagaaaatgcaagcgccgaacaagcgaggaccaatgtgcctttgttaagagagttgtacgtg
cttttgtgcccaccagagtaacaatgatcaatggtcgagttagcatgatcaccccagtgactag
tgaaaatacagtaggattctatgaaaattaccagaaggccaacaagagggaaagggcgcgtctg
atcgaagaatacaaaattgttaagggtgcttcagctaccttgcccgacgaatacgtagagggta
gagcatctaaacaagtgtctcccagggaactgaggaggtctcttatcaaggcagctgcttatgt
tgcccgcacccaagaaagtaacttgaatattatctttgacgctctcaccacaacatcaaacgcc
actctagttaatgacccatctactcttttgggtgatacacttttgttcgccaaacaactagagg
caattaccgagaggaggaatagctaatgaaagacctaactgaaatctctccttcacttttcac
atcattcggtgatgcaagtaaagacacccaaatgatggccgatgccaaacagatcgtttcagga
ggaaatttcaagtctgccggctatctaggtgtccctctcagaactcttgcttcatgtattaagg
gcactaatacagttgatcgtcttttggctaccaaaaataagaaccatctcgaatggatgaccac
agccgctattgtttttgcacgttcattcaacgatactactttccatgcactcgaagatacacta
aaaatgacctccgctttgacagacatgtacagcgctttcaccaacctcgtcggatcggaacatt
ctcagcgtctaaaagtaaagagtactcttttagattctattttcaacactaggatggctcacac
tgaagcagtcatgggtctcgtataccctacagcgttcatcaaccatgaaatgccctctgattac
acacagcgcagagagatgcaatcactcgctcttaacattcttaggggagttaattgtagccaat
tgccacgaaaggatattggagacactgctggcctgttgacctttattacatcacgtaaatttgc
aggttatggaggagaaaggggaggtttgtctctatacagaatgtccattgttgatgctctttct
tgcccctctgacaatcggctcaagggagcagtctctctagaggtaggaaagtggcaggatatgg
gagaggaaatcttctacaagaggagcaacgatctggtcgatttttgttcaaagaacaatatttc
tctggaaaatgccgtaggtcctattgctaggtttgttcccaatggaactaacatggctgatatt
ggcatgaccgatatcatttctagaacagtcaaggatgacgcttcaatgatcaggcttaggcgcg
cagaagagggcgctggcgcagcaggaaaattcattacagcctcagccatgggtaatttgtacgg
aggtattgataccgttgtgaacctaactgaaaaactatacgactcgttcgttctgctccaagat
tcagactcgttcaatacaccaacagaaatggccactgctattatcaaccgtatgaagtcgagga
aacataaggctctcaaaacaccattcggggggagatattgccacctataagaacttcccatcctc
ctctgaagcaattgtagttagagccaaggaaatgcgtaactctattagcactatcgtgatggac
atttccaagtcaaggggaatcaactcgttctcatcccgcagtggttctactttggccaagattt
ccacatctgaatttgaaaggatactagaaacatctgctgttctttcaaatacaaaggccaatct
gagaaccattgagaatagactcgccgaacactacaacaaactaaaacaattcagccatattagt

aatgatggactttccgagacacgcgcagtcgttgccgtaattgctgaatctttaacccccgtgt
atgcggatgacaccagcgagagaggagcatctgttagtgaactattgacagacaatactctcct
caaatttattgttcaaaatgaactgaaaaacattgaagaggcaaaacgtcacgtgaccgccgca
attgaaggttcatcccaactgcacgaaaaaatgttgagcctgctcgttgcctcagccgacatca
accgtatgtccgcccaaaataacctcgaatgtaagaaattgactgaaggaaatagtaactttgt
accaatgactaacgaccaaggtggtacattcataaagcacaaagaaacaggtatctggctgaag
accgatgaagaaaataacaccagttctatcaaggacaatgatcagcgtagagtagctaaaacca
tcctcgcaattgtagaggacaatagaaatgcaaccatccgttctcgtctacagtctctttgctt
tggaaaatatgccatgaacgacattttgcacttgatgatgccgatattaagaatatggacaaa
ctcattgaaaaactaggcgaagcactcgcagagaaggcatctccttctagctcggccatttctt
cttcctcatcatctaacacaacatcctcctcttcttctcccagttcttccccatcatcatcatc
ttcctctttctcaatggactattcaaacaaccttgccaaaactatcccctacatgcctatcgtc
ttccaaaacaaacaatctaatgtcaattcttctgacgcatcatcctcatcaccatcatcatctt
cttcttcatctgccaatattgataatgttgagcacaaaaaagtggctctccaacaacttcaaac
acaagaatctaacgatttgagtaacgtactttctgttaccaccaagcacagatttgcgtctcat
aatcaagctgcaactgttggcatcttcaacggaaggcaacacgcagagacagttgttgctatac
caaatgcaaacaaggctaataataatgccaccgtttccgcaggccaaggaattcttacccgctt
ctcagcccctgaaaatgtttcctccaccagcatgcaattgcctccatcatcatcatcatcatca
aatggagatgataataaggtaccagtaactgtcaggcttaaccagtacgccaactcaatcttat
catctattgaaaacgcatcagaatttaaggacttgaaggaagcagaaaggaaaatcgatctggc
catccaggcagcttccaccacagaaacaaaggaaatggtcaccgtgtctaagtgcccctctgct
aaccagactgccatcactgccatctctcaagctaaatcccttaagaaaagtgccctcgaattat
tggaaagagttatcaaggcagtcgaggtttacaccccagattcatctattgcagccgtttctct
tcccgttaatggagattctatggtttcttcttcctcgggatcaggatctgctccctcttcatca
tcctcctcctcatcctcatcctcttcctctaatgtgacagactatttcaactatgcttacggaa
aattgaagaacattgatgaaaatactgaagaaggggcagaaactgtccagaaaaacatggtcga
acaagatgctgccgttcgcatccctcttctagtatcatatgctccattcagcgaaatgatgaga
cgtgctattgacaagttgaacgaatactaccaactgattgatgccatcaaaacaaagatcgtgt
cagacactaaacaggcttcctcatgggccatcaaggaaacggacaaggagcttgatatggacaa
agaacaggtgatttcaaagattaataacttgcaacaaaacttttcaaacgaatcagacaagata
aagatggctattagtgttttggacaacaaaaggaacgaattagagcttcagaacaacaaaacta
ggagctttattgaaactacaaagagccgtatcgaggctggtggaggagatgtagcaaacttcaa
ggagattatcgattacgaaaacacatctgaaaatgacaacaatctcttccagagcctgaaagca
ttcgctgctgataactcggggacagtttacacccccactgacatgagcaatggaagagacacaa
aatcagacagtaaatttgtcgacatgtacaacaaacagattctcgagggaggaatcaaactcat
caatgagggacaaaatactgtaaaggtagacttttcaaaggctttggaggctttccctagacaa
tccaacggtgcttcagagcctgtatcttcttcagttgtggagaggagacagcgagaacgtcttc
aggctgtcgagatgtttatggcaataatgatggagcgcaccgagcgtctgaggaagaggttggc
agattcggctgctcagtggaatactgttaataatgtagaagaaactgttaatagtggtatggtt
aacatcaagagtgaaaggctcacagagattaggaatcaagcacaaatcgctgaaagcactgcac
taaactccatcaacgacgagattgtagagtctcctctcaccctctctttgggagcacgagtcga
ccagctcttgatcaaggtagatagagtaggaagtatccaacagcagcaacagcaacagcagcaa
cagcagcagcttcccaaattgacagctacagaacagagaaaggaacaacaatacgctgcagata
gggttgtttacgatccttcatacacctgcttcctgcaacctcttcacgagacaattaaacgtat

ttcttctgtctataattcaaagaacaagggtcctctcagtaacacacgtggtgttcccactagc
gatgccgacttgcaactgatgaccatcactgacttgtctaggtctgtactcgactcttcttcca
cttcctccaagaaaatgctgtacgaaaatgttccctcatcaattgttcctggactctgccagca
atgcgcaatgatgatcaccaacgtccacgaagccactcatacttctcctcattcattcaatttc
gagaacaaaagatccctgaagcagctgacagaaatgttgaacgctgccacttcatccagtgacg
gtcctgccgtgagacacgatgtactaacaatgttagagtccaacaatggttacgtcaaagattt
tggattcactcaccgccaaaaggttgcgtgtatcaccccctgttaatacacttctgggaggtact
ttcagtggaaatgttgcacctaatactgttatccttcctacttctgagttgtttaactgcccag
gagttgaaaatgacaaatttagatccatggttaacaggacaaccgacaagaatgtggctgacgc
acccaagtcatctgcaagcatcgtggagactcttgctcgcacgtctcccaacgccgagcacctt
tacttccccttcaaggaccagaggcgacacttcaactccatcaccgacgccatcatttctggta
tgagcggcgaatcttcatctcaattgaacactacttgtgatcaaaatctggtaaacattgatca
aactactggcttcccagtgtttacaggaagaaagcagggcgaaagaaggattgtgcacactgaa
aacactatggaaggagctcgcaaggacaagaacagtggcatcccttcatgtacaaaggaccgtc
aaacttatatcgatatgggcacaaaattcatggttgctccaggctctcttctgaatgctaacaa
ggaagaaactctccgtctaaacaggctttcagacattaacaacgtgagacattatggcactgat
gttcatgtggcaggcgcaaactctgcatggagaattggtgaggtggtgagagccgcctcctcat
tccctgacggagataaggaatcggctatgaaaaagatgcttcttctaggatctgtatctgccat
ctctgctcaaaaatctgccagtcacattaacgatcctactgctttgttgagcaccaacacgtct
atccagaatctggtcaaggaagctttcccagaccctgtttgttcctctaattacttggggtctg
ctgaatctacgttcgccactcaactcgcctaccgccagcgcctgttccctaacggagatgacga
aaacgttacaaccgtttcaaatatctgccctatggatttgatgggaagtacaaagcgctacaat
gacgctttcaacaacatctttggctctaaaatgacatctactaacaaaaaggggtcaaattgtg
aaaatctactgaaatctgccatgtctaacgttcctgctatcaacactgcctttggagcctttga
agaagcttcatcttctgtcaggaataggctttctcccctttatgaagacagcaccaaatattcg
tccaaccaacttgctgtacaggccatgaccgatactgctgtggatgctttgtccgctgtttcta
ctgttgtcggtcgccagaatggcagaaatactcttctttctctccctacttctattacttctat
cgcaaccagtggccgtccatcactctcttattcttcggacatgaaatctaacctcatcaagaca
atttcccgcatcaatagagacgctagcctcctgtcaatgggagacagccaagtagctgcaggtt
cttccttctttaactctttccttcgttcttcttccatccctgtcaccaccagccaggatggaaa
tgttgcagcagcggaaattgttctggggactattctcgacaagactgtggagatcaacaagaga
ttcgagatgcttggaggaggaaaaatggtcgccgggagtcctgaagctcgtgccatccagcgca
atacaatgtcctctattctccagatgaacgaaaatgaactcgctcgtgacttgtgcgaaattga
aaataaaattgagactaggcaactgagggatgctttccaggatttgaagaggtctatgctgatg
actccaggaggcgtgggagccatttcttctggagcaagtaccaacaatgttcccctttctcttc
tcatgtcacgtgtcgatgcatccagcggtcttctgatgaacaacaacagtgccaatgtaatgga
agctgtggatagtttcaatactactcctttgctcgttaggcacatgatgttggatagtggaaag
tcccccgttcccatggccaaggaaattaggagcatgctgacccaaccaagagctctcaccgccc
gcgctctactgagcgaatcttcccctcttctcactgaaatctgcctctacaacacccgcgacac
tcaaccagaaagggcagtcgacagactactaacttcagcctatctagtaaaacaagctaaaaga
ttcgacggagttgacccagccttccctgccgccctcacctgcgcttctcacctcatgctttctt
ccatggattcccatacaaagtcatctttcatggacaacatcaaattgcacatgactgatactca
atgcttcttcaagaacattgaacgatttgagaaattcttgggaagatatggggacgaatacgcc
atgtcccacaagcaaaattgtaactgccccttccatctccaccacactttactccctcagata

acgagcatctggtatcctctttcgcattcgcccgcccagaagtctccatggaagaaattagagc
cacaccctatcaggccaacaagcttattagtgacaaacattacgtgatgaacatgtccaagatc
gattctagagtaacaggatcttccctccttaagaaggttagcgaatggactgaaatgagaatga
actccaactttaatggaacatttgaaccatcaagactcgccctctccaactctggcatgacaac
ggcaggagtcaacctcgacgttattgtcaaaccaaataatgcaagaagtgtactaggaatattg
gaatgtcatcgccagcacgtgtgcaccgccgacgccaagggaactgtcgcttcagccatgccag
ccgtcttccaggcaaccgatggaaacggtaacgaatctgaactgatccagaatgctctgccaag
gaacagatacatccaaaagagcacaatgaacgctcaaactgtcgtgtttgctaatgttttggaa
caacttatcgccgatcttggaaaggttatcgtgaacgaactggccggcaccatcgctgaatctg
taccagaaagcgtatatgaaaacaccaaggaaatgattgatagactaggctctgacgacctctt
caaatctaataataatggaggagtagaatcaatggattatgaagatagcgaaacaacatccaac
aatggtcccgtcctcatctcagaagccatgaagaatgccgtctatcacacactaatttccggca
aggcagctcgcccggaaaatgtaccattcgcctcatgcgccagcggccctctcgcctttgattt
ccttctgtcaaagggagatacattcgaagaaaagaacgccgaacaaggtgcagcagctgccgta
tcctctacctattcttcctcttctaacactactcttcgtaagcatttggctcgagttttcgaag
ccatctctaagcaagtaactgatgctgaattcaaggatatcctcaacgatatcgaacgtaatat
ttcttctgactatactaactgtccaccaaatactaaccaaaatgcctttgctctagctatcaag
agagaattcagcagaattgtttccttcttaaccattcttcgtaagaacattacacccgcattag
tcgaccctaaaggcgcgttacacgagaaagtagccatctatttgacccttctttcaaccaaatc
aaaactagaaaactttttccaatacggtctcagtaattcgtcctcagttgatcttagccatcta
aaacccattaattgtagcaacaatgtcaagaatattgaagacacattcatgtacagaaatgtcc
accctattcttattatggccctcccagaaaatttcacagctctcttgcaacaggaacaaatgga
ccccgatactgccattgaaagcagacgctcccttaccaccttccttaatcatcccaacactgct
tcaatggcgaacggtgcaagagccgctgtgggtgcaggaggaggaaacccaatgggcttgtatc
tttcttcccacattcttcacgagtctaccgtcacaacatcaaaccccgtcacagacaccacaga
aaacgtcaactatcattcctctgttacacaagatcctgttatggtagtgaaccccttcaaggat
tctgctaggttgatcgttaacaacaacaatactggaattgatgtcttgaatgataagtcgtgca
actacttgcaagtatccatgccatctgaatcatctggcctcgtcaccaatactggatgctcttc
ttcttcttcctcatcttcgtctgataccttcaagtacgtcaggagagacaatacgcctgtgaat
cttccccgtgtcacaccagccgttctctgttctgatgcttcctctaatctcttggacgtgttct
ccagggcagatattgtcctcgaaaacatgaacgtgagatttggtttcatgcccgagattattgc
tgccgtctccaaattcaaggggctgaccaaggaagaggttattaagcaaatggtttctcagaac
aacatcaacaacaacagcaacaacaacaacggaaatgggaagaaaacaaccgtcgatccagtca
ctggggatattgttatcaccaatgccacattccccgacactcgtcctctatacactgcagcaaa
tggaggaacatcatcattcaaatggggagatatcaacgacagaaaaatgcacgccaaggctttc
cccaccttctttattggtaacccaaccgccgccgcaacagctaacggagtgcctcttacatctg
agggaatttccctcactgaagaaaaacgcaagaaaatcgcaggcatctctgaaggatcaattgg
cacgggggctctgcgtgcagccgccaacacccgcctctcatccgacatggaacctgtcatgaag
ggatggaacaacattgttcagcttcaacaaacattcaagaaagcttcagataaactcactcatc
ttttgagatcgggaggaattccacccagaagccaagaaacaaacgctattattaacaagatgca
cgacagcttcaagacattggaggaatgtcgtagggtgatccaagacgaggctgctctgctcgtt
gccaccagcgatcttttgaccggtggctacggaggagatgctgctctggccatggtttctccag
tacgtccagaaatgactggtcttattggtgcaatctccgcgccagttagaggtattagccactt
gttgaaactgggaggtgtttctgctgctaacgcagctatccgcaagcgcctcaacctacctaca

tccaacgggaaaacactaccagaacatggaatcgtacacaaatcagccaagacacttttgcttg
attcagactctattagcaacctatacaacactgatcttcaagacgttgtctctaacgctaggga
taacaacaatttgggaagaattatgcaatctttgggacttaaggggaataatgcaggggatttg
gtttattctgctagacaactgacggaccttattactgtaccagaatatggaaacaatcgcgatc
ttaccaagcgtcaagctatccttaaaatgctcatttctaaccctgaaattctagaaaatgttgc
agataccatttaccttacaacaggtaaaaatgctctcgcaccggtatctgctcaggaaatggct
tgtgcgtctctgacagtcggaggaagtggaggaggaaaactgtcatcagacgacaatgttcaat
ccctgaaccgcctttattttcgggtctagactagtgtaatattgtatgtttaatttattgtgta
acaattggtttatatttgtatgtgatttttttcggacaaataaaagaattggaataaaagacttt
gtattatttaccaaatttattgattttttttaaacaccttttttatgccacggggttgattgtag
catttcctccccctggagatttcatagtgggacatccaaagagtgtaactcctagtgctgatac
acccaaacctaggaggaatccggtaagtgcaggagtaacagaaagcatgaaagcgaccatgctc
aatgcgatgacgacaacggccaagccgacgatgatgtttctggtcatatcagacatgatttctg
gtatctgtttatagggtttcacccagaagtgttttaaacccaaaagtataaattttattctata
ctgctcatctcactcttccgacctcacaaaggagaaaagagtcagtcttgctccagccaccacc
gaggcaggagctcctcggacgctcctgtatttttgtccagaaatggataaagtttgtgttatat
caaatactagggagcgcacgtttaaggtacctgccgatctactgtgcgttgcaacagaaccgga
aatctctaccaaggaagaagatgcaggtatagaaatcgagacaagagtggtggtgttttcaagg
tgcgtctcggtccaggaactacatacaataaatccaaatgatgaaggattttctgtccaacttt
tcaaggactacctgaaattgcaatctgcacaaggaaaaaaacccattggtttgtacatccaaat
aaaggctggagaggatcttgaaaggagattaatcagtggaggaactgcatacctggatccggca
acacaccttttctatcttgatttctccctttaccctaattattcaatattcaatgacatttcat
cccgtctgaaaatcattgatgaagacacgtacaatggtgttgttttctctaacagtgaagaaaa
agagaaggatgcactagtgctgataagggtgactttttctacgcatgaaaaggcaattgaagca
gccataaaaaaaataatgctaaggaaagtgtttttcaaggatggagatcttgatttcgggtact
tacgtataccaaaatctaaactggacaaatttactccctattttcggagtcaatacgggatagt
aaatgttgaaaaaaatatccctggttacatatggggagaaattatgaagcaacgagtgcgatgt
tccagatggtacctttacaacaccgactcggaatgggaatataaaaatgtggccgaagaaagag
ttggacctcgccagttagtgaaaaaatatggtgccaagtgtgaaaatttatgttttagggacat
agacctcagaaaaaaggaagcaaaggaaaaaagggatatagaaagagaaactgaaagcagatat
gtggtcgtaacactaacccataagcatgaaatgcctgaaaatatgccctattttggaccaaagt
gttcagtggtgaggttggatgaaactagaatacttttatgttttgtggatgaaatttcttataa
tgatgaagatgtagacgaaattttgtctgagaatagatcactaagaaatgtttctattagacat
aaggaaaatgtacctgtacacacgttattaaaaaaaggtgtgtctattcatgctagatttaccc
ttaatggtttggatgatgctttaataattttaaagagaataccaaaaacttattttgaagatga
ggaactacaagccgcttgtgcgcatgttaaccttgaacagtacgaatggctttgttctaataat
agagggaataaagtagaacatgtaaagtcgcgggtagtgactcgagcagttaagcgtaggagaa
aatgtagacactggatttattttgataaagacactttaaatttaaactacaaatactttgataa
aaaagttactgctagtatggcatctaaaatatgtaatgcaaaacacgactgtttagttttccat
agaaaaatggaattggaagatttgactgagagcgcatatttcaaggtagaaccttccccaataa
attttgccaagttaaaatcttgcccggatgttaaatatgtgcagaaaaaaacagatggtacatt
ttctgttataagattctttagaaacatgacaaagggtgatcttattcaaaggatggatcttttt
tgtaggtttattcccgactcacacactattacacttttgagtagggcggatttttatgcatgta
aaagaggagaatctatgcatatgtgcacaaacaaacaccgtattcttcactacaaattctccaa

cgctccccatgcggccatcgaacaaataaccaatatcatcagtgatacaagaggacgtaagggt
atacacatagaatacgcgatcgaaaatgtacaagaaatgtacgaagaagatggaagaagatatg
aagctaaatacactggaactttaaccgagtacaaaagaaatgaggacaaaaccttcaaatctct
tcttgctcctcatttaacacctgtcaacaaaccatataatattaaccatttgtatgagcaatat
ggaaattttgatgaagaattagaagacaagttgaggagtggtttcatttcttatgacacgtatg
ttactgcaaaagacaactggggcaggtgtgcaactggaaagggggcgtgcatctaggacccata
actcaaagtcaatactggtatgtgtttttttgagaaggtgtcagaatgtgcacattaaaaacata
caaaatgactacttcaacagaaatatcaaagaacctttcagatgtgttatccatcaaggcaact
ggagattggtgcagtaatatcaagacggtattttcacccttcacagaaggcaagggaaatttac
caaacagtctcccgtttacgagaagtcccaatacaacatgtggttcaagagaggcggcaaacgc
cacagagcattttatcaccgtctttgcaaaggacaaatataagcggaaaagagtaaaacgtaca
atcggattcaccctcgacaacacaaaggagttgacgcccaacagatacttggtagcagatgtat
actcttggcaagaagagaaaatggtgtttgaaggattttgtgtcccaccaggaaagtcgggaac
atttgtacgctactctaatgaagataaaagttttctactagcagataccggaagatatatgaaa
aagaagtacgatgatccagaaaataagaccagtagtgggggtgatgatgacgatgacgacgatg
atgatgatgacgacaacaacaatgttgacgtgtatgaagaaaacgaccccagaaatgtattcga
ggtcgaaaaggatgaaaaatatgcctgtactttttcaattttggtctatagagcaatgaaaaag
tctcctcctgtatgtagagggttattagtagagacagatggaccctcatctcaccctaaacggg
ccccgtcagcatttaatccattcggaggaagttctatgttgaacggttatggtgcaggtgcaga
tgcactagaagaagaggatgaagttgatggagttcctgaaagagagaggattacaaattttgct
ctcaagagaggacctgcaactggccagaactttgtatctgttaaactggaacatgatggatcta
aagcagacctgtacaacgtcacgtgcttctccaagcagcgtggagtataaaaggcgggacgcat
cagcaaatgcaactcattctttctcatcatctaaccatggctggtcgtgtagagctcgtcactg
gacccatgtttgcgggcaagtctacctacctgaaaaacatataccaacaagaaaatggaggcaa
taaacattgcctgtttgtcaaacactccctagaaactaggtacggttgtggaactggaacaata
gtcactcatgccggagaagtgattgaaggttgtactacagtttcttctatcaaggaactaatca
gtgtgttaccagaagttgtggatgtgattctcattgacgaagggcaattcttcacggatttggt
gctagtcaatagactggctgacaaggggaaaaggattgtgattgcagcacttgatggaacttct
gaccagcaaatgttcagtcctattcataagctattgccttatacaaattccattgttaagctag
catctaaatgtatgatttgtaaaattgataccaaagaagctccttttactgtaaggtttggtaa
tgacaatgataataatgttatatgtgtaggaggagctgaaatgtacgctgctgcctgccgggac
tgttacaaaaaaattaacaagaaaaagaacaaggggaaacttgttgtacttgaaggaggtgaca
ggtgcggtaagagtacccaagccaaactcttgttgaccaataaaaactcgcctctttatggagg
agaatacatgtgctttcccgacaggagcagccatacgggtaaactcatcaatgattatttaact
aagaaaattgaactagatgatcatgcagctcacttgttattttctgcaaatagatgggaagttt
gtagtaaaattaagcagttgttagacgatggaatccatgttgtgatggatagatattactactc
ggggattgttttctctttagctagaggagtggataccgttgagtggtgctctgctagcgatgag
ggacttcctcagcccgatcttgtattgttgatgcttttagatgttgaaaagtgttcaaataggg
atacttttggtgtcgaaagatttgagacaaattccattcaagaacgtgctagagccctattcct
agacctcgcaaataaggacgaaaagaatgtatggattaaggtagacgctcgcggcaccattgag
gaggtgcaaactaaaattataaatattgtatataatattgttgaagaataaagaattgtaaact
gtttccatgatgtgtttctttcctagtgattttcacatgttacctagaaagactttgcccgaca
ctgaaaatgattattttgtcttggacgagtctcttctggagaaggtgtactatgataacaacaa
tgaactgattgtaagagttggtgggatttatatgcagatatgcaagtcaaaatacatcttccat

cacgatgatccagagaggttcttttatagtgtgttggaggattatcaccccatcaaagagattg
ttgaacgactagcagaagaggatggggtatttttaggaccgtgggagtttttatcgcgcaaaca
agtgaacctccaacacgggtgctacaaagctcttttgtcattgccagaggacaaatattgtaac
ctattattaccccagcaaatgaaaaccaacctggaaaaaatggaagaaatacagcgtactagac
tcattcactctagaacgtacaatacaccccagatagaattgtctgaccagctagatggatgtgt
tatatgttaacactttttcagtaccatgtaatatttttgatatcaaattgaaaggaaaatggtt
cagaaattggtagtaaaaactttattgtttctttcctctttcttttgtctgtcactttattcgt
cagacataacaaccataatactgagtatgggtcttggatctttgttgggacattttgggtgtgt
taaaagtatcacaggaaaaatcgtctcaagaaaggcaatggataattataggctcgtgtcggtc
ctcggctctgtatgtggagcaggaattttctgtggataccagcaatattctacacctcagtctg
cacccatggcgttgctgtctgtcttctgctgcactgctactgctgctacatcaactattgtagg
attctggatatcgatgattatccaggcattgaaaaggagtaataaagatataatataaaatttg
tgtgttttatttgtttgataatacaattttcaccttgtaccgaaacccattctctacaagcact
gtgtctttggacacggggatcaatatcttggattttggatgaatgttcctgaccctcatatctt
cttcattttcaaatacgaggagggatgttttctcagtattcctgttgtgtatttggaggtgaac
agggaaggagaatagttgattgttctttggtgttagatggaactcgtataccatgaattgtact
cctccaatatcttcaagatagatgcatgatgtatttttgtcataccatctcgtatggtcgtcag
tgctcaggtacaatctcttgtggtggtggttacacatctttccactggtgggagaggaagtatt
atggttcaatattgatggaggatggattttttataccctcatgcgaatgcgatgtggaaaatttt
atatgtttccgacaaggggaaaaatatgaataaactgagcatacacatgtcgtatccagatatc
taggtcgggaagaatccagaaacgattcaaaccatttctggaacattcatttctggaaagggtt
acattttattgtatctggtgcattatttctggtaccatttttggcacctctgtgtaatctgaca
ttgggtcgacccagaggtccaccctccgaacttgacattaggccgacccagcggtccaccctct
aaactcgagtgagctgaaaaaatttttataaaatttttgatgaggaaaaatagaagtaagatgc
ttgtcaatgcatgctccctgtggaaggtggtggatgagataggagggagaggctatccagatat
ctaggtcgggaagaatccagaaacgattctagccatttctggaacaatcatttctggaaagggt
tacaatttattgtatctggtgcattatttctggtaccatttctggcacctctgtgtaatctgac
attgggtcgacccagaggtccaccctccgaacttgacattaggccgacccagcggtccaccctc
taaactcgagtgagcagaaaaaatttttataaaatttttgatgaggaaaaacactaccagtgga
gcgaccgtacacactcctctagctaactactaaccgagagagtgaggcagagtctgtccagata
tctaggtcgggaagaatccagaaacgattcaaaccatttctggaacaatcatttctggaaaggg
ttacaatttattgtatctggtgcattatttctggtaccatttctggcacctctgtgtaatctga
cattggcctgacccagcggtccaccctccgaacttgacatcaggcctagccagcggtccaccct
ctaaactcgagtgagcagaaaaatttttaaaaatttttttgagatggagatggagtgaaacttcc
taggtgtaaggtgcactacagaggcttgtgatcctttctgggcggctcactctctagaaacgtc
tgctccagaaacacataaaaaagataaagtacatttctggaccactcccatttctggtgtaatc
tgacattgggtcgacccagcggtccaccctcggaacttgacatcgggcctacccagcggtccac
cctctaaactcgagtgacgcagaaaaatttttttaaaaatttttgatgagggaatatagtactcc
gtagccaacatatacacatgaacacatgaggcggtctacaacagaaagagaactgatagctgtt
ccagatatctgggtcggccagaacccagaaacgtttcaactcatttctggacaagccatttctg
gaaaggggtacaatttcttataactggtatatcatttctggtataatttctggcaccttcgtgc
aatctgacattgggtcgacccagcgatccaccctccgaacttgacatcgggcctacctagcggt
ccaccctctaaactcgagtgagctgaaaaaatttttgaaaaatttttgatgaaggaatatagta
gtatagtgcccgccaaagcatacacacactcgcccatgctcgtctagctgatagctgttccaga

tatctgggtcggccagaacccagaaacgtttcaactcatttctggacaagtcatttctggaaag
ggggtacaatttcttataactggtatattatttctggtataatttcaggcaccttcgtgcaatc
tgacattgggtcgacccagcggtccaccctccgaacttgacatcgggcctacccagcggtccac
cctctaaactcgagtgacgcagaaaaatttttgaaaaatttttgatgaagagattgagtaaaat
ttcttgacgataagaggaggcagtaggtgaggctgcttgtttgatgtgtcagccacatctgcgt
catacattatatttccaagaattttgctgacgtcaatggaccataaaaggctttgtacgtccag
agacaagttttagtctgatagatttcttaaaaaaaaagagggtgggaggtttgtttttgtgggt
tctgtgtgtgtataaaagataggtgcaaaggtagagaatcatcatatggacaaaatctgtccat
gagacctcagtagagaacgcaccatggttgcttcaactccgtgtccaggcccaggaccagttcc
aacccaagaacttctttctacaaactttcttgaagctcacaagcttgtcgtggaacttcttctc
ccgtcctacagtagtgatgtagtttattgtgactctgagacgtacaccaaacctataccgattt
ttgggaacaagagtatagtttctaccattggagactatgtcttatcaaaccccaatgaagatgt
gagttaccaaatggtttcttccgtcttagaaaaatttcccttgctattccactgcacttataag
acgaatgaagaagataaaggtattcctctgtggaagaagttgtacaacaaaagaaaattcaaac
tcctcaactcattgttggttcataacaacaagaactggactcctgttccagctatcccgtttga
cagggagaatatatgtgatgcttcaggaaggagtgttcttatgagtgaaataatgtccacgtca
acttttcagacaatttgcaaaaacaacacacattacttgtttgatatgttaaatatggaacgtg
gcaaacaaggagggagttttcttcacttctttgcatctaggaagaattcttttactaactttga
aaatgaagaaatggactctcatgtgctcagtaacatagcgaaattcatatgcaatgaaaaggaa
aaactagactctttcatacctgccaacggaaaaataccatgccctgataaaactaatgatgaag
ggtacatcccgctggaaatagcaattatggaagacaattaccctgcattgctatatctcgtttg
taggtatggagcatcttgggcaaacacatacggggatcataatgaatctctcaaagcgtttgca
ataagaaatgatgcaaaagattgtctggaaattatagagtttataagtgatcactacagtttca
acaaaaatgtgacgaaggaagaatttgttaaagagaagactgtagaatgtgttggatgtttata
tgatattgaagacgagaaacgttgttacaaactcccatgtggacatttcatgcatacattttgc
ttgtctaataagtgttctaaagctaactttagatgtgttaaatgtttccaaacctttgatgaca
caatttttagaaaatgtcccccaactatacaatggaaaatgggtataaaccaaacgactaacca
taaggaaatggatttgttcaatcgtgcatttgacacatatttagattttatttgctcatataac
gtcaaattagacaaaaaatcaaaacctaaacacaaacctgaaaacaaaaaggtggaagaagaac
tagcaaaaaggacagcagaaattgaagaggccataaagaaaaaggaagaagaactagcaaaaag
gacagcagaaattgaagaggccatgaagaaaaaggaagaagaactaacaaaaaggacagcagaa
attgaagaggccatgaagaaaaaggaagaagaagaactctcaaaatataataaaataattgaaa
agggaaaaagacgactgaatgaagaatgtgtcaagctgagagatatttcaactgcagccataaa
catgtacaaagagaaagtgagaattaatggtgtattactaaaagattccgatcaggagttggct
gaggcgaaagagaggttgaggaaaattttattgctagaagaagaaacaaaacttgacagatttt
tgtttagaccgaaacgagtagaagaacgtatattcctaactaaagatgatgaaacgttagcctt
caagttagccctagaaaagaaaacggaggacataattgcgaagaaaaacaaccaaaaaggcagt
gaaagaagagatggagaatatactataacttctcatattgagaaactacctcaatccactgctt
tggctagtgtgtgtgtgttaaacgaataaataaaagtataaaatgtaataatattgttttaata
tcattaatattttaccacaatgtctacttgttcgaatttgttgtcagtatttggtggaggagat
tggacaacaacattcccattcgacctcgtccatacacgtcaagagtgtgataaaaagagagagc
aagactactcatttttcattactgaaacgtgtaaaggagagaatattggtatacattcgtatga
acacacgtcaaagattattgacacgggtaataatgattctacctcaatagaggaactagaagta
ctgaatatatacaaagctataaaccatttagaaaatatcctaaaactcaacaaaggagaaaaaa

ttatactgatggatgtagaaacaatgatactggaaactcataaaatttaatgaaaggattct
tcccaagggtaaaaatggaagtttcagtacatgcgtacgctttgctgtaaataagaacaatgaa
cggcattactaccctgtatttgaaacagagaaagaagcgttcaattctatacaaaatctagtag
attattataatgaaattgtagctcacaccaatgaccaaattaaaataataaaagcgtgcgcata
tttcatgtacaactttctaactctccaccctttcaatgatggtaatggaagaacagctagatta
ttgtatagttttctattgaaaggtaatggtatcgtacctcatttttcacccataacacaccctа
gggatcaatttgttgatactttagtgtattttagagaacatggagatggacgacctttattgta
tgttttgctggaatcaataaaaaataagtaaaattcattttgaggcatttatttcttcatcaat
tcatatggcatcttgccagttttctgtattgttatctcagcaataatgcgtaacacatcagcag
atgtgcagattttatccccttccccatccctcatggtatttcctcctgcaaagtaatggaggaa
agaatctccagtaatggggttagttacattcaatacagcaaagaattggccaatatctttgtcg
gccaacttgtacaaaagctccttggtcatgacttcactcataatgatagattttccagtggtgt
cacaaatggggggatttatcaaacggcatggcggggatattggggtggaatgggttggagaataa
caccaggtcgccgaggagggcaaacttcccgtgcttgtaaagctccttccacaggggcgtattc
gtctgcttgtagttgcaatgaagtaggacagggaatcgcctgagtacgtgggacagcactttga
agctcatcttttcgtccgaagaagaagacagtacactctctccaacatcggtgacaatattctt
ccctcccgacttgttcttgtacaatggagactcgcaatcggtgaacatggcgtcggtagggagg
agcatggtttgcatgaacatgctgtccgcgttgaaaaagtcctgctcggcaatggagaatatgt
tttccaatttgcttataagtgagtccattgctgctgcttgctgcttgctgctgttaggcgtcca
gaaagcgtgtgttaatttctcggcggaggactgttcttatatacaaattcgttaaagggtgacg
cccacagataagaatgtggttagacttgaatctatttgaaaccatgacaaggcatttcttttg
taaggggtgaataaggactagactcctctaccagaattcatgtgcgtcattgcaagcaaaaact
ccctcttgttccaagaattggctagtgggcggctgcatgccagtctgtatgacatcatttcctt
atgtgcaatggataattataatttgtaatatttgaagtcgtcgggtccggtccacccagaaagg
gggcgaatacacataacatttctgggggtgtatttagtgcattatatctggcatccagaaacgg
atgcaagcagtatctgaaactctctctcacttttgacatcaatgaaaaattggacatcaacacg
cattggtgtggtggataaaagggggcaaccggctatgactaacaagcatttcctgcctaacctg
cagccaagcaacagcatattacacaatggaggacctaaaatccactatcgagagagtatatgaa
gaaagagtggagaatctagaacaatggacaaatactgtagaggaagaagaaaggactgtctcag
caatcgattctgtcctggaggaacaaaaaagggccctggacgcatgggaagcagcgataaagga
acgagaaaacgacctcgcagtaaaagaagggatatctgcactcgttttcaacgcagcagacgcc
aaaacacgtaaagaattgataaatacgtggatagccgaaagggaaacgtcagaaaaaagaagaa
aggaagcaacctctaccaataatcaactgaagaaccagatgtcatctctagtcaacacaaccaa
aacactcaaagaaaagtacaacaaatattacagaagaagtgccatactcaacatgcaatacatc
aataacaaaagggattatgaagcaagtcaattttgggtgtatacaaacaatgcataagttttca
aataaatttatttataataaaaggtgttttaattataattttttgtccctgatgttgtgtttt
ctatattttttactgcctcctcttggggtaagacataaaaacggtggcggcggaaacgaggaaca
gaagagcggacccagccagaagcatcatatccctggtcctgttcttatatttgtcctcatcatc
gttatcgttggcagtgtcgtcatcatcatcggtgtccttatcagtgtcagaatcgctgtccttc
tttggtcccatccatacattcatgacggccaggacgaggataccaattgagatagcaacgatca
aaaagagacccatgcgagccatagacatggagccttcaagatcttccatggtgtaagaagggcc
agcggcctgggctccggtcctgccgaaaggaagagtgttagtcgtggtggccatttttttttctg
tttgatgaagaggtacgtcaatgtaacgtaaagacctgttgttttatcgtgtacggtttttgtc
aggacaccttacgaacctatttgtctttttagacttactcagcgttagttgtcgcagttggtca

tgttccgtcaattctgttcactatatcttcttcaacgccgggttaacgacaatcttagatccac
ggcgtccgcttcggcagcggcctcattaaaaggagatggaactgaatttataaccggagaacca
ccttctcataaaatgaggggaccttcttatagcgttttaggacctgatccgtgcgaggacccag
aaagggtatatgttgatattgtagtgtctattttgcagacaaataatatacaggtaacaaaaga
atgggaattgttttccgataagttgagaaaattgggtccatggattgataggagcggaattgag
aataatggcgaaggagaagaagatggagatgaaaatgaagacgggggtggaaatgggggaagaa
ttgaagacagagaagcacatcgacgaaaaatgatgaagaaattgtcctttgttggaagagaaga
tccagtcgctgtagatttacccacgtggcgagaaaacagtacagaatttgcacgtcgtttaaca
ctcaaggaattgtgcgatttaatagttgaatgtggatgcatcaaatcaaaagaggaactctttg
acttcattttgaagaaccgtgggagattaaagaggctgctgacgttaggggtatggcaaacag
gagtaaattcaccaaggaatcattaattgactggtttttgagttcgacacatatagtaaatgt
gtagtatttttgaagcagtcaactggtacttgaaatctcaagcgtctccaatttcattggtac
tagatgatatatattgttgtgtcttttcctacataagacgccaaacctttttaactagggcaaa
aaacccatctttaacagtggcttcatccttttctcccacgcccgacacaaagcttttggctatc
gacgagtgcgtgcaacacttttaaaatcagacattaatattagccagatggcattaactgaaa
gggactgcttcttccctcttttaactgaaatgccccgccaacaaaaaaaagtaaacaccttcct
ggacacaatgaagagacctaccttatctcttctaccttccacctcctcctcctcttcttccaac
aacaagagaaagagaaatactgccgctgccaatattcttcttccagtgtacaggagtaacttttt
ctacagcatccaataacaagagactgaaaactgatgatggggaaaatgcatcagcctgtattct
tatcgaagggtatgcgaatggaaaaataagccctataaggattatggtaagaaaatcaactatt
attccagaagtgtttaaccatcttttgttccctgtctttgcctctaaagacactggtgcgaata
tcttatttttttatcaaaatgaaatcctttgcaagtgcatctttactcctccctggactttttag
acaccccaaacaatttctcaacgggccgtgcaaatggatgactctagcagaaaacaacatcaac
gacaacaacataaactcttccacgatgtggagttacacgctagcagattattgtcctctgggct
attacacccaagagagccctcaaccctatcagacatgcggcaattttacttcgactacaaacaa
gagactacaaaacgtgcagccattatactttttaaacactcttttggaatactacaggacacctt
cagaagagtgggaaattccgtttaatctcttgcttaatgtgatgaataacaagtggagtacact
cattccaggtgtcaaaataagtgcaggtatcatatcgaaactcccatggaccatgaaaacaatg
tacgagattgtttcttcgcccaataataataataacaacggagactactattctacatgcaggc
gaatggtaatggaatatcctatcgggggtttattgcacacgcctgccataactaataagtatcc
acgctccagaatggtcacctgtacaaagggcaaagaccaccagaagctatatgacatctctaga
caaatgtttgatataatagaagcaaatggacaactctgattattattattacggttacgaaaat
tccccaaagactaaaaaattcaatactgcactgatgattgggttcattttatttattttattaa
tcatcggaatttggggtttatttagatctcgaaatgcaaccacccaagagagcaaaacttcttc
cccaacaatctcctcgaccccaactacatattctggcagctcaaccagcaggggtccaggttct
ggatctggaaacaaacccaaagatgacacatccgttgaaggaatagaccctggcttactgtaac
agaaaaaagagtaaaaggcgacagctcgcttgccaattgtcctgttacgtactctgtggtttca
cgaggttgtcatcaccaaaggtaacctttttttttgtcctcgccgacaaaacgacatcttaata
accaagcaacgttcgataaagaaaaaaactcgtcatggatctttctttcactctttcggtcgtg
tcggccatcctcgccatcactgctgtgattgctgtatttattgtgatttttaggtatcacaaca
ctgtgaccaagaccatcgaaacccacacagacaatatcgagacaaacatggatgaaaacctccg
cattcctgtgactgctgaggttggatcaggctacttcaagatgactgatgtgtcctttgacagc
gacaccttgggcaaaatcaagatccgcaatggaaagtctgatgcacagatgaaggaagaagatg
cggatcttgtcatcactcccgtggagggccgagcactcgaagtgactgtggggcagaatctcac

ctttgagggaacattcaaggtgtggaacaacacatcaagaaagatcaacatcactggtatgcag
atggtgccaaagattaacccatcaaaggcctttgtcggtagctccaacacctcctccttcaccc
ccgtctctattgatgaggatgaagttggcacctttgtgtgtggtaccacctttggcgcaccaat
tgcagctaccgccggtggaaatcttttcgacatgtacgtgcacgtcacctactctggcactgag
accgagtaaataaatcgtgctttttttatatagatagggaattttaatattacaacaataagaaa
ataaaacaattgaggaaatttataccatattttattgacctacttaaccttcttgctatacaat
gaatgtttaggtgactggaaaagtttagcaatattatccttgaacgggaaacatgcaccaatta
caggcgcaatttcatacgctctcggcctattggtcttttcctggtcatacattttagatacaat
agacaaaaatggaatgtttgtatagatagaattggcagacaaatctgcagttctcttaatcaaa
atggacaacatgtctattaacaaataagccaacccaaaagtcatggcagtttctgaacacaact
cactgttaataaattcaggagctgtatgaggatggttactaaagaacctctcatcagttcccca
acatttaaaattgtagtactttttacatggtacaattaaaccaaaatcaatcatcttaggttga
ccagttattccatcaattactatattgtcacttttttatgtccggattcactaatccttgttgag
acaaccgagtgacaatatttacaatttctaccaaaacaaagggcaattggtttaacattctctc
cctcatttttccaacgatagctatggctgaaattgaatccgtaatgggtttcttgcatttagat
tgtagaccttcaggcaggcggccagtagcttgaagcatcctaacaccgtacagagtatccctca
tcctacacaagctgccatgatcttgcatgagctcagcggctggcttggaggaagtagtcgaggg
gttaggttgctgtacaacattctcgtccattttacatttcaggtctccatttaatacatcaatc
acagaaatgcctgcaaaatccatctcgacacaaaaaccttcaacacacatctttctcacacct a
ctgcgcctttcattcttccctcaagagcgtgactaaaaacagtttcaaatacaaattcacagta
catttccttgtccgtcataaacttgataacttttccagtttcaagcatgtaatataccccgact
ttaacttcgagatataattcagatggtccaatcattacgacttgagaagcatattctggttgga
cgtgtgctggtacaggaatatttctgcggtaactttcccatttcttacattgggcagtgacctc
ccccaagagtgtagttttcttctccaatatagattcacatgctccaggggaaagcttaaaaaca
ttctttgccatgtgatcgatgctcatggtttttatccaccctttaccattattcttgcataacc
tgttccagttcaagagagggctagtgtctaaaacggataggtttttactaccaaacccaacagg
ccaccaagatgaagggggttgtttagtacggttgttagaccacaaaaacttggacgctttttcc
tttgcgtcaatacagctctgttttatggtctcgtagttggacgacacatcttgcacgtcagaaa
tgaggcctaattttgcggcctgtctaatcaaatggaacggagattctagaccggctgcaatatt
ggtatcagcctctgccactgcaaagtaacacgggggtttttctagcttgacccacttgtcaatg
gaagaaaagttcctctttactacatttgtttcagtcacggtatcacaagaagatttcaagtgtt
ttggccccttgacatgggaacggaggacagacagtggagtatagtctctagccttcaaattgac
agtagagaattttgcacaatttggatgagagaatctttttctgtactgggtgtcggcattggcg
cggtgcttcctcaacacagacgcggcactaacagaaggcaattgctgcttcttgataatgtctg
tcgcatacgttggatttaatgttgcaaattgattttcatcaatagtaataggcctcttcttctt
atttctattaaagttgtaaggtgtgctctcgtagtcctcgcggagcctcaaagtcacaggaggc
ggtgcttttcctcccacctctccattattcttctttagtctcttcatgggcctaagagaataag
gagaatctgagggaggggatcggcgagtagcgtttttagagcaacaagatttcacagctatggc
tgaaggtatcttgaattgccctcctccttctcctccttctccttctcctggcgttttcgattgg
taaagtcccatcacggttgctggcgtaagttttgtccgttggtccccaccctccattttaacga
cgtttttatactctgctcgggctctgggtcagagggggctatagaaatcactcgtggatagtt
cctttatatatttaaccaatttttcaaacaggggttattaaaactactcttcttttctgtatat
attaggtacaaacacacaatacaggtcaggcctgcaaaggactgggtatttaccagtccgaata
taacagtcaaaaggcttattttccttgatctagagctggaaatgagtgagtaaaaacttgctga

gacatatactgtctggccgccccttccttattagcctgaatagaatcctcgaagcttacaacta
cattaaatttccccataccgttctgggttttcaacatatttgcacatccaaacgttacagtttt
catggatactacaccctcgtctcctaacaaatcacagttcctactatttttgtcagccgcattc
ctttgcctcttgacagattctgttacagcgtcctggattttcttggcccacagagctttaatag
atctctcggacaattgttcaccataccctaaaacagcacctctggcagtaatggggcagtaatt
aaaacagacaacagaacctccttctctccaaattctggtagatagattgtccgcttctttattc
aacaataaacttcttagacagtttgaaataatgtccaattctttactcttgtgggaaaagggtc
ctccaataaatcgaccaaacacacccgcaccagaagaaagggtctcgttagaagatggttcatt
aaacatcatacaaaatttgacatcagaaggaggtttcccgctcttacaaaaatcccagaagtta
gcagcgatgggtaaaatctgtacagggttcacctttgaagctctaacttctattacagcatttg
atatgtcagtttccatggccttcattaatttggggatattcctattaacaaaatcacacatagg
tattttccattcagggtggatgtggggtgaaagaacaagctctagaagcactttaaagatgtaa
tcgccagtgattgttgtgactggagtgttgattgggtatagagattcttggtgcttcttttccc
tttctgatgcttcatcacaaacatccatgccttcacagtcatcgtcatcatcatcatcatcatc
ttcactgtccgacatttggtgggcactattattattagtgttcttgacactgttaccaattttc
ttgtacatttttttcaatagttaaaacattagcattatcagcactctcattcttcattatgttct
tcagtgaaagatccctctccacgttgttgtgtttggacacgtaagccttgacgatcttcctcca
tgtccttataaagtgtatgactgatgcatatttcttacccgtaacaggtttatgagtcagcgag
gcttggtagagcttgaaggcgtttgtccagtgattatatacagtcgtgtacaaggcctcgatgg
tttcctttgccagctcaggggatgtagttttactcccatatttcgaatccatatgtagtagttc
ttgtttgttgtcgagaatgtccttcaataaagggtccaacgtactcttatttaaaattttgtgt
tcgaattcatatatcagcgaacaagttttcatgtctttgtatgacacacaacatctcacaagac
agatgactgcagtcctcagaaacgtatcacttttcccacttctttctttcttcgtgtcaagaag
aggtataacgttcttgtaccagtaatcgagtatagattctgcattggctgcgatggtttcttta
acagcttggggtaaattagatacagttctgtcctctccttcaaaaatcactttgtcttcttcta
cctggcggcattgtcttttgtttgtgtttttgtttccagagagagtaattgcagaatttttctg
ttcaactaccagaactctataaagattttccacttttttggaagagagatttctggcggtcatct
tcaacaactggagccatgtctgtaccggagccaactttgttcacaactaaccgctcggtgaatg
cgtcttttaaagccattacggtccatgccatatctcaaaggttgccaatgaccaacataaatat
cggcacagcatacattgaattgcctaatactataaaggaaggactcgataacccgaaacctata
tactttgatcctttatctaaaaaatataaaccggatacttgcactgacgtgctggactcgttgg
acgtgtcgctagaggactatagaaaaagtataggatggcgtcctcgggggggattctttacagga
atagatgaccttttcaagacagtgattcaacaagaaaaacaagagaaaaataaaccaactcaag
caccagaaacagaaccaaaaccaggccatctcaagctccagatccagtcccagacccagttcc
taaaacaccaaccaatttctgtcctcctccacctaatcctctccctcctcctcctcctcctcct
cctaagccctcaagagaagaacggctaaagacgtcaaaaatacgtttaaacaaagctcttagtg
atattgttgaagccacaaacgagcgtgttgatgcgttgaaagagaaccaagcattaaatacaga
atatgacaagaaggataattacttccaggttttaaagtgctcgataacaccttctgtaccaaca
gctattataggcgcacacgtgaaacaggtggccaaaagtagcgaaatcgaactggccgtgaacg
aactcgatataaaaaataagtgctctttagtgtacaacgaaaatgagtcgttaaaatttttcag
ggaccatgagaaccttatactacaaattgccgtccagttattctctaggcacgataacaccaaa
tgcgtgggggcagaaatatgtgttaaaggcaacgaaaaaaacaagtttgttaacaaactggtgg
taaaaaaactccccaatgcaccatcctcatcttcaactgtgctagaaattagaggcgctaccag
aaatttactggagaataatttcaacaagggagaaaataacactgtcaatgaaaacaaggacatt

cctccttcagaacgagccaacctggacacgaccaaggcagaaatatcgcacgtcttttccactc
tacacagactggacactaaaaggaagcttttctttaaaggcaacacttttatcaacgaaaacc
aacattcgataataaattcaggtggacagaagttatagggtggacagaaagtgaagcatcaaaa
caaaccactaaatcgctagacaagccaacggacgacaatttattcgtgctaccccattctttca
ataatttggcagaccacttacgtttgaaatttaaaaacgtcctctataaaaatagtaccgcaca
tcccggcaaacgaaattactacaagactcaagagacgctaataaatccccagattgattcggcg
aaagagtacaagatggtctttgcagaaatcgacaagtgtttggatgttcttttggccataggga
agaatgacaaatacacaaaaagcactgtcatacaatatagaggaaagtttagaaggtatttaat
attctgctacgccttttatgctctaaataaggcaaaacattctcgcgcagtatcccctctacca
tttaatttctttaacctttctccttcatgtattgtcatggtccgtttctccattccgccagtt
ttttgtccacattgacgttcgtctatcaacacatgtttttccccatgggcacagccgccccatc
cgtctcagccaagcggctcatggatatcgattccgccctaatgaaaggaggaaaggggggtgggt
gtgagggattttggttcaccttcaaaaacaagtctccatacaagaacattggtgtctttcttag
gttttgctgaaatggctatgggaacaatgacggctctcttatctggtgtagaagtgcgtgtatc
tccagctctccaacaaaggatatctaaatccctagaaagatggtgtgattcagtcatctttata
tatttcacctttgttttattccacagattcagtggtgcgaaaaaagtatcactcgaatcggcgc
ttcgcctcatcatggggcagacgcacgcccacacaaataaggtgagggccgccaagagatgccg
aatagaagcagcggaaatggaaggtgtggaagaagaagaggcgggcctgacactctcttatgcc
catctattgggtcttccttactctatacaaaaagccctcggattacctgtccctaagataaacc
ctctcatgacagcatcttcttctcaatacaatttaggggatttttgtaggcgtggaacaacttct
aaaggctaagagagagtttccagccgaaggagaaaccgcaggatttctcggcatgtttgataat
ctagtgaaagattctattgacaaatactacggcgaaggagccttttcagacgtggttgaaaatg
taaaacaaggcatggaacaaaacacaccgtatgacacatcttcagcgttgatgacacctatccc
taaagcattctacgaagaagaaaaggatgttccacagcaggaagaaaattctacacaacaaaga
tatagattgaatagagacgtggaggaatatttaatggcttctcctatgaagatggtgtttgtgt
ctatactcgataaaactaaccaaaaagaacgtttcatgtctgttggggatattgcccttctggc
cgtgtggtgcaaaaggaacgtactgaaaaaggattggaacgaatacgctatcgctaaaggcaac
tacgaatggcttggtgctaaaatgtgcaaccatttacttttagctgatttagtgaattttggaa
tattaggtgacttgaaaataaccaataaacttgacacaaataccgacacctttcacagagacag
tgatagattaccctcagttgcagatcagaaaaaatttataaaaaacacatccctatctgatcga
aaacaattggcccttgttcactcgtgcgttaacgtgagcacccgaacccacgtaggaagagtga
ctgcaacatcatgggctgtcgatgcgcttcgtacctatacaagaggtgataaagacatgtttgc
cgccctatcttcatcgctggatatgtaccatcttgggcacacgaattcagctaattttgttcca
tattttagtagaaattacctatgtaacgaacaagagaatggattgtgggggtatactcgcagaa
cctctgaaaaattggccaaagaagaattgggaagaggacgtttaggggggcctgaataaggtagg
ggtggctaaaacagaactggctgctgcagccattgcaatttcttctgccttagatatgggggaa
gtagaagctgtaatggacgactcttctaaagtgagaaaaatagcctccacctgcttaaatgtta
atgcagccaaggtctcggccgccagagaaaaggcgagagaagctagtattaaacgtcttcttct
ggccactaatgcaccagcagctggttcatccagaaacagtaacaggtttctcctcaaagatttg
tgggggttcttttctgacccagacaagcgccagaagcttataaagggtgaagcagtttctgtac
tatgtcccaatacaggatttcttcatgctgctgttcctgattttgttattgagtattccttcga
aagtgaaacctctatagtgagattacgtttgagactgattaaacctgaaaaacaagacgaaatg
gtatgcccttcaacagctcccgaagctaataagaagaggaaattagtaaggaataatcaagacg
ctgtactgacgttggatgatgaagataacatcgttaaatacaacaaatatgatatggttgaaga

cgaggaagcgcgtgaaagattacgccaccaggacaaacaatcggttattgcagcccgtatcagt
aaagtgtgtgagcggaaaaatccaaagaaaaaacgtcgtttagaagaccctgaattgcaaagtg
tggatgaacaattgatacgggaactggctgccattgcctactgacgagatctaagattttgtaa
tatattatgtgtcttgcagacctaatatcctgaggattaataataataacaacaactgttcaaa
aatgagagacgatacttttaaccaagaaactgcggtgaaacttgtacgatggtatacagagtac
gattgttgttgcccattggttaaccgcgtggagcgccttctaggatcgttcggaggaggcgtgg
acgccacgtctgtgcgaagccgaccggctctttatgaagaagataagaagggagataaatgcat
accctttaggataacgtcccttattgagggtatacttttggaaagggctctaactaaacccgat
ttagctgctgcagcttttgatgtatcagaaaagctggtgtattgtagttgtaataacactcaag
gcaattttgatgtctcttcaatgaccatatggattgatggcaataatagtaaaaagtatgaagt
tacatgcccgtcatgcactgtcgagaaaattagtggaggtgccgaatctattcacaagaaaccc
atgtctcttcttgccttctttaacaatctggtagagaaagaagccttcgccgaaagaattgaac
tcaagaaattgtacctctccttactaacgggctcggcagccggaggaggaggtatgtacaagga
cagctcccaacaatcttccttcaacggctcttggacgtcgttgttgttccacacatctaaaaag
gacaagactcgtttagaggctgaagttttagtcagtaacaagataaaacacacatcaagattgc
agcctaggtgcgtctgttccgatctgttatatgccctatgctccaccactaacaactctgcatc
ttacgcgtacaaggcaagaaatttgtgtgttattgaaggtggggaatttttatatttaaatac
acaatctttgaagagaatggacctttcgactccaaaacagaccttcaatcattagttaataacg
agcctgtttctgagacaaattcatcggcactggccgcttcttcttcctctttagaagacgacga
tgattgttgtgatgatgatgacgatgatgatgatgatgaagacgaaaaaactaagaagaaacaa
cccaagaaacaaaccaagaaacaaaaaacaacaacatcaacacttccacctatcagcaaaacca
atcacgacaacatgttgatgaatgtacttaaaaaaggagctgttaatggaaaacggaaaatgat
ggattctttgtcaggaaaaaagggccaacactctaaaaaattgaaaacctccgctgctgctggt
ggtggtgcttcatccgacgttgttgcaggagaaaatgaggaagagaacaacccctcttcagtga
gtcctactaacaatagggatagaaaagactatgtgcttccatgccctcaaatagaagaagtcac
tatttttcacaacacaggatgaacaataacaagttggcagaaagtgtagtcaaacattctgtt
gttattaatggaaattgtttaaacttgtttgttactcaacacagaaaaaagtatatcctgcctc
acgaaaatattctttttgcccacctttagtccagcatgtaggatttaacaaatttcgcatttt
gactggcgtttcttgctttttgatagaattgaaattgtttttccgaccaatctgactctgtg
gtgttgagtaataatgctgcccattcggctattctaaggctattgtcatacataagagaaaact
cattgaagcgaagtgtgaggactgcttcggtgaaaggaatcgattttgtcgtgaaatcacagga
cactaatataggcatacctttaagcaacaaggaaataagagaacggcaattatgctcagcttca
accctgagtatgctagctggtttgggaaaatgatcaccgacccgggggtcattttacccgtttc
caaggatgttgtactctttggatcacgaggacaatcagatgtgggaataatgacactggaccct
cacgatttggatataaaaatcacatctaaacgcataggtgtggaagaaagactagctcaataca
atactctacctatggattttacacgggcaatggaaaaggaactaaataatagtagaaatatgaa
agagtcaatattcacgggaatatttttagacaccggttcggcaatcttcgaagacaacatgttc
aacggaggaggttcagctttgcgcttaattagatcccccgctttgaattctgctgtattttcaa
gcaagaactacatcatcaaacaattgcccaccataaccaaatctctaaggagaagtcaagctag
agataagcaagtggataaaacaagagagaagatagtggtggattctttcagcatacttagtgct
atagctgctcaagtaatgcacctcacagacggagagatgacgtacgtccccgatgggcactgcg
ttaatgttgtcatgtcagagaccaatgcttcgtccatctacttgatcataaacgaccccactgg
ttcgggatggaaaattatgcctaacaatttcaataaaacacttgaaatgagagacggtgtaata
gatagagtagaaacattagtggagtttgcgtgcaagtgcgtcgcatcatccttgattaaaggg

gcatggatttagtggatatgcaaagaactataaggtctatggatttcctccctccagcttcttc
tacttccaataatactcctagagtagcgataatgacatctggaagtagtactactacgggcatt
ggatccttgtccattcttgcagaagatggatcaacacaccaccaaatcaagctatcggaatata
ggactggattatccattactgaaaataatagagaggtgtcttttacggtagaaccttcaataga
cggcgttcaagcagagcatcctctatccccttctattcttcagtggttacctcctctagttaaa
aggccagaagtggtagcagcagcagcagcagcagtagtagaagaagaaaacggggacaataaac
cttctgataaagataacgaagacaagtacagtgatactgattttggtctaatgtccccgtcac
acctctaattacacccaagaaatggagagcgtgcaaaataaacgatcgggcaatgattagtagt
tggaaaaataatctagtgaaactccacaaatatgattggacaaataaaactacaaaggttgatt
attttgataagatggctgcctttgttgccctcatgacctttagaaaattccaagacatactagc
ggataactatgttcctcctcaaccccttctcagggaagtgaatacgcagtgaccatgtctaac
gtggctacactctttactgacgtgtacggttttgaatcgaatggaaataagccattgtttgccc
tagaacagctagaaaatgaaaccgggattgaaagcatatacgtcctaaatatcataggaaattc
ccctgatggtaattctgtcagggtcgtcagactggaaaaggaaatgagtttcctcttgaaggcg
aagcagtactttacagaaatggccatacctcctattaatgaaaaatgcaaatggacagataagg
ccccgtcatctgtaaaggagtacaagtattttgtgatctaacagcacccatttcaaagagacc
tagaaaagataacaacgacggcggtgtggagcattctgcgttgacttatacacctaggtgcata
taccacactgaacgttgtttagtccatctttactctgagccagaaaaaataacagaacacgtat
ctttcaacaaggatttgaacatattagaaattggaaaaaatattaccaaccaataccaaacaaa
ctacaaaagcatattcgaaattgtggacgttcccataattgtcgcctctatgtcatcaacaaaa
acaatgactgtaaacaactacataatttcaacaccttctgccacgaccaagtttgttcaggatc
cgccaaaaacagggaaacaacttctggcagttgaagaggtcagaaactttaaactcaaatctgt
tcttgttcctcctccttattttagggacaataagcgcaacacaactctttgttcacaaataact
gaacaaaattgcccgtcatcttctgaaggtgggcgtttttcatgtccatcagagtcacttattc
tcaagtactctaatctctctaaaaagcgcgcactggaagaaattgccccagagactgagactag
cattttgtcactagccatgtaacgtgtctcgatatatgacaatttaaacaacctcttaataaag
aggataattatcataatagaaaattaggtaaaatgggagtacagaagaatatcctggtcggtgg
tggtggtggtgtatcattgttattgggtgttgttacacttctaggaacagtaacagaaggagca
ccggcagtcccacctttttcatcgtcttcctattcctttactcctgaatcgagtgttttctggg
tcgaaggaaatcgtgttttaagtggaaccaagaaggacaccttaatcaacgttctggggaagaa
gattccttattatgctaattccatattcagacatgactgttctgaaactcgttctattcaatgg
ccagaaacttcccctttgggcttgaaccttattttctgttcatgtgcgagtcatgaacatcaac
accgtactcatgaaacaacagaacctgacgatttattgtgggacggatcaagaaaaactaccac
cataattctacctaaaaaatggtggtctgatgttgtgtggacatctttatggagggataacgac
cagaagtgtgggtgcgggcaggcatttgtttcttcctttacttctacgcagaaagaagtccaag
gggaatggcttgctgctcacactaacggaaagacatctgagggtgatactaattcagcctacct
tttcataagccttcaacgaactacactcaagcctatcatcactgacgtcacagaagataatatg
atgatgggaagaatgtcaggcacacccatgaaccctaaggatatgacatattttgtcaacgatt
tttcagacgatataggaagtactcctcagtgtcttgtatcgaattcggacatcctgaacaagag
ggaagaatggatagctgtttggggtgttgcagactctaaagacctcctaactaaacatcaactg
ggggagcgggaatatgggagcgaaggaagaagaagaaatcccggtgttgaggaggaagaagaag
agagagtggaagaagaagaagaagtagaagttgcgctaccttacattaagaaaagtggaaaact
tatcggacctcgtagaagacctttgacaacaacaacaaccactactactactactactactaat
cctattgttagagaggttgtggaagattttgattacgagtcttttaatgaaccagaaatctttg

gcagtaactcaaaacttcccttcattagatttcttgatcaaaaaaattggagacttggtatcat
gagcagagtttcttcttccatcgccaactttaaaattgaacaagagtcatcaaaagccttattt
tgtttggcagtctgggttggggatgaacataccccctaaattcagacttagtgtatggaagaact
ggaagccttttacttctgcacctattattgtccagaatgtaggttattcctctgatgttttctg
gcatgaaactcttagaagcaaaattgttgatcggtcaagggacctgatagaaacaaaagtgaca
aagaaaattggggaagattgggctaataaaaaacaaactgtagttgctatgtttatttcaggta
ttgtttgtataacagtaacagttatttctatattttcaattgtaatatattacaaaataaaaat
gcctaaattctaatacaatggtttattcattccttattcctactactagtgtatagcacatgaa
acaaagaaagagacacgcttcccttgttccttaaacaatatgtttgatacattttcacaataa
atttgtcaactttatccattacttcattaccttctcctgatatcttttccaaatccaccacagt
tccaacaccagtaaacatgctctttctgatatccaatgttgaatacttggcaaattcatgccct
tgcatttcagtcatggcaatgattatagcaccatacatttacatttttgaatagaggatatg
tggcaaagttgggagagaatatctttgtgggaagaccttcttcttcctcaaaagtacttttcag
tgctcctccagttctctttacatgcaaagaaaattgtaccctttttcattggatgaacttgtcca
cagaacaggacatcttcagacattgaaaagtggagattgttagtaggtgaaattgtctttcttg
gtgccaaaagactaatcgtatcagatggtgtttctgggttactttcaagaatactttgtcctt
aatggggaattcactaacaccagaagctgctgctattttgacatgaacattaacatacaatat
acattagcttcgtctgacatacacagccatccctttctgcatctacatggatatttctccct
tctcttgagctgtgcatgtggacgttacaatagacccatgtttcgatagaccacaagtttccgt
aggacgacaaaatttaccagtcctacgcaagttgtgctgcatactcttacatacaacatccatg
tatttattaaccttattttgattagatgtgtcactaacttttttcaaaataccttcaatgttat
ttgaaaactcatccgcgcttgttataggaagacgcaatttgacatctctttctcccccgtcctt
gcaatgggcaaaatctttagactcgtaacccagatttggtacatgagtagaattggtggtaatt
gattttgggcgtacatactgattcaggcacggattgggagatggcaaaaaggcgaatgaagaat
tcaaaacgttcaatttttcatacactaaaaatatataatggacataaaattggaacacaattgt
agccttgttatggggtgcaatatcaaacaacaacatttcaatagtggactcctttggtgcccta
ttcacaaaaactgggtgtattttgatatagtctttcttgttcttttgtttcgtctcatattctt
cacacaactttgaagagggctgttttacaataccactggctatagagtagtgtgtgattgaacg
aacgatagcagtgtatagctcaatcattttcttgttacataatttctcataatgtttagccaca
tctccattattaacaccaaagaaaaagtctctaatgtttttaagttctgtttctagcccatgtt
gctgaataaccttagcagtgttggccatccaagacgcattaaacgataaagattttcccgatct
acttaggatgaattcttcagcggcagctaccacatctttagaagggtcataggcagaatacatt
gttactcctccatctccattatagtcatcatccattgcagcggaagattctgccacctctctag
agacactaactgtatcgtggttaataaagtcgctcccattaaaatcagcaatgcctccaatagg
catagtataagccttgtctttactcatggcgcatttaatgttacacaagttaccccgttcaaca
gaatgattttgaataatgaacaagttttgaggattactattgcgggttacagcaacatacgcat
cctgggtagatgcaccagaaaggtcaacaatagtgtctctaaagaaagtgtgcccttggcaaga
atatatcgtcatggcttgactagattcaaatggaagatattgaacgtatacattgttcccaaat
cctccattcttgatatttggattaccgataatttgtcgtccttctttaatttctaggcatttt
gtcctagccgttccacgtacacaaatactgtcaattcaccattcttgtacattaaattagtaac
cactccagtgtccttagtcacgaacctttcctgagtgccatggatcattctattagatgtagtg
aaaatcacattttggccctgataaagagtaatagatttagccctagttaccttctttttctcat
ccaaaagaatcgtagttaatgccctaggagattcggctgccctcaaaattttcctgtctgtaaa
cttgtcaaaaagtggtgtactgcgagttttaatggcgttaattaattgttgccggtacttgtga

gagatggttttctcgtggctaatgtcagacagatccatattcaaaactacaaattttacttcca
ctcttaatgcgggcacagaattttcaactgccgcagccgctgcaagtgaagtagaagaggagga
tgaggaattgttattgttctttttagtttctttgatgatagaagtatccactaaattcgagact
gcattctttaaaacgtgtttttttagtgagtgtgattgcacccatcatcttgtcagttttggaag
aaattgcatcctttttcttgtttggctttagagttggccatgaggcgttcacacatatccttagg
ctgcccttgttcttttgttataaactcttcaatcttcttcctcctctcatttccctttgaaata
ctagtcaccatttgcatggccaacactatagattttttcagagtatttaagtgagttattgtgcc
tattgttaatgttgttattttcagtgtgagcagtttgaagcactctggaaatctttaaactacc
cttaacaccctccatcatttgaccattattgaaaacatcttcctttctgtacataagaaattcg
ttaaccattgaagggaagatgttatcaaaatattgcactgggtcaaagttgtccatgaaaacac
tcccatttctggatgatctaccggccattcgcgtatctaccaagtcttgaaggttggcagtcat
atcaaacaatgaaataccgtacattaacgaataagcgtgcttaataatgtctattctctttaac
gacgacatgatatatgttctcgaaagggcagaaagataggaagaatatgcaggtaataacatac
tcttccctacatctcctcctgttgatatgggcatagataataccctcaaccaatattcgtcatg
gagaggcttatctgcccctgctcctcctcctcctcctactcctgtgcttgtggcccattctgag
ggcgagtggaacaagcgtccagacagtgcagatttatcaagagaagggagttttccattatcaa
atttaacctgttgcattctcactagtgtatacaaataccacacacgggctcgtaccaaatctga
aggcttattttcagtgagccattttttccacacaacatgaaagtcaactaaatgacctacacga
tctagagtagatgcgtcacggtgcaatttatctggtatatcagttctatatatcatttctccac
tttcttcatcatcgtcatcacctcccccaccactacttttcataccagaacctccacttcctcc
gtcttccaaatccattgcttcttcttcatcgaaataacaatagtcctcgtcgtcctcataataa
gctgctgctgcagctgtacgtgatgatgcactattattattaccagtttctttcttcaagagaa
tagatcttatattttcagatttcgctagtagttctttgtatgttaaagtttcattctctaaaaa
tgaagcaactgtacgcaaattatctttaaagtaattagcgataggagtttcatccacaaagtct
tttccatctctaaagaaggcgttcctcacacacgctgccacagcagacgcacggttatgtgcag
aattgaatggtactgcatctatacacttgggatcagaaccgtgcacatacttgacgttcaatat
ctcttcatccattaaggaggtcagatgtgtatccttaaatctctctgataggctagaaacaata
tacaatttttctcggccactgtaatcaatttctctcatggctttggccatcatgtcgaacacaa
tactgcatacattttcagtgtacttttctagatttgcattgctcattttcttgagttcagatat
gtgagctagctctgaacatattccttcttctattagagcggaaagggggagagatttcatcata
tccaaagaagacttcttgtctgatacagaagttgttccacttcttatcttttcgtttgcatcag
aaatgctcttgcagcgtaaagagaatccattcaaagctcccgatgctctgtccttttcaagtgc
atttctttccatcagtttcaaaaatgcttcattgttaaagaaatcgtcatcttcttcgtcatca
aattcgtcacaattttcttctgccgtggattgggccggggaagaacttggtgccgtcacttcct
ttttattagaagcattaccacagtcttcaactttaatagcacttgagactaaattgttgttaac
tgacttgacgttattactcttcattaaaattgcttcatggcgtttattgacaatattccgcagg
ttacttgggttgccaaaaaagtctctagagcaaaacattttcagtatctttataaaaacttcag
tgtatagttttgcattatcttcttggccaaaattatcttgttcgactgacccttggaagaaggt
actcttttcttcctctttcttcgtctcagcttcatcgtcactttcttcttcttcttcctcctcc
tccccctcttcttcttcgttttcgtctccctcttcttcttcaccacctttctctttcttctctt
tcttcttcttcttgttcttgttgttcttgttgttcttctgaggtttttcttcctcactcgggtt
ctttattttcttctggatatggttagtgttatcttccactactgcagaattttgcctgttatct
ccaagtagtataacaaacactcctccgaaaggtatatctggcctgtgtttcgctatacgtaaaa
cagcatcaataaacaccaattccctgcaactggccattgtatactcatcgataacaaggaacct

taaatttgccaacacttgtatcactttccattttagtttatcggccacaaattggatttttct
agagttatttcatctttcctaaatataactggaatctcaaatcctttgttgagggtagaaaaca
cactttctgggtacatcaaggaagaacctcctggtttcttgcatgcacacatgagactctcctt
tgcagaattagatttgcaacacaggagcatgtctgcagatccagaatcaatcaagccgtggtcg
ttaagaactgcaattagttccttgaccattgtagttttcccagctccaggaagaccacccagat
ggaacacagatattcctcctccagcataactgacagaaatcgagttgcacagatacctcactgc
cagagactgttgcaaggaaacagtgctcaatacctttctggcctccttgcacgtatctgggagc
ttgaccgcagaagcagaggaggaggaggaagaagaagacccagaaggggatgccgctgcagctg
caactgcacttctgagcacctgggttctggtagccagaaaggggtatgatttgatgttgataac
tggaactggatttgaattattgttgttgttcttcttttgtttttttggtaactggctcttggccg
ctggagcacggacgtttactccccatgatgcactctcggtgaatgtcggacacgggggtaaaac
caatcctttttatactagttctacctccacgaggttgccagatggtacgatactttattaccaaa
aatggatcgtcctcttttgcttaagattttgtgtgtaacccttatcgttacgattgttacaata
tgtcttatggcatttttttatacatagcaattcctcatcttcttcaggcgccactgccgccgcta
ccgttgcagcggcaggagggatatcttcatctgctttatttggtacctacatagggtccttggt
gcagtcagaatttttttcagaagatatcgatttaccagaaaaacaaaagaaaaaggctgataag
gaaaaggcatcaaaaaccgataacaataacaacaacaacaagcgtcaacgccgccagacaagga
agcagagcccgcgcgccagaaacaggactttaagaaggaagaggaaattatccaaaagaaaaat
acacgtcaagagaaagaagactctaattaatcggcgcagtctcagaggaggaagaggattaaag
cgagcatctagaggcaaaggactgcttagagggattggaaaaaggacatttggattgtttttcc
tccccttttagaaaggatgtttcttttaattatcatgttgattatattagccccattaatattt
gttggggtgaatttcttcgtatacataaagaagaataactggttctctggggccattagtaatg
acactactattaactccacaaattctggaggagtaggaggattatgggataatgtttctgattc
agagtcagggacgtttccatctagggtacaatattccacatcaacgaaaaatttagtcaatata
gccatggatggagattatgcgacattagtaaggaacggaatgtccacaaatcaaaggccatatg
aaacctacaaagacgtggaggatagccagttctatttacatttttttccacgttagaaattttaa
acctttaaacggtgatgaaaataaagaccaccttgaaagggatgaaagttttgtgttgatcgaa
tcaccatattataatggagggtttttatcatacaatatcaacaacccaaatcccatttacaatt
ctactgaaaagccgtatattaacacggagataacttccatcgtcagcaccactggtacagatga
aaggttcttctgcctcgagaaggaatacgtcgaagatggtgaagaaggagttacagaaaatagg
tactttttacgccacatggcaagtaattatgttgtaaaggctagtttcaaatcagtcatgccca
ctattgaaattagtgatctgactggcacatacaataataagaatagtgttaaaattaagcctgt
aactggtacttctataatttatgaaaaattacaaggaacaaaataaaatacattttaaagtatt
atgtttattgttttatttcttctaaaattttattttaggtatcctcttgaatatggaacgacgg
gtgggggtttttttcttacccttctttggtttttggttagttttcttggccccaccatctggtg
tttttggtcctcctttcccctttcctttttccaaaggctttcttgaatgcaccgcccactcctgg
tattgatcccagcgcagaagcgatcgtgccggccagtgctgtgcccgccaggagagatgctaag
agaggagccttgattattattacagcaataacaatgacaattattgttattaaaagtaaaccaa
aaagtatattttttcctcctcctaaagacgccaccaatggcgaagccatcttggctatcttatt
gttattattgttattgttattatccccttccatggttgttgcacataataatagtgtgtattat
aacattaaaagaaacatataaattttggctcaaggcaccttcaaggctgtgaaaatattacatg
cagataatgttgattctgaattcagggttgtgatttttggtcgcacgtttactgacgtccaaca
taggcatatggatagtacaaattgtcttgaattcctcatcttctttgtcctctgtaccatcctg
gggttttgctgggaacaatgtcctatatgttccctttttttatatagtaattattcgggtttgaa

tacaagtagttatggaaatggagatgcgaaccactaaaagcttgtatatttatcaaaaaattca
agtcaactttctcgcactcttctcattttgggtaatatctctcccgttaatgacgtagataatt
gttgcattcaaagcactgatgtgcttttcatgatattcgtttaaaacaattttataatgatttt
taaagggataaaaatttggttacaaatagttaaaaatatgtaaccatgcagtacaataaaaggt
gatcaactggtttttcatttctatatcgtaatgaaaattttgcattacgcgtattctttgtaac
tatatcggaatatacttcatcccatacagcagatgaacctctttttttcaggcttaattgcgata
tccttttcagattccatttcgtggaagatctttacaaagcaagatttatccgtttgattattga
acaaaaaatcgagccgtttttttcccattatctacttcaaaattctcaattgttagagaaaaatc
cctcttccagactacaatatcttgggccttagccagtgttctgattagcccgtgatttattttt
cctcccatagaaggagataaattgtagctggtttcatacctcccttggtcatcacattttactc
taaaaattgccactgtaaaatcactcatgtgaagattttcaatcaattgaggaattctcttccc
tatatcaatttcaacgctagctggcgtttttagtacgtcccatgcaaacgcattgaggctcttg
ttcggtacaaaaaaagttaatgccgatttcggatcagggaaaacatccgcagccacctcagtta
cccaggcactaatgttgtcttcagacattggtaaagttgctgctgctttttcatttattagtgc
tctaattttccctctgttgccctgcattttattattgaatacagtctctccgtagggtttaata
tgtcctccatacaaatctaccccagccatggaagcagttgcagtatctgacgacctcgtgtcca
aaacatttgtcaccctagaggaaatttccccatctttaaatagttctgcaagtctcgcctccat
tattattattttaatgtgtacaataaaaacaattaatttgaaacaaaaggtaataacaaaata
aaatacataagaaagattcaaaaagttttattcattatccttatcacacacatatttttgacg
gacaagtacattaacattatcaacattcaacagaccactaccatcattcattacaacaacagaa
aatggggaagttttgttgcacaagaacttccatacatcattcttctttacatgaatcaagaaag
tattccaatcatcccattgctcatcgagcctgtaccccaaccatgggtttacaaatagactatt
aaatttatcccatgaacagtattcctggagagaaatgaaggggtctttcccgtccaggtcattt
ggtccaacattttccactacctttacttccccgagtccaattagattgactgtcgtcttttctt
ttgtctcgatcgcacactcgccaatggcagccaacaaggaatcgggaagatcactcctcatttt
ctttggcggtgaagaaggagaagtgacctgaagaggcgctggtctcttattattatttgttgc
agtttatttccttcggcaattgcagtgttcttgagtttcaagaaatagtcgataatgtaaaact
cttcaccctctcccagttcttcatacatcacatcaccagatgtcctgatggcttcttccaccat
agccaaagaaggtgctccacattcaatcaaatccatcagcagtttcttccctcctctccccatt
gcgacgagtttgttggcgtacctgcaccagggacattttctgttctgcggcttgtccggaccaa
gagtagacatcatgtacttggatttgcagcaatcacattctacacatacagcaactggaatatc
aatattgccctcattcaatagcttgtagagaagacaagtcttcccatacaaaatccccacagcc
ttcgctgcagctgaaacagcaatttcattgtctttatctgttgggggaagattgagagctccaa
tgatatcactagccatctcctttactttgtcatcaattttcttggcattcaaaagattgagggt
tgtgggtgatgcagttttggcacgaggatctctggtcaatttattgtcccagtgatagaccatt
ggtttgcccatcctgtagtttcccttgcatctcaagcacaatttttcatcattatcatattcca
tctttagagattttacaccacatcttttgcatatatatttcaagtccttctctgataggtcaag
tcgacgtacagggaacgtctcatatttctccccaatctttgagtcgccagcctttgtcaagatt
ctcagagccaaaaagtgttctagtttattggtaaagttcttgtcaactccaatcttattggccc
atgtaattaagcacctcaaaaatactacaccggaaagattattctcgatctctccatcggcagt
tccttcaagaatagagggcaaaataatatccaaatcagtctcggaactgctattctgctcttgg
taatactttgtgaatgccatttttgcaatagagaggatcatgtttgaagtgttggagggtactt
cacaggaggcagcccaagaggcggctgcacacacagatttgattgctgcataacctccttctgg
cagactcagacgtacatcatacaaagttcccaatttttgaatcacagtctccaagtcgataaac

atcatttctgccacatcttctcgtcctccaaatagatcgctggccaattcttctcgaagcactt
ccttctggtcatctgatccacaaaagactgggatggcaaaatatctgttggaggtttttgacgt
ctccaaaagcacatcttcaacatttctagtaatctttagacatgataccaatttcttgatgaaa
tcaaagcactgtccaaactcgaacctcttgtattcgtccagtgataataatcctcctcctgtat
atgaattggagaaactaaatgactggtggaagatttgccagcttcttccaaggcatcattggg
cacatagaaccacataaacatggaggattgttcaggtgtgagatctgtattgttaatcacttgg
agtgcatcatagctagcagatttcatgtgctttccttctttaggaaggcatattctgccttgt
ttccgcattcagttcccagatcagaaaggatggtagagaccctcgagcacactcctgcattatt
gcctgtaacaaaggcataaatagccccaatttcatcaggcacagcactgcaagcgtacatcatt
gattgttccttgatcatcattttgaagagtttttcagcaaacatcttggcccagtgtacgcgag
catacttgcgagacaacttggtttttcttccttgatcagatgtagcttcaacacttgccatcaa
ttttgtacaacccttacaatcacagaggtttgtatggtcaattccaaaatcgtccaacgactgg
aaacacgcgttcatgtgcagtgccagagtagaggcggcatttcctccccctgtaattgactggg
ccctttgagtcactacagccagtttgctgataccaccaccaccgctagaagactgttgttgttg
ttgctgctgtggattaaaggtttgcttagagcaacaaaaggcactatcatatgcacaattcaag
aacacgctacgcttgtggttgttatatcttcctcctccctgtacaactcttccaaaatctaaag
gtgccatatttaaaaccctagacttgaaagattcacgttgagcgtcgccatactttttcaaatcc
tgcaggctctgatcttccttcactttcaataaaaacattcaattcttcttggttgatttccctc
aagatggggaaggcactaaattgatcattcaggttcttgaagagggaaatagattgcctcataa
taaaggttgcgatattcttgcgcacattgcacgatcttctgtcaaaggattcaaaaagttgcca
tctgttgttgtcgtatccagaaaaattgatcacatcttcttggcacaattcactctggttactg
ccactacatctagagaaattgaggatgcgtagccaacaatcattctccactagcgccttcctca
tgtcaagaatagtcaagggtttttgtccccaagtcttcatgatgaattcttcttctggtgcagt
ttccgaatcgtacgagttggcacataattttccagtgtcactattggcaatacgcatatcctca
tcggtgaacacaaaaaatgaggatggggaatttttcatccctggtaatgccatgcattttcttga
atgcaggaatcatgttgttaaacacgtacatcagggaagaatgaagagcttcactgccaccgcc
gccgcctccgcacacttcggtcttgcgcatatgcttcacaaaatctccccccttcccactctgaa
cagtctggtgcacgcacaacaggtccactttcaatggagtgtttgtcgaaatcattgtagtaaa
ctatagcattttgaatgcctggagcaatcacattgagtgtagccatgatgccagaacaactatc
agccacagccgatttcacacttcccattgaaccactaatatccatcaacgagatacttagacca
gtaggttttgcagttggagtatctgtatcagcagcagcagctttcttagtccatccatcttcta
aaagttcaatcttggaggaaggtgcattttccatcaaagagttcaaaaaagatgcagatttcac
agtagaactagaactcttgctcatggtggaagaagcgcttgaacggatacggcccaacattgta
gtatatctttatggtagaagttgatgttgacggtaggaaacgatctcgacaactgtatgctgag
gtgcctccgcaggccgcttttataccccttttcgtgtgacgtcaattgacgctcgtacgtataaa
cagggtctatttttagagatgacatttttcaaaaacgtttatataatggtgcaatttcctatta
ctcaaactagtcatatccggctaagacaggatgttttttggccaatacgtttctggcgcaatc
tgacattggcccgacccagcggtccacccttcgaacttgacatcaggccgacccagcggtccac
cccctaaactggagtgagctgaaaaattttcaaaagtttttgagatgaagaagagggtgaaaa
gtgggtacgtactatacactcagagggtggcagatcggccctgttccagaaatggctgtccaga
aatctgggtcggacagattccagaaacgtttctaaccatttctggaacagtcatttctggaaag
ggttacaatttcttataactggtgcattatttctagcatatattctagcccctttgtgcaatct
gacattgggtcgacccagcggtccacccttcgaacttgacatcaggccgacccagcggtccacc
ccctaaactggagtgagctcaaaaaatttttgaaaagtttttgaaacgaggatgagggtgaaaa

cacctgttagaaagtggttgctggtcggtagcatccgttcactgctgttccagaaatggctgtc
cagaaatcagattccagaaacgtctataaccatttctggaacagtcatttctggaaagggttac
aatttcttagatgtggtacaataacttaccatacatttctggtatcttcgtgcaatctgacatt
gggccgacccagcggtccacccttcgaacttgacatcaggccgacccagcggtccaccctctaa
actggagtgagctcaaaaaatttttcaaaagtttttgaatcgagtaagagggtgaaaacaccct
cttgaaaggcacgatctggatggtgtcgtccgttcactgctgttccagaaatggctgtccagaa
atctgggtcggccagattccagaaacgtttctaaccatttctggaacagtcatttctggaaaga
gttacaatttcttagatttggtacaataacttaccatacatttctggcaccttcgtgcaatctg
acattgggtcgacccagcggtccacccttcgaacttgacatcaggccgacccagcggtccaccc
tctaaactggagtgagctcaaaaaatttttcaaaagtttttgaatcgagaaagagggtgaaaac
ctggtaggagaaggtacgcacgcatactggcggcacatgcctccagaaatggctgttccagaaa
tctaggtcgaccagaatccagaaacgtttatagccgtttctggaacagccatttctggaaagtg
ttatattttctcgcatcttgtacattaacttgattatggtatagtacacctctctgcaatctga
cactgggtcgacccagaggtccaccctcggaacttgacatcaggccgacccagcggtccaccct
ctaaactcgagcgaccctaaaaaatttttcaaaagtttttgagacggaggaagagggtgaaaac
ccttgcaacagaggggtataaaggaggtaccctcgcacaccttagacacacaacctcatcac
cctccgtccaatcaacatcatcaccccatctctaaataatccatcatgtatatcttcgtcgaag
gttccccctcacagggaagagttcatggatgtccaagttgatagatacaggatcatgtggaat
gtctttcctcaatttcttcgtatgaacacttctgactactacaactggcctgccgaaatcggg
acagaacatctccagttaggtttcagagaaaccagagtggtggatggaatgtttgaacctgtcc
taaagacctttgtcgactcgtggaagaaagagcaaggaaaagagagtttgaaggaatatctgga
ctacaacggccaagtcatggagatctacatcgcagaatggttgagacaaaggccactagccttc
cacgtgtttacctatacagatgaagctgtcaagagtggattcttgaacgaggaggatctagata
tggatactgcaaccaagtggatggctgaaattattagagagaagaggggcaatattcaagaaat
aaaagtgaccccctagagtagtcttcaatggcaatgtttgtagtgcatgtttctctaacactaag
agaaacttgtataactttggaacaaactataacaatgttgtacattgtgatttgttgtgccctt
ttgcaaggcataggattgtacatttcttataaaaaataaaacaatatataaaattcagttgtat
ttttattgctcaaataagttactacacccaattctccccctctctagtgagagatcccaatca
ctttctactgtcactgttgctgctgtttgttgttcctcttcttcctcctcttcttcctcttcat
cttcatcttctttagttcgttcttcattatatgctttaattatcctcaatacatttaaaatggt
gcgctcttctcggttaaacttctgattctttccctcaccaccaacatcgaccctacgtccatcc
tggtatactgtttcaccatcaccagaaaaaattctaatagacggatttttttatagaacgtacaa
caatgtccactaatgattctggttctggtgctaactgatcatcgccaataaaagataaaacaac
tccagaaacacatggataatattgcacattgaattgatcatataaagtattgaagatgataggg
taagatgttggcccgtaggcagacatgatcaaacggggcctgaaaccaccctcatctctcattg
tacaataatttgaacacatgaatctgattcctctttctgtaccagaatataggggtgtgtcttc
attgaatctgtttgtgaggaaaaagtctcgtgcagataatgcaaacacgagacacgtactttg
tctcctattcttcctatttcgtttccatattcttcccttgcttcttcatcgatagcatccaatt
tgttggtttcctctagtttttcttctaggtatttcctccttgcggcgtcaaggtatacctcgtt
tggcatatggatgaccttattacgttcatgcttaccgtccatttttttcggccgaatgtgtaaca
actgcattcaaaaataatgccatgcattgatacgtctttagaatgtctcggaatgtcatatttc
tttgtgcgtagtacacaaaatgtggcacttttcaagccaccacctctacatcccagtgaggcgg
tggaggtggacgtcatatttaactggaatatgtgtaataatatagtccaattcttaaaaaatca
atagaccaataaaacaacaaaaaacattttgtaatttatttattcaactaaaatctcatcaaat

aatcttctcagcaataaacttacatttcatatcacagtgtacaatactattcgtgcatttacca
acacggtacacgtaattcttactcgtcgagaaacatgaactacaaatattccctttgtacacat
ttctggggataacttcaatctttacaaacaacgctctctcctccttgagaacgtcaataaacca
gttatgtgcatcattcaattcttgaataattggttccatttcttgacatcttcttccctgtaa
accatcacatggaaaactggcttgtatttgacccactcggcgacacatgctttgaattcttctc
catacatctccaaatactccctcaatccaattttgattctggaaactgttctgtgtcgtgcct
ccacatgcctgtgaaaaagttcaatgatgatccaaacattccatccacgagcctgggacgttcc
tggtaatcggatgcacgcaaatgctcttgaatggtccatgggaagatgggtaaatagtccctat
aattggtgtacatgaagttgaggaaagactgtttgccctttccggcagttctcatgttatccac
ccatgaagttttcccagttaggggagatccttcgacgaatacaatcatgttgaagggtaagact
tgtctaactctctcgagcgagctctctctctttatagaggcaaaaatgtagttgccagccacc
accttaaaattcgtgcattaataatatataattttatattttgtatcattcaaccgtagtcca
tctaagagtcaacatgtcctcctcctcgtcgtcgtcgttctcgttccgcatctccacctaccag
acctttctcaaggctctggctcacccagatctggtggacaagattacccagaaatgtgacgaga
caggaagaaaccagaagtgccccatccagtttctggccgacatctcgcacctgatccaaggaga
aagaaatggaggaaatctgttccctttgcacccgttcaagaaccaaccacatctggaaccaaga
atagtgggaagtcttcacgggagaacattggacaatgacattgaagaatcatactgttatttg
tcaaggatctgtataatggagtattttcctatgtgaacggcgtcaaggagttacagggcgttct
ggacaagaaaatatctggatctggatctggagaatcctcctcttctagagctcccctgatccca
ataactgatgtggatttgctgtacattttcggtactttggtagttcttcccccagatctaaag
cgtaccgagtcatcactgaagctgttctagcactccccttcaatgaattcagtaacaactggcc
tcctacaaatatcaaaggagcatacgtgtctagagatttcaggatgtttaatctgttggccggt
ttagatcatatagaaggagaagttgggggagaaagtgaatgggaatccatacacgcatctgtcg
tcaagcgaatggtcaccattatgcgcaacaaagctgagaagaaacctccatcaacatctagaat
ttttagagtgtatgtggctgaaccagttaatgatgcagtgacaaagatccctatacgtgtactc
agtaaattattcggttcaagactcgccggtattctccagaaagtgtactcttattcaatgctaa
atcttccatatctcctgtcttcaaattctatagacatcaagcaaggagtaaaaggaattacatt
atctataccatctgcaagaaaattgggattctatttactccaaaaggatacaacattacaatct
tccctatcacaagatgttgccgactgcatagtttcaatcaacgctggtattattggtgatgatt
tctctgaaaaaatacgacagtgcattgaggagaaaaacaagccagaaaactgttgtatgtgctt
ttgtgaaattgacaagacgcccgatttttcttatagtgaacatgtggcaaggcacaatttcttc
cccgtccacgcattctcctcatcacatgatgacaagtgttgtggagcaaagatttgttccgaat
gtatattcccgtacatcatttccctgtatgagaaaatgactggtgtggcaggtgtaaaagttgt
agatttgttccagtgccctggttgtaaaagtggcatgctcaatctaaagggaagatgttacgag
ttttcaaatttgtgtaagagaatgatactaccatacacatcgactcattgttcttctctctttg
atgctaccataaatcgcgcagaggcttgtttttactccctagagtttctccagtatgattttga
aactgcgaggagaattgcgcatggagctaaagacattccccatgtttacaataaggtagtaaag
aatgtaaaagatttggatagactgtgtgccttgtactgttacaaatgcgtatctcctgtcgtat
gtgatgagccgaatgaaagtacggactatgaaatggtggatgtaactccccctctaattaatct
taccgagattgttgattcggaagagtatgatgatggtcccggaaatcatatgtggccagcaaaa
tttacctgtaactttattgcgggttccagtggagaaacacccaccattagtacgtgcagagatg
ctgtaacttttctaggaagagcaccgaggaagaaaatggcaggatgggatgatcaatcggcagt
ggggcaagccattatagcgctagccaactggagaaagagtggggaattgcccaaaaatatgttt
gatttactagaaggggtaaatgccgtactttatagaggcgacagtttcttgttacgtgcgataa

actacccctgtgttattggtagatccatgagccctagtctggaactcgttaaaagaaaggtgaa
taaaattgctttaataaaggccttcttccacgagaaaagggtgcgtccagacgcatctaaaaag
ttacttgaatgggcagaactattagtcaaaagttatctcatggaagttttacttcagacgccag
aatgtgtcatacaccgcgcccattcatttgtaggcaaaactctcctcattactgacgaattggt
tcacatgcgtccagatgatgccacaagaaacgcctatatccagaacctaaatgcggccagacag
aatgcggctgctgcagcctcattttctgggtcgctccccaaacctgaatttgtcccctgcaaag
aaaggacgattgaatggatgtatgaaaaggacaatgatgatgttagagttgtaaattgtccttc
atgtaaaaaggctatccagaaatatggaggttgtgtgaatgtgttttgtgaatgtggaacaaac
atgtgctggatatgtgaagagaaggtttctcctgctgattctaatcattgtgtggagaaacaca
ggattgtttatagtaactgtgttagggttaaatatgccttagaaagtatgtacgggtttgagat
ttgtaccatgaaaaatgtagaagaaggagttaaaaattattatgtaatggagaatggatttttc
tttgatgtacaagaaatggttgctaagaaataataaaacacttgtgaaatgttataacttgttt
tattgtccattatattcaacaaactttaatttctttgcatgccttgttcaaaagggcaacataa
tcgccttctttctcctccctctttctctttgttcctccactgcttgtggctacttcttcttctt
ccccacgttcctcgttgtacgtctcaattgccttaactgtgttgaaaatcatgcgacgctctcg
gcgcagacgcttcctttcctcctctggtaatgtttcttcctttttattatgtccttgccttct
ttagaagaggggtcaatcactttttatttctacaccatcttgatatactttttccattatccatga
agattttgatgtctgatccagtaacctctctgaccatctccatcaacaacctttcattcggagg
atcgatgaatgatatttcattaccctttccggatgcaatgaaatggttattaaaatggtttcgt
atactatctctgatgcatataggtgcatctgagccagttatggacatgaccaatctgggcctac
gttcatccacgtccctctttctgtagtcgtccgaagagacgtatctgattccttcttctgtggc
agagtaaaaggaaccatttccattggttctgcacaacaaggagaaatctcggtcagaaaaggca
aagaccagacaggcaccgccgtcatatctggatgatgcaattgaggactttttatgcacatctc
gaagagaaatcctttgttcttctgaggcagcatatagctttttggattctttaagtttttcttc
cagaacacttctttcgctaactagaaggttgacttcatttttttgcaaatctgatttccttttcg
aaattatctaattttgattactcttttctagcgtgtatacaactgcagcattgaaaaggcatg
acatgtaatttccaatgcttttagctgacgggtgtgtgctgttgtagaaaagtaatccaagttt
atccattggtacaattaggcacatatttgaaaaactgccaactatggggtttgcgatacttaag
ccattgtacgttaacctatcacagatatcatacactgatacacgagaagaggtaccaccgtgtt
gcactaacacttgttccatattcttaagagtacgggtacaatttctaggagaaaacccacaccc
tactaagaagtctgttgtgattatcatataatcacatttcttaacggccgcatttgaaagacgt
tcatgtatgattctgtatagggggtgaaaatttaaagttcttgtcattaactagagccttttctt
ccacaattttgttcatttcctggaatgggcttggtagtgattcgttccatttttttccataatt
agggtcttcatcttctaagaaactaccttgttttgggagactggatgtgtattttagtccaatc
tgaagcgtaggatctcctgttaattgtctcaatctttcgttaccgttcttgatgtggagtactt
ccttgtctctaagacgaactataaaatgtccttcctttagctcacttctcctgacactcttgac
tgatttgaatgttaatagtacccgttcataattttcggtattttccccagacaaacaaatacca
ttttttacttttaattgtttaaagtcttccattttttataccgtgtaaccctaacaatggtgtgc
cagtataataaatctacggaccatgctcttgatctgggaagtgtactcgtgatttctgtagaaa
ttgctatttcctattcattatttccattacaggaaaagatgataaaatgaagacgtgcgatgag
atatgtgtttttttcaatatcgtatatcaaatttgggcaaattacatatttttttagcgtcaacac
tcatcttcatgcaagactttttggagtgaaattactaaaaaaggctatatttcatgtacaacat
aaaacacatgtgcagacattaaattggtttgctcaccccccccaacatcgttttcatcaactctt
tcctcacctctatataaaaccagcctgcaccccctcggaacccacagtcgacttgtaccctccg

tcgagatgacatctccagctccatcaccctcttccacccccaaatccagttgtaccactattgt
aaaccgatgtggtttcctccttgacaacaacaaggaagtggtcatctacgacaccaattccaaa
ttcaagtgtgaacccaaaaatctggaactaattggtgtactttctggagtctctgataatgttg
ttacccagatatcccccgaccagatatttgtgggaacatatatggtcaaatataactggtctaa
atctggtcatgaacgcttcagtgacatgagtaacaactgtctggacaatattacacgcccttca
gaagtgattgaaagtgtgataaagaaaacgtccagcgactttaaaatgaagtacacacgttcct
tgatggaccacaccgagaaatactatttttctggtgaccaaaaattgagcaaaattagtagttg
gtgtacaaccccctataacgacagtgggtatgcaactccgtctagaaaactttgtcaaggaagaa
catgaaactgtcgtggtgcacaacccttctggaatgactggattcaacatatttaatagttccc
ccgtgtattttgaagtgcacaatgagatggacgccctaatttttatggcggctttcttgaagca
caatagtttatggggagaaattaacgccaatatggacttgtacacgtttgattatgcgggtgct
tttctggacgaaagatggtgccaccacgagaagagtttttctgtcgtccgagcacaacttatca
actcgtattacaagtgcaggagaaaaatcatgcaagccctggacaataactacaacaacaagaa
taagaagaggaagaatgttggtggagcacctgcgttcacatttatgagcggggacggagaggga
ggaaaggaagccctagaagctagtttcgatgtgattggggaacaagaggaggaagatttggtg
ttgattcaacaccatgcccccattcttcagccatgcaactaaaactggacaatgaaggaaacta
tggatgtattgcctgcttcgcatcaatgttctttgtattggagaacccaggtgatgaatcttcc
ttcatatcaacggatgcctctaaaattggacaagcgcaagcatggatagatgaacgactacgaa
acaatgaaaatggaggagaagaaaataatgtctttaaaaagaccttccatatgctggctgatat
tacccaaaaggctcatgaaactgcctattccaataccatcccacttggacccaatggcaggcag
tggaattggcctactcacactgtggaacctattgcccatgaatttgttacccattctctagtaa
acacattgaaaaatctaggggatagaaaacttccccgattcaattttgatatcttgtacaactt
gcttaatccatttggaaaaatgttgctagtgtttattcaaaattgtcacattttaactggacat
aaaaacaatgaaaatgtggtgcctcgaggttctgcttctgggaagtggtggactattaatttg
tgggtgtgaacatgtggacttttcaagtaacaaaatgtaaagttgaaaaggatagaaaaatatc
cgatttggcctgtatggaaactctccctcgtctacctaatccaggaagcactaccgtcgatgac
agaatagtttttaagggattctgtagaggggaaaatctagggagtgtaggtgaagtcgtatccg
acattacacagagtgtcaagaatttttgtctcatggttgaaaataggaaatttagtgtggataa
agaaactggtttcatctcttcagaatcgatagtgtctgatcccttcttttcactagaagtgact
ggctgtagatctaatcgtgcccaagatactattaataatggccgagttagtgctcgtgtaatga
ggatcctaaagtcacgtgaaggtgctcgtgtatggttggccaaggatgaaaatgccatcatctt
tgaaaacgttaaccacgatacggccatctctacggacgctatggagcgagctatagggcagcac
aagatactgtactatgatattgaaacaacagataaagatttcaccgacaaaaaatcagtcatca
catctattgggttctgtttgtgtacgggaggcgatatgacacatggaggagagagaggagtatt
tggactggttgcacctggatccgacgtggaaaaggtgaaagagactataataaattcgtacgat
cctgaagaaaaggaagacattatgaaacagtgccctcaagtgattgaaattttcaccaacgagt
ttgaaatgttgcttggttttggaaagtatatagataaagtgaagcctcacgtgattagtgggtg
gaacaatgtagcttttgacgacccctttgtctttactcgtatcgtcaaacatttgagtgatcac
accaaagacatgtcttattgtgtagcagatgcatctacagcagaatctgtccttcctagagcaa
cagaaggaggaggaggagaaactccatatagattgagcacccctcaagaaagaatacaactagc
aagcactggtattttcaataaattgggaaaatttgtagacaagaaaactggcatgttgaaacct
gaaatgactgcagatttattggccggggcagaaagtcaggccaataccaagtttaaggaacgca
acaagttatcctccagtaataaaggatcagcaggatggttccagaaaattattggcggtatgtg
cagtgctattcggttggatctcatgaaagtgtgcgaaaaggcctataaagaatccctctctgaa

tttaatttgaacgccgtgctcgccaaagtgagtagtgtcggcgacaaggttaaaaatgtaaaag
atgaagtagacctacactttcatctattgggattcttgaagctgaagaaggcccaggatcaggc
aaaagtacacgtctattgttgcaaggatgcctacttgactggtatagtttctacctccatcaac
aaggaaggggagatttttaggctgtgtatggactctgctttaaccgaggcggtcgtgacagcca
acctggccactcctctatgtataggagaaggagcaatctgtagaaatatgggagaagaaagggc
agatagaagaggtgtgggagtaagaagacactctattgccacagacacaaagggaggtatggtg
agtcaacctatcgtcaatcatgttccctatcaaacgattgacatgacaagtttgtacccgatga
ccatgtgtcagaataatctgtgcaccactacctttgtgacccatcgacaaattatgcaactgag
ggatagattggtacttgaaaaaatgaaaaacaaaaccaccgactctttattgttgttggacgtt
attgacgagtgcaatcagattgtgttgtccgagtacagacccattgatattgcagtcgcatcat
ggaagaatagcaactctaatagacaaactccaattactcgcatagaggaaagtttgggtctaag
attcatagaaaatttggatgccgagaagacaaataataaaacgtggtgcaccaatacatctccc
aatatgaatgtcactgccgcaggtatggattacttccccgagattgtgtgtgacattaatatgc
agtttgcggccaaggtgaatgatgatatgcatatagccccagcaagtttagagtatatgcttca
agtattgcccataatgttaatcgacagaccgtacattggcgcacacataacagctggaaaatgt
cgtacattggaggatattctctcagaactcgaaaaggacttttctgttgaaaaagatgaggaaa
ttataagaacccattggacatttaagggtcaaaaacaatacgatttctgtcatagtcctgtgac
ccaaatggctcgtcacattattgaatctacgggaagaaatatccgtgattatgaaggcaatgaa
aaattcgagaggttggttagcttatcagacagaatttatcgccgcgttggcgcatttgattcgg
ccaatgatccagctgttagactgtggtcttctcgtctaatcaatgttggaatgttggttaggac
atggaacgtaaaaactgacattcttaagggaatcatccctcaaatgcaagccacttacagagcc
gatcgagttgtgatgcagaacaaggccaaggagtttgccaagatgggagacatgaaaagagctg
gtctaaacaaagttggacaaaatattatgaagctcggtatgaattccatgtatggtcatttggc
cctaagagcacgttcgagccgtaaagagtttgcgtctggatctgccaatactgcctcaagtatt
tccaacatgtcagccaccggaggaattggaggaggcacaaggcactcggtgacggccaatcaga
ttacagaaaatgctcgatgtgtatttggcaatattggttgtggattacagatggctcttcctgg
tactaagcagacgtacggggatacagattctgtattctgcgtgcataatattgtaggtgatgga
ggaatgataccagaatatgatgaacaaactggcaaatattattatgtgatggatattgctctaa
aaaataaaatggctgcaattattcccatcctagtcaactcgttaacaaagggcatccagtttgt
agagcgccgagacgctggtgtgggcatgatgaatatcgcccatgaacgtctagctgtcgctggt
cttttgtttgccaagaaaacataccatatgcttcactttaatgaaaatagtgcagcgttcaatg
acatgataaaattgaaatcaaccgataacaataataagtttgcatcctttatcaagagacctag
ccacgcagatgggtatgttgttccccataatccttcattgattcttagagcggccgaaggaccc
gctggtaagaaattgaaaagcttttttggaagaggaaggaattcatgacgagaagagtatggagg
aatggtttacctcttcacctacgtggatggccatggatgcttctgttatcaacaacttgtatgc
ctcacaaattgtaggggtggagaagggtaactggattgacgccatgacttcccgccccatagaa
gcgggtacagaaatgatggaggcggtgacgcaagcgaatgcagctttcaccccttacaaaaagg
gagcctttgtgaagaagggaattacacccaccaccaaactaaagggtctccaatcattgattgc
aagatttttaccaaaaatagaggaaaagaaaagctgttatttggatgtgatgaagaatcatgtg
gagaattttgcatcccatataacaaatcctgctatgatgattactagttcccgagtcaacaagt
ttgatacgtcaaaagaacagagtagacctaatcctctagctctagcgataaataaccacctgaa
cccttcttcagaaatttcattggggcagaaatttaagacggtgacatcagtttcttcttggagt
ctttcggcagaggaaggggaagtccctgctggttattttaacgctggtagcgtgcgttgggatg
ccaccaacatgaagggaagtgttcctgcattttcagtcaagaatttatctgttgtgcccaacgc

catcacatctgtatacaagatggttgagagcgataagacggcaataaaatccatgattgctaaa
aatgtagaagtgttgtgttctacatctgccaatactggattttctctgagaagaggagcattgt
catttaatacaggcgtcattgttacaaaggacgtggctatggcttgtatacgatctctaaataa
taaacaaatgttattgtttgttggagggggaaaggattacggtgaagacgacgacgacgacgac
gaagaagcagaagaagaggacgaagaaaatggtgaaaacgaagagaacaaaggtgactgtgtca
cggaaaaaaagatccctggacgaagcactaacaaggatgttggtgaagaaactaaaacaagcga
gaaaacggagggagaaagaaagggctctaagacggcaaagggaaagacggaggaaattgctagt
tcgttgagtaaatgtgggaagaaagatgcgagagatgtcattcttgaccgtttactaaaagcaa
cacattcttcttgcaccaacaatgaagagagaaccagagtcttacaacaatatagcaattgtac
attatcttcctatataacttcagtcatgaaattggaccaaagagtagcagaccaaatggaaaat
ttaatatctcaattggatcaaatacgtaatctctccaacaagaagaggcaagaaaagggagggc
cttttaagtctgaattggacgccatggttgctgcagttaaggttaagtttttcccagttttaga
tgcgtctagaaaattgactcaagaccattggaaaaagtgccccgtgtccatcccagaaacgcgt
gaagaaaaaccattaatgggtgtgccttttgaagttgcactcaattctctaataggaaaacaca
agtgcacagatacatgcgacatggcttgttgtcaatcattgtattttgtcctcttgtacacgct
agctttaaaatttgagaacgaaagattggcccggcaaattggcctagatgactctgtagatttg
atggctgagatgttgttcggaggggataaactattggcccaggaagtgttaaaaagggtaaaag
atgctcaagatagaaagttggtgaaatctttattgcctttaaattataaccatgacacaaatac
aattatatttttgtttgagtctttaaggtttgctcagaaacctgtagctggtatgagtgttagt
gaaataaaagacgctgttagaggtctggccttttctaccactacaggtactgtgtggaattata
ctgatgaaagatttttggaccattgtataacatggatgaactttgtaacgaacgtgtcaatgg
aaattgtaaattgtcctttataactggtatttatcatacggcagcagtagaattggctgctgca
tgtctatcttgtgttttgtaagaaatgataataaaaaacatgtataatttcattctggtttta
ttttatatcctcaagagttgtagtagtagcagcagcagcagcaatagcagcattattttcagcg
acagcttcttcatttctattgattgatatctggtcattttctacagcccatggtcgtttaagga
atattctttgaagcagaaacacatacccagcaatgattgcaatgatcaatattaatagtagcga
attaattaaaataagcattttgtccacattctggctaaaataaaaaagtttatctctcaatgaa
aaatttttattgaccaatcacttcgggaagttttgcagcatgacacactattactccaaaacta
tgctcactcgtatctagtcccacaacatcacgcgtcatatttaaaacatgcacctttaaagcct
gtttttcttatacaattctgtattgattatagggtttacaacctttaggccttcctttagaga
tactattttcatgtattttacaatcgaatatgagttgtcttgtgcaccagacgggagcctgtca
tgaaactctacttctctcccacgtgtattgagcgagagatcattaagcacaacttctgaagaaa
cgagaggttgtaaaatagagttatatttaggtttaattaggaaacatttgtggaaacttgaact
gttaatatcaatagaaactgagctatatgctgacgacatggcttgcattttcaaatcatgttca
aatcttactacataacccaagaaattataatctgcttcttcaccatcatcttcttccatggcat
cctctcctcttggctcttttagtacaatttcttttggttggttagtcatgttgtatatgttcat
tgagctatgatttcccacaacatcatcaaagaaaccccttctgggataatgcatgcaccgaca
gaggtgtagaataatcctccatatctaatgacaccgactgcggtagcgttctcttcatcatagt
aaccgagcttgattttcacatgactcatgggtccgatagagatattggaagccggttcagtagc
ggaaaaaatagacacttgttggaacattttgtcgtcaggaagagtctcgaataaaataccgaca
ggggaattatcttcaacactattaaagtttgttgcataagatagccgccctgaactacttgtat
tataaattgttatatcggatgctatttcagaacaaagatctcctataaaattgtgattagggca
gaccaacatcccgtctccagtgtaagacgatacatgaagtcctggtccagttggcacgaaaatg
gcagccgttcgtgaaggatttctcacgtcaactcgtctcaccttcactggtactttccaaatc

```
gtcgtggatctacattgaaactaaaatctctttcgtggggatcacttccatcctttaaagagaa
gagaggcactctgtaaacaggagaccaatctgaatagtaccttaaagctgttctatttttttct
tctagagtgggtaccgctctgcggtgcattcggtacacctccaaatccgtttcctttacttgaa
tcgattttgctccttcattttcccagagcgcatcagaatcgtacctgtcgtcgaacgaatcttc
acctcccattttatttattttcctctatatgataaaaaccacacctaaaatgtctaatatttta
ctacactgaaaaa
```

Table S2. Composition of LAMP primer evaluation reactions, Cas sgRNA evaluation reactions, one-pot SHERLOCK reactions, 10X LAMP primer mix, and sample dilutions used in one-pot SHERLOCK reactions.

a) Composition of LAMP primer evaluation reactions.

LAMP Reaction component	Initial Concentration	Final Concentration	WSSV reaction volume (µL)	TSV reaction volume (µL)
Isothermal Amp Buffer (NEB)	10x	1x	2	2
MgSO4 (NEB)	100 mM	8 mM	1.6	1.6
glycine	2 M	200 mM	2	
taurine	500 mM	50 mM		2
water			4.3	5.8
dNTPs (NEB)	10 mM	1.4 mM	2.8	2.8
WarmStart RTx Reverse Transcriptase (NEB)	15000 units/mL	300 units/mL		0.4
WarmStart Bst 2.0 (NEB)	8000 units/mL	320 units/mL	0.8	0.8
Primer mix	10x	1x	2	2
Template			4	2
SYTO-82 LAMP dye (Thermo)	100 µM	0.5 µM (1 µM for TSV)	0.1	0.2
5’ FAM-polyT(5) reporter (IDT)	100 µM	2 µM	0.4	0.4
**Total**			20	20

b) Composition of Cas sgRNA evaluation reactions.

Cas/gRNA evaluation reaction component	Initial Concentration	Final Concentration	WSSV reaction volume (µL)	TSV reaction volume (µL)
NE Buffer 2.1 (NEB)	10x	1x	1	0
Isothermal Amp Buffer (NEB)	10x	1x	0	2
MgSO4 (NEB)	100 mM	8 mM	0	1.6
Cas12b (GenScript)	8.73 µM	200 nM (250 nM for TSV)	0.229	0.570
sgRNA	5 µM (2.5 µM for TSV)	400 nM (250 nM for TSV)	0.8	2
water			4.571	7.83
Template			3	5
5' HEX-polyT(20) reporter (IDT)	5 µM	200 nM	0.4	0
5’ FAM-polyT(5) reporter (IDT)	50 µM	2.5 µM	0	1
**Total**			10	20

c) Composition of one-pot SHERLOCK reactions.

One-Pot SHERLOCK Reaction component	Initial Concentration	Final Concentration	WSSV reaction volume (µL)	TSV reaction volume (µL)
Isothermal Amp Buffer	10x	1x	2	2
MgSO4	100 mM	8 mM	1.6	1.6
glycine	2 M	200 mM	2	
taurine	500 mM	50 mM		2
Cas12b	8.73 µM	200 nM (150 nM for TSV)	0.458	0.344
sgRNA	10 µM	200 nM (600 nM for TSV)	0.4	1.2
water			3.44	2.46
dNTPs	10 mM	1.4 mM	2.8	2.8
**Incubate for 15 minutes at room temperature then add…**				
WarmStart RTx Reverse Transcriptase	15000 units/mL	150 units/mL		0.2
WarmStart Bst 2.0	8000 units/mL	320 units/mL	0.8	0.8
Primer mix	10x (20x for TSV)	1x	2	1

DNA/RNA			4	5
SYTO-82 LAMP dye	100 µM	0.5 µM (1 µM for TSV)	0.1	0.2
5' FAM-polyT(5) reporter	100 µM	2 µM	0.4	0.4
Total			20	20

d) Composition of 10X primer mix.

10x Primer Mix component (100 µM)	10x concentration	Reaction Concentration	Volume (µL)
F3	2 µM	0.2 µM	2
B3	2 µM	0.2 µM	2
BIP	16 µM	1.6 µM	16
FIP	16 µM	1.6 µM	16
LF	4 µM	0.4 µM	4
LB	4 µM	0.4 µM	4
Water			56
Total			100

e) Composition of sample dilutions used in SHERLOCK reactions.

Sample dilution component	WSSV	TSV
Sample DNA/RNA	1ng	10 ng
Pathogen free shrimp nucleic acid (DNA for WSSV, RNA for TSV)	20 ng	5 ng
water	variable	variable
Total volume/one-pot reaction	4 µL	5 µL

Table S3. Viral concentrations estimated by qPCR, SHERLOCKv1, and SHERLOCKv2 assays ('NA', not detected).

Target	Sample Name	qPCR predicted copies/µL (Mean)	qPCR predicted copies/µL (Std Dev)	SHERLOCKv1 predicted copies/µL (mean)	SHERLOCKv1 predicted copies/µL (Std Dev)	SHERLOCKv2 predicted copies/µL (Mean)	SHERLOCKv2 predicted copies/µL (Std Dev)
TSV	Val.1	9.449E+06	2.946E+04	0.000E+00	0.000E+00	3.667E+06	1.241E+06
TSV	Val.2	6.475E+02	8.442E+01	0.000E+00	0.000E+00	NA	NA
TSV	Val.4	2.040E+09	4.584E+08	0.000E+00	0.000E+00	1.715E+08	5.258E+07
TSV	Val.5	9.907E+06	3.761E+05	0.000E+00	0.000E+00	1.131E+06	1.548E+05
TSV	Val.6	2.943E+04	1.010E+03	0.000E+00	0.000E+00	4.000E+03	3.287E+03
TSV	Val.7	2.340E+03	2.518E+02	0.000E+00	0.000E+00	1.150E+02	7.981E+01
TSV	Val.8	9.453E+02	1.844E+02	0.000E+00	0.000E+00	4.200E+01	3.861E+01
TSV	Val.9	9.985E+03	4.586E+02	0.000E+00	0.000E+00	1.198E+01	1.288E+01

TSV	Val.10	4.252E+03	2.525E+02	0.000E+00	0.000E+00	2.465E+02	1.626E+02
TSV	Val.12	2.001E+01	6.875E+00	0.000E+00	0.000E+00	NA	NA
TSV	Val.13	7.932E+05	2.680E+04	0.000E+00	0.000E+00	8.302E+04	8.499E+04
TSV	Val.14	4.780E+03	1.094E+03	0.000E+00	0.000E+00	8.364E-01	8.537E-01
TSV	Val.15	1.947E+04	6.245E+02	0.000E+00	0.000E+00	5.203E+02	6.394E+02
TSV	Val.16	2.503E+05	9.313E+03	0.000E+00	0.000E+00	2.252E+05	1.353E+05
TSV	Val.18	8.489E+03	1.669E+03	0.000E+00	0.000E+00	1.435E+03	8.932E+02
TSV	Val.19	1.298E+04	2.445E+02	0.000E+00	0.000E+00	1.404E+03	7.166E+02
TSV	Val.20	4.244E+03	1.343E+03	0.000E+00	0.000E+00	4.699E+02	2.488E+02
TSV	Val.21	9.617E+07	5.850E+06	0.000E+00	0.000E+00	1.667E+07	1.775E+07
TSV	Val.22	3.308E+04	1.023E+03	0.000E+00	0.000E+00	2.837E+02	3.080E+02
TSV	Val.23	5.702E+03	2.979E+02	0.000E+00	0.000E+00	5.226E+02	1.716E+02
TSV	Val.25	8.645E+02	9.886E+01	0.000E+00	0.000E+00	NA	NA
TSV	Val.26	5.215E+06	1.170E+05	0.000E+00	0.000E+00	1.168E+06	1.211E+06
TSV	Val.28	6.022E+03	1.445E+02	0.000E+00	0.000E+00	6.070E+00	4.024E+00
TSV	Val.29	8.226E+07	3.939E+06	0.000E+00	0.000E+00	8.163E+05	2.325E+05
TSV	Val.30	1.832E+04	8.273E+01	0.000E+00	0.000E+00	1.398E+02	8.417E+01
TSV	Val.31	5.549E+03	2.953E+02	0.000E+00	0.000E+00	2.279E+02	1.174E+02
TSV	Val.32	1.044E+02	5.121E+00	0.000E+00	0.000E+00	1.046E-03	9.807E-04
TSV	Val.33	5.150E+03	5.260E+02	0.000E+00	0.000E+00	NA	NA
TSV	Val.34	3.735E+03	3.124E+02	0.000E+00	0.000E+00	4.455E+02	2.415E+02
TSV	Val.35	3.080E+07	1.027E+06	0.000E+00	0.000E+00	6.240E+06	7.671E+06
WSSV	Pvan_001	1.261E+06	4.825E+04	1.777E+06	6.257E+05	5.467E+06	1.360E+06
WSSV	Pvan_002	1.718E+06	5.008E+04	1.669E+06	5.453E+05	4.610E+06	1.110E+06
WSSV	Pvan_003	1.437E+07	4.244E+06	9.002E+06	7.179E+06	9.668E+07	6.755E+06
WSSV	Pvan_004	8.136E+05	8.335E+04	3.494E+05	1.934E+05	1.781E+06	5.254E+05
WSSV	Pvan_005	1.491E+07	9.171E+05	1.180E+07	6.902E+06	7.704E+07	9.320E+06
WSSV	Pvan_006	2.455E+05	1.066E+04	3.294E+05	5.066E+04	1.162E+06	8.754E+04
WSSV	Pvan_007	1.990E+06	6.025E+04	2.411E+06	6.014E+05	8.351E+06	3.014E+06
WSSV	Pvan_008	8.500E+06	1.241E+06	8.461E+06	9.652E+06	4.689E+07	4.589E+06
WSSV	Pvan_009	2.043E+07	2.886E+06	1.453E+07	5.325E+06	5.841E+07	1.437E+07
WSSV	Pvan_010	3.483E+07	5.419E+06	3.315E+07	5.680E+07	1.292E+09	2.086E+08
WSSV	Pvan_011	7.553E+02	1.339E+02	2.282E+03	4.922E+02	1.355E+03	1.869E+03
WSSV	Pvan_012	8.253E+02	8.739E+01	1.709E+03	7.888E+02	NA	NA
WSSV	Pvan_013	9.842E+02	5.217E+02	2.258E+03	8.400E+02	7.382E+02	5.447E+02
WSSV	Pvan_014	3.520E+02	4.074E+02	5.704E+02	1.858E+02	1.695E+02	1.682E+02
WSSV	Pvan_015	2.770E+06	6.137E+05	2.963E+06	1.853E+05	5.662E+04	3.025E+04
WSSV	Pvan_016	3.145E+06	2.963E+05	3.008E+06	1.441E+06	2.550E+04	2.349E+04
WSSV	Pvan_017	7.379E+06	4.678E+05	3.945E+06	3.114E+05	3.251E+05	3.747E+05
WSSV	Pvan_018	5.524E+06	2.616E+05	1.211E+07	2.421E+07	5.117E+04	5.783E+04
WSSV	Pvan_019	5.898E+07	1.231E+07	6.127E+07	1.210E+08	3.249E+07	9.313E+06

WSSV	Pvan_020	2.851E+05	1.344E+04	4.409E+01	1.516E+01	7.085E+03	2.078E+03
WSSV	Pvan_021	2.651E+07	4.818E+05	1.278E+07	1.324E+07	6.260E+07	1.339E+07
WSSV	Pvan_022	2.378E+06	2.214E+05	1.323E+07	2.645E+07	1.401E+05	8.987E+04
WSSV	Pvan_023	5.685E+06	8.777E+05	1.094E+07	2.152E+07	8.905E+04	4.511E+04
WSSV	Pvan_024	6.622E+05	1.041E+05	6.296E+05	1.090E+06	3.659E+02	4.427E+02
WSSV	Pvan_025	7.149E+02	1.300E+02	9.502E+03	1.892E+04	5.317E+02	4.620E+02
WSSV	Pvan_026	5.440E+01	2.210E+00	3.462E+01	1.709E+00	NA	NA
WSSV	Pvan_027	4.713E+02	2.112E+01	8.036E+03	1.563E+04	4.571E+03	6.644E+03
WSSV	Pvan_028	2.509E+07	7.114E+05	4.033E+07	6.985E+07	3.000E+07	1.250E+07
WSSV	Pvan_029	3.811E+01	7.100E-01	3.440E+01	1.733E+00	NA	NA
WSSV	Pvan_030	4.017E+02	1.276E+02	7.409E+02	1.216E+03	1.842E+03	3.875E+02
WSSV	Pvan_031	5.157E+07	1.085E+07	5.494E+07	5.642E+07	6.302E+07	2.063E+07
WSSV	Pvan_032	3.280E+05	3.823E+04	1.353E+05	1.617E+05	1.325E+05	4.616E+04
WSSV	Pvan_033	4.521E+07	1.392E+07	2.271E+07	1.533E+07	1.521E+08	4.032E+07
WSSV	Pvan_034	2.561E+06	4.552E+05	2.179E+06	3.746E+06	1.760E+04	1.003E+04
WSSV	Pvan_035	6.993E+06	8.791E+05	5.778E+06	9.374E+06	1.386E+04	6.544E+03
